# Research trends and hotspots of acupuncture for knee osteoarthritis from 2004 to 2024: a bibliometric analysis

**DOI:** 10.3389/fmed.2025.1604209

**Published:** 2025-06-10

**Authors:** Jun Zhou, Kuayue Zhang, Fanying Zhao, Xueyan Liu, Jiarun Zhang, Fang Yuan, Lu Liu, Bin Li

**Affiliations:** ^1^Center of Acupuncture and Moxibustion, Beijing Hospital of Traditional Chinese Medicine, Capital Medical University, Beijing, China; ^2^Beijing Key Laboratory of Digital Intelligent Acupuncture, Beijing, China; ^3^Beijing Key Laboratory of Acupuncture Neuromodulation, Beijing, China; ^4^Beijing University of Chinese Medicine, Beijing, China

**Keywords:** acupuncture, knee osteoarthritis, bibliometrics, VOSviewer, CiteSpace

## Abstract

**Background:**

Knee osteoarthritis (KOA) is a leading cause of pain and disability worldwide. Acupuncture has emerged as a prominent non-pharmacological treatment for KOA. This study aims to analyze the general research status, hotspots, and trends of acupuncture in the treatment of KOA.

**Methods:**

On January 19, 2025, a comprehensive search was conducted in the Web of Science Core Collection (WoSCC) for all available literature related to acupuncture and KOA. VOSviewer and CiteSpace software were employed for bibliometric analysis.

**Results:**

A total of 295 publications were retrieved, covering 26 countries, 133 institutions, and 107 journals, with contributions from 1,711 authors. Since 2015, the number of publications on acupuncture for KOA has seen a rapid increase, indicating growing global interest in acupuncture as a potential treatment for KOA. China contributed 73.56% of the research, followed by the United States (10.17%) and the United Kingdom (6.44%). China, the U.S., and the U.K. maintain close academic collaborations, especially between China and the U.S. Beijing University of Chinese Medicine published the most articles (53), but cross-institutional collaboration remains limited. The journal *Medicine* published the most papers, while *Osteoarthritis and Cartilage* was the most cited (470 times). Key researchers such as Jianfeng Tu (19 papers), Cunzhi Liu (17 papers), and Liqiong Wang (15 papers), focused on comparing electroacupuncture and traditional acupuncture for KOA. Brian M Berman, the most cited researcher (106 times), made significant contributions to electroacupuncture research. Keyword analysis revealed chronic pain, analgesia, randomized controlled trial (RCT), and meta-analysis as key themes, with electroacupuncture emerging as the current research hotspot.

**Conclusion:**

With the support of high-quality randomized controlled trial, acupuncture is increasingly recognized as an effective treatment for KOA. Future research should focus on standardizing treatment protocols and determining the optimal dosage and frequency of acupuncture to maximize clinical efficacy.

## 1 Introduction

Osteoarthritis (OA) is a prevalent degenerative disease, causing significant personal suffering and disability (1). It is the most common cause of activity limitations in adults and the leading cause of deformity among the aging population (2). Among OA types, knee osteoarthritis (KOA) is the most prevalent, accounting for approximately 85% of the overall burden, and affecting 3.8% of the global population (3, 4). KOA is a multifactorial disease, characterized by pathological changes such as cartilage degeneration, osteophyte formation, remodeling of the osteochondral unit, and joint inflammation (5). Epidemiological studies and risk factor analyses of KOA have identified several major contributors, including advanced age, female gender, body mass index (BMI), ethnicity, history of knee joint injury, physical labor, family history of KOA, and environmental factors (6, 7). Moreover, increasing life expectancy, an aging population, and rising obesity rates exacerbate the clinical and economic impact of KOA, posing significant public health challenges (8). Pain from KOA significantly reduces physical function and quality of life (QOL), increasing the risk of all-cause mortality (9). Alleviating pain remains a primary goal in the management of KOA, aimed at enhancing patient function and QOL (10).

Pain in KOA arises from inflammatory, mechanical, and neuropathic mechanisms, necessitating tailored management strategies (5). Inflammatory pain is treated with non-steroidal anti-inflammatory drugs (NSAIDs) and corticosteroid injections. Mechanical pain is managed through interventions that reduce joint stress, such as physical therapy, weight management, and assistive devices. Neuropathic pain is addressed with gabapentinoids, antidepressants, or radiofrequency ablation (11). Advanced regenerative treatments, including platelet-rich plasma and stem cell therapies, show potential in addressing mixed-source pain (12, 13). However, the widespread use of opioids and NSAIDs is limited by gastrointestinal, cardiovascular, and renal complications, restricting their clinical application (14). Long-term adherence to exercise or weight loss regimens proves challenging for certain KOA patients. Given this context, there is an urgent need for safe and effective treatments that provide immediate benefits to patients. Current guidelines emphasize non-pharmacological interventions, such as patient education, weight management, exercise therapy, physical therapy, and traditional Chinese medicine (TCM) non-pharmacological therapies (15, 16).

TCM non-pharmacological therapies refer to external treatment methods guided by the principles of traditional Chinese medical theory. Numerous studies support the effectiveness of TCM non-pharmacological therapies intervention in KOA, including acupuncture, Tai Chi, and moxibustion. These therapies have been shown to improve pain, depression, and sleep quality, as well as reduce surgery rates and alleviate economic burdens on patients (17–20). Acupuncture has long been considered a potentially effective non-pharmacological treatment for KOA. Both the American College of Rheumatology (ACR) and the American Academy of Orthopedic Surgeons (AAOS) have explicitly recommended acupuncture as a treatment option for knee OA (15, 16). A meta-analysis on acupuncture conducted in 2025 found that patients receiving acupuncture showed clinically significant improvements in their primary health issues and symptoms, and these improvements appeared to persist in the medium to long term (21). Acupuncture is increasingly studied as a potentially effective intervention for alleviating mild-to-moderate knee OA, and may help reduce the associated healthcare burden. Currently, over 183 countries and regions employ acupuncture, motivated by an increasing body of evidence supporting its efficacy. However, only a few countries (including the UK, Germany, the US, China, and Switzerland) cover acupuncture through public or private health insurance (22).

In recent years, numerous randomized controlled trial (RCT) of acupuncture for KOA have been published in top international journals, such as Arthritis & Rheumatology and Pain, demonstrating that acupuncture can improve pain and joint function, offering long-term benefits (23, 24). Acupuncture acts through multiple pathways, including modulation of inflammation, bone metabolism, and neuroimmunological responses, thereby improving KOA symptoms and alleviating joint damage (25, 26). In 2023, Xu et al. developed a novel needle-based acupuncture technology (nd-Acu), which has shown to effectively alleviate pain, reduce inflammation, and slow the progression of KOA at biochemical and histological levels (27). Despite the growing body of evidence supporting these treatments, systematic evaluation is still required to better understand their clinical efficacy, underlying mechanisms, and optimal treatment protocols.

Bibliometric analysis plays a critical role in advancing research in the medical field by identifying research trends, measuring the impact of findings, mapping academic networks and collaborations, uncovering research gaps, and providing scientific evidence for policy-making (28). Recent bibliometric studies, such as the one by Li et al., focused on acupuncture for KOA from 2010 to 2019 (29); however, no similar analysis has been conducted for the past 20 years. With an increasing number of high-quality studies published recently, a bibliometric investigation is warranted to capture the latest research trends. This study aims to systematically analyze the recent literature on acupuncture for KOA using CiteSpace and VOSviewer, exploring the current state, hotspots, and emerging trends, and providing guidance for future research directions.

## 2 Materials and methods

### 2.1 Literature sources and search strategy

**FIGURE 1:**
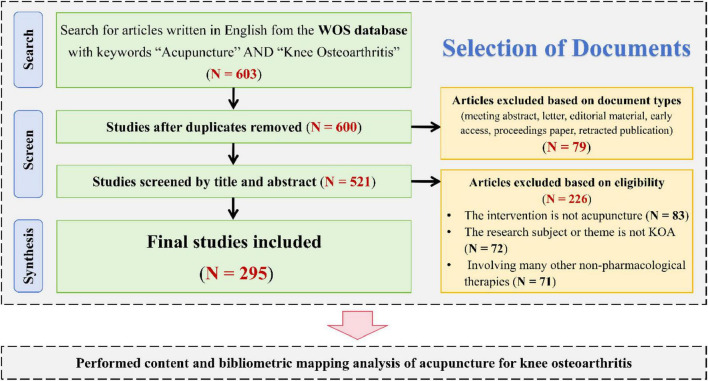
Flow chart of document retrieval and screening.

This study uses the Web of Science Core Collection (WoSCC) database as the data source. The search strategy is as follows: TS = [(“acupuncture” OR “acupoint” OR “acupuncture point” OR “acupuncture therapy” OR “acupuncture treatment” OR “Pharmacoacupuncture” OR “manual acupuncture” OR “auricular acupuncture” OR “ear acupuncture” OR “warm acupuncture” OR “body acupuncture” OR “electroacupuncture” OR “electro-acupuncture” OR “electrothermal acupuncture” OR “acupoint catgut embedding” OR “embedding thread” OR “acupoint thread-embedding” OR “fire needling” OR “Acupotomy”) AND TS = (“Osteoarthritis, Knee” OR “Knee Osteoarthritis” OR “Osteoarthritis of knee” OR “osteoarthritis of the knee” OR “knee osteoarthritides” OR “KOA”)] AND “publication year”: 1985–2025 and “language:” English. To ensure consistency, we limited the article types to “articles” and “reviews.” All literature was searched and downloaded in plain text format within 1 day, on January 19, 2025. After obtaining the search results, we first excluded duplicates, and then further excluded articles unrelated to the topic by reading titles and abstracts. The specific exclusion criteria were: (1) interventions not involving acupuncture; (2) research subjects or themes unrelated to KOA; (3) guidelines, reviews, or meta-analyses that did not consider acupuncture as an independent subject of investigation, and in which acupuncture was only briefly mentioned as an adjunctive component of non-pharmacological interventions in the main text. This screening process was conducted independently by two researchers, with any disagreements resolved by a third expert. Publications meeting the criteria would be included in this analysis, then a total of 295 target documents were obtained. The search and screening process is illustrated in Figure 1. The search results are shown in Supplementary material 1.

### 2.2 Visualization and statistical analyses

CiteSpace (Version 6.4.R1) was used for data collection, organization, and visual analysis, including analysis of institutions, authors, and keywords. VOSviewer (Version 1.6.20) was employed to explore the network relationships between keywords and citations. The temporal partition setting for CiteSpace was set from 2004 to 2025, with time slices configured as “1.” The g-index was set to the default value of 25 in CiteSpace. This value is used to select literature nodes with higher citation frequencies in each time slice, which helps to balance the inclusion of highly cited papers while limiting the interference caused by low-impact articles. In terms of network pruning methods, we applied Pathfinder to eliminate redundant edges and enhance the clarity of the network structure. Pruning the Merged Network was used to simplify the overall network, facilitating a clearer presentation of the macro-level structure. Pruning Sliced Networks was employed to highlight the core structures within each time slice and ensure consistency across different temporal segments.

## 3 Results

### 3.1 Annual publications analysis

**FIGURE 2:**
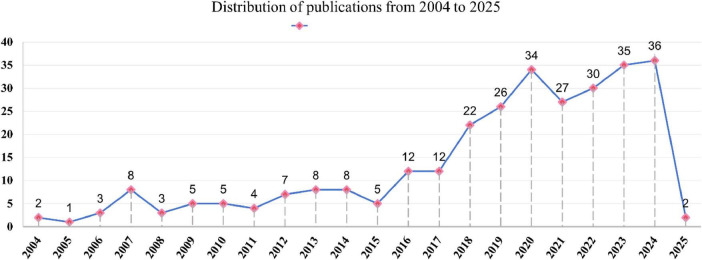
Distribution of publications from 2004 to 2025.

This study ultimately included 295 articles for analysis. The first article in this field, retrievable from WoSCC, was published on November 18, 2004 (30). Figure 2 illustrates the number of publications in this field from 2004 to January 19, 2025, by year. From the line chart, it is evident that the field was in its nascent stage between 2004 and 2006, with fewer than three publications per year. From 2007 to 2015, the field entered an exploratory phase, with an increasing number of publications, stabilizing at 5–8 per year. Since 2016, there has been rapid development in this field, with a sharp rise in the number of publications per year, showing a generally upward, fluctuating trend, indicating growing researcher interest in this area.

### 3.2 Countries analysis

**FIGURE 3:**
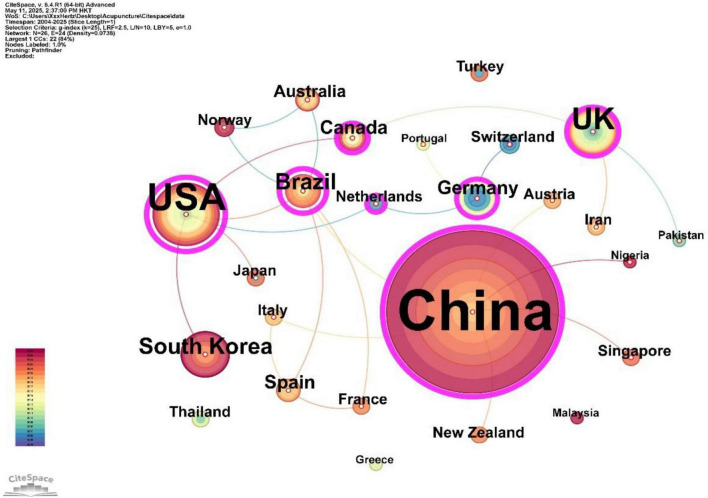
The co-occurrence maps of countries.

A total of 26 countries have contributed to the publication of articles in this field. The co-occurrence map of country collaborations (Figure 3) was generated using CiteSpace, and the intermediary centrality of different countries was calculated. The size of the nodes on the map is proportional to the number of publications, and the links between the nodes represent the strength of their collaborative relationships. As shown in Table 1, China, the birthplace of acupuncture, is the core contributor to this field with 217 publications, accounting for 73.56% of the total research, and an intermediary centrality of 0.68. These publications have been cited 2,004 times. The United States follows with 30 publications (10.17%), an intermediary centrality of 0.54, and 1,458 citations. The United Kingdom published 19 papers (6.44%) with an intermediary centrality of 0.28 and 1,121 citations. South Korea also published 19 papers (6.44%), with an intermediary centrality of 0.01 and 131 citations. In summary, China, the United States, and the United Kingdom play significant roles in this field and maintain close collaborative relationships.

**TABLE 1:** Top 10 countries in terms of the number of publications and citation.

Rank	Country	Time of first publication	Publications	Citations	Centrality
1	China	2007	217	2,004	0.68
2	USA	2004	30	1,458	0.54
3	South Korea	2013	19	131	0.01
4	UK	2007	19	1,121	0.28
5	Germany	2005	8	1,075	0.14
6	Brazil	2007	8	287	0.11
7	Spain	2004	5	212	0.02
8	Australia	2007	5	240	0.01
9	Canada	2011	4	142	0.03
10	Switzerland	2005	3	479	0

### 3.3 Institutions analysis

A total of 433 institutions have contributed to the research in this field, with 22 institutions publishing five or more papers. Table 2 presents the top 10 institutions by publication count. Among these, nine are from China, with Beijing University of Chinese Medicine (*n* = 53), Capital Medical University (*n* = 28), and Guangzhou University of Chinese Medicine (*n* = 18) ranking as the top three institutions in terms of publication volume. The top 20 institutions by betweenness centrality are listed in Supplementary material 2, with the top five being: Beijing University of Chinese Medicine (0.13), Capital Medical University (0.09), Korea Institute of Oriental Medicine (0.08), Guangzhou University of Chinese Medicine (0.06). These institutions play a crucial role in collaborative development within this field, with universities and colleges serving as dominant forces, while hospital participation remains relatively limited. However, the highest centrality score among institutions in this field reaches only 0.13, suggesting an overall lack of cross-institutional collaboration in the domain.

**TABLE 2:** Top 10 institutions in terms of the number of publications and citations.

Rank	Organizations	Time of first publication	Publications	Citations	Centrality
1	Beijing University of Chinese Medicine	2017	53	572	0.13
2	Capital Medical University	2016	28	389	0.09
3	Guangzhou University of Chinese Medicine	2009	18	61	0.06
4	China Academy of Chinese Medical Sciences	2011	17	315	0.07
5	Chengdu University of Traditional Chinese Medicine	2018	16	227	0.06
6	Shanghai University of Traditional Chinese Medicine	2009	15	104	0.06
7	Fujian University of Traditional Chinese Medicine	2010	12	136	0
8	Korea Institute of Oriental Medicine	2020	10	36	0.08
9	Sichuan University	2013	9	89	0
10	Huazhong University of Science and Technology	2015	8	168	0.02

### 3.4 Journals and co-cited journals analysis

**FIGURE 4:**
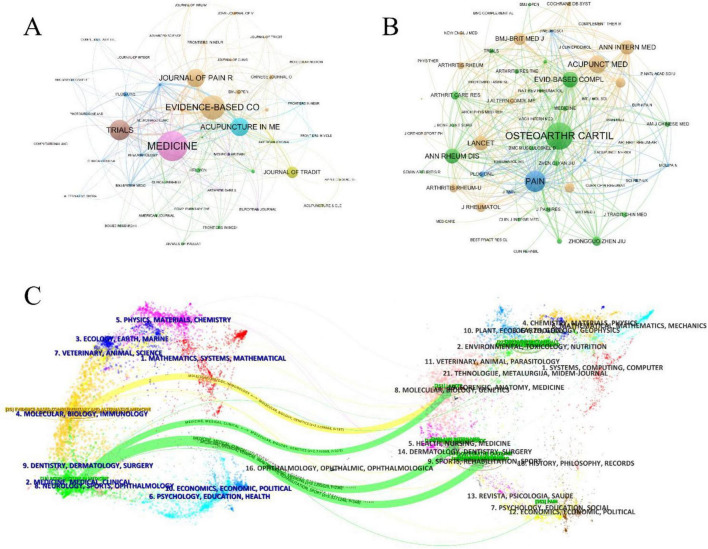
Visual analysis of Journals. **(A)** Co-occurrence maps for journals. **(B)** Co-occurrence maps for Co-Cited Journals. **(C)** Double image overlay for journals.

A total of 107 journals have published research related to this field. Among them, *Medicine* (IF = 1.4/Q2) is the most prolific, with 31 articles, followed by *Evidence-based Complementary and Alternative Medicine* (IF = 2.65/Q3) with 25 publications, and *Trials* (IF = 2.0/Q3) with 21 publications (Table 3). Figure 4A presents the co-occurrence map of journals. Each node represents a journal, with node size indicating the number of publications, and edge thickness denoting the strength of co-occurrence links between journals. The colors reflect clusters identified by VOSviewer’s modularity-based clustering algorithm. The journals that are centrally located and highly active include Medicine, Evidence-Based Complementary and Alternative Medicine, Acupuncture in Medicine, and Trials, indicating its dominant role in publishing research on acupuncture for KOA. They have formed a close co-occurrence relationship, reflecting a high degree of overlap in research topics and disciplinary fields among them. The blue and green clusters mainly involve comprehensive medical journals, while the yellow one represents the fields of pain research and neuroimaging. The positions of *Journal of Pain Research*, *BMJ Open*, and *PLOS One* at intersecting locations suggest their function as interdisciplinary bridges between core clinical, pain, and integrative medicine journals.

**TABLE 3:** Top 10 journals in terms of number of publications and citations.

Rank	Journals	Publications	Rank	Journals	Cited
1	Medicine	31	1	Osteoarthritis & Cartilage	470
2	Evidence-based Complementary and Alternative Medicine	25	2	Pain	364
3	Trials	21	3	Acupuncture in Medicine	304
4	Acupuncture in Medicine	19	4	Annals of Internal Medicine	243
5	Journal of Pain Research	19	5	Lancet	251
6	Journal of Traditional Chinese Medicine	12	6	Evidence-based Complementary And Alternative Medicine	273
7	PLoS One	5	7	Annals of the Rheumatic Diseases	256
8	Chinese Journal of Integrative Medicine	5	8	BMJ-British Medical Journal	214
9	BMJ Open	5	9	Journal of Rheumatology	164
10	Heliyon	5	10	JAMA-Journal of the American Medical Association	164

The field currently involves 2,717 co-cited journals. The most frequently cited journal is *Osteoarthritis and Cartilage* (IF = 7.2/Q1), with 470 citations, followed by *Pain* (IF = 5.9/Q1) with 364 citations, and *Acupuncture in Medicine* (IF = 2.4/Q2), as shown in Table 3. Figure 4B illustrates the co-citation relationships among journals that are frequently cited together in the literature on acupuncture for KOA. *Osteoarthritis and Cartilage* is the journal with the highest co-citation frequency, indicating its authoritative position in the research fields of basic mechanisms and clinical efficacy of KOA. Other journals with high co-citation include *Pain*, *Annals of the Rheumatic Diseases*, *The Lancet*, and *Arthritis and Rheumatism*, reflecting the evidence base drawn from pain science, rheumatology, and high-impact clinical research. The presence of high-impact general medical journals such as *BMJ*, *NEJM*, and *Cochrane Database of Systematic Reviews* underscores the growing evidence synthesis in acupuncture studies. The yellow and green clusters focus on the combined Chinese and Western treatment of arthritis, including osteoarthritis and rheumatoid arthritis, with an emphasis on pathogenesis, research advancements, and efficacy evaluations of integrative therapies. These citations aim to summarize current findings, highlight issues and gaps, and provide theoretical support for acupuncture in treating osteoarthritis. The blue cluster primarily includes pain-related journals, which focus on pain pathogenesis and management methods. Citations from these articles primarily explain the principles and efficacy of acupuncture in pain relief.

Figure 4C presents the dual-overlay map of journals, providing a macro-level visualization of citation trajectories between citing and cited journals, suggesting that the knowledge foundation of this research area encompasses diverse disciplines such as clinical medicine, biology, rehabilitation science, and molecular genetics. The left cluster represents the distribution fields of the published journals, while the right cluster indicates the distribution fields of the cited journals. Curved lines connecting both sides represent citation paths, highlighting cross-domain citation relationships. The vertical axis of each ellipse on the left indicates the volume of publications, while the horizontal axis reflects the number of authors contributing to that domain. As shown in Figure 4C, there are four major citation paths in the journal data set of this field. The most dominant path is the green path, which illustrates a major knowledge flow from journals in medicine, clinical health, and rehabilitation to those in molecular biology and genetics. Influential journals cited along this trajectory include *Osteoarthritis and Cartilage*, *Annals of Internal Medicine*, and *The Lancet*, highlighting the integration of evidence-based clinical studies and basic biological research. The yellow citation path reflects citations from clinical and integrative medicine journals to broad-scope multidisciplinary journals such as *PLoS One*, which provide molecular, biological, and genetic background knowledge for advanced research in acupuncture mechanisms. These citation flows demonstrate that acupuncture research in KOA not only relies on traditional medical literature but also engages with high-impact biomedical and genetic science domains, underscoring its evolution toward mechanistic rigor and global scholarly integration.

### 3.5 Authors and cited authors analysis

**FIGURE 5:**
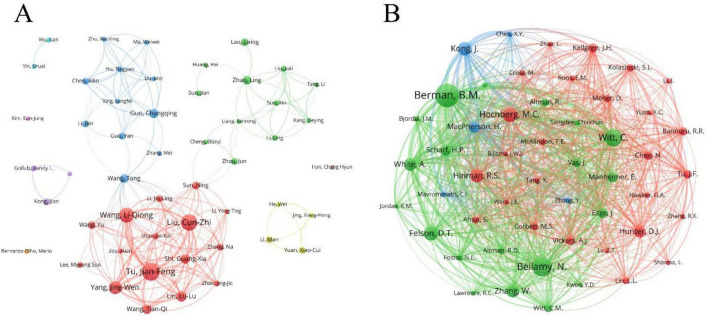
Visual analysis of author. **(A)** Map of author collaboration network; **(B)** collaborative network map of cited authors. Node size represents author frequency, and lines indicate co-occurrence relationships between authors.

In the field of acupuncture treatment for KOA, a total of 1,711 authors have contributed to related publications. Table 4 presents the top 10 authors by number of publications and the top 10 authors by citation count. Jianfeng Tu is the highest-producing author in this field, having published 19 articles. He is followed by Cunzhi Liu (*n* = 17) and Liqiong Wang (*n* = 15), who are part of the same research team. Their research focuses on the clinical efficacy evaluation and comparative analysis of electroacupuncture and traditional hand acupuncture in the treatment of KOA. We used VOSviewer to map the collaboration network of core authors with four or more publications (Figure 5A). It is evident that several collaborative teams have formed in this field, with Cunzhi Liu, Tong Wang, Changqing Guo, and Ling Zhao at the center. To explore co-citation relationships, we constructed a co-authorship network with authors having over 20 co-citations (Figure 5B). The research by Brian M. Berman is the most frequently cited (co-citations = 106), focusing primarily on acupuncture (especially electroacupuncture) and herbal medicine in the treatment of various musculoskeletal disorders. Additionally, Bellamy N. (co-citations = 88) and C. Witt (co-citations = 80) are also prominent authors in citation counts, highlighting their significant impact in this field.

**TABLE 4:** Top 10 authors in terms of number of publications and citations.

Rank	Author	Publications	Rank	Author	Cited
1	Jianfeng Tu	19	1	Brian M. Berman	106
2	Cunzhi Liu	17	2	Bellamy N	88
3	Liqiong Wang	15	3	C. Witt	80
4	Jingwen Yang	13	4	Marc C. Hochberg	67
5	Lulu Lin	10	5	Felson DT	66
6	Tianqi Wang	9	6	Jian Kong	62
7	Guangxia Shi	9	7	R. S. Hinman	60
8	Tong Wang	8	8	Adrian White	58
9	Ling Zhao	8	9	Wei Zhang	57
10	Yu Wang	7	10	David J. Hunter	53

### 3.6 Co-cited references analysis

**FIGURE 6:**
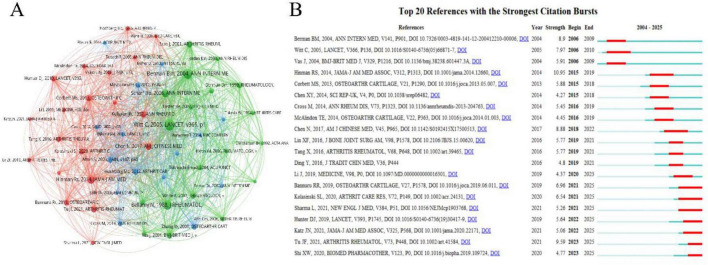
Visual analysis of Co-Cited References. **(A)** Visualization map of co-cited references network, node size represents keyword frequency, and lines indicate co-occurrence relationships between references; **(B)** top 20 keywords with the strongest citation bursts and their burst time periods.

In the field of acupuncture for knee osteoarthritis (KOA), there are 7,813 co-cited references. We have visualized the articles cited at least 15 times (Figure 6A). Table 5 lists the top 10 most-cited references. The most frequently cited article is “Acupuncture in patients with osteoarthritis of the knee: a randomized trial” by Witt C., published in The Lancet (co-citations = 80) (31). This randomized controlled trial (RCT) demonstrated that acupuncture significantly improved joint function in KOA patients, marking a significant breakthrough in the international acceptance of acupuncture. Among the top 10 co-cited articles, 5 are RCTs (23, 31–34) and 2 are meta-analyses (35, 36). These 7 articles provide strong evidence supporting the efficacy of acupuncture in treating KOA.

**TABLE 5:** Top 10 co-cited references.

Rank	Co-cited references	Cited
1	Witt C, Brinkhaus B, Jena S, et al. Acupuncture in patients with osteoarthritis of the knee: a randomized trial. Lancet. 2005;366(9480):136–143. 10.1016/S0140-6736(05)66871-7	80
2	Berman BM, Lao L, Langenberg P, Lee WL, Gilpin AM, Hochberg MC. Effectiveness of acupuncture as adjunctive therapy in osteoarthritis of the knee: a randomized, controlled trial. Ann Intern Med. 2004;141(12):901–910. 10.7326/0003-4819-141-12-200412210-00006	68
3	Bellamy N, Gilbert JR, Brooks PM, Emmerson BT, Campbell J. A survey of current prescribing practices of antiinflammatory and urate lowering drugs in gouty arthritis in the province of Ontario. J Rheumatol. 1988;15(12):1841–1847.	60
4	Scharf HP, Mansmann U, Streitberger K, et al. Acupuncture and knee osteoarthritis: a three-armed randomized trial. Ann Intern Med. 2006;145(1):12–20. 10.7326/0003-4819-145-1-200607040-00005	48
5	Hinman RS, McCrory P, Pirotta M, et al. Acupuncture for chronic knee pain: a randomized clinical trial. JAMA. 2014;312(13):1313–1322. 10.1001/jama.2014.12660	47
6	Chen N, Wang J, Mucelli A, Zhang X, Wang C. Electro-Acupuncture is Beneficial for Knee Osteoarthritis: The Evidence from Meta-Analysis of Randomized Controlled Trials. Am J Chin Med. 2017;45(5):965–985. 10.1142/S0192415X17500513	40
7	Kellgren JH, Lawrence JS. Radiological assessment of osteo-arthrosis. Ann Rheum Dis. 1957;16(4):494–502. 10.1136/ard.16.4.494	36
8	Corbett MS, Rice SJ, Madurasinghe V, et al. Acupuncture and other physical treatments for the relief of pain due to osteoarthritis of the knee: network meta-analysis. Osteoarthritis Cartilage. 2013;21(9):1290–1298. 10.1016/j.joca.2013.05.007	35
9	Hochberg MC, Altman RD, April KT, et al. American College of Rheumatology 2012 recommendations for the use of non-pharmacologic and pharmacologic therapies in osteoarthritis of the hand, hip, and knee. Arthritis Care Res (Hoboken). 2012;64(4):465–474. 10.1002/acr.21596	33
10	Tu JF, Yang JW, Shi GX, et al. Efficacy of intensive acupuncture versus sham acupuncture in knee osteoarthritis: a randomized controlled trial. Arthritis Rheumatol. 2021;73(3):448–458. 10.1002/art.41584	33

Figure 6B presents the top 20 most cited references with citation bursts. The article with the highest burst intensity is “Effectiveness of acupuncture as adjunctive therapy in osteoarthritis of the knee: a randomized, controlled trial” by Berman BM, published in Annals of Internal Medicine (32). This study confirmed that patients receiving acupuncture treatment showed better improvements in knee pain and function compared to those who received health education and sham acupuncture. Notably, among the 7 citations that burst between 2021 and 2025, two refer to the latest KOA treatment guidelines (15, 37), and three are recent KOA reviews (38–40), indicating a surge in interest and rapid development in this area following acupuncture’s recommendation in international KOA guidelines. Additionally, one randomized controlled trial (RCT) focused on enhanced acupuncture, highlighting increasing attention on the role of dosage in acupuncture efficacy (23). Another study explores the mechanisms of electroacupuncture in treating KOA in a rabbit model, providing mechanistic insights for electroacupuncture as a treatment for KOA (41).

### 3.7 Keywords analysis

**FIGURE 7:**
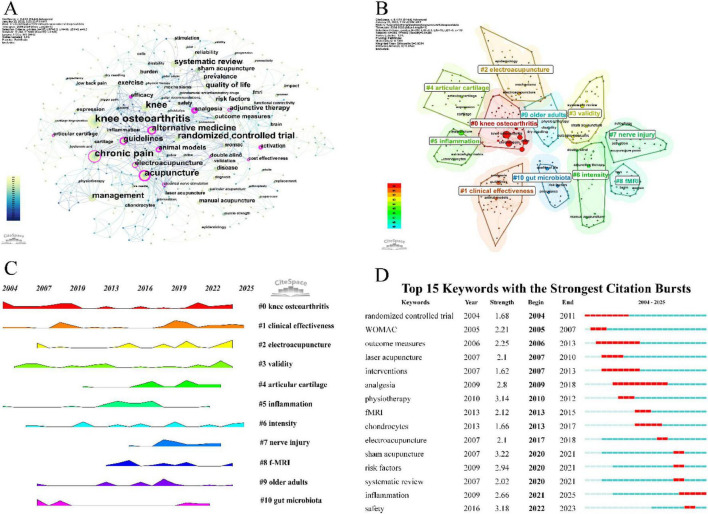
Visual analysis of Keywords. **(A)** Visualization map of keywords, node size represents keyword frequency, and lines indicate co-occurrence relationships between keywords. **(B)** Cluster map of keywords, different colors represent different thematic clusters. **(C)** Time evolution of keywords cluster, shows changes in the prominence of each thematic cluster over time. **(D)** Top 15 keywords with the strongest citation bursts and their burst time periods.

Keywords are a concentrated extraction of the article’s content, and analyzing these keywords helps explore the hotspots and frontier research within the field. Table 6 presents the top 15 keywords based on their frequency and centrality rankings. Figure 7A shows the co-occurrence relationships of keywords that appeared more than three times. The size of the nodes reflects the frequency of keyword appearances, while the thickness of the connecting lines in the network indicates the strength of the relationships between keywords. The top keywords ranked by both frequency and betweenness centrality, offering insights into research focuses and structural importance. Among the most frequent terms, “knee osteoarthritis” (*N* = 188) “knee” (*N* = 98) and “acupuncture” (*N* = 82) reflect the core topic of our bibliometric study. These keywords also possess high centrality scores, underscoring their pivotal role in connecting various research subfields. The keywords “chronic pain” (*N* = 82) and “analgesia” (Centrality = 0.43) together highlight pain management as a central theme in acupuncture research for KOA. Increasing efforts have been devoted to elucidating the mechanisms and clinical effectiveness of acupuncture-induced analgesia. Likewise, the frequent appearance of “randomized controlled trial” (*N* = 77) and “systematic review” (*N* = 43) indicates the long-standing focus on evaluating acupuncture’s efficacy and safety through rigorous clinical study designs. The keywords “management” (*N* = 57) and “guidelines” (Centrality = 0.30), both appearing frequently and centrally, signify the growing international recognition of acupuncture in clinical practice. Their presence reflects the inclusion of acupuncture in numerous clinical guidelines and expert consensus documents, which has contributed to the development and standardization of treatment protocols. “Alternative medicine” (*N* = 72) and “adjunctive therapy” (Centrality = 0.18) are also among the top-ranking terms by frequency and centrality, emphasizing acupuncture’s well-established role as a complementary therapy. While acupuncture is increasingly adopted in mainstream medicine, many studies still examine its use as an adjunctive treatment alongside pharmacological or physical interventions. The prominence of “animal models” points to the considerable volume of preclinical research focused on elucidating the underlying biological mechanisms of acupuncture for KOA. Meanwhile, “quality of life,” another frequently cited keyword, reflects the research interest in patient-centered outcomes such as pain reduction, functional improvement, and long-term quality-of-life gains. “Electroacupuncture” ranks highly in both frequency and centrality, confirming its status as a research hotspot and a key branch of modern acupuncture techniques. Clinical trials have increasingly demonstrated electroacupuncture’s advantages over manual acupuncture in pain relief and functional outcomes. As shown in Figure 7A, other specialized acupuncture modalities include laser acupuncture (*N* = 8), auricular acupuncture (*N* = 4), dry needling (*N* = 4), and fire needle (*N* = 3), all of which have emerged as focused areas of study. Additionally, the keyword sham acupuncture, though relatively low in frequency, holds moderate centrality, reflecting its essential role as a control intervention in clinical trials and its evolution into a relatively mature methodological tool.

**TABLE 6:** Top 15 keywords in terms of number of publications and centrality.

Rank	Keywords	Counts	Rank	Keywords	Centrality
1	Knee osteoarthritis	188	1	Analgesia	0.43
2	Chronic pain	131	2	Guidelines	0.30
3	Knee	98	3	Acupuncture	0.27
4	Acupuncture	82	4	Efficacy	0.24
5	Randomized controlled trial	77	5	Animal models	0.23
6	Alternative medicine	72	6	Alternative medicine	0.20
7	Management	57	7	Electroacupuncture	0.20
8	Guidelines	46	8	Activation	0.20
9	Systematic review	43	9	Chronic pain	0.18
10	Quality of life	31	10	Adjunctive therapy	0.18
11	Electroacupuncture	30	11	Double blind	0.17
12	Adjunctive therapy	27	12	Cost effectiveness	0.16
13	Animal models	24	13	Articular cartilage	0.11
14	Prevalence	22	14	Electrical nerve stimulation	0.11
15	Sham acupuncture	21	15	fMRI	0.10

This study employs CiteSpace for keyword clustering (Figure 7B), with the clustering sequence determined by the number of keywords within each cluster. The largest cluster is labeled as #0, and the analysis generated 11 clusters. Cluster #0 reflects the main topic of the study, with the primary patient population being middle-aged and elderly individuals (#9). Clusters #1 and #3 highlight the focus on clinical efficacy research within this field. Cluster #2 identifies electro-acupuncture as the most studied therapeutic modality. Clusters #4, #5, and #7 emphasize research on the mechanisms of acupuncture, particularly its effects on joint cartilage degeneration, local and systemic inflammation, and peripheral and central nerve damage. Cluster #6 indicates that dose-effect relationships are a key research focus in this area, with previous findings suggesting enhanced efficacy with intensified acupuncture. Cluster #8 shows that chronic pain in knee osteoarthritis (KOA) involves central sensitization and changes in relevant brain regions, with resting state functional magnetic resonance imaging (fMRI) technology being successfully applied to reveal the central mechanisms of acupuncture in treating KOA. Cluster #10 demonstrates that some studies have identified the correlation between gut microbiota and the development of KOA, confirming that acupuncture exerts its therapeutic effect by modulating gut flora. The frequency peaks of keywords within each cluster over time are shown in Figure 7C.

Keyword burst analysis includes two attributes: burst intensity and duration, which reflect the rapid changes of keywords over time and are helpful in understanding the developmental process of the field. We used Citespace to analyze the top 15 keywords with the highest burst intensity (Figure 7D). The keywords “RCT” (Strength = 1.68) and “analgesia” (Strength = 2.80) were the earliest and had the longest duration of bursts in this field. The “Western Ontario and McMaster Universities Osteoarthritis Index (WOMAC)” (Strength = 2.21) was the most commonly used efficacy assessment tool in the early phase of the field. The keyword with the highest burst intensity, “sham acupuncture,” began to emerge in 2020 (Strength = 3.22). In terms of specific acupuncture therapies, “laser acupuncture” (Strength = 2.10) was researched relatively early, and “electroacupuncture” (Strength = 2.10) became a research hotspot starting from 2017. In recent years, there has been a notable increase in systematic reviews (Strength = 2.02) in this field, providing higher-quality evidence for the efficacy of acupuncture in KOA. These reviews focus on the differences in efficacy between various acupuncture therapies for KOA and examine the risk factors (Strength = 2.94) influencing efficacy. Additionally, the role and efficacy of acupuncture in inflammation (Strength = 2.66) has become a recent research hotspot in this field.

## 4 Discussion

### 4.1 The general status of acupuncture intervention in KOA research

This study conducted a comprehensive bibliometric analysis of acupuncture treatment for KOA by reviewing literature from 2004 to 2024. The analysis included 295 publications from 133 institutions across 26 countries and 107 journals, with contributions from 1,711 authors. Results indicated a rapid increase in related publications since 2015, reflecting growing global interest in acupuncture as a potential treatment for KOA. China emerged as the primary contributor, responsible for 73.56% of the total articles, followed by the United States (10.17%) and the United Kingdom (6.44%). These three countries play a pivotal role in this field and maintain close collaborative ties. Co-authorship network analysis revealed strong cooperation between China and the United States, highlighting their extensive academic collaboration in advancing acupuncture research for KOA.

In terms of institutions, Beijing University of Chinese Medicine published the highest number of articles (53 publications), although minimal inter-institutional collaboration was observed. The China Academy of Chinese Medical Sciences, on the other hand, played a significant role in fostering collaborative developments. *Medicine* was the journal with the most publications (31 publications), while *Osteoarthritis and Cartilage* garnered the highest citation count (470 citations). These journals have played a critical role in reporting acupuncture research for KOA. Prominent authors include Jianfeng Tu (*n* = 19), Cunzhi Liu (*n* = 17), and Liqiong Wang (*n* = 15), all of whom are affiliated with the Beijing University of Chinese Medicine, focusing on the clinical efficacy and differential analysis of electroacupuncture vs. traditional manual acupuncture in KOA treatment, thus contributing to the evidence base for the inclusion of acupuncture in KOA clinical guidelines. Brian M Berman was the most cited author (106 citations), contributing extensively to the research on acupuncture (especially electroacupuncture) and herbal medicine for musculoskeletal disorders, significantly advancing the basic research on electroacupuncture for KOA. Notable articles include an RCT by Witt C., published in The Lancet, with 80 citations, marking a breakthrough in international acupuncture research and promoting its inclusion in clinical guidelines (31). Another influential article is an RCT by Brian M. Berman in Annals of Internal Medicine, demonstrating a strong citation burst, reflecting the standardization of acupuncture clinical research and its growing clinical application (32).

Keyword analysis emphasized research themes such as “chronic pain” and “analgesia,” indicating a strong focus on pain management in osteoarthritis. The use of “randomized controlled trial” and “systematic review” reflects the field’s reliance on RCTs and meta-analyses to evaluate acupuncture’s efficacy and safety, providing high-level evidence for KOA treatment. Keywords like “management” and “guidelines” point to the international recognition of acupuncture’s effectiveness, with numerous guidelines and consensus recommending its use, further accelerating research in this area. Compared with traditional manual acupuncture, research on electroacupuncture has significantly increased since around 2016 and has become a hotspot in acupuncture research for knee osteoarthritis (KOA) in recent years. Although research on manual acupuncture has persisted, its growth has been relatively steady. This result demonstrates the rising trend of electroacupuncture in recent studies. “Electroacupuncture,” a specialized form of acupuncture, with numerous clinical trials confirming its advantages over traditional manual acupuncture in pain relief and functional improvement. In addition to electroacupuncture, other acupuncture therapies such as laser acupuncture, auricular acupuncture, dry needling, and fire needle are also applied in clinical settings. Although research related to “gut microbiota” is currently limited in quantity, preliminary studies have focused on the impact of acupuncture on the gut microbiota of KOA patients, suggesting that acupuncture may exert its therapeutic effects by regulating the gut microbiota.

### 4.2 The research trends and hotspots

The upward trend in publications and the emergence of new research topics indicate several important directions for future studies in acupuncture and KOA. Firstly, acupuncture has been widely applied in the treatment of KOA, and is the focus of increasing research interest regarding its clinical efficacy, yet the absence of universally accepted and standardized outcome measures has hindered the translation of research findings into high-quality clinical evidence. The development of a core outcome set for acupuncture in KOA treatment would enhance the scientific, rational, and feasible design of clinical studies in TCM, providing more robust evidence for clinical research (42). Therefore, more large-scale, high-quality RCTs are needed to determine the clinical efficacy of acupuncture in KOA management (43). These RCTs should focus on standardizing outcome measures, ensuring the proper use of safety indicators, calculating sample sizes, setting follow-up intervals, and evaluating long-term outcomes, particularly long-term efficacy (44, 45). Numerous acupuncture studies in KOA have been published in journals with lower impact factors; thus, research in this field should adhere to guidelines from top-tier journals to ensure standardized reporting and enhance the generalizability of findings (43). Additionally, acupuncture’s placebo effect is notable, with some patients showing significant improvement even under sham acupuncture conditions (46). Exploring the contribution of the placebo effect and optimizing the interaction between patients and providers may help enhance acupuncture’s clinical efficacy.

Secondly, challenges remain regarding the “dose” of acupuncture in KOA treatment. David J. Hunter, in his 2021 commentary in Arthritis and Rheumatology, highlighted the lack of clarity around the optimal dosage (47). Dose refers to the number of treatments, needle quantity, insertion depth, type and intensity of stimulation, or retention time of the needles. This uncertainty complicates the standardization of treatment protocols and the assessment of acupuncture’s true efficacy (47). Moreover, the frequency of treatment plays a critical role. A recent study suggests that more frequent treatments (e.g., three times per week) may yield better outcomes compared to less frequent ones (23). This study has initiated a paradigm for quantitative acupuncture research. Future research should focus on clarifying the dose-response relationship, exploring the feasibility of frequent treatment regimens, and investigating potential mechanisms that explain the sustained benefits of acupuncture.

Finally, there is growing interest in comparing different acupuncture techniques with other therapeutic modalities in the treatment of KOA (44). Current research includes electroacupuncture, manual acupuncture, as well as electro-thermal acupuncture, fire needling, and auricular acupuncture. Future studies should explore the advantages of various acupuncture techniques to improve the KOA acupuncture management system. The application of advanced technologies, such as nanoneedling and bioelectronic acupuncture, is receiving increasing attention, demonstrating how acupuncture can integrate with modern medical advancements to provide more precise and effective treatments (27). Research is also beginning to explore acupuncture’s role in altering knee joint metabolism, reducing inflammation, and promoting cartilage regeneration, highlighting acupuncture’s multifunctionality in KOA management (5). Moreover, research is shifting toward exploring the molecular mechanisms of acupuncture, particularly its effects on cytokines, bone metabolism, and neuroplasticity (48). These emerging trends offer broader insights, not only viewing acupuncture as a symptomatic relief modality but as a treatment strategy capable of altering the underlying mechanisms of the disease.

### 4.3 Limitations

Despite the positive trends and expanding body of research on acupuncture for KOA, there are several limitations in the current literature. Firstly, while there has been a surge in the number of studies, many of these are published in journals with low impact factors or with small sample sizes, limiting the generalizability of the findings. Secondly, many studies use variable outcome measures, making direct comparisons across studies challenging. The quality of some RCTs remains a concern, with biases in randomization, participant blinding, and control group selection affecting the reliability of results. Last limitation is the lack of standardized treatment protocols, with variations in acupuncture techniques (e.g., needle insertion depth, duration, and frequency) between studies, which hinders the ability to assess the overall efficacy of acupuncture for KOA.

Future studies should aim to improve methodological quality, standardize acupuncture protocols, and implement larger, multicenter trials to enhance the robustness of the evidence. Furthermore, the exploration of acupuncture’s synergistic effects when combined with other treatments, such as pharmacological therapies, could open new research avenues. Finally, more studies are needed to delve into the molecular and physiological mechanisms by which acupuncture mediates its effects, potentially offering a more comprehensive understanding of its role in KOA treatment.

## 5 Conclusion

This study highlights the growing global interest in acupuncture for KOA, evidenced by an increasing number of high-quality publications and emerging research hotspots. Despite the increasing recognition of acupuncture as a promising non-pharmacological intervention, challenges remain, particularly in standardizing outcome measures and clarifying the optimal treatment protocol. Future research should focus on large-scale RCTs that standardize outcome measures and explore acupuncture’s long-term benefits. Additionally, exploring the role of acupuncture’s placebo effect and optimizing patient-provider interactions could enhance treatment efficacy. The integration of advanced acupuncture techniques and the exploration of molecular mechanisms are promising avenues for further study. Moreover, research should also delve into the differences in the efficacy of various acupuncture techniques, such as electroacupuncture, manual acupuncture, and others, to identify the most effective approaches for KOA treatment.

## Data Availability

The original contributions presented in the study are included in the article/Supplementary material, further inquiries can be directed to the corresponding authors.
