# Supplementary material for: Research trends and hotspots of acupuncture for knee osteoarthritis from 2004 to 2024: a bibliometric analysis
Source: Front Med (Lausanne). 2025 Jun 10;12:1604209. doi: 10.3389/fmed.2025.1604209 (PMC12185490; doi:10.3389/fmed.2025.1604209)
Supplement: Supplementary file 1 [file Table_1.docx]

**Supplementary Material 1 List of 295 publications**

FN Clarivate Analytics Web of science

PT J

AU Lee, Y. J.

AU - Han, C. H.

AU - Jeon, J. H.

AU - Kim, E.

AU - Park, K. H.

AU - Kim, A. R.

AU - Il Kim, Y.

PY 2025

TI Combination Treatment with Thread-Embedding Acupuncture and Electroacupuncture for Knee Osteoarthritis Patients with Postoperative Pain: A Randomized Controlled Feasibility Study

PG 89-103

DA 2025/1/1

SO JOURNAL OF PAIN RESEARCH

JO JOURNAL OF PAIN RESEARCH

VL 18

SN 1178-7090

Z9 Times Cited in Web of Science Core Collection: 0 Total Times Cited: 0 Cited Reference Count: 53 ER -

M3 10.2147/JPR.S453133

N1 LeeHan-218

Y2 2025/1/18 17:04:00

N1 2025/2/13 1:16:00

DE thread-embedding acupuncture; electroacupuncture; osteoarthritis; knee; randomized controlled trial; ADJUNCTIVE THERAPY; PHARMACOLOGICAL-TREATMENT; RISK-FACTORS; NECK PAIN; POLYDIOXANONE; SAFETY; TRANSPLANTATION; VALIDATION; WOMAC; HIP

AB Purpose: This study aimed to evaluate the effectiveness and safety of combination treatment with thread-embedding acupuncture (TEA) and electroacupuncture (EA) in patients with persistent knee pain after arthroscopic surgery, autologous chondrocyte implantation, or autologous osteochondral transplantation. Patients and Methods: Twelve patients with knee osteoarthritis (KOA) who experienced postoperative pain were randomized to either the treatment group (TG) or control group (CG) in a 1:1 ratio. The TG received TEA once a week for four sessions and EA twice a week for eight sessions while continuing usual care, defined as standard conventional treatments. The CG received only usual care for four weeks. The primary outcome was the visual analogue scale (VAS) score at week 4 compared with the baseline. The secondary outcomes were the VAS scores at weeks 2, 6, and 8, the Korean version of the Western Ontario and McMaster Universities Osteoarthritis Index (K-WOMAC), the EuroQol 5-Dimension 5-Level (EQ-5D-5L), and rescue medication consumption at weeks 2, 4, 6, and 8. Adverse events were assessed at each visit. Results: The TG showed significant improvement in the VAS scores at weeks 4, 6, and 8 compared with the CG (week 4: -24.5; p = 0.0106, week 6: -19.667; p = 0.0228, week 8: -28.667; p = 0.0036). In the TG, significant differences were observed in K-WOMAC total scores at weeks 2, 4, 6, and 8 (week 2: 17.167; p = 0.0083, week 4: 23; p = 0.0018, week 6: 29.833; p = 0.0009, week 8: 30.5; p = 0.0006); however, there were no differences between the two groups. The two groups had no significant differences in the EQ-5D-5L and rescue medication consumption. No adverse events were observed in either groups during the study period. Conclusion: This feasibility study suggests that adding combination treatment with TEA and EA to usual care might relieve pain in patients with KOA. Large-scale clinical trials are needed to confirm the long-term effects of combination treatment.

C1 Daejeon Univ, Coll Korean Med, Dept Acupuncture & Moxibust Med, Daejeon, South Korea; Korea Inst Oriental Med, KM Sci Res Div, Daejeon, South Korea; Univ Sci & Technol UST, Campus Korea Inst Oriental Med, Korean Convergence Med, Daejeon, South Korea; Pusan Natl Univ, Korean Med Hosp, Dept Acupuncture & Moxibust Med, Yangsan, South Korea; Pusan Natl Univ, Sch Korean Med, Div Clin Med, Yangsan, South Korea; Korea Inst Oriental Med, KM Data Div, Daejeon, South Korea; Korea Inst Oriental Med, Clin Res Coordinating Team, Daejeon, South Korea

DI 10.2147/JPR.S453133

ID 218

ER

FN Clarivate Analytics Web of science

PT J

AU Zhang, C.

AU - Yu, M.

AU - Zhang, L. Y.

AU - Zhou, X.

AU - Han, J. C.

AU - Fu, B. F.

AU - Xue, H. F.

AU - Zhang, C.

PY 2025

TI Exploring the Analgesic Effect of Acupuncture on Knee Osteoarthritis Based on MLT/cAMP/PKA/ CREB Signaling Pathway

PG 237-249

DA 2025/1/1

SO JOURNAL OF INFLAMMATION RESEARCH

JO JOURNAL OF INFLAMMATION RESEARCH

VL 18

SN 1178-7031

Z9 Times Cited in Web of Science Core Collection: 0 Total Times Cited: 0 Cited Reference Count: 60 ER -

M3 10.2147/JIR.S498202

N1 ZhangYu-238

N1 2025/2/13 1:16:00

DE acupuncture; knee osteoarthritis; MLT/cAMP/PKA/CREB pathway; melatonin; MELATONIN; INFLAMMATION; GUIDELINES; KINASES; PROTEIN; MODEL; HIP

AB Background: Acupuncture is an effective treatment for knee osteoarthritis (KOA), reducing pain and improving function. While melatonin (MLT) has notable pain relief benefits, the analgesic mechanism of acupuncture in KOA and its relationship with melatonin are still unknown. This study aims to explore this mechanism. Methods: In this work, the KOA rabbit model was constructed using the traditional Hulth method, and the therapeutic effect was assessed by the Lequesne MG score and Pain assessment by hot plate test. The pathological alterations of cartilage tissue were observed using hematoxylin and eosin (H&E) staining, Safranin O-fast green and MASSON staining to observe the pathological changes in cartilage tissue, and the efficacy was evaluated according to the principles of Mankin score and Osteoarthritis Research Society International (OARSI) score. Meanwhile, MLT in serum, cyclic adenosine monophosphate (cAMP) in cartilage, and matrix metalloproteinase-3 (MMP-3) in joint fluid were detected by enzyme-linked immunosorbent assay. In addition, the expression of aromatic L-amino acid N-acetyltransferase (AANAT), melatonin receptor 1 (MT1) and 2 (MT2) mRNAs in cartilage was determined by real-time quantitative reverse transcription-polymerase chain reaction, and the levels of proteins related to PKA/CREB signaling pathway were detected by Western blotting. Results: Based on the results of Lequesne MG score and Pain assessment by hot plate test experimental data, the treatment group presented significant improvements in knee pain and overall function relative to OA (Osteoarthritis) group. Besides, according to results of histologic staining, Mankin and OARSI scores, articular cartilage degeneration of treatment group remarkably improved. In addition, acupuncture significantly reduced the expression of the inflammatory factor MMP-3 in knee joint fluid and significantly increased the levels of MLT, AANAT, MT1, MT2, cAMP, PKA and CREB. Conclusion: By regulating sympathetic excitability, acupuncture may activate the MLT/cAMP/PKA/CREB signaling pathway, decrease inflammatory factor expression and slow down degradation of articular cartilage, resulting in the relief of knee pain.

C1 Tianjin Univ Tradit Chinese Med, Teaching Hosp 1, Orthoped Dept, Tianjin 300380, Peoples R China; Natl Clin Res Ctr Chinese Med Acupuncture & Moxibu, Tianjin 300380, Peoples R China; Tianjin Univ Tradit Chinese Med, Hosp 2, Dept Nephrol & Rheumatol, Tianjin 300250, Peoples R China

DI 10.2147/JIR.S498202

ID 238

ER

FN Clarivate Analytics Web of science

PT J

AU Luo, H. S.

AU - Jing, C. Y.

AU - Liu, H. B.

PY 2024

TI Curative Effect of Electroacupuncture and Manual Acupuncture for Knee Osteoarthritis: A Meta-Analysis

PG 1951-1963

DA 2024/1/1

SO IRANIAN JOURNAL OF PUBLIC HEALTH

JO IRANIAN JOURNAL OF PUBLIC HEALTH

VL 53

IS 9

SN 2251-6085

Z9 Times Cited in Web of Science Core Collection: 1 Total Times Cited: 1 Cited Reference Count: 41 ER -

N1 LuoJing-6

Y2 2025/1/18 22:14:00

N1 2025/2/13 1:16:00

DE Knee osteoarthritis; Electroacupuncture; Pain; Function; SHAM; CARE; PAIN

AB Background: We aimed to examine how electroacupuncture and manual acupuncture affect treatment results, pain levels, and joint function in individuals with knee osteoarthritis. Methods: Research was carried out in various databases including PubMed, Medline, Embase, CENTRAL, and CNKI. Following the "Cochrane manual", the risk of bias of included RCTs was assessed. A funnel plot was utilized to evaluate any potential bias in the publications. The impact size was indicated by the average discrepancy along with its 95% confidence interval. Results: The EA group showed a higher effectiveness rate (P = 0.001) and a lower WOMAC pain score (P < 0.00001) compared to the control group. The EA group had a lower WOMAC pain score compared to the SA/exercise group and the group that received manual acupuncture. The WOMAC pain score was significantly lower in the EA group compared to the manual acupuncture group under intense electroacupuncture stimulation (P < 0.0001). The WOMAC pain score was significantly lower in the EA group compared to the manual acupuncture group when weak current acupuncture was applied (P = 0.0001). However, no significant difference in WOMAC function score between EA and control group. Conclusion: Comparison to manual acupuncture, placebo acupuncture, and exercise training, electroacupuncture enhanced the effectiveness of treating KOA and decreased the WOMAC pain score in patients with KOA. The level of pain relief achieved may be linked to the strength of the current stimulation. However, electroacupuncture had no significant effect on WOMAC function score.

C1 Baisha Li Autonomous Cty Peoples Hosp, Dept Tradit Chinese Med, Baisha 572800, Hainan, Peoples R China; Hainan Med Univ, Hainan Acad Med Sci, Coll Tradit Chinese Med, Haikou 571199, Hainan, Peoples R China

ID 6

ER

FN Clarivate Analytics Web of science

PT J

AU Huang, Y. J.

AU - Huang, H.

AU - Chen, Q. Q.

AU - Luo, Y. T.

AU - Feng, J. N.

AU - Deng, Y. X.

AU - Li, G. Y.

AU - Li, M.

AU - Sun, J.

PY 2024

TI Efficacy and immune-inflammatory mechanism of acupuncture-related therapy in animal models of knee osteoarthritis: a preclinical systematic review and network meta-analysis

DA 2024/1/1

SO JOURNAL OF ORTHOPAEDIC SURGERY AND RESEARCH

JO JOURNAL OF ORTHOPAEDIC SURGERY AND RESEARCH

VL 19

IS 1

SN 1749-799X

Z9 Times Cited in Web of Science Core Collection: 0 Total Times Cited: 1 Cited Reference Count: 95 ER -

M3 10.1186/s13018-024-04660-9

N1 HuangHuang-14

N1 2025/2/13 1:16:00

DE Acupuncture; Acupotomy; Knee osteoarthritis; Animal models; Immunity; Inflammation; Systematic review; Network meta-analysis; SYNOVIAL MACROPHAGES; ARTICULAR-CARTILAGE; ELECTROACUPUNCTURE; ACUPOTOMY; CHONDROCYTES; RECEPTORS; MEDIATORS; CELLS; PAIN

AB BackgroundMany KOA patients have not reached indications for surgery, thus we need to find effective non-surgical treatments. Acupuncture is thought to have the potential to modulate inflammation and cytokines in KOA through the immune system. However, the mechanisms have not been elucidated, and there is no network Meta-analysis of acupuncture on KOA animals. So we evaluate the effect and mechanism of acupuncture-related therapy in KOA animals.MethodsA comprehensive search was conducted in multiple databases including PubMed, Web of Science, Embase, CBM, CNKI, WanFang, and VIP Database to identify relevant animal studies focusing on acupuncture therapy for KOA. The included studies were assessed for risk of bias using SYRCLE's Risk of Bias tool. Subsequently, pair-wise meta-analysis and network meta-analysis were performed using Stata 15.0 software, evaluating outcomes such as Lequesne index scale, Mankin score, IL-1 beta, TNF-alpha, MMP3, and MMP13.Results56 RCTs with 2394 animals were included. Meta-analysis showed that among the 6 outcomes, there were significant differences between acupuncture and model group; the overall results of network meta-analysis showed that the normal group or sham operation group performed the best, followed by the acupotomy, acupuncture, and medicine group, and the model group had the worst effect, and there were significant differences between 6 interventions.ConclusionsAcupuncture-related therapy can be a possible treatment for KOA. The mechanism involves many immune-inflammatory pathways, which may be mediated by DAMPs/TLR/NF-kappa B/MAPK,PI3K/Akt/NF-kappa B pathway, or IFN-gamma/JAK-STAT pathway. It needs to be further confirmed by more high-quality animal experiments or meta-analysis.Systematic review registrationPROSPERO identifier: CRD42023377228.

C1 Guangzhou Univ Chinese Med, Clin Med Coll Acupuncture Moxibust & Rehabil, Guangzhou, Peoples R China; Guangzhou Univ Chinese Med, Clin Coll 2, Guangzhou, Peoples R China; Guangzhou Univ Chinese Med, Clin Coll 1, Guangzhou, Peoples R China; Southern Theater Gen Hosp, Guangzhou, Peoples R China; Guangzhou Med Univ, Affiliated Hosp 3, Dept Tradit Chinese Med, Guangzhou, Peoples R China; Guangzhou Med Univ, Affiliated Hosp 3, Guangdong Prov Key Lab Major Obstet Dis, Guangzhou, Peoples R China; Guangzhou Med Univ, Affiliated Hosp 3, Guangdong Prov Clin Res Ctr Obstet & Gynecol, Guangzhou, Peoples R China

DI 10.1186/s13018-024-04660-9

ID 14

ER

FN Clarivate Analytics Web of science

PT J

AU Yu, S. C.

AU - Te, K. K.

AU - Yap, Y. P.

PY 2024

TI Recent Advances in Research on Acupuncture Treatment for Knee Osteoarthritis

PG 39-46

DA 2024/1/1

SO JOURNAL OF THE ANATOMICAL SOCIETY OF INDIA

JO JOURNAL OF THE ANATOMICAL SOCIETY OF INDIA

VL 73

IS 1

SN 0003-2778

Z9 Times Cited in Web of Science Core Collection: 0 Total Times Cited: 0 Cited Reference Count: 57 ER -

M3 10.4103/jasi.jasi_110_23

N1 YuTe-24

Y2 2025/1/17 15:57:00

N1 2025/2/13 1:16:00

DE Acupuncture treatment; knee osteoarthritis; literature; research progress; review; INFLAMMATION

AB Background and Objective: Knee osteoarthritis (KOA) is one of the leading causes of disability worldwide. In recent years, the incidence of KOA has been gradually increasing due to population aging and lifestyle changes. As a result, there has been a growing body of research and clinical applications related to acupuncture treatment for KOA. The aim of this study is to analyze and summarize the clinical research progress of acupuncture treatment for KOA in the past 5 years. It seeks to understand the current developments and trends in this field and lay the foundation for future research. Methods: Conducted a systematic review and analysis of clinical research literature on acupuncture treatment for KOA published in the last 5 years, both domestically and internationally. Results: Acupuncture treatment for KOA has shown diverse and effective methods. However, the quality of research literature varies, leading to differences in reported treatment outcomes and a lack of high-quality research findings. Conclusions: It was recommended that future research should develop comprehensive and well-designed study protocols, conduct research with scientific rigor, and improve the overall quality of scientific investigations. In addition, it is advisable to include a few horizontal comparative studies to provide more evidence for the clinical application of acupuncture in treating KOA.

C1 Univ Tunku Abdul Rahman, M Kandiah Fac Med & Hlth Sci, Dept Chinese Med, Kajang, Selangor, Malaysia

DI 10.4103/jasi.jasi_110_23

ID 24

ER

FN Clarivate Analytics Web of science

PT J

AU Zhu, W. T.

AU - Guo, C. Q.

AU - Du, M.

AU - Ma, Y. X.

AU - Cui, Y. Q.

AU - Chen, X. L.

PY 2024

TI Acupotomy alleviates knee osteoarthritis in rabbit by regulating chondrocyte mitophagy <i>via</i> Pink1-Parkin pathway

PG 468-477

DA 2024/1/1

SO JOURNAL OF TRADITIONAL CHINESE MEDICINE

JO JOURNAL OF TRADITIONAL CHINESE MEDICINE

VL 44

IS 3

SN 0255-2922

Z9 Times Cited in Web of Science Core Collection: 0 Total Times Cited: 0 Cited Reference Count: 44 ER -

M3 10.19852/j.cnki.jtcm.20240402.001

N1 ZhuGuo-29

N1 2025/2/13 1:16:00

DE acupuncture therapy; osteoarthritis; knee; ARTICULAR-CARTILAGE; METABOLISM; AUTOPHAGY

AB OBJECTIVE: To investigate the effect of acupotomy, on mitophagy and the Pink1-Parkin pathway in chondrocytes from rabbits with knee osteoarthritis (KOA). METHODS: A KOA model was established via the modified Videman method. Rabbits were randomly divided into a control group (CON), KOA group and KOA + acupotomy group (Acu). Rabbits in the acupotomy group were subjected to acupotomy for 4 weeks after model establishment. The behavior of the rabbits before and after intervention was recorded. Cartilage degeneration was evaluated by optical microscopy and fluorescence microscopy. The level of mitophagy was evaluated by transmission electron microscopy, immunofluorescence and enzyme-linked immunosorbent assay (ELISA). The expression of phosphatase and tensin homolog (PTEN)-induced kinase 1 (Pink1)-Parkin mitophagy pathway components was evaluated by immunofluorescence, Western blotting and real -time polymerase chain reaction. RESULTS: In rabbits with KOA, joint pain, mobility disorders and cartilage degeneration were observed, the Mankin score was increased, collagen type II (Col- II) expression was significantly decreased, mitophagy was inhibited, mitochondrial function was impaired, and factors associated with the Pink1-Parkin pathway were inhibited. Acupotomy regulated the expression of Pink1Parkin pathway-related proteins, the mitophagy-related protein microtubule-associated protein -1 light chain-3, the translocase of the outer membrane, and the inner mitochondrial membrane 23; increased the colocalization of mitochondria and autophagosomes; promoted the removal of damaged mitochondria; restored mitochondrial adenosine-triphosphate (ATP) production; and alleviated cartilage degeneration in rabbits with KOA. CONCLUSIONS: Acupotomy played a role in alleviating KOA in rabbits by activating mitophagy in chondrocytes via the regulation of proteins that are related to the Pink1Parkin pathway. (c) 2024 JTCM. All rights reserved.

C1 Beijing Univ Chinese Med, Affiliated Hosp 3, Beijing 100029, Peoples R China; Beijing Univ Chinese Med, Sch Acupuncture Moxibust & Tuina, Beijing 100029, Peoples R China; Shijiazhuang Med Coll, Dept Med Technol, Shijiazhuang 050599, Hebei, Peoples R China; Shijiazhuang Med Coll, Shijiazhuang 050599, Hebei, Peoples R China

DI 10.19852/j.cnki.jtcm.20240402.001

ID 29

ER

FN Clarivate Analytics Web of science

PT J

AU Yan, J.

AU - Jiang, S. Y.

AU - Zhou, X.

AU - Zhao, M.

AU - Huang, J. L.

AU - Zhu, H. M.

AU - Huang, B. Y.

AU - Li, E. M.

AU - Chang, H.

PY 2024

TI Use of the improved tug-of-war acupuncture for promoting cartilage repair by inducing macrophage polarization in knee osteoarthritis

DA 2024/1/1

SO HELIYON

JO HELIYON

VL 10

IS 4

SN 2405-8440

Z9 Times Cited in Web of Science Core Collection: 0 Total Times Cited: 0 Cited Reference Count: 31 ER -

M3 10.1016/j.heliyon.2024.e25495

N1 YanJiang-39

Y2 2025/1/17 16:50:00

N1 2025/2/13 1:16:00

DE Knee osteoarthritis; Tug of war acupuncture; Macrophage polarization; MANUAL ACUPUNCTURE; ELECTROACUPUNCTURE; PAIN

AB Introduction: Knee osteoarthritis (KOA) is a type of joint disease causing degenerative changes that are challenging to treat. The improved tug-of-war acupuncture (BHZF) can improve joint pain in KOA. However, the associated mechanism has not been validated. Methods: The KOA rabbit model was established. After the surgery, the improved BHZF was provided as an intervention, and the animals were euthanized after 2 weeks. Histopathological changes in the synovium and cartilage were observed on hematoxylin & eosin staining and Safranin O-Fast Green staining. Synovial fluid and serum samples were collected to assess the presence of cytokines using the enzyme-linked immunosorbent assay. The expression of M1 macrophage (CD86) and M2 macrophage (ARG1) markers in the cartilage and synovium was detected via immunohistochemistry and immunofluorescence assays. Results: The improved BHZF could reduce KOA-related pain and inhibit joint swelling. Further, it significantly maintained the morphology of articular chondrocytes in KOA and reduced the decomposition of the cartilage matrix. Then, it significantly reduced the expression of CD86positive cells (P < 0.05), and increased the expression of ARG1-positive cells in the cartilage and synovium (P < 0.05). Moreover, it significantly decreased the expression of inflammatory factors interleukin (IL)-1 beta and tumor necrosis factor-alpha in the serum and synovial fluid (P < 0.05), and significantly increased the expression levels of anti-inflammatory cytokines IL-4 and IL-10 (P < 0.05). Conclusions: The improved BHZF can relieve pain and improve cartilage damage by regulating macrophage polarization in KOA.

C1 Shenzhen Hosp Integrated Tradit Chinese & Western, Dept Rehabil, Shenzhen 518104, Guangdong, Peoples R China; Shenzhen Hosp Integrated Tradit Chinese & Western, Hosp Infect Control Sect, Shenzhen 518104, Guangdong, Peoples R China; Guangdong Pharmaceut Univ, Affiliated Hosp 1, Dept Orthoped, Guangzhou 510000, Guangdong, Peoples R China; Guangdong Pharmaceut Univ, Affiliated Hosp 1, Dept Orthoped, 19 Nonglin Xia Rd, Guangzhou 510000, Guangdong, Peoples R China

DI 10.1016/j.heliyon.2024.e25495

ID 39

ER

FN Clarivate Analytics Web of science

PT J

AU Liu, C. Y.

AU - Duan, Y. S.

AU - Zhou, H.

AU - Wang, Y.

AU - Tu, J. F.

AU - Bao, X. Y.

AU - Yang, J. W.

AU - Lee, M. S.

AU - Wang, L. Q.

PY 2024

TI Clinical effect and contributing factors of acupuncture for knee osteoarthritis: a systematic review and pairwise and exploratory network meta-analysis

DA 2024/1/1

SO BMJ EVIDENCE-BASED MEDICINE

JO BMJ EVIDENCE-BASED MEDICINE

SN 2515-446X

Z9 Times Cited in Web of Science Core Collection: 0 Total Times Cited: 0 Cited Reference Count: 52 ER -

M3 10.1136/bmjebm-2023-112626

N1 LiuDuan-42

Y2 2025/1/20 13:39:00

N1 2025/2/13 1:16:00

DE Acupuncture; Knee; PAIN MANAGEMENT; MANAGEMENT; THERAPY; PAIN; HIP

AB Objectives This study aims to evaluate (1) the effect and safety of acupuncture in patients with knee osteoarthritis (KOA) and explore (2) whether the effect of acupuncture differed according to acupuncture type, acupuncture dose and follow-up time. Design Systematic review and pairwise and exploratory network meta-analysis. Setting PubMed, Embase, Cochrane Central Register of Controlled Trials, Web of Science, China National Knowledge Infrastructure, Chinese Biomedical Literature Database, VIP Database for Chinese Technical Periodicals and Wanfang from inception to 13 November 2023. Participants Randomised controlled trials comparing acupuncture with sham acupuncture, non-steroidal anti-inflammatory drugs (NSAIDs), usual care or waiting list groups, intra-articular (IA) injection and blank groups in patients with KOA. Interventions Eligible interventions included manual acupuncture (MA) and electroacupuncture (EA). Main outcomes measures The primary outcome was pain intensity at the end of treatment. Results 80 trials (9933 participants) were included. Very low certainty evidence suggested that acupuncture may reduce pain intensity compared with sham acupuncture (standardised mean difference, SMD -0.74, 95% CI -1.08 to -0.39, corresponded to a difference in Visual Analogue Scale of -18.50 mm, -27.00 to -9.75), NSAIDs (SMD -0.86 to -1.26 to -0.46, corresponded to -21.50 mm, -31.50 to -11.50), usual care or waiting list groups (SMD -1.01, -1.47 to -0.54, corresponded to -25.25 mm, -36.75 to -13.50) and blank groups (SMD -1.65, -1.99 to -1.32, corresponded to -41.25 mm, -49.75 to -33.00), but not IA injection. Similar results were also found in other outcomes. For most of the subgroup analyses, acupuncture type, acupuncture dose and follow-up time did not show a significant relative effect. Only when compared with NSAIDs, a higher dose of acupuncture may provide greater pain relief (interaction p<0.001). The network meta-analysis revealed that electroacupuncture (SMD -0.75, 95% CI -1.34 to -0.17) had a greater effect on pain relief in patients with KOA compared with manual acupuncture. Conclusions The findings suggest that acupuncture may provide clinically important effects in reducing pain and improving physical function in patients with KOA, but the certainty of evidence was very low. Electroacupuncture and higher dose of acupuncture probably are two potential contributing factors. PROSPERO registration number CRD42021232177.

C1 Capital Med Univ, Affiliated Beijing Hosp Tradit Chinese Med, Beijing, Peoples R China; Beijing Univ Chinese Med, Sch Tradit Chinese Med, Beijing, Peoples R China; Beijing Univ Chinese Med, Int Acupuncture & Moxibust Innovat Inst, Sch Acupuncture Moxibust & Tuina, Beijing, Peoples R China; Korea Inst Oriental Med, KM Sci Res Div, Daejeon, South Korea

DI 10.1136/bmjebm-2023-112626

ID 42

ER

FN Clarivate Analytics Web of science

PT J

AU Yuan, S. G.

AU - Chen, J.

AU - Chen, M. X.

AU - Zheng, N. S.

AU - Zhang, Z. W.

AU - Wang, H. J.

AU - Li, J.

AU - Li, L.

AU - Gao, Y. P.

PY 2024

TI High-intensity electroacupuncture is superior to low-intensity electroacupuncture for knee osteoarthritis: a meta-analysis of randomized controlled trials

PG 303-310

DA 2024/1/1

SO ACUPUNCTURE IN MEDICINE

JO ACUPUNCTURE IN MEDICINE

VL 42

IS 6

SN 0964-5284

Z9 Times Cited in Web of Science Core Collection: 0 Total Times Cited: 0 Cited Reference Count: 23 ER -

M3 10.1177/09645284241298718

N1 YuanChen-45

Y2 2025/1/17 13:42:00

N1 2025/2/13 1:16:00

DE electroacupuncture; high intensity; low intensity; knee osteoarthritis; meta-analysis; MANUAL ACUPUNCTURE; PAIN

AB Background: Electroacupuncture (EA) has been demonstrated to be efficacious and safe in patients with knee osteoarthritis (KOA), yet the optimal current intensity for pain control in KOA remains unspecified. The present meta-analysis aimed to compare the effects of high-intensity and low-intensity EA in terms of pain relief and functional improvement in KOA. Methods: A thorough and comprehensive literature search for randomized controlled trials (RCTs), all looking at the intensity of EA for KOA, was carried out in PubMed, EMBASE, Cochrane Library, China National Knowledge Infrastructure (CNKI), China Science Journal Citation Report (VIP) and Wanfang database, as well as ClinicalTrials.gov. All databases were searched from their inception until April 2022. Study quality was assessed using the Cochrane risk of bias (RoB)2 tool. Finally, a meta-analysis of all eligible RCTs was performed using Review Manager 5.3. Results: Three studies with 472 individuals were included in the meta-analysis. The pain intensity reductions were significantly different between the high-intensity EA group and low-intensity EA group (mean difference (MD)=-0.22, 95% confidence interval (CI)=-0.26 to -0.18, p<0.00001). There was no significant difference between the two groups in the Western Ontario and McMaster Universities Osteoarthritis Index (WOMAC) scores (MD=-3.62, 95% CI=-12.22 to 4.98, p=0.41). High-intensity EA significantly improved emotional scale (ES) scores compared to low-intensity EA (MD=-0.72, 95% CI=-0.76 to -0.67, p<0.00001). Conclusion: The findings of this systematic review and meta-analysis indicated that high-intensity EA provides superior pain relief and has a bigger impact on emotional scale scores in patients with KOA.

C1 Hainan Med Univ, Hainan Tradit Chinese Med Hosp, Dept Orthopaed, Haikou, Hainan, Peoples R China; Guangzhou Univ Chinese Med, Hainan Hosp, Guangdong Prov Hosp Chinese Med, Dept Orthopaed, Haikou, Hainan, Peoples R China; Jinan Univ, Guangzhou Key Lab Precis Orthoped & Regenerat Med, Affiliated Hosp 1, Dept Sports Med,Guangdong Prov Key Lab Speed Capa, Guangzhou 510630, Peoples R China; Guangdong Acad Med Sci, Guangdong Prov Peoples Hosp, Dept TCM Bone Setting, Guangzhou 510080, Peoples R China; Guangdong Acad Med Sci, Guangdong Prov Peoples Hosp, Div Rheumatol, Guangzhou, Peoples R China; Southern Med Univ, Dept TCM Orthoped & Traumatol, Affiliated Hosp 3, Guangzhou, Peoples R China

DI 10.1177/09645284241298718

ID 45

ER

FN Clarivate Analytics Web of science

PT J

AU Jia, W. R.

AU - Zhang, Y. A.

AU - Wang, T. Q.

AU - Liu, C. Z.

AU - Tu, J. F.

AU - Shi, G. X.

AU - Cai, L. Y.

AU - Yang, J. W.

AU - Huang, G. R.

PY 2024

TI Electroacupuncture Ameliorates Knee Osteoarthritis By Rebalancing T Cell Homeostasis as Revealed By Immune Repertoire (IR) Sequencing

DA 2024/1/1

SO COMBINATORIAL CHEMISTRY & HIGH THROUGHPUT SCREENING

JO COMBINATORIAL CHEMISTRY & HIGH THROUGHPUT SCREENING

SN 1386-2073

Z9 Times Cited in Web of Science Core Collection: 0 Total Times Cited: 0 Cited Reference Count: 75 ER -

M3 10.2174/0113862073303471240805061026

N1 JiaZhang-51

Y2 2025/1/20 13:39:00

N1 2025/2/13 1:16:00

DE Electro-acupuncture; knee osteoarthritis; immune repertoire; t cell receptor; neutrophil; transcriptome; CLASSIFICATION CRITERIA; SYNOVIAL-FLUID; HIP; SERUM; RECOMMENDATIONS; INTERLEUKIN-6; INFLAMMATION; PATHOGENESIS; ACUPUNCTURE; ASSOCIATION

AB Background In this study, we used immune repertoire (IR) sequencing technology to profile the diversity of peripheral blood T cell receptors and used transcriptomics to profile the gene expression of peripheral blood neutrophil mRNA in patients with mild-moderate knee osteoarthritis (KOA) before and after electroacupuncture (EA) treatment. Methods An 8-week intervention with EA was performed on 3 subjects with KOA. IR sequencing of complementarity determining region 3 (CDR3) was performed using RNA extracted from peripheral blood T cells of KOA subjects prior to and at the end of the intervention, as well as healthy volunteers (controls) who matched the subjects in sex and age. Neutrophils were extracted from the plasma of healthy individuals, pretreatment patients, and posttreatment patients for further transcriptome sequencing. Results The D50, diversity index (DI), and Shannon entropy values of circulatory T-cells were significantly lower in pretreatment KOA patients compared to healthy controls. Posttreatment KOA samples displayed significant decreases in serum proinflammatory factors, IL-8 and IL-18 (P < 0.01), as well as a substantial reduction in serum matrix MMP-3 and MMP-13 (P < 0.01, P < 0.05). Transcriptome analysis revealed that the expression of CXCL2, IRF8, and PEAR1 (P < 0.05) was significantly higher in patients before the treatment than in the healthy population and was significantly down-regulated after the treatment. In contrast, the expression of SMPD3 (P < 0.05) showed the opposite trend. Conclusion EA may alleviate KOA by rebalancing T-cell homeostasis and improving systemic inflammation. At the same time, EA treatment can significantly enhance TCR diversity, reduce levels of proinflammatory factors, and increase levels of anti-inflammatory factors, thereby achieving therapeutic effects.

C1 Beijing Univ Chinese Med, Sch Acupuncture Moxibust & Tuina, Beijing 102488, Peoples R China; Beijing Univ Chinese Med, Sch Life Sci, Beijing 102488, Peoples R China

DI 10.2174/0113862073303471240805061026

ID 51

ER

FN Clarivate Analytics Web of science

PT J

AU Qu, Y. Z.

AU - Peng, Y.

AU - Xiong, Y.

AU - Dong, X. H.

AU - Ma, P. H.

AU - Cheng, S. R.

PY 2024

TI Acupuncture-Related Therapy for Knee Osteoarthritis: A Narrative Review of Neuroimaging Studies

PG 773-784

DA 2024/1/1

SO JOURNAL OF PAIN RESEARCH

JO JOURNAL OF PAIN RESEARCH

VL 17

SN 1178-7090

Z9 Times Cited in Web of Science Core Collection: 3 Total Times Cited: 3 Cited Reference Count: 71 ER -

M3 10.2147/JPR.S450515

N1 QuPeng-54

Y2 2025/1/17 15:57:00

N1 2025/2/13 1:16:00

DE acupuncture -related therapy; knee osteoarthritis; cerebral response; neuroimaging study; ANTERIOR CINGULATE CORTEX; FUNCTIONAL CONNECTIVITY; CHRONIC PAIN; SYNAPTIC PLASTICITY; CORTICAL THICKNESS; BRAIN; ELECTROACUPUNCTURE; HIP; METAANALYSIS; ACTIVATION

AB Acupuncture has been widely applied for treating knee osteoarthritis (KOA). Numerous studies have found that acupuncture can effectively alleviate KOA symptoms. With the advancement of neuroimaging techniques, integrating neuroimaging with indepth investigations of acupuncture mechanisms has emerged as a hot topic in traditional Chinese medical neuroscience research. This review aimed to analyze the study design and main findings from neuroimaging studies of acupuncture-related therapy for KOA to provide a reference for future research. Original studies were sourced from English databases (PubMed, Embase, and Cochrane Library) and Chinese databases (Chinese National Knowledge Infrastructure, Chinese Biomedical Literature Database, the Chongqing VIP database, and Wanfang database). As a result, thirteen articles were ultimately included in this review. Functional magnetic resonance imaging was the most frequently used neuroimaging technique to explore cerebral responses to acupuncture-related therapy for KOA. Findings suggested that acupuncture-related therapy could regulate some brain regions in patients with KOA. Specifically, for acupuncture, it showed that the medial pain pathway and the limbic system were involved in the regulation of KOA. Meanwhile, moxibustion induced a wide range of functional activity throughout the entire brain.

C1 Chengdu Sport Univ, Affiliated Sport Hosp, Postdoctoral Sci Res Workstat, Chengdu, Sichuan, Peoples R China; Chengdu Univ Tradit Chinese Med, Acupuncture & Tuina Sch, Chengdu, Sichuan, Peoples R China; Chengdu Univ Tradit Chinese Med, Acupuncture & Brain Sci Res Ctr, Chengdu, Sichuan, Peoples R China; Chengdu Univ Tradit Chinese Med, Med Aesthet Dept, Affiliated Hosp, Chengdu, Sichuan, Peoples R China; Sichuan Univ, Dept Osteoporosis, West China Hosp 4, Chengdu, Sichuan, Peoples R China; Tianjin Univ Tradit Chinese Med, Med Technol Sch, Tianjin, Peoples R China; Chengdu Univ Tradit Chinese Med, 1166 Liutai Ave, Chengdu 611137, Sichuan, Peoples R China; Tianjin Univ Tradit Chinese Med, 10 Poyanghu Rd, Tianjin 301617, Peoples R China

DI 10.2147/JPR.S450515

ID 54

ER

FN Clarivate Analytics Web of science

PT J

AU Zheng, X. H.

AU - Lin, J.

AU - Wang, Z. Z.

AU - Zeng, Z. M.

AU - Chen, H. X.

PY 2024

TI Research of the analgesic effects and central nervous system impact of electroacupuncture therapy in rats with knee osteoarthritis

DA 2024/1/1

SO HELIYON

JO HELIYON

VL 10

IS 1

SN 2405-8440

Z9 Times Cited in Web of Science Core Collection: 0 Total Times Cited: 0 Cited Reference Count: 48 ER -

M3 10.1016/j.heliyon.2023.e21825

N1 ZhengLin-60

N1 2025/2/13 1:16:00

DE Knee osteoarthritis; TongDu TiaoShen; Center system; Analgesic; MANUAL ACUPUNCTURE; PAIN; HIP

AB It aimed to observe the effects of TongDu TiaoShen (TDTS) electroacupuncture (EA) on the analgesia and central system of knee osteoarthritis (KOA) rats and explore its mechanism. SD rats were rolled into the blank group, model group (KOA), control group (duloxetine 500 mg/kg/d, Ctrl), conventional EA group, and TDTS-EA group. Radiometric pain measurements and the Lequesne MG scale were used to evaluate the behavioral performance of the rats. Dopamine (DA), norepinephrine (NE), 5-hydroxytryptamine (5-HT), beta-endorphin (beta-EP), and leucine-enkephalin (L-ENK) were detected in the midbrain and spinal cord of lumbar enlargement. Interleukin (IL)-1 beta protein expression was detected by Western blot. The incubation period of thermal pain and foot contraction was decreased in the KOA group versus blank group, the Lequesne MG score was increased, DA, NE, 5-HT, beta-EP, and L-ENK in the midbrain and spinal cord were increased, and synovial tissue IL-1 beta protein expression was increased (P < 0.05). EA group and TDTS-EA group had an increased incubation period of thermal pain contraction, decreased Lequesne MG score, decreased DA, NE, etc. In the midbrain, increased 5-HT and NE in the spinal cord, and decreased IL-1 beta in the synovial tissue versus KOA group (P < 0.05). The Lequesne MG score and midbrain DA, NE, 5-HT, beta-EP, and synovial tissue IL-1 beta expression were decreased in TDTS-EA group versus EA group (P < 0.05). EA can effectively improve the behavioral score of KOA and participate in central analgesia by regulating central DA, NE, 5-HT, beta-EP, and L-ENK.

C1 Guangzhou Univ Chinese Med, Affiliated Hosp 3, Dept Rehabil, 261 Longxi Ave, Guangzhou 510375, Guangdong, Peoples R China

DI 10.1016/j.heliyon.2023.e21825

ID 60

ER

FN Clarivate Analytics Web of science

PT J

AU Lu, M.

AU - Meng, D. H.

AU - She, Z. Y.

AU - Wu, X.

AU - Xia, S.

AU - Yang, K. N.

AU - Liu, C. B.

AU - Li, T.

AU - Yang, Y. H.

PY 2024

TI Promotion and Mechanism of Acupotomy on Chondrocyte Autophagy in Knee Osteoarthritis Rabbits

PG 809-817

DA 2024/1/1

SO CHINESE JOURNAL OF INTEGRATIVE MEDICINE

JO CHINESE JOURNAL OF INTEGRATIVE MEDICINE

VL 30

IS 9

SN 1672-0415

Z9 Times Cited in Web of Science Core Collection: 0 Total Times Cited: 0 Cited Reference Count: 27 ER -

M3 10.1007/s11655-024-3759-8

N1 LuMeng-89

N1 2025/2/13 1:16:00

DE acupotomy; knee asteoarthritis; autophagy; AMPK/mTOR/ULK1 signaling pathway; rabbit

AB Objective: To explore the effect of acupotomy intervention on autophagy of chondrocytes in rabbits with knee osteoarthritis (KOA), and to determine the possible mechanisms of acupotomy to alleviate cartilage degeneration. Methods: The modified Videman method was used to construct a KOA rabbit model. After modeling, 40 rabbits were randomly divided into 4 groups by a random number table: control; KOA (model); KOA + acupotomy (acupotomy), and KOA + sham acupotomy (sham), 10 in each group. After a 3-week treatment course, the knee joint activity was determined by the modified Lequesne MG index. Hematoxylin-eosin staining staining was used to examine the morphological changes of chondrocytes. Autophagy of chondrocytes was observed by transmission electron microscopy. The surface morphology of cartilage tissue was observed by scanning electron microscope. The mRNA and protein levels of AMP kinase/mammalian target of rapamycin/Unc-51 (AMPK/mTOR/ULK1) signal pathway key proteins, autophagy-related factor Beclin-1 and microtubule-associated protein 1A/1B light chain 3 (LC3) in rabbit knee cartilage were assessed by real-time fluorescence quantitative polymerase chain reaction and Western blot, respectively. Results: The modified Lequesne MG score of acupotomy group was significantly lower than that of model group (P<0.05). Pathological results showed that chondrocyte autophagy decreased and cartilage surface was rough in the model group, which recovered after acupotomy treatment. The mRNA expressions of AMPK, ULK1, Beclin-1 and the protein levels of p-AMPK, p-ULK1, Beclin-1, and LC3 II/LC3 I were decreased in the model group, while the mRNA and protein expressions of mTOR were increased (P<0.01). However, acupotomy treatment reversed these abnormal changes (P<0.05). Conclusions: Acupotomy could effectively up-regulate the expressions of AMPK, ULK1 and Beclin1, reduce the expression of mTOR, promote autophagy, and alleviate joint degeneration. Acupotomy is a promising complementary and alternative therapy for KOA.

C1 Anhui Univ Chinese Med, Affiliated Hosp 1, Hefei 230031, Peoples R China; Anhui Univ Chinese Med, Affiliated Hosp 2, Hefei 230038, Peoples R China; Anhui Prov Hosp Integrated Tradit Chinese & Wester, Dept Acupot Rehabil, Hefei 230031, Peoples R China; Anhui Univ Chinese Med, Affiliated Hosp 2, Hefei 230061, Peoples R China

DI 10.1007/s11655-024-3759-8

ID 89

ER

FN Clarivate Analytics Web of science

PT J

AU Chen, C.

AU - Liu, D.

AU - Guo, S. X.

AU - Chen, B.

AU - Wang, S. Y.

AU - Chen, P. H.

AU - Li, P.

AU - Li, F. F.

AU - Chen, Q.

AU - Li, X. F.

AU - Li, F. Y.

AU - Hu, Z. J.

PY 2024

TI Clinical Effects of Ultrasound-guided Acupotomy in Knee Osteoarthritis Treatment

DA 2024/1/1

SO JOVE-JOURNAL OF VISUALIZED EXPERIMENTS

JO JOVE-JOURNAL OF VISUALIZED EXPERIMENTS

IS 206

SN 1940-087X

Z9 Times Cited in Web of Science Core Collection: 0 Total Times Cited: 0 Cited Reference Count: 33 ER -

M3 10.3791/66587

N1 ChenLiu-99

N1 2025/2/13 1:16:00

AB The protocol presented here demonstrates the operation method of ultrasound-guided acupotomy for knee osteoarthritis (KOA), including patient recruitment, preoperative preparation, manual operation, and postoperative care. The purpose of this protocol is to relieve pain and improve knee function in patients with KOA. A total of 60 patients with KOA admitted between June 2022 and June 2023 were treated with ultrasound-guided acupotomy. Pathological changes and knee function scores were compared before and after the treatment. After 1 week of treatment, the synovial thickness of the suprapatellar bursae was significantly lesser than before treatment (p < 0.05), the Hospital for Special Surgery Knee Score (HSS) was significantly higher than before treatment (p < 0.05), the Visual analogue scale (VAS) was significantly lower than those of the control group (p < 0.05) and Western Ontario and McMaster Universities Osteoarthritis Index (WOMAC) were significantly lower than those of the control group (p < 0.05). Therefore, ultrasound-guided acupotomy for the treatment of KOA can reduce synovial thickness, relieve pain, improve knee joint function, and have a remarkable curative effect.

C1 Shanghai Univ Tradit Chinese Med, Longhua Hosp, Shanghai, Peoples R China; Guangming Hosp Tradit Chinese Med, Shanghai Pudong New Area, Shanghai, Peoples R China

DI 10.3791/66587

ID 99

ER

FN Clarivate Analytics Web of science

PT J

AU Wang, X. Z.

AU - Wang, R. K.

AU - Liu, Q.

AU - Shi, G. X.

AU - Mi, B. H.

AU - Liu, C. Z.

AU - Tu, J. F.

AU - Lin, J. H.

PY 2024

TI Electroacupuncture versus exercise in patients with knee osteoarthritis: Study protocol for a randomized controlled trial

DA 2024/1/1

SO PLOS ONE

JO PLOS ONE

VL 19

IS 6

SN 1932-6203

Z9 Times Cited in Web of Science Core Collection: 1 Total Times Cited: 1 Cited Reference Count: 38 ER -

M3 10.1371/journal.pone.0305105

N1 WangWang-103

Y2 2025/1/20 13:41:00

N1 2025/2/13 1:16:00

DE COST-EFFECTIVENESS; ACUPUNCTURE; HIP; PHYSIOTHERAPY; MANAGEMENT; THERAPY; HEALTH; PREVALENCE; INSTRUMENT; PROGRAM

AB Purpose Knee osteoarthritis (KOA) is a common disorder among middle and older individuals. Electroacupuncture and exercise are present as two popular physical therapies for the management of KOA, and both were demonstrated to produce considerable results. However, the clinical decision-making process between these therapeutic interventions remains challenging due to the limited evidence of distinctions in their respective effects. This study aims to evaluate the clinical effect and cost effectiveness of electroacupuncture versus exercise in patients with KOA.Study design and methods This is a randomized controlled trial in which 196 symptomatic KOA patients will be randomly assigned 1:1 either to the electroacupuncture group (n = 98) and the exercise group (n = 98). Patients in the electroacupuncture group will receive acupuncture with electric stimulation 3 times a week for 8 weeks, whereas patients in the exercise group will receive neuromuscular training twice a week for 8 weeks. Education concerning KOA management will be provided in both therapies. Co-primary outcomes include changes in numerical rating scale (NRS) and Knee injury and Osteoarthritis Outcome Score (KOOS) Activities of Daily Living (ADL) subscale from baseline at week 8. Secondary outcomes include KOOS Pain subscale, KOOS knee-related Quality of Life (QOL) subscale, Short Form 6 Dimensions (SF-6D), five-level EuroQol five-dimensional questionnaire (EQ-5D-5L), Credibility/ Expectancy Questionnaire, Patient's global assessment (PGA), 30-second Chair Stand Test (30s-CST), 40m (4*10m) Fast Paced Walk Test (40m FPWT), and Daily Physical Activity level (DPA).Discussion The results of this study will provide evidence regarding differences between these 2 physical therapies in multiple aspects and will provide specific guidance for the development of treatments based on the needs of individual patients.Trial registration ChiCTR2300070376.

C1 Beijing Univ Chinese Med, Int Acupuncture & Moxibust Innovat Inst, Sch Acupuncture Moxibust & Tuina, Beijing, Peoples R China; Peking Univ, Peoples Hosp, Arthrit Clin & Res Ctr, Beijing, Peoples R China; Peking Univ, Arthrit Inst, Beijing, Peoples R China; Beijing Univ Chinese Med, Affiliated Hosp 3, Beijing, Peoples R China

DI 10.1371/journal.pone.0305105

ID 103

ER

FN Clarivate Analytics Web of science

PT J

AU Huang, R. R.

AU - Wu, J. J.

AU - Shen, J.

AU - Xing, X. X.

AU - Hua, X. Y.

AU - Zheng, M. X.

AU - Xiao, L. B.

AU - Xu, J. G.

PY 2024

TI Limbic system plasticity after electroacupuncture intervention in knee osteoarthritis rats

DA 2024/1/1

SO NEUROSCIENCE LETTERS

JO NEUROSCIENCE LETTERS

VL 820

SN 0304-3940

Z9 Times Cited in Web of Science Core Collection: 0 Total Times Cited: 0 Cited Reference Count: 38 ER -

M3 10.1016/j.neulet.2023.137580

N1 HuangWu-118

N1 2025/2/13 1:16:00

DE Knee osteoarthritis; Electroacupuncture; Pain; Limbic system; Neural plasticity; BRAIN NETWORK; CHRONIC PAIN; MODEL; FMRI

AB Knee osteoarthritis (KOA) is characterized by debilitating pain. Electroacupuncture (EA), a traditional Chinese medical therapy, has shown promise in KOA pain management. This study investigated the therapeutic potential of EA in KOA and its impact on limbic system neural plasticity. Sixteen rats were randomly assigned into two groups: EA group and sham-EA group. EA or sham-EA interventions were administered at acupoints ST32 (Futu) and ST36 (Zusanli) for three weeks. Post-intervention resting-state fMRI was scanned, assessing parameters including Amplitude of low frequency fluctuations (ALFF), regional homogeneity (ReHo), functional connectivity (FC) and nodal characterizations of network within limbic system. The results showed that EA was strategically directed towards the limbic system, resulting in discernible alterations in neural activity, FC, and network characteristics. Our findings demonstrate that EA had a significant impact on the limbic system neural plasticity in rats with KOA, presenting a novel nonpharmacological approach for KOA treatment.

C1 Shanghai Univ Tradit Chinese Med, Yueyang Hosp Integrated Tradit Chinese & Western, Dept Rehabil Med, Shanghai, Peoples R China; Shanghai Univ Tradit Chinese Med, Sch Rehabil Sci, Shanghai, Peoples R China; Minist Educ, Engn Res Ctr Tradit Chinese Med Intelligent Rehab, Shanghai, Peoples R China; Shanghai Univ Tradit Chinese Med, Yueyang Hosp Integrated Tradit Chinese & Western, Dept Traumatol & Orthoped, Shanghai, Peoples R China; Shanghai Univ Tradit Chinese Med, Guanghua Hosp, Shanghai, Peoples R China; Shanghai Univ Tradit Chinese Med, Shanghai Acad Tradit Chinese Med, Arthrit Inst Integrated Tradit Chinese & Western, Shanghai, Peoples R China; Shanghai Univ Tradit Chinese Med, Yueyang Hosp Integrated Tradit Chinese & Western, Dept Rehabil Med, 110 Ganhe Rd, Shanghai, Peoples R China

DI 10.1016/j.neulet.2023.137580

ID 118

ER

FN Clarivate Analytics Web of science

PT J

AU Zhang, J. C.

AU - Pang, T. T.

AU - Yao, J. J.

AU - Li, A. L.

AU - Dong, L.

AU - Wang, Y. T.

AU - Wang, Y. F.

PY 2024

TI Acupotomy therapy for knee osteoarthritis: An overview of systematic reviews

DA 2024/1/1

SO MEDICINE

JO MEDICINE

VL 103

IS 47

SN 0025-7974

Z9 Times Cited in Web of Science Core Collection: 0 Total Times Cited: 0 Cited Reference Count: 27 ER -

M3 10.1097/MD.0000000000039700

N1 ZhangPang-125

N1 2025/2/13 1:16:00

DE acupotomy; AMSTAR 2; GRADE; knee osteoarthritis; overview; systematic review; BURDEN; RISK

AB Background:This study aimed to evaluate the methodological quality, report quality, and evidence quality of a meta-analysis (MA) and systematic review (SR) of the efficacy of acupotomy in the treatment of knee osteoarthritis (KOA), and provided a reference for clinical decision-making.Methods:We searched 8 databases to collect systematic reviews and meta-analyses on the efficacy of acupotomy in the treatment of KOA from January 30, 2018, to January 31, 2023. The methodological quality of the studies was assessed using the assessment of multiple systematic reviews (AMSTAR) 2 scale, the quality of the literature reports was scored using the Preferred Reporting Items for Systems Reviews and Meta-Analyses 2020 Version (PRISMA 2020),and the quality of the evidence was graded using the grading of recommendations assessment, development, and evaluation (GRADE) scale.Results:Nine systematic reviews including 35 outcome indicators were included. AMSTAR 2 evaluated the methodological quality of the included studies, and 1 was of low quality, 8 were of very low quality, and the entries with poor scores were 2, 3, 4, 8, 10, 12, and 13. By PRISMA 2020, there were some reporting deficiencies, and quality problems were mainly reflected in the abstract, information sources, search strategy, synthesis methods, reporting bias assessment, certainty assessment, reporting biases, certainly of evidence, registration and protocol. The GRADE classification results showed that there were 2 medium-quality evidences, 7 low-quality evidences, and 26 very low-quality evidences. The main factors of degradation were limitations, imprecision, and publication bias.Conclusion:Acupotomy had been a promising complementary treatment for KOA. However, due to the low quality of the SRs/MAs supporting these results, high-quality studies with rigorous study designs and larger samples were needed before widespread recommendations could be made.

C1 Changchun Univ Chinese Med, Dept Acupuncture & Tuina, Changchun, Peoples R China; Changchun Univ Chinese Med, Dept Rehabil Med, Changchun, Peoples R China; Zhejiang Prov Peoples Hosp, Rehabil Med Dept, Hangzhou, Peoples R China

DI 10.1097/MD.0000000000039700

ID 125

ER

FN Clarivate Analytics Web of science

PT J

AU Ma, W. W.

AU - Mei, D.

AU - Juan, L.

AU - Xing, L. F.

AU - Chen, X. L.

AU - Hu, T. Y.

AU - Zhu, W. T.

AU - Guo, C. Q.

PY 2024

TI Electroacupuncture improves articular microcirculation and attenuates cartilage hypoxia in a male rabbit model of knee osteoarthritis

PG 414-423

DA 2024/1/1

SO JOURNAL OF TRADITIONAL AND COMPLEMENTARY MEDICINE

JO JOURNAL OF TRADITIONAL AND COMPLEMENTARY MEDICINE

VL 14

IS 4

SN 2225-4110

Z9 Times Cited in Web of Science Core Collection: 1 Total Times Cited: 1 Cited Reference Count: 54 ER -

M3 10.1016/j.jtcme.2024.01.002

N1 MaMei-159

Y2 2025/1/17 16:03:00

N1 2025/2/13 1:16:00

DE Electroacupuncture; Osteoarthritis; Cartilage; GLUT1; PKM2; LDHA; Microcirculation; Rabbit; SYNOVIAL-FLUID; OXYGEN; ACUPUNCTURE; PRESSURES; JOINT; HAND; HIP

AB Background and aim: Hypoxia of the cartilage has been considered as a potential pathogenic factor in knee osteoarthritis (KOA). Studies have shown that impaired blood perfusion of joint leads to cartilage hypoxia. Electroacupuncture (EA) has proven effects on pain relief and improving microcirculation. This study aimed to explore the effect of EA on articular microcirculation and cartilage anoxic and the underlying mechanisms. Procedures: Videman ' s method was used for 6 weeks to establish the KOA model. EA intervention was performed in four points around the knee for 3 weeks after KOA modeling. The Lequesne MG score was used to assess ethology. We recorded the oxygen tension of synovial fluid and the synovial microcirculation in vivo. HE-staining was used to assess cartilage morphology, and immunohistochemistry (IHC), Western blotting, and RT-PCR were used to assess expression of the major glycolytic enzymes glucosetransporter1 (GLUT1), pyruvate kinase M2 (PKM2), and lactate dehydrogenase A (LDHA). Enzyme-linked immunosorbent assay (Elisa) was used to detect lactate content. Results and conclusion: There was a significant decrease in Lequesne MG score and improvement in Mankin score after EA intervention (P < 0.01), a significant increase in synovial microcirculation (P < 0.05) and synovial fluid oxygen tension (P < 0.01), and there was significant decrease in the expression of GLUT1, PKM2 and LDHA (P < 0.01) and lactate (P < 0.05). This study suggested that EA ameliorate cartilage hypoxia and regulate glycolytic metabolism in chondrocytes in KOA model rabbits by improving articular microcirculation and oxygen tension.

C1 Beijing Univ Chinese Med, Sch Acupuncture Moxibust & Tuina, 11 East North Third Ring Rd, Beijing 100029, Peoples R China; Southern Med Univ, Shenzhen Hosp, Shenzhen, Peoples R China

DI 10.1016/j.jtcme.2024.01.002

ID 159

ER

FN Clarivate Analytics Web of science

PT J

AU Wei, Y.

AU - Liu, L. Y.

AU - Ge, H. Q.

PY 2024

TI Clinical effect of acupuncture on knee osteoarthritis and its effect on p38 MAPK signaling pathway

PG 335-343

DA 2024/1/1

SO TURKISH JOURNAL OF PHYSICAL MEDICINE AND REHABILITATION

JO TURKISH JOURNAL OF PHYSICAL MEDICINE AND REHABILITATION

VL 70

IS 3

SN 2587-1250

Z9 Times Cited in Web of Science Core Collection: 0 Total Times Cited: 0 Cited Reference Count: 35 ER -

M3 10.5606/tftrd.2024.13186

N1 WeiLiu-163

N1 2025/2/13 1:16:00

DE Acupuncture; inflammation; knee osteoarthritis; p38 MAPK signaling pathway.; CARTILAGE; ELECTROACUPUNCTURE; CHONDROCYTES; INFLAMMATION; NECROSIS; HIP

AB Objectives: This study aims to investigate the curative efficacy of acupuncture on knee osteoarthritis (KOA) and its improvement on related scores and blood indexes. Patients and methods: Between January 2019 and January 2020, a total of 108 patients (48 males, 60 females; mean age: 61.0 +/- 6.8 years; range 43 to 79 years) with KOA were randomly divided into control group (n=54) and patient group (n=54). Both groups received standard treatment, including adequate rest and exercise and oral celecoxib capsules. The patient group performed acupuncture operations on the Inner knee eye (EX-LE4), outer knee eye (EX-LE5), Yanglingquan (GB34), and Zusanli (ST36). In the control group, three non-acupuncture points were determined for sham acupuncture. The level of Michel Lequesne index of severity for osteoarthritis (CRP), interleukin-1beta (IL-1I3), IL-6, transforming growth factor-beta (TGF-I3), insulin-like growth factor-1 (IGF-1), fibroblast growth factor-2 (FGF-2) and p38 mitogen-activated protein kinase (p38 MAPK) were compared before and after treatment. Results: The reduction of inflammatory markers in the patient group was greater than that in the control group after treatment. The levels of cytokines such as TGF-I3, IGF-1, and FGF-2 were significantly increased after treatment, and the levels in the patient group were higher than those in the control group during the same period. In addition, p38 MAPK messenger ribonucleic acid (mRNA) was significantly downregulated after treatment, and the level in the patient group was lower than that in the control group during the same period. Conclusion: Acupuncture combined with standard treatment can effectively promote the relief of symptoms and the improvement of knee joint function and effectively inhibit the expression of p38 MAPK signaling pathway.

C1 Nanjing Univ Chinese Med, Affiliated Hosp Integrated Tradit Chinese & Wester, Dept Needle Knife, Shizi St, Nanjing 210000, Peoples R China; Nanjing Univ Chinese Med, Affiliated Hosp 1, Dept Acupuncture & Moxibust, Nanjing, Peoples R China

DI 10.5606/tftrd.2024.13186

ID 163

ER

FN Clarivate Analytics Web of science

PT J

AU Lu, H. Y.

AU - Wu, J. J.

AU - Shen, J.

AU - Xing, X. X.

AU - Hua, X. Y.

AU - Zheng, M. X.

AU - Xiao, L. B.

AU - Xu, J. G.

PY 2024

TI Altered Brain Functional and Effective Connectivity Induced by Electroacupuncture in Rats Following Anterior Cruciate Ligament Transection

PG 2495-2505

DA 2024/1/1

SO JOURNAL OF PAIN RESEARCH

JO JOURNAL OF PAIN RESEARCH

VL 17

SN 1178-7090

Z9 Times Cited in Web of Science Core Collection: 0 Total Times Cited: 0 Cited Reference Count: 62 ER -

M3 10.2147/JPR.S465983

N1 LuWu-168

N1 2025/2/13 1:16:00

DE knee osteoarthritis; anterior cruciate ligament transection; chronic pain; electroacupuncture; functional connectivity; effective connectivity; KNEE OSTEOARTHRITIS; ZONA INCERTA; CHRONIC PAIN; ACUPUNCTURE; MECHANISMS; MODELS; CORTEX

AB Background: The chronic pain arising from knee osteoarthritis (KOA) is a prevalent clinical manifestation. As a traditional Chinese approach, electroacupuncture (EA) has a positive influence in relieving chronic pain from KOA. The study aims to explore functional connectivity (FC) and effective connectivity (EC) alterations induced by EA in anterior cruciate ligament transection (ACLT) rat model of KOA using resting-state functional magnetic resonance imaging (fMRI). Methods: After the establishment of ACLT, rats were randomly divided into the EA group and the sham-EA group. The EA group received EA intervention while the sham-EA group received sham-intervention for 3 weeks. Mechanical pain threshold (MPT) assessment was performed before and after intervention, and fMRI was conducted after intervention. Results: EA intervention effectively relieved pain in post-ACLT rats. Results of rest-state functional connectivity (rs-FC) analysis revealed that compared with the sham-EA group, the EA group had higher FC between the right raphe and the left auditory cortex, the left caudate_ putamen and the left internal capsule (IC), as well as the right zona incerta (ZI) and the left piriform cortex, but lower FC between the right raphe and the left hippocampus ventral, as well as the right septum and the left septum. Furthermore, Granger causality analysis (GCA) found the altered EC between the right septum and the left septum, as well as the left IC and the right septum. Conclusion: The results confirmed the effect of EA on analgesia in post- ACLT rats. The alterations of FC and EC, mainly involving basal ganglia and limbic system neural connections, might be one of the neural mechanisms underlying the effect of EA, providing novel information about connectomics plasticity of EA following ACLT.

C1 Shanghai Univ Tradit Chinese Med, Yueyang Hosp Integrated Tradit Chinese & Western M, Dept Rehabil Med, Shanghai, Peoples R China; Shanghai Univ Tradit Chinese Med, Sch Rehabil Sci, 1200 Cailun Rd, Shanghai, Peoples R China; Shanghai Univ Tradit Chinese Med, Guanghua Hosp Integrat Chinese & Western Med, Dept Orthoped, 540 Xinhua Rd, Shanghai, Peoples R China; Shanghai Univ Tradit Chinese Med, Shanghai Acad Tradit Chinese Med, Arthrit Inst Integrated Tradit Chinese & Western M, Shanghai, Peoples R China; Shanghai Univ Tradit Chinese Med, Yueyang Hosp Integrated Tradit Chinese & Western M, Dept Traumatol & Orthoped, Shanghai, Peoples R China; Minist Educ, Engn Res Ctr Tradit Chinese Med Intelligent Rehabi, Shanghai, Peoples R China

DI 10.2147/JPR.S465983

ID 168

ER

FN Clarivate Analytics Web of science

PT J

AU Lee, B.

AU - Kwon, C. Y.

AU - Lee, H. W.

AU - Nielsen, A.

AU - Wieland, L. S.

AU - Kim, T. H.

AU - Birch, S.

AU - Alraek, T.

AU - Lee, M. S.

PY 2024

TI The effect of sham acupuncture can differ depending on the points needled in knee osteoarthritis: A systematic review and network meta-analysis

DA 2024/1/1

SO HELIYON

JO HELIYON

VL 10

IS 4

SN 2405-8440

Z9 Times Cited in Web of Science Core Collection: 2 Total Times Cited: 2 Cited Reference Count: 32 ER -

M3 10.1016/j.heliyon.2024.e25650

N1 LeeKwon-173

N1 2025/2/13 1:16:00

DE Acupuncture therapy; Knee osteoarthritis; Placebo; Sham acupuncture; Network meta-analysis; CONTROLLED-TRIAL; PAIN; MANAGEMENT; QUALITY; HIP

AB Objective: In sham acupuncture-controlled acupuncture clinical trials, although sham acupuncture techniques are different from those of verum acupuncture, the same acupuncture points are often used for verum and sham acupuncture, raising the question of whether sham acupuncture is an appropriate placebo. We aimed to examine the effects of sham and verum acupuncture according to the points needled (same or different between verum and sham acupuncture) in knee osteoarthritis. Methods: Ten databases were searched to find randomized controlled clinical trials (RCTs) assessing the effects of verum acupuncture with sham acupuncture or waiting lists on knee osteoarthritis. Sham acupuncture was classified as using the same acupuncture points as those in verum acupuncture (SATV) or using sham points (SATS). A frequentist network meta-analysis (NMA) was conducted, and the certainty of evidence was evaluated. Results: A total of 10 RCTs involving 1628 participants were included. Verum acupuncture was significantly superior to SATS but not different from SATV in terms of pain reduction. Additionally, SATV was significantly superior to the waiting list. For physical function, no difference were found between verum acupuncture, SATV, and SATS. The certainty of evidence was low to moderate. Conclusion: For knee osteoarthritis, the pain reduction effect of acupuncture may differ according to the needling points of sham acupuncture, and the control group should be established according to the specific aim of the study design and treatment mechanism.

C1 Korea Inst Oriental Med, KM Sci Res Div, Daejeon, South Korea; Dong eui Univ, Dept Oriental Neuropsychiat, Coll Korean Med, Pusan, South Korea; Korea Inst Oriental Med, KM Convergence Res Div, Daejeon, South Korea; Icahn Sch Med Mt Sinai, Dept Family Med & Community Hlth, New York, NY USA; Univ Maryland, Ctr Integrat Med, Sch Med, Baltimore, MD USA; Kyung Hee Univ, Korean Med Hosp, Korean Med Clin Trial Ctr, Seoul, South Korea; Kristiania Univ Coll, Sch Hlth Sci, Oslo, Norway; Inst Hlth Sci, Fac Hlth Sci, Natl Res Ctr Complementary & Alternat Med NAFKAM, Dept Community Med, Tromso, Norway; Korea Inst Oriental Med, KM Sci Res Div, 1672 Yuseong-daero, Daejeon 34054, South Korea

DI 10.1016/j.heliyon.2024.e25650

ID 173

ER

FN Clarivate Analytics Web of science

PT J

AU Chen, H.

AU - Shi, H. Y.

AU - Gao, S.

AU - Fang, J. F.

AU - Yi, J. M.

AU - Wu, W. H.

AU - Liu, X. K.

AU - Liu, Z. S.

PY 2024

TI Durable Effects of Acupuncture for Knee Osteoarthritis: A Systematic Review and Meta-analysis

PG 709-722

DA 2024/1/1

SO CURRENT PAIN AND HEADACHE REPORTS

JO CURRENT PAIN AND HEADACHE REPORTS

VL 28

IS 7

SN 1531-3433

Z9 Times Cited in Web of Science Core Collection: 0 Total Times Cited: 0 Cited Reference Count: 59 ER -

M3 10.1007/s11916-024-01242-6

N1 ChenShi-183

N1 2025/2/13 1:16:00

DE Acupuncture; Knee osteoarthritis; Durable effects; Pain; Function; NONSTEROIDAL ANTIINFLAMMATORY DRUGS; PERIPHERAL-NERVE STIMULATION; COST-EFFECTIVENESS; CLINICAL-TRIALS; PAIN; THERAPY; HIP; VALIDATION; DIAGNOSIS; PATIENT

AB Purpose of Review Knee osteoarthritis (KOA) is a degenerative joint disease which can result in chronic pain and disability. The current interventions available for KOA often fail to provide long-lasting effects, highlighting the need for new treatment options that can offer durable benefits. Previous studies have suggested the efficacy of acupuncture for knee osteoarthritis (KOA) with its durability remaining uncertain. In this review, we aimed to investigate the durability of the efficacy after completion of treatment.Recent Findings We performed thorough searches of PubMed, EMBASE, Web of Science, and Cochrane Central Register of Controlled Trials from inception to November 4, 2023. The outcomes were assessed at all available time points after completion of treatment. Primary outcomes were changes from baseline in pain and function measured using the Western Ontario and McMaster Universities Osteoarthritis Index (WOMAC) pain and function subscales. Secondary outcomes included response rate, overall pain, the WOMAC stiffness subscale, total WOMAC index, and physical and mental health components of 12/36-item Short-Form Health Survey. A total of 10 randomized controlled trials (RCTs) involving 3221 participants were included. Pooled estimates suggested that acupuncture may offer potential improvements in function and overall pain for 4.5 months post-treatment versus sham acupuncture (SA). Acupuncture may provide durable clinically important pain relief and functional improvement up to 5 months post-treatment versus usual care, and up to 6 months post-treatment versus diclofenac. For acupuncture versus no treatment, one trial with large sample size indicated that improvements in pain and function persisted for 3 months post-treatment, while the other trial reported that significant pain reduction and functional improvement were only observed at the end of the treatment, not at 9 months post-treatment. However, acupuncture as adjunct to exercise-based physical therapy (EPT) showed no superiority to SA as an adjunct to EPT or EPT alone up to 11.25 months after completion of treatment.Summary Acupuncture may provide pain alleviation and functional improvements in KOA patients for 3 to 6 months after completion of treatment with a good safety profile.

C1 China Acad Chinese Med Sci, Guanganmen Hosp, Dept Acupuncture, 5 Beixiange St, Beijing 100053, Peoples R China; Beijing Univ Chinese Med, Grad Coll, Beijing, Peoples R China; Guangzhou Univ Chinese Med, Guangzhou, Peoples R China

DI 10.1007/s11916-024-01242-6

ID 183

ER

FN Clarivate Analytics Web of science

PT J

AU Liu, X. Y.

AU - Ma, Y.

AU - Huang, Z. Y.

AU - Xiao, X. X.

AU - Guan, L.

PY 2024

TI The Efficacy of Acupuncture, Exercise Rehabilitation, and Their Combination in the Treatment of Knee Osteoarthritis: A Randomized Controlled Trial

PG 2837-2849

DA 2024/1/1

SO JOURNAL OF PAIN RESEARCH

JO JOURNAL OF PAIN RESEARCH

VL 17

SN 1178-7090

Z9 Times Cited in Web of Science Core Collection: 0 Total Times Cited: 0 Cited Reference Count: 31 ER -

M3 10.2147/JPR.S465058

N1 LiuMa-184

Y2 2025/1/17 17:41:00

N1 2025/2/13 1:16:00

DE knee osteoarthritis; acupuncture; exercise rehabilitation; HIP; MANAGEMENT; STATEMENT; PAIN

AB Objective: To assess the effectiveness of acupuncture, exercise rehabilitation, and their combination in treating Knee osteoarthritis (KOA) Methods: This randomized controlled trial was done on patients with KOA, who were randomly allocated to three groups: acupuncture (AP), exercise rehabilitation (ER), or a combination of acupuncture and exercise rehabilitation (AE). The study lasted 12 weeks with 4 weeks of treatment and 8 weeks of follow-up. The primary outcome was the response rate, which was determined by the percentage of participants who experienced a significant improvement in pain and function by the fourth week. The primary analysis utilized a Z test for proportions in the modified intent-to-treat population, consisting of all randomized participants with at least one post-baseline measurement. Results: Out of the 120 patients initially enrolled in the study, 110 completed the trial and were included in the intention-to-treat analysis. Response rates at week 4 were 65.7% (23 out of 35), 58.3% (21 out of 36), and 83.3% (32 out of 39) in the AP, ER, and AE groups, respectively. The response rate in the AE group was found to be significantly higher than that in the ER group at week 4. No significant differences were observed in the overall response rates between the AP and ER groups, as well as between the AP and AE groups. Conclusion: Our research indicates that both acupuncture and exercise rehabilitation can effectively enhance pain relief, functional improvement, and joint mobility in individuals aged 45 to 70 with moderate to severe chronic KOA. Furthermore, the AE group demonstrated the highest response rate. These beneficial outcomes were sustained for a minimum of 8 weeks post-treatment. The combination of acupuncture and exercise rehabilitation appears to enhance the overall therapeutic efficacy for KOA patients, suggesting a synergistic effect that may be particularly advantageous for those with moderate to severe symptoms.

C1 Chinese Peoples Liberat Army Gen Hosp, Med Ctr 6, Dept Acupuncture & Moxibust, Beijing, Peoples R China; Chinese Peoples Liberat Army Med Coll, Grad Sch, Beijing, Peoples R China

DI 10.2147/JPR.S465058

ID 184

ER

FN Clarivate Analytics Web of science

PT J

AU He, J.

AU - Zuo, J.

AU - Fan, X. C.

AU - Li, Z.

PY 2024

TI Electro-acupuncture modulated miR-214 expression to prevent chondrocyte apoptosis and reduce pain by targeting BAX and TRPV4 in osteoarthritis rats

DA 2024/1/1

SO BRAZILIAN JOURNAL OF MEDICAL AND BIOLOGICAL RESEARCH

JO BRAZILIAN JOURNAL OF MEDICAL AND BIOLOGICAL RESEARCH

VL 57

SN 0100-879X

Z9 Times Cited in Web of Science Core Collection: 1 Total Times Cited: 1 Cited Reference Count: 39 ER -

M3 10.1590/1414-431X2024e13238

N1 HeZuo-186

N1 2025/2/13 1:16:00

DE Osteoarthritis; Electro-acupuncture; miR-214; BAX; TRPV4; KNEE OSTEOARTHRITIS; ACUPUNCTURE

AB Osteoarthritis (OA) is a highly prevalent joint disorder characterized by progressive degeneration of articular cartilage, subchondral bone remodeling, osteophyte formation, synovial inflammation, and meniscal damage. Although the etiology of OA is multifactorial, pro-inflammatory processes appear to play a key role in disease pathogenesis. Previous studies indicate that electroacupuncture (EA) exerts chondroprotective, anti-inflammatory, and analgesic effects in preclinical models of OA, but the mechanisms underlying these potential therapeutic benefits remain incompletely defined. This study aimed to investigate the effects of EA on OA development in a rat model, as well as to explore associated molecular mechanisms modulated by EA treatment. Forty rats were divided into OA, EA, antagomiR-214, and control groups. Following intra-articular injection of monosodium iodoacetate to induce OA, EA and antagomiR-214 groups received daily EA stimulation at acupoints around the knee joint for 21 days. Functional pain behaviors and chondrocyte apoptosis were assessed as outcome measures. The expression of microRNA-214 (miR-214) and its downstream targets involved in apoptosis and nociception, BAX and TRPV4, were examined. Results demonstrated that EA treatment upregulated miR-214 expression in OA knee cartilage. By suppressing pro-apoptotic BAX and pro-nociceptive TRPV4, this EA-induced miR-214 upregulation ameliorated articular pain and prevented chondrocyte apoptosis. These findings suggested that miR-214 plays a key role mediating EA's therapeutic effects in OA pathophysiology, and represents a promising OA treatment target for modulation by acupuncture.

C1 Xi An Jiao Tong Univ, Xian Honghui Hosp, Dept Tradit Chinese Med Orthoped, Xian, Shaanxi, Peoples R China; Shaanxi Prov Hosp Chinese Med, Dept Acupuncture, Xian, Shaanxi, Peoples R China

DI 10.1590/1414-431X2024e13238

ID 186

ER

FN Clarivate Analytics Web of science

PT J

AU Mou, J. J.

AU - Wang, Q.

AU - Wu, J.

AU - Zhang, L. X.

AU - Li, Y. A.

AU - Luo, Z. C.

AU - An, J. Y.

AU - Sun, T.

AU - Zheng, X. L.

AU - Wang, Y.

AU - Hu, K. H.

PY 2024

TI The effect of Fu ' s subcutaneous needling in treating knee osteoarthritis patients: A randomized controlled trial

PG 562-571

DA 2024/1/1

SO EXPLORE-THE JOURNAL OF SCIENCE AND HEALING

JO EXPLORE-THE JOURNAL OF SCIENCE AND HEALING

VL 20

IS 4

SN 1550-8307

Z9 Times Cited in Web of Science Core Collection: 4 Total Times Cited: 4 Cited Reference Count: 43 ER -

M3 10.1016/j.explore.2023.12.015

N1 MouWang-205

N1 2025/2/13 1:16:00

DE Fu 's subcutaneous needling; Electroacupuncture; Knee osteoarthritis; MUSCLE; MANAGEMENT; COSTS

AB Background: Fu's subcutaneous needling (FSN) is an acupuncture technique for the treatment of soft tissue diseases. Knee osteoarthritis often involves lower limb muscles. This study aimed to observe and compare the clinical efficacy of Fu's subcutaneous acupuncture and electroacupuncture in the treatment of patients with knee osteoarthritis. Methods: 62 patients with early or medial stage of knee osteoarthritis were randomly divided into the FSN therapy group or the electroacupuncture(EA) therapy group (1:1). The Lysholm score, range of motion, and equilibrium function were observed over a 3-month follow-up period. A total of 60 participants completed the study. Results: Over the 3 months of follow-up, both treatment regimens showed equally favorable results on all prognostic measures compared with their respective baseline data (P<0.05). Compared with the EA group, the FSN group had a significantly greater improvement in claudication, joint stability, swelling, pain, and ROM after treatment (P<0.05). At 3 months after treatment, the FSN group revealed better scores of claudication, joint stability, swelling, walking up stairs, squatting, pain, ROM, and equilibrium function (forward and backward movement speed, left and right movement speed, movement ellipse area, movement length) compared to the EA group (all P<0.05). Conclusions: This study showed that FSN can significantly improve the pain symptoms, joint stability, and joint function of patients with knee osteoarthritis, and the clinical efficacy can be maintained at least 3 months after treatment.

C1 Suining Cent Hosp, Rehabil Med Dept, Suining, Peoples R China; Suining Cent Hosp, Dept Pain treatment, Suining, Peoples R China; Sichuan Univ, West China Hosp, Inst Integrated Tradit Chinese & Western Med, Div Internal Med, Chengdu, Peoples R China; Suining Cent Hosp, Teaching & Training Dept, Suining, Peoples R China

DI 10.1016/j.explore.2023.12.015

ID 205

ER

FN Clarivate Analytics Web of science

PT J

AU Yu, Y.

AU - Liu, C. Z.

AU - Wang, X. Z.

AU - Xi, Y. W.

AU - Fu, Y. M.

AU - Mi, B. H.

AU - Tu, J. F.

PY 2024

TI Effect of 4 weeks vs 8 weeks of acupuncture for knee osteoarthritis in China: protocol for a randomised controlled trial

DA 2024/1/1

SO BMJ OPEN

JO BMJ OPEN

VL 14

IS 1

SN 2044-6055

Z9 Times Cited in Web of Science Core Collection: 0 Total Times Cited: 0 Cited Reference Count: 44 ER -

M3 10.1136/bmjopen-2023-079709

N1 YuLiu-216

Y2 2025/1/17 14:04:00

N1 2025/2/13 1:16:00

DE COMPLEMENTARY MEDICINE; Protocols & guidelines; Musculoskeletal disorders; HIP OSTEOARTHRITIS; GLOBAL BURDEN; PAIN; MANAGEMENT; OUTCOMES; DISEASE

AB Introduction Knee osteoarthritis represents the prevalent and incapacitating disease. Acupuncture, a widely used clinical treatment for knee osteoarthritis, has been shown to ameliorate pain and enhance joint function in affected individuals. However, there is a lack of evidence comparing different courses of acupuncture for knee osteoarthritis. In this trial, we will assess the effect of 4 weeks vs 8 weeks of acupuncture in patients with knee osteoarthritis. Methods and analysis The protocol is a pragmatic, parallel, two-arm randomised controlled trial, with the data analyst and assessor being blinded. 148 eligible patients with knee osteoarthritis will be randomly allocated in a 1:1 ratio to receive 4-week or 8-week acupuncture. Electroacupuncture will be administered three times per week for 4 or 8 weeks, respectively. Patients with knee osteoarthritis in both groups will be followed up to 26 weeks. The primary outcome is the response rate at week 26, and secondary outcomes include knee joint pain, knee joint function, knee joint stiffness, quality of life, patient global assessment, the Osteoarthritis Research Society International response rate and rescue medicine. A cost-effectiveness analysis will be carried out over 26 weeks. Ethics and dissemination The protocol has been approved by the Medical Ethical Committee of Beijing University of Chinese Medicine (2023BZYL0506). The study findings will be disseminated through presentation in a medical journal. Additionally, we plan to present them at selected conferences and scientific meetings.

C1 Beijing Univ Chinese Med, Int Acupuncture & Moxibust Innovat Inst, Sch Acupuncture Moxibust & Tuina, Beijing, Peoples R China; Beijing Liangxiang Hosp, Acupuncture Moxibust Dept, Beijing, Peoples R China

DI 10.1136/bmjopen-2023-079709

ID 216

ER

FN Clarivate Analytics Web of science

PT J

AU Han, R.

AU - Guo, C. X.

AU - Lau, K.

AU - Hu, J. L.

PY 2024

TI Efficacy of knee osteoarthritis by use of laser acupuncture: A systematic review and meta-analysis

DA 2024/1/1

SO MEDICINE

JO MEDICINE

VL 103

IS 25

SN 0025-7974

Z9 Times Cited in Web of Science Core Collection: 1 Total Times Cited: 1 Cited Reference Count: 68 ER -

M3 10.1097/MD.0000000000038325

N1 HanGuo-272

Y2 2025/1/17 14:12:00

N1 2025/2/13 1:16:00

DE efficacy; laser acupuncture; meta-analysis; osteoarthritis; LOW-BACK-PAIN; DOUBLE-BLIND; THERAPY; JOINT; WOMAC; HIP

AB Background:Previous studies need to be aggregated and updated. We aim to assess the efficacy of laser acupuncture (LA) in knee osteoarthritis (OA) through a meta-analysis.Methods:Electronic databases were searched for studies investigating laser acupuncture's efficacy in managing OA. Data were collected from the beginning of each database to 2022 (up to March). The "WOMAC total score," "WOMAC stiffness score," "WOMAC pain score," "WOMAC physical function score," and "VAS score" were the key outcomes of interest. The Der Simonian-Laird method for random effects was used.Results:Twenty-five randomized controlled clinical trials met our criteria and were included (2075 patients). Comparisons of interest is the LA versus Sham LA (efficacy), LA versus. A (Acupuncture) (comparative effectiveness), LA combined with A versus A (effectiveness as an adjunct), and any other research used LA in their treatment. Laser irradiation is effective in patients with Knee OA. LA is also effective and has almost the same outcome as laser irradiation. LA can achieve almost the same effect as manual acupuncture, even better than acupuncture in some studies.Conclusion:Laser acupuncture is more or less effective in patients with OA; better efficacy will be achieved under appropriate laser parameters (810 nm, 785 nm) in the LA versus Sham LA group. Many studies have diverse results, possibly due to unstaged analysis of patients' disease, inappropriate selection of acupoints, lack of remote combined acupoints, and unreasonable laser parameters. Furthermore, a combination of acupoints was found to be more effective, which aligns with the combined-acupoints application of traditional Chinese medicine.

C1 City Univ Hong Kong, Dept Biomed Engn BME, 83 Tat Chee Ave, Hong Kong 999077, Peoples R China

DI 10.1097/MD.0000000000038325

ID 272

ER

FN Clarivate Analytics Web of science

PT J

AU Yuan, X. C.

AU - Guo, Y. X.

AU - Yi, H. Y.

AU - Hou, X. M.

AU - Zhao, Y. L.

AU - Wang, Y. Y.

AU - Jia, H.

AU - Baba, S. S.

AU - Li, M.

AU - Huo, F. Q.

PY 2024

TI Hemoglobin α-derived peptides VD-hemopressin (α) and RVD-hemopressin (α) are involved in electroacupuncture inhibition of chronic pain

DA 2024/1/1

SO FRONTIERS IN PHARMACOLOGY

JO FRONTIERS IN PHARMACOLOGY

VL 15

SN 1663-9812

Z9 Times Cited in Web of Science Core Collection: 0 Total Times Cited: 0 Cited Reference Count: 38 ER -

M3 10.3389/fphar.2024.1439448

N1 YuanGuo-284

Y2 2025/1/18 22:16:00

N1 2025/2/13 1:16:00

DE Knee osteoarthritis (KOA); Electroacupuncture analgesia; VD-hemopressin (alpha); RVD-hemopressin (alpha); 26S proteasome; Chronic pain; RAT; DEGRADATION; EXPRESSION; UBIQUITIN; CB1; VD-HEMOPRESSIN(ALPHA); OSTEOARTHRITIS; PROTEASOMES; NEURONS; PROTEIN

AB Introduction Knee osteoarthritis (KOA) is a chronic degenerative bone metabolic disease that primarily affects older adults, leading to chronic pain and disability that affect patients' daily activities. Electroacupuncture (EA) is a commonly used method for the treatment of chronic pain in clinical practice. Previous studies indicate that the endocannabinoid system is involved in EA analgesia, but whether endocannabinopeptide VD-hemopressin (alpha) and RVD-hemopressin (alpha) derived from hemoglobin chains are involved in EA analgesia is unclear.Methods RNA-seq technology was used to screen which genes involved in EA analgesia. The expression of hemoglobin alpha chain and 26S proteasome were determined by Western blotting. The level of VD-hemopressin (alpha) and RVD-hemopressin (alpha) were measured by UPLC-MS/MS. Microinjection VD-Hemopressin (alpha), RVD-Hemopressin (alpha) and 26S proteasome inhibitor MG-132 into vlPAG, then observe mechanical and thermal pain thresholds.Results Therefore, we used RNA-seq to obtain differentially expressed genes Hba-a1 and Hba-a2 involved in EA analgesia in the periaqueductal gray (PAG), which were translated into the hemoglobin alpha chain. EA significantly increased the expression of the hemoglobin alpha chain and the level of hemopressin (alpha) and RVD-hemopressin (alpha). Microinjection of VD-hemopressin (alpha) and RVD-hemopressin (alpha) into the ventrolateral periaqueductal gray (vlPAG) mimicked the analgesic effect of EA, while CB1 receptor antagonist AM251 reversed this effect. EA significantly increased the expression of 26S proteasome in KOA mice. Microinjection of 26S proteasome inhibitor MG132 before EA prevented both the anti-allodynic effect and upregulation of the concentration of RVD-hemopressin (alpha) by EA treatment and upregulated the expression of the hemoglobin alpha chain.Discussion Our data suggest that EA upregulated the concentration of VD-hemopressin (alpha) and RVD-hemopressin (alpha) through enhancement of the hemoglobin alpha chain degradation by 26S proteasome in the PAG, then activated the CB1 receptor, thereby exerting inhibition of chronic pain in a mouse model of KOA. These results provide new insights into the EA analgesic mechanisms and reveal possible targets for EA treatment of chronic pain.

C1 Xian Jiaotong Univ Hlth Sci Ctr, Sch Basic Med Sci, Dept Physiol & Pathophysiol, Xian, Peoples R China; Xi An Jiao Tong Univ, Minist Educ, Key Lab Environm & Genes Related Dis, Xian, Peoples R China; Bayero Univ Kano, Coll Hlth Sci, Fac Basic Med Sci, Dept Human Physiol,Neurosci & Pathophysiol Unit, Kano, Nigeria; Huazhong Univ Sci & Technol, Inst Brain Res, Tongji Med Coll, Sch Basic Med,Dept Neurobiol, Wuhan, Peoples R China; Huazhong Univ Sci & Technol, Inst Brain Res,Minist Educ, Tongji Med Coll,Key Lab Neurol Dis, Sch Basic Med, Wuhan, Peoples R China

DI 10.3389/fphar.2024.1439448

ID 284

ER

FN Clarivate Analytics Web of science

PT J

AU Yuan, Y.

AU - Li, P.

PY 2024

TI Effect of warm needle therapy guided by ultrasound on pain relief and improvement of physical function in patients with knee osteoarthritis

PG 7467-7479

DA 2024/1/1

SO AMERICAN JOURNAL OF TRANSLATIONAL RESEARCH

JO AMERICAN JOURNAL OF TRANSLATIONAL RESEARCH

VL 16

IS 12

SN 1943-8141

Z9 Times Cited in Web of Science Core Collection: 0 Total Times Cited: 0 Cited Reference Count: 44 ER -

M3 10.62347/KFNE8547

N1 YuanLi-309

N1 2025/2/13 1:16:00

DE Knee osteoarthritis (KOA); warm needle therapy; ultrasound guidance; meloxicam; pain relief; knee; function; patient satisfaction; inflammatory factor; ACUPUNCTURE

AB Background: Conventional treatments for knee osteoarthritis (KOA) often fall short in providing optimal outcomes. Objective: To evaluate the effect of warm needle therapy guided by ultrasound on pain relief and physical function in patients with KOA. Methods: In this retrospective study, the clinical records of patients with KOA undergoing either meloxicam alone or meloxicam combined with warm needle therapy were reviewed. Various parameters, including pain evaluations, Western Ontario and McMaster Universities Osteoarthritis Index (WOMAC) scores, range of motion for knee flexion, knee society scores, quality of life scores, inflammatory factor levels, and patient satisfaction were comparatively analyzed. Results: A total of 140 patients were included, including 68 patients treated by Meloxicam and 72 patients treated by Meloxicam and warm needle therapy. Following treatment, the meloxicam combined with warm needle therapy group exhibited lower pain scores (3.62 +/- 1.98 vs. 4.38 +/- 1.95, P=0.023), improved WOMAC scores (27.82 +/- 8.75 vs. 31.25 +/- 8.82, P=0.022), increased range of motion (136.82 degrees +/- 8.58 degrees vs. 133.43 degrees +/- 8.86 degrees, P=0.023), higher knee society scores (93.32 +/- 7.21 vs. 90.21 +/- 7.78, P=0.016), and superior Short Form-36 Health Survey (SF-36) scores (81.24 +/- 6.33 vs. 78.43 +/- 6.85, P=0.013). Furthermore, a significant reduction in inflammatory factors including interleukin-8 (IL-8), matrix metalloproteinase-3 (MMP-3), Interleukin-1 beta (IL-1 beta), Tumor Necrosis Factor-alpha (TNF-alpha), and cyclooxygenase-2 (COX-2) levels was observed in the meloxicam combined with warm needle therapy group compared to the meloxicam alone group (all P<0.05). Patient satisfaction was likewise notably higher in the meloxicam combined with warm needle therapy group (8.43 +/- 2.15 vs. 7.58 +/- 2.24, P=0.024), with a greater proportion of patients willing to recommend the treatment (81.94% vs. 64.71%, P=0.034). Conclusion: Warm needle therapy guided by ultrasound, in combination with meloxicam, significantly improves pain relief, physical function, inflammatory modulation, and patient satisfaction in KOA patients.

C1 Henan Vocat Univ Sci & Technol, Med Coll, 6 East Sect Wenchang Ave, Zhoukou 466000, Henan, Peoples R China; Zhoukou City Sixth Peoples Hosp, Tradit Chinese Med Dept, Zhoukou 466000, Henan, Peoples R China

DI 10.62347/KFNE8547

ID 309

ER

FN Clarivate Analytics Web of science

PT J

AU Li, H.

AU - Yang, C. C.

AU - Bai, T. Y.

AU - Sun, J.

AU - Fu, Z. H.

AU - Mi, J.

AU - Chou, L. W.

PY 2024

TI The Impact of Fu's Subcutaneous Needling on Lower Limb Muscle Stiffness in Knee Osteoarthritis Patients: Study Protocol for a Pilot Randomized Controlled Trial

PG 3315-3326

DA 2024/1/1

SO JOURNAL OF PAIN RESEARCH

JO JOURNAL OF PAIN RESEARCH

VL 17

SN 1178-7090

Z9 Times Cited in Web of Science Core Collection: 3 Total Times Cited: 3 Cited Reference Count: 63 ER -

M3 10.2147/JPR.S482082

N1 LiYang-321

Y2 2025/1/17 14:05:00

N1 2025/2/13 1:16:00

DE Fu's subcutaneous needling; electroacupuncture; muscle stiffness; knee osteoarthritis; shear wave elastography; SHEAR-WAVE ELASTOGRAPHY; QUADRICEPS WEAKNESS; ACUPUNCTURE; PAIN; VALIDATION; QUALITY; THERAPY; DISEASE; AGE; HIP

AB Background: Knee osteoarthritis (OA) is a leading cause of disability worldwide, with clinicians often observing increased muscle stiffness associated with joint pain and dysfunction. This study examines the impact of Fu's Subcutaneous Needling (FSN), a nonpharmacological technique, on muscle stiffness in the lower limbs of individuals with knee OA. Materials and Methods: This study protocol is a pilot, single-center, randomized controlled trial. Sixty knee OA patients will be allocated equally for FSN or electroacupuncture (EA) treatments. Interventions will be applied thrice weekly for the first two weeks and twice weekly for the subsequent two weeks for a total of ten sessions. Assessments will be conducted at baseline, post-initial session, after four weeks of intervention, and at the end of a four-week follow-up. The primary outcome will be the muscle stiffness in the lower extremities, as measured by shear wave elastography (SWE). Secondary outcomes include response rate, a reduction in the mean pain intensity on the Numerical Rating Scale (NRS) by at least two points and on the Western Ontario and McMaster Universities Osteoarthritis Index (WOMAC) function subscale by six points at week four compared with baseline. Weekly monitoring of the NRS and WOMAC scores will determine the rapidity of pain alleviation and functional improvement, along with 12-item short-form (SF-12) score changes from baseline to week four. Results: This is the first standardized protocol examining the effects of FSN on lower limb muscle stiffness in patients with knee OA by SWE. We hypothesize that FSN could outperform EA in alleviating lower limb stiffness associated with knee OA. Findings will contribute to the body of knowledge regarding the efficacy of acupuncture-derived interventions in managing muscle stiffness and may guide future research directions.

C1 Shandong Univ, Shandong Prov Hosp 3, Dept Acupuncture Moxibust & Tuina, Jinan 250031, Peoples R China; Shandong Univ Tradit Chinese Med, Sch Acupuncture & Tuina, Jinan 250355, Peoples R China; Shandong Univ, Shandong Prov Hosp 3, Dept Ultrasound, Jinan 250031, Peoples R China; Guangzhou Univ Chinese Med, Clin Med Coll Acupuncture & Moxibust & Rehabil, Guangzhou 510405, Peoples R China; Guangzhou Univ Chinese Med, Clin Med Coll 2, Guangzhou 510006, Peoples R China; Beijing Univ Chinese Med, Inst Fus Subcutaneous Needling, Beijing 100029, Peoples R China; China Med Univ, China Med Univ Hosp, Dept Phys Med & Rehabil, 2 Yuh Der Road, Taichung 404332, Taiwan; China Med Univ, Dept Phys Therapy, Taichung 406040, Taiwan; China Med Univ, Grad Inst Rehabil Sci, Taichung 406040, Taiwan; Asia Univ, Asia Univ Hosp, Dept Phys Med & Rehabil, Taichung 413505, Taiwan

DI 10.2147/JPR.S482082

ID 321

ER

FN Clarivate Analytics Web of science

PT J

AU Jin, K.

AU - Zheng, A. J.

AU - Zheng, F. F.

AU - Mu, J. J.

AU - Chi, M.

PY 2024

TI EFFICACY OF OPPOSING NEEDLING TECHNIQUE IN MANAGING KNEE OSTEOARTHRITIS AMONG ATHLETES: A META-ANALYSIS

PG 396-413

DA 2024/1/1

SO REVISTA INTERNACIONAL DE MEDICINA Y CIENCIAS DE LA ACTIVIDAD FISICA Y DEL DEPORTE

JO REVISTA INTERNACIONAL DE MEDICINA Y CIENCIAS DE LA ACTIVIDAD FISICA Y DEL DEPORTE

VL 24

IS 97

SN 1577-0354

Z9 Times Cited in Web of Science Core Collection: 0 Total Times Cited: 0 Cited Reference Count: 30 ER -

N1 JinZheng-325

N1 2025/2/13 1:16:00

DE Contralateral Point Selection; Acupuncture; Knee Osteoarthritis; Meta-analysis; Literature Research

AB Objective: This meta-analysis aims to assess the clinical efficacy of opposing needling for treating knee osteoarthritis (KOA) in athletes, focusing on pain management, mobility improvement, and overall recovery, which are critical for maintaining athletic performance. Methods: A systematic search was conducted across several databases including China National Knowledge Infrastructure, China Biology Medicine disc, Wanfang Data Knowledge Service Platform, VIP Database for Chinese Technical Periodicals, PubMed, Web of Science, Cochrane Library, and EMBASE. Randomized controlled trials (RCTs) were selected based on strict inclusion and exclusion criteria. Data extraction and quality assessment were performed, followed by a meta-analysis using Revman 5.4 and Stata 14.0 software. Results: The meta-analysis revealed a higher clinical efficacy rate in the opposing needling group compared to the control group (RR=1.18, 95%CI [1.12,1.25], P<0.0001). Significant improvements were also observed in pain reduction, as indicated by lower VAS scores (MD=-1.31, 95%CI [-1.70,-0.93], P<0.0001), and in functional mobility, as reflected by improved WOMAC scores (MD=-10.17, 95%CI [-13.64,-6.69], P<0.0001). However, the differences in Lysholm scoring, which relates specifically to knee stability and function in sports, did not show statistical significance (MD=18.37, 95% CI [-1.02, 37.75], P=0.06), potentially due to limited studies focusing on this outcome. Conclusion: Opposing needling is an effective and reliable intervention for managing KOA in athletes. It shows significant advantages in enhancing clinical outcomes, reducing pain, and improving stiffness and daily activities compared to other treatments. Given its benefits in promoting quicker recovery and maintaining athletic performance, opposing needling therapy is recommended for broader clinical application in sports medicine.

C1 Peking Univ Third Hosp, Beijing Yanqing Dist Hosp, Yanqing Hosp, Beijing 102100, Peoples R China

ID 325

ER

FN Clarivate Analytics Web of science

PT J

AU Duan, Y. S.

AU - Wang, Y. R.

AU - Li, B. Y.

AU - Fu, Z. T.

AU - Tu, J. F.

AU - Zhou, H.

AU - Wang, Y.

AU - Wang, L. Q.

AU - Liu, C. Z.

PY 2024

TI Overall Reporting Quality of Randomized Controlled Trials of Acupuncture for Knee Osteoarthritis: A Systematic Review

PG 3371-3383

DA 2024/1/1

SO JOURNAL OF PAIN RESEARCH

JO JOURNAL OF PAIN RESEARCH

VL 17

SN 1178-7090

Z9 Times Cited in Web of Science Core Collection: 0 Total Times Cited: 0 Cited Reference Count: 46 ER -

M3 10.2147/JPR.S477000

N1 DuanWang-326

N1 2025/2/13 1:16:00

DE randomized controlled trials; acupuncture; knee osteoarthritis; CONSORT; STRICTA; outcomes; CONSORT STATEMENT; GUIDELINES; BIAS; METAANALYSIS; THERAPY; IMPACT

AB Objective: To evaluate the reporting quality of randomized controlled trials (RCT) of acupuncture for knee osteoarthritis and explore factors associated with the reporting. Study Design and Setting: Eight databases were searched from inception to August 2024 to assess the quality of acupuncture for knee osteoarthritis RCTs based on the CONSORT, the STRICTA, and the CONSORT-Outcomes. We performed regression analyses on pre-specified study characteristics to explore factors associated with reporting quality. Results: One hundred and seventy-four RCTs were evaluated by 69 items from 3 checklists. Seventeen of 37 items on the CONSORT were under-reported (reported in less than 20% of RCTs), and the weakest reported item was why the trial ended or was stopped (0%). Four of 17 items on the STRICTA were under-reported, and the weakest reported item was the number of needle insertions per subject per session (9.2%). Eight of 17 items on the CONSORT-Outcomes were under-reported, and the weakest reported item was identifying any outcomes that were not pre-specified in a trial registry or trial protocol (0.6%). RCT locations include countries other than China, published in English, or funded were more likely to have better reporting. Conclusion: RCTs of acupuncture for knee osteoarthritis need to focus more on reporting details of acupuncture interventions, the reporting of protocol amendment, and the complete reporting of outcome-related content. Journals should encourage authors to adhere strictly to reporting guidelines, which is necessary to improve the quality of reporting, which is very important for Chinese journals.

C1 Beijing Univ Chinese Med, Int Acupuncture & Moxibust Innovat Inst, Sch Acupuncture Moxibust & Tuina, 11 Bei San Huan Dong Lu, Beijing 100029, Peoples R China

DI 10.2147/JPR.S477000

ID 326

ER

FN Clarivate Analytics Web of science

PT J

AU Xing, B. F.

AU - Liu, Y. Y.

AU - Zhou, X.

AU - He, G. H.

AU - Pei, W. Y.

AU - Liang, Z. M.

AU - Ruan, J. W.

AU - Duan, Y. H.

PY 2024

TI Electroacupuncture with extensor exercise improves the contraction elastic density of quadriceps in short and long term for knee osteoarthritis

DA 2024/1/1

SO CLINICAL RHEUMATOLOGY

JO CLINICAL RHEUMATOLOGY

SN 0770-3198

Z9 Times Cited in Web of Science Core Collection: 0 Total Times Cited: 0 Cited Reference Count: 29 ER -

M3 10.1007/s10067-024-07243-5

N1 XingLiu-370

Y2 2025/1/17 14:03:00

N1 2025/2/13 1:16:00

DE Elastic shear wave imaging; Electroacupuncture (EA); Extensor training; Knee osteoarthritis (OA); Quadriceps femoris; WOMAC scoring scale; MUSCLE STRENGTH; EPIDEMIOLOGY; INCREASE

AB BackgroundExtensor training improves the quadriceps contraction intensity of knee osteoarthritis. But the duration of effects is limited. This study aimed to assess whether electroacupuncture (EA) with extensor training (EA + E) has better short-and long-term effects than simple extensor training (E).MethodsFifty-four patients were categorized into EA + E and E groups. Elastic shear wave imaging was employed to ascertain the quadriceps elastic modulus values (EMV) at different time points and extension angles. WOMAC scale was used to measure knee function.ResultsCompared to before EA, the vastus lateralis (VL) EMV with 180 degrees knee extension immediately after EA increased significantly. After 1 month, the vastus medialis (VM) EMV with 180 degrees extension increased and WOMAC scores decreased significantly than immediately after EA. The EMV of the VM and VL of group EA + E increased, and WOMAC scores decreased significantly compared to group E after 1 month. After 6 months, EMV of the VM and VL in EA + E group significantly increased, and WOMAC scores significantly decreased compared to E group.ConclusionsIn short- and long-term, EA with extensor training enhances the contraction strength of the quadriceps and knee function for OA.Trial registrationThis prospective single-center randomized controlled trial was approved by the Review Committee of the First Affiliated Hospital of Sun Yat-sen University (Registration date: 28/02/2023, Ethical Number: [2023] 005; The Clinical trial registration number: ChiCTR2300076651.Key Points center dot The contraction strength of the quadriceps femoris muscle in OA was detected with shear wave elastic ultrasound, and electroacupuncture immediately enhanced the contraction strength.center dot After the completion of electroacupuncture treatment, the quadriceps strength and the function of knee joint in OA can be continuously improved, which will last for 6 months.center dot In short and long term, electroacupuncture with extensor training has better enhancement of quadriceps contraction strength of and knee function for OA than extention only.Key Points center dot The contraction strength of the quadriceps femoris muscle in OA was detected with shear wave elastic ultrasound, and electroacupuncture immediately enhanced the contraction strength.center dot After the completion of electroacupuncture treatment, the quadriceps strength and the function of knee joint in OA can be continuously improved, which will last for 6 months.center dot In short and long term, electroacupuncture with extensor training has better enhancement of quadriceps contraction strength of and knee function for OA than extention only.Key Points center dot The contraction strength of the quadriceps femoris muscle in OA was detected with shear wave elastic ultrasound, and electroacupuncture immediately enhanced the contraction strength.center dot After the completion of electroacupuncture treatment, the quadriceps strength and the function of knee joint in OA can be continuously improved, which will last for 6 months.center dot In short and long term, electroacupuncture with extensor training has better enhancement of quadriceps contraction strength of and knee function for OA than extention only.

C1 Guangdong Pharmaceut Univ, Affiliated Hosp 1, Clin Med Sch 1, Guangzhou, Peoples R China; Sun Yat Sen Univ, Affiliated Hosp 1, Dept Rheumatol, Guangzhou, Peoples R China

DI 10.1007/s10067-024-07243-5

ID 370

ER

FN Clarivate Analytics Web of science

PT J

AU Huang, H.

AU - Tang, K. M.

AU - Song, X. L.

AU - Zhao, L.

AU - Liang, Y. Y.

AU - Xu, H.

AU - Xiao, L. B.

AU - Chen, Y. L.

PY 2024

TI Effects of contralateral versus ipsilateral electroacupuncture for analgesia and rehabilitation after unilateral total knee arthroplasty: a randomized controlled trial

PG 183-193

DA 2024/1/1

SO ACUPUNCTURE IN MEDICINE

JO ACUPUNCTURE IN MEDICINE

VL 42

IS 4

SN 0964-5284

Z9 Times Cited in Web of Science Core Collection: 1 Total Times Cited: 1 Cited Reference Count: 48 ER -

M3 10.1177/09645284231211601

N1 HuangTang-376

N1 2025/2/13 1:16:00

DE contralateral needling; electroacupuncture; knee osteoarthritis; ipsilateral needling; pain management; TOTAL HIP; ACUPUNCTURE; PAIN; REPLACEMENT; SATISFACTION; MECHANISMS; RECOVERY; RELIEF

AB Purpose: Total knee arthroplasty (TKA) is a treatment for advanced knee osteoarthritis. Since postoperative pain affects rehabilitation, this study aimed to determine whether electroacupuncture (EA) contralateral to the surgical site is more effective than ipsilateral EA or sham EA in terms of relieving postoperative pain and promoting post-TKA rehabilitation. Methods: In this parallel, single-blind randomized controlled trial, 114 patients undergoing unilateral TKA were assigned to the contralateral EA (EA on the contralateral side + sham EA on the ipsilateral), ipsilateral EA (EA on the ipsilateral + sham EA on the contralateral side), or sham EA (sham EA on both sides) groups (n = 38 each). Treatment was performed once daily on postoperative days 1-3. The visual analog scale (VAS) scores, additional opioid doses via patient-controlled analgesia (PCA) pump, Hospital for Special Surgery (HSS) knee scores, active/passive range of motion (AROM/PROM), swelling around the knee joint, and Hamilton anxiety scale (HAMA) scores were used for postoperative evaluation. Results: At 3 days postoperatively, the VAS scores, HSS scores, AROM/PROM, swelling around the knee, and HAMA scores in the contralateral EA and ipsilateral EA groups were significantly improved compared with baseline. In addition, VAS scores, HSS scores, PROM and swelling around the knee were significantly better in the contralateral and ipsilateral EA groups than in the sham EA group, but similar in the two true EA groups. Furthermore, PCA additional dose release was significantly higher in the sham EA group than in the two true EA groups (which did not significantly differ). At 10 days postoperatively, the HSS scores, AROM/PROM, and HAMA scores were better in the contralateral and ipsilateral EA groups than in the sham EA group, but similar in the two true EA groups. Conclusion: Contralateral EA is more effective than sham EA for treating postoperative pain following TKA, but has an analgesic effect similar to that of ipsilateral EA.

C1 Shanghai Univ Tradit Chinese Med, Shenzhen Hosp, Shanghai, Peoples R China; Shanghai Univ Tradit Chinese Med, Shanghai, Peoples R China; Shanghai Univ Tradit Chinese Med, Guanghua Hosp, Shanghai, Peoples R China; Shanghai Univ Tradit Chinese Med, LongHua Hosp, Shanghai, Peoples R China

DI 10.1177/09645284231211601

ID 376

ER

FN Clarivate Analytics Web of science

PT J

AU Song, Y. F.

AU - Du, X. Y.

AU - Che, X. L.

AU - Li, M. S.

AU - Zhang, R. J.

AU - Wang, D. M.

AU - Shi, Q. F.

PY 2024

TI Analysis of rehabilitation effect of electroacupuncture for patients after total knee arthroplasty

DA 2024/1/1

SO DISABILITY AND REHABILITATION

JO DISABILITY AND REHABILITATION

SN 0963-8288

Z9 Times Cited in Web of Science Core Collection: 0 Total Times Cited: 0 Cited Reference Count: 28 ER -

M3 10.1080/09638288.2024.2402506

N1 SongDu-397

N1 2025/2/13 1:16:00

DE Electroacupuncture; total knee arthroplasty; visual analogue scale (VAS); knee Society score (KSS); range of motion (ROM); OUTCOMES; PAIN

AB Purpose To investigate the effect of electroacupuncture on the rehabilitation of patients after total knee arthroplasty (TKA). Materials and methods Eighty patients with knee osteoarthritis who underwent total knee arthroplasty randomly divided into two groups, with 40 patients in each group. The control group was treated with traditional rehabilitation methods. In addition to traditional rehabilitation treatment, patients in the experimental group received electroacupuncture after operation, while patients in the control group received fake electroacupuncture. Both groups started electroacupuncture treatment and fake electroacupuncture treatment on the third day after operation for 10 consecutive days Data of patients in both groups were collected before surgery, three days after surgery, two weeks after surgery and one month after surgery, including the visual analogue scale(VAS), Knee Society Score (KSS) and range of motion (ROM) Results Compared with before treatment, after the treatment cycle, the VAS, KSS and ROM of both groups were significantly improved (p = 0.001, p = 0.001). Compared with the control group, the ROM and KSS of the experimental group were significantly improved at two weeks after surgery and one month after surgery, and the VAS was significantly decreased, with statistical significance (p < 0.05). Conclusions Electroacupuncture therapy has a positive effect on the recovery of patients after total knee arthroplasty, which can alleviate the pain after total knee arthroplasty, promote the recovery of knee function, which is worthy of clinical promotion.

C1 Shanxi Med Univ, Hosp 2, Rehabil Dept, Taiyuan, Peoples R China; Shanxi Med Univ, Clin Med Coll 2, Taiyuan, Peoples R China; Shanxi Med Univ, Publ Hlth Coll, Taiyuan, Peoples R China; Shanxi Med Univ, Hosp 2, Orthopaed, Taiyuan 030001, Peoples R China; Xinghualing Dist Cent Hosp, Dept Rehabil, Taiyuan, Peoples R China

DI 10.1080/09638288.2024.2402506

ID 397

ER

FN Clarivate Analytics Web of science

PT J

AU Qi, B.

AU - Wang, Z. Y.

AU - Cao, Y.

AU - Zhao, H. S.

PY 2024

TI Study on the treatment of osteoarthritis by acupuncture combined with traditional Chinese medicine based on pathophysiological mechanism: A review

DA 2024/1/1

SO MEDICINE

JO MEDICINE

VL 103

IS 14

SN 0025-7974

Z9 Times Cited in Web of Science Core Collection: 0 Total Times Cited: 0 Cited Reference Count: 64 ER -

M3 10.1097/MD.0000000000037483

N1 QiWang-419

Y2 2025/1/17 14:07:00

N1 2025/2/13 1:16:00

DE acupuncture; acupuncture combined with TCM; OA; pathophysiological; TCM; KNEE OSTEOARTHRITIS; HEALTH-CARE; RISK; RECOMMENDATIONS; PREVALENCE; PEOPLE; IMPACT

AB Osteoarthritis (OA) is a major contributor to disability and social costs in the elderly. As the population ages and becomes increasingly obese, the incidence of the disease is higher than in previous decades. In recent years, important progress has been made in the causes and pathogenesis of OA pain. Modern medical treatment modalities mainly include the specific situation of the patient and focus on the core treatment, including self-management and education, exercise, and related weight loss. As an important part of complementary and alternative medicine, TCM has remarkable curative effect, clinical safety, and diversity of treatment methods in the treatment of OA. Traditional Chinese Medicine treatment of OA has attracted worldwide attention. Therefore, this article will study the pathophysiological mechanism of OA based on modern medicine, and explore the treatment of OA by acupuncture combined with Chinese Medicine.

C1 Shenzhen Baoan Dist Shiyan Peoples Hosp, Shenzhen, Peoples R China; Shenzhen Pingshan Dist Hosp Tradit Chinese Med, Shenzhen, Peoples R China; Community Hlth Serv Ctr Nanhui New Town, Shanghai 200120, Peoples R China

DI 10.1097/MD.0000000000037483

ID 419

ER

FN Clarivate Analytics Web of science

PT J

AU Huang, H.

AU - Liu, R. X.

AU - Shao, J. Y.

AU - Chen, S. Y.

AU - Sun, J.

AU - Zhu, J. X.

PY 2024

TI Biomechanically based Fu's subcutaneous needling treatment for senile knee osteoarthritis: protocol for a randomized controlled trial

DA 2024/1/1

SO JOURNAL OF ORTHOPAEDIC SURGERY AND RESEARCH

JO JOURNAL OF ORTHOPAEDIC SURGERY AND RESEARCH

VL 19

IS 1

SN 1749-799X

Z9 Times Cited in Web of Science Core Collection: 0 Total Times Cited: 0 Cited Reference Count: 53 ER -

M3 10.1186/s13018-024-04878-7

N1 HuangLiu-485

Y2 2025/1/17 14:06:00

N1 2025/2/13 1:16:00

DE Fu's subcutaneous needling; Knee osteoarthritis; Gait analysis; Muscle elasticity; FUNCTIONAL PERFORMANCE; MUSCLE; MANAGEMENT; HIP; INDIVIDUALS; GUIDELINE; GENESIS

AB IntroductionFu's subcutaneous needling (FSN) is a new type of acupuncture that uses subcutaneous tissue to oscillate from side to side to improve muscle pathology status and can be effective in treating Knee osteoarthritis. Nonetheless, whether the clinical effect is similar to that of most commonly used drugs is unclear. Thus, this study aims to determine the pain-relieving effect and improvement in the joint function of the FSN therapy by comparing it with that of a positive control drug (celecoxib). Furthermore, this clinical trial also aims to evaluate the effect of FSN on gait and lower limb muscle flexibility, which can further explore the scientific mechanisms of the FSN therapy.Methods and analysisThis study is a randomized, parallel-controlled, single-center prospective clinical study that includes 60 participants, with an FSN group (n = 30) and a drug group (n = 30). The Fu's subcutaneous needling (FSN) group undergo the FSN therapy 3 times a week for 2 weeks, while the drug group receives 0.2 g/day oral celecoxib for 2 weeks, with a follow-up period of 4 weeks after the completion of treatment. The primary outcome is the difference in the visual analog scale score after 2 weeks of treatment compared with baseline. The Western Ontario and McMaster Universities (WOMAC) Osteoarthritis Index, joint active range of motion test, three-dimensional gait analysis, and shear wave elastic imaging technology analysis in lower limb muscles are also performed to demonstrate clinical efficacy.Ethics and disseminationThe trial is performed following the Declaration of Helsinki. The study protocol and consent form have been approved by the Ethics Committee of Guangdong Provincial Hospital of Chinese Medicine. All patients will give informed consent before participation and the trial is initiated after approval. The results of this trial will be disseminated through publication in peer-reviewed journals.Trial registration number: NCT06328153.Ethics and disseminationThe trial is performed following the Declaration of Helsinki. The study protocol and consent form have been approved by the Ethics Committee of Guangdong Provincial Hospital of Chinese Medicine. All patients will give informed consent before participation and the trial is initiated after approval. The results of this trial will be disseminated through publication in peer-reviewed journals.Trial registration number: NCT06328153.

C1 Guangzhou Univ Chinese Med, Clin Coll 2, Guangzhou, Guangdong, Peoples R China; Guangzhou Univ Chinese Med, Clin Med Sch 8, Guangzhou, Guangdong, Peoples R China; Guangzhou Univ Chinese Med, Clin Med Coll Acupuncture & Moxibust & Rehabil, Guangzhou, Guangdong, Peoples R China; Guangzhou Univ Chinese Med, Affiliated Hosp 1, Baiyun Hosp, Guangzhou, Guangdong, Peoples R China

DI 10.1186/s13018-024-04878-7

ID 485

ER

FN Clarivate Analytics Web of science

PT J

AU Luo, X. C.

AU - Liu, J. L.

AU - Li, Q. R.

AU - Zhao, J. P.

AU - Hao, Q. K.

AU - Zhao, L.

AU - Chen, Y. M.

AU - Yin, P. B.

AU - Li, L.

AU - Liang, F. R.

AU - Sun, X.

PY 2023

TI Acupuncture for treatment of knee osteoarthritis: A clinical practice guideline

PG 237-245

DA 2023/1/1

SO JOURNAL OF EVIDENCE BASED MEDICINE

JO JOURNAL OF EVIDENCE BASED MEDICINE

VL 16

IS 2

SN 1756-5383

Z9 Times Cited in Web of Science Core Collection: 12 Total Times Cited: 14 Cited Reference Count: 43 ER -

M3 10.1111/jebm.12526

N1 LuoLiu-23

Y2 2025/1/18 22:03:00

N1 2025/2/13 1:16:00

DE acupuncture; clinical practice guideline; knee osteoarthritis; rapid recommendation; EXERCISE THERAPY; PAIN; REHABILITATION; MANAGEMENT; DIAGNOSIS; HIP

AB Clinical questionIs acupuncture effective in treating knee osteoarthritis (KOA)? Current practiceAlthough increasingly used in the clinical setting, acupuncture is not mentioned or weakly recommended in guidelines for the treatment of KOA. RecommendationsWe suggest acupuncture rather than no treatment in adult KOA (weak recommendation, moderate certainty evidence), and acupuncture combined with nonsteroidal anti-inflammatory drugs (NSAIDs) rather than acupuncture alone when KOA symptoms are severe (weak recommendation, moderate certainty evidence), with duration of acupuncture for 4-8 weeks depending on KOA severity and treatment response (weak recommendation, moderate certainty evidence), and discussing with patients in shared decision-making. How this guideline was createdThis rapid recommendation was developed following the Making GRADE the Irresistible Choice (MAGIC) methodological framework. First, the clinical specialist identified the topic of recommendation and demand for evidence. Then the independent evidence synthesis group performed a systematic review to summarize available evidence and evaluate the evidence using the GRADE approach. Finally, the clinical specialist group produced recommendations for practice through a consensus procedure. The evidenceThe linked systematic review and meta-analysis included 9422 KOA patients, 61.1% of whom were women. The median mean age was 61.8 years. Compared with no treatment, acupuncture had beneficial effect on KOA in improving the Western Ontario and McMaster Universities Osteoarthritis Index (WOMAC) total score (moderate certainty evidence), and WOMAC pain (very low certainty evidence), WOMAC stiffness (low certainty evidence), and WOMAC function (low certainty evidence) subscale scores. Compared with usual care, acupuncture improved WOMAC stiffness subscale score (moderate certainty evidence). Subgroup analyses showed different effects in the improvement of WOMAC total scores by different durations of acupuncture and whether acupuncture combined with NSAIDs, but no difference between manual acupuncture and electroacupuncture was found. Understanding the recommendationsCompared with no treatment, acupuncture is suggested to reduce pain, stiffness, and disfunction in KOA patients, ultimately improving the patient's health status. Acupuncture can be used as an alternative therapy when usual care is ineffective or there are adverse reactions so that patients can no longer continue the treatment. Manual acupuncture or electroacupuncture is suggested for 4-8 weeks to improve the health status of KOA. The patient's values and preferences should be considered when selecting acupuncture for KOA treatment.

C1 Sichuan Univ, Chinese Evidence Based Med Ctr, Cochrane China Ctr, Chengdu, Peoples R China; Sichuan Univ, West China Hosp, MAGIC China Ctr, Chengdu, Peoples R China; NMPA Key Lab Real World Data Res & Evaluat Hainan, Chengdu, Peoples R China; Sichuan Ctr Technol Innovat Real World Data, Chengdu, Peoples R China; Sichuan Univ, West China Hosp, Dept Nucl Med, Chengdu, Peoples R China; Beijing Univ Chinese Med, Dongzhimen Hosp, Dept Acupuncture & Moxibust, Beijing, Peoples R China; Sichuan Univ, West China Hosp, Ctr Gerontol & Geriatr, Natl Clin Res Ctr Geriatr, Chengdu, Peoples R China; McMaster Univ, Sch Rehabil Sci, Hamilton, ON, Canada; Chengdu Univ Tradit Chinese Med, Acupuncture & Tuina Sch, Chengdu, Peoples R China; New York Coll Tradit Chinese Med, Mineola, NY USA; Chinese Peoples Liberat Army Gen Hosp, Dept Orthoped, Beijing, Peoples R China; Natl Clin Res Ctr Orthoped Sports Med & Rehabil, Beijing, Peoples R China

DI 10.1111/jebm.12526

ID 23

ER

FN Clarivate Analytics Web of science

PT J

AU Chen, X. L.

AU - Guo, Y.

AU - Lu, J.

AU - Qin, L. X.

AU - Hu, T. Y.

AU - Zeng, X.

AU - Wang, X. Y.

AU - Zhang, A. R.

AU - Zhuang, Y. X.

AU - Zhong, H. G.

AU - Guo, C. Q.

PY 2023

TI Acupotomy ameliorates subchondral bone absorption and mechanical properties in rabbits with knee osteoarthritis by regulating bone morphogenetic protein 2-Smad1 pathway

PG 734-743

DA 2023/1/1

SO JOURNAL OF TRADITIONAL CHINESE MEDICINE

JO JOURNAL OF TRADITIONAL CHINESE MEDICINE

VL 43

IS 4

SN 0255-2922

Z9 Times Cited in Web of Science Core Collection: 1 Total Times Cited: 1 Cited Reference Count: 47 ER -

M3 10.19852/j.cnki.jtcm.20230404.001

N1 ChenGuo-25

N1 2025/2/13 1:16:00

DE acupuncture therapy; osteoarthritis; knee; subchondral bone; mechanical properties; bone morphogenetic protein 2; Smad1 protein; TRABECULAR BONE; DISEASE; MODEL

AB OBJECTIVE: To investigate the effects of acupotomy on the subchondral bone absorption and mechanical properties in rabbits with knee osteoarthritis (KOA).METHODS: The rabbits were divided into blank control, model, acupotomy and electroacupuncture (EA) groups, with 12 rabbits in each. Modified Videman's method was used to prepare KOA model. The acupotomy and EA group were given indicated intervention for 3 weeks. The behavior of rabbits in each group was recorded. Subsequently, cartilage-subchondral bone units were obtained and morphological changes were observed by optical microscope and micro computed tomography. Compression test was used to detect the mechanical properties of subchondral bone, Western blot and real-time polymerase chain reaction (RT-PCR) were applied to detect the expression of bone morphogenetic protein 2-Smad1 (BMP2-Smad1) pathway in subchondral bone.RESULTS: Compared with the control group, rabbits in the KOA group showed lameness, knee pain, and cartilage degradation; the subchondral bone showed active resorption, the mechanical properties decreased significantly and the BMP2-Smad1 pathway downregulated significantly. Both acupotomy and EA intervention could increase the thickness of trabecular bone (Tb. Th), the bone volume fraction (BV/TV) and the thickness of subchondral bone plate, reduce the separation of trabecular bone (Tb. Sp), improve the maximum load and elastic modulus of subchondral bone, and effectively delay cartilage degeneration in KOA rabbits. This process may be achieved through upregulation the related proteins of BMP2-Smad1 pathway. The maximum load and elastic modulus of subchondral bone in the acupotomy group were slightly better than those in the EA group.CONCLUSIONS: Acupotomy could effectively protect cartilage by inhibiting abnormal bone resorption and improving mechanical properties of subchondral bone thorough the related proteins of BMP2-Smad1 pathway in KOA rabbits.& COPY; 2023 JTCM. All rights reserved.

C1 Beijing Univ Chinese Med, Sch Acupuncture Moxibust & Tuina, Beijing 100029, Peoples R China; Capital Med Univ, Beijing Hosp Tradit Chinese Med, Acupuncture & Moxibust Dept, Beijing 100010, Peoples R China; Southern Med Univ, Shenzhen Hosp, Shenzhen 518000, Peoples R China; China Acad Chinese Med Sci, Inst Bone Injury, Beijing 100010, Peoples R China

DI 10.19852/j.cnki.jtcm.20230404.001

ID 25

ER

FN Clarivate Analytics Web of science

PT J

AU Wang, M. A.

AU - Xu, X. B.

AU - Zhao, B. C.

AU - Liu, L.

AU - Zhao, L. P.

AU - Zhang, F.

AU - Ji, X.

AU - Yuan, F.

AU - Xia, Q. Y.

AU - Wang, S. S.

AU - Tian, W.

AU - Wang, L. P.

AU - Li, B.

PY 2023

TI Fire Needling Therapy of Different Frequencies versus External Diclofenac Diethylamine Emulgel for Knee Osteoarthritis: Study Protocol for a Pilot Randomized Controlled Trial

PG 1381-1390

DA 2023/1/1

SO JOURNAL OF PAIN RESEARCH

JO JOURNAL OF PAIN RESEARCH

VL 16

SN 1178-7090

Z9 Times Cited in Web of Science Core Collection: 1 Total Times Cited: 1 Cited Reference Count: 44 ER -

M3 10.2147/JPR.S408084

N1 WangXu-31

N1 2025/2/13 1:16:00

DE knee osteoarthritis; fire needling therapy; different frequencies; dose-effect property; ACUPUNCTURE; OUTCOMES; PAIN; HEALTH; HIP; RELIABILITY

AB Purpose: Knee osteoarthritis (KOA) is regarded as one of the leading musculoskeletal diseases. Although the efficacy is under exploration, fire needling therapy is considered an effective alternative for KOA. This trial aims to investigate the effectiveness of different frequencies of fire needling therapy in attenuating pain and promoting function in KOA patients. Methods: This is a study protocol for a pilot, three-arm, single-center, randomized controlled trial. A total of 90 participants with KOA will be recruited and randomly assigned to the high-frequency fire needling group (3 sessions per week, for 6 weeks), the lowfrequency fire needling group (1 session per week, for 6 weeks) or the positive control group (Diclofenac Diethylamine Emulgel, 3 times per day, for 6 weeks) in a 1:1:1 ratio. Participants will accomplish the trial at Week 14 after a follow-up evaluation. The response rate will be set as the primary outcome that the proportion of participants obtaining a minimal clinically important difference, which is identified as >2 units on the numerical rating scale (NRS) and >6 units on the Western Ontario and McMaster Universities Osteoarthritis Index (WOMAC) function score at Week 6 compared with Week 0. Secondary outcomes are NRS, WOMAC, Brief Pain Inventory, Short-Form Health Survey-12, Timed Up and Go Test, and pain threshold. Discussion: This is the first standardized protocol comparing fire needling therapy and positive control drugs. This trial may provide reliable evidence for the effectiveness of fire needling therapy and dose-effect property of it in KOA. Trial registration: The trial has been registered on Chinese Clinical Trial Registry (Registered number: ChiCTR2100043041), registered on 4 February 2021.

C1 Capital Med Univ, Beijing Hosp Tradit Chinese Med, Dept Acupuncture & Moxibust, Beijing, Peoples R China; Beijing Univ Chinese Med, Grad Sch, Beijing, Peoples R China

DI 10.2147/JPR.S408084

ID 31

ER

FN Clarivate Analytics Web of science

PT J

AU Zhou, J.

AU - Zeng, F.

AU - Cheng, S. R.

AU - Dong, X. H.

AU - Jiang, N. N.

AU - Zhang, X. Y.

AU - Tang, C. J.

AU - He, W. H.

AU - Chen, Y.

AU - Sun, N.

AU - Zhou, Y. F.

AU - Li, X. L.

AU - Hu, S. J.

AU - Sun, R. R.

AU - Wintermark, M.

AU - Yang, W. H.

AU - Liang, F. R.

AU - Li, Z. J.

PY 2023

TI Modulation effects of different treatments on periaqueductal gray resting state functional connectivity in knee osteoarthritis knee pain patients

PG 1965-1980

DA 2023/1/1

SO CNS NEUROSCIENCE & THERAPEUTICS

JO CNS NEUROSCIENCE & THERAPEUTICS

VL 29

IS 7

SN 1755-5930

Z9 Times Cited in Web of Science Core Collection: 9 Total Times Cited: 11 Cited Reference Count: 104 ER -

M3 10.1111/cns.14153

N1 ZhouZeng-38

Y2 2025/1/19 0:16:00

N1 2025/2/13 1:16:00

DE acupuncture; celecoxib; fMRI; knee osteoarthritis; periaqueductal gray; placebo; resting-state functional connectivity; ACUPUNCTURE; DISEASE; NSAIDS; CLASSIFICATION; SENSITIZATION; METAANALYSIS; VALIDATION; MECHANISMS; ARTHRITIS; THERAPY

AB Background: The analgesic effect of acupuncture is widely recognized, but the mechanical characteristics of acupuncture for pain relief, compared to non-steroidal anti-inflammatory (NSAIDs) and placebo medication, remain unknown.Aims: To compare the modulation effects of acupuncture treatment with NSAIDs and placebo medication on descending pain modulation system (DPMS) in knee osteoarthritis (KOA) patients.Methods: This study recruited 180 KOA patients with knee pain and 41 healthy controls (HCs). Individuals with KOA knee pain were divided randomly into groups of verum acupuncture (VA), sham acupuncture (SA), celecoxib (SC), placebo (PB), and waiting list (WT), with 36 patients in each group. VA and SA groups included ten sessions of puncturing acupoints or puncturing non-acupoints acupuncture treatment for two successive weeks. Celecoxib capsules were continuously given orally to patients in the SC group at a dosage of 200 mg daily for 2 weeks. In the PB group, patients received a placebo capsule once a day for 2 weeks at the same dosage as celecoxib capsules. In the WL group, patients did not receive any treatment. Patients underwent a resting-state BOLD-fMRI scan pre- and post-receiving the therapy, whereas HCs only underwent a baseline scan. Seed (ventrolateral periaqueductal gray, vlPAG, a key node in DPMS) based resting-state functional connectivity (rs-FC) was applied in the data analysis.Results: All groups demonstrated improved knee pain scores relative to the initial state. There was no statistical difference between the VA and SA groups in all clinical outcomes, and vlPAG rs-FC alterations. KOA knee pain individuals reported higher vlPAG rs-FC in the bilateral thalamus than HCs. KOA knee pain patients in the acupuncture group (verum + sham, AG) exhibited increased vlPAG rs-FC with the right dorsolateral prefrontal cortex (DLPFC) and the right angular, which is associated with knee pain improvement. In contrast with the SC and PB group, the AG exhibited significantly increased vlPAG rs-FC with the right DLPFC and angular. Contrary to the WT group, the AG showed greater vlPAG rs-FC with the right DLPFC and precuneus.Conclusions: Acupuncture treatment, celecoxib, and placebo medication have different modulation effects on vlPAG DPMS in KOA knee pain patients. Acupuncture could modulate vlPAG rs-FC with brain regions associated with cognitive control, attention, and reappraisal for knee pain relief in KOA patients, compared with celecoxib and placebo medication.

C1 Chengdu Univ Tradit Chinese Med, Chengdu, Peoples R China; Chengdu Univ Tradit Chinese Med, Acupuncture & Brain Res Ctr, Chengdu, Peoples R China; Hosp Chengdu Univ Tradit Chinese Med, Chengdu, Peoples R China; Univ Tradit Chinese Med, Affiliated Hosp Shanxi 2, Taiyuan, Peoples R China; Sichuan Univ, West China Hosp, Rehabil Med Ctr, Chengdu, Peoples R China; Sichuan Univ, West China Hosp, Inst Rehabil Med, Chengdu, Peoples R China; Key Lab Rehabil Med Sichuan Prov, Chengdu, Peoples R China; Stanford Univ, Radiol Dept, Stanford, CA USA; Dali Bai Autonomous Prefecture Chinese Med Hosp, Dali, Peoples R China

DI 10.1111/cns.14153

ID 38

ER

FN Clarivate Analytics Web of science

PT J

AU Lee, C. J.

AU - Luo, W. T.

AU - Tam, K. W.

AU - Huang, T. W.

PY 2023

TI Comparison of the effects of acupotomy and acupuncture on knee osteoarthritis: A systematic review and meta-analysis

DA 2023/1/1

SO COMPLEMENTARY THERAPIES IN CLINICAL PRACTICE

JO COMPLEMENTARY THERAPIES IN CLINICAL PRACTICE

VL 50

SN 1744-3881

Z9 Times Cited in Web of Science Core Collection: 5 Total Times Cited: 5 Cited Reference Count: 87 ER -

M3 10.1016/j.ctcp.2022.101712

N1 LeeLuo-47

Y2 2025/1/17 15:45:00

N1 2025/2/13 1:16:00

DE Acupuncture; Acuputomy; Cure rate; Meta-analysis; Osteoarthritis; Systematic review; SAFETY; MANAGEMENT; DIAGNOSIS; EFFICACY; INDEX

AB Background and purpose: Acupotomy and acupuncture are both treatments for knee osteoarthritis symptoms. However, acupotomy also has the additional anatomical effect of dissecting inflamed tissue. The problem this study aims to address is whether acupotomy is a better treatment than acupuncture in treating knee osteoarthritis. Methods: We searched the PubMed, Embase, Cochrane Library, China National Knowledge Infrastructure, Airiti Library, and Wanfang Data databases from inception to March 2022 for randomized controlled clinical trials (RCTs) comparing the effects of acupotomy and acupuncture in patients with knee osteoarthritis. Results: In total, we identified 43 RCTs in this meta-analysis. Compared to the acupuncture group, acupotomy had a higher cure rate (odds ratio (OR) 2.94, 95% confidence interval (CI) 2.36 to 3.65), indicating a better improvement in daily activity function. Acupotomy was also more effective in pain relief and knee score improvement. However, some RCTs indicated that adverse events in the acupotomy group were greater than in the acupuncture group (OR 1.23, 95% CI 0.42 to 3.60). Conclusion: Our findings indicated that acupotomy was a more effective treatment for knee osteoarthritis than acupuncture. However, most of the included RCTs had moderate risk of bias, meaning that more high-quality RCTs were needed.

C1 Taipei Med Univ, Coll Med, Sch Med, Taipei, Taiwan; Taipei Med Univ, Shuang Ho Hosp, Ctr Evidence Based Hlth Care, New Taipei, Taiwan; Taipei Med Univ, Shuang Ho Hosp, Dept Med Res, New Taipei, Taiwan; Taipei Med Univ, Coll Med, Sch Med, Dept Surg, New Taipei, Taiwan; Taipei Med Univ, Shuang Ho Hosp, Dept Surg, New Taipei, Taiwan; Taipei Med Univ, Cochrane Taiwan, Taipei, Taiwan; Taipei Med Univ, Coll Nursing, Sch Nursing, 250 Wuxing St, Taipei 11031, Taiwan; Taipei Med Univ, Wan Fang Hosp, Ctr Nursing & Healthcare Res Clin Practice Applica, Taipei, Taiwan; Taipei Med Univ, Wan Fang Hosp, Dept Nursing, Taipei, Taiwan

DI 10.1016/j.ctcp.2022.101712

ID 47

ER

FN Clarivate Analytics Web of science

PT J

AU Ma, W. W.

AU - Zhang, C. Y.

AU - Huang, X.

AU - Cheng, W.

PY 2023

TI Network meta-analysis of 7 acupuncture therapies for knee osteoarthritis

DA 2023/1/1

SO MEDICINE

JO MEDICINE

VL 102

IS 43

SN 0025-7974

Z9 Times Cited in Web of Science Core Collection: 1 Total Times Cited: 1 Cited Reference Count: 49 ER -

M3 10.1097/MD.0000000000035670

N1 MaZhang-49

N1 2025/2/13 1:16:00

DE acupuncture; knee osteoarthritis; network meta-analysis; randomized controlled study; systematic review; HAND; HIP

AB Objective: With the progression of society aging demographic, the prevalence of knee osteoarthritis (KOA) continues to rise steadily, exerting a significant impact on individuals' quality of life. Acupuncture therapy has garnered extensive utilization in the management of osteoarthritis; however, a comprehensive systematic review integrating acupuncture with traditional Chinese medicine remains absent. This study compared the clinical efficacy of 7 acupuncture methods (electroacupuncture, conventional acupuncture, warm needle, floating needle, fire needle, needle knife, and silver needle) for the treatment of KOA through a network meta-analysis.Methods: This study examined the databases-PubMed, EMbase, The Cochrane Library, the China Biology Medicine, Chinese Journal Full-text Database, Wanfang Database, and VIP Database-for randomized controlled trials of the 7 methods for KOA treatment. The search time spanned from the database establishment to March 5, 2022. The primary outcome indicator was the total effective rate, and the secondary outcome indicator was the visual analog scale. After the layer-by-layer screening, the quality of the literature was assessed using the Cochrane systematic reviewer manual 5.1.0 bias risk assessment tool for randomized controlled trials. After data extraction, the R4.0.1 software was used for network meta-analysis.Results: Based on the network meta-analysis, the ranking of interventions based on the surface under the cumulative ranking curve for the total effective rate is as follows: silver needle (0.99) > floating needle (0.97) > needle knife (0.66) > fire needle (0.56) > warm needle (0.44) > conventional acupuncture (0.35) > electroacupuncture (0.13). Regarding the improvement in visual analog scale scores, the surface under the cumulative ranking curve ranking is as follows: silver needle (0.97) > conventional acupuncture (0.67) > needle knife (0.64) > floating needle (0.51) > warm needle (0.44) > fire needle (0.14) > electroacupuncture (0.09).Conclusion: Based on the network meta-analysis, silver needle therapy emerged as the most efficacious and analgesic intervention for KOA. Nevertheless, given the notable variations in the quality and quantity of studies encompassing diverse treatment modalities, the findings of this research necessitate further substantiation through forthcoming high-quality multicenter, large-sample, randomized double-blind trials.

C1 Hubei Univ Chinese Med, Sch Acupuncture Moxibust & Orthoped, Wuhan, Peoples R China; Wuhan Hosp Tradit Chinese Med, Wuhan, Peoples R China; Hubei Univ Chinese Med, Sch Acupuncture Moxibust & Orthoped, Wuhan 430061, Peoples R China

DI 10.1097/MD.0000000000035670

ID 49

ER

FN Clarivate Analytics Web of science

PT J

AU Lee, B. R.

AU - Kim, T. H.

AU - Birch, S.

AU - Alraek, T.

AU - Lee, H. W.

AU - Nielsen, A.

AU - Wieland, L. S.

AU - Lee, M. S.

PY 2023

TI Comparative effectiveness of acupuncture in sham-controlled trials for knee osteoarthritis: A systematic review and network meta-analysis

DA 2023/1/1

SO FRONTIERS IN MEDICINE

JO FRONTIERS IN MEDICINE

VL 9

SN 2296-858X

Z9 Times Cited in Web of Science Core Collection: 8 Total Times Cited: 8 Cited Reference Count: 37 ER -

M3 10.3389/fmed.2022.1061878

N1 LeeKim-52

N1 2025/2/13 1:16:00

DE acupuncture; knee osteoarthritis; systematic review; network meta-analysis; comparative effectiveness; acupuncture therapy; placebo; RANDOMIZED CONTROLLED-TRIAL; MANAGEMENT; EXERCISE; PAIN; HIP; PHYSIOTHERAPY; DEVICE

AB Objectives Although many trials have assessed the effect of acupuncture on knee osteoarthritis (KOA), its efficacy remains controversial. Sham acupuncture techniques are regarded as representative control interventions in acupuncture trials and sometimes incorporate the use of sham devices (base units) to support a non-penetrating needle. To achieve successful blinding, these trials also use acupuncture base units in the verum acupuncture group. Base units are not used in real-world clinical settings. We aimed to assess the effect sizes of verum and sham acupuncture for KOA in sham-controlled trials with or without base units.Methods A total of 10 electronic databases for randomized controlled trials (RCTs) comparing the efficacy of verum manual acupuncture and sham acupuncture for the treatment of KOA were searched for articles published before April 12, 2022. The primary outcome was pain intensity, and the secondary outcomes included physical function. The first assessment after the end of treatment was chosen for analysis. Effect sizes are reported as standardized mean differences (SMDs) with 95% confidence intervals (95% CIs). The risk of bias was assessed using the Cochrane risk of bias tool, and publication bias was evaluated using a funnel plot and Egger's test. The quality of evidence for estimates was evaluated using the Grading of Recommendations, Assessment, Development, and Evaluations (GRADE) approach.Results Fifteen RCTs were included. There was generally a low risk of bias except for the difficulty in blinding acupuncture therapists (performance bias). Compared to verum acupuncture in sham-controlled trials using base units, verum acupuncture in sham-controlled trials without base units was more effective for improving pain (SMD -0.56, 95% CI -1.09 to -0.03) and function (SMD -0.73, 95% CI -1.36 to -0.10) in KOA. The quality of evidence for network estimates was moderate to low due to the risk of bias and imprecision.Conclusion These findings suggest that verum acupuncture in different types of sham-controlled trials has different effect sizes for KOA. Because base units are not used in clinical settings, the results of verum acupuncture in sham-controlled trials with base units need to be interpreted carefully.

C1 Korea Inst Oriental Med, KM Sci Res Div, Daejeon, South Korea; Kyung Hee Univ, Korean Med Hosp, Korean Med Clin Trial Ctr, Seoul, South Korea; Kristiania Univ Coll, Sch Hlth Sci, Oslo, Norway; Natl Res Ctr Complementary & Alternat Med, Fac Hlth Sci, Dept Community Med, Tromso, Norway; Korea Inst Oriental Med, KM Convergence Res Div, Daejeon, South Korea; Icahn Sch Med Mt Sinai, Dept Family Med & Community Hlth, New York, NY USA; Univ Maryland, Ctr Integrat Med, Sch Med, Baltimore, MD USA

DI 10.3389/fmed.2022.1061878

ID 52

ER

FN Clarivate Analytics Web of science

PT J

AU Zheng, A. J.

AU - Zheng, F. F.

AU - Jin, K.

AU - Chi, M.

AU - Mu, J. J.

AU - Wei, H. X.

AU - Lei, J.

AU - Xu, C.

PY 2023

TI Manual Acupuncture at LI11, Local Points and Both for Knee Osteoarthritis: A Pilot Randomized Controlled Trial

PG 4393-4404

DA 2023/1/1

SO JOURNAL OF PAIN RESEARCH

JO JOURNAL OF PAIN RESEARCH

VL 16

SN 1178-7090

Z9 Times Cited in Web of Science Core Collection: 1 Total Times Cited: 1 Cited Reference Count: 19 ER -

M3 10.2147/JPR.S445770

N1 ZhengZheng-55

N1 2025/2/13 1:16:00

DE knee osteoarthritis; manual acupuncture; Quchi; LI11; pilot randomized controlled trial; HIP

AB Objective: Knee osteoarthritis (KOA) is a common chronic degenerative joint disease, and acupuncture is an alternative therapy for KOA. This study aims to detect the effectiveness of acupuncture at LI11 in improving pain and function for KOA patients.Methods: A total of 108 patients with KOA were randomly allocated to Control Group (local points), Treatment Group A (LI11), and Treatment Group B (local points and LI11) with a treatment phase of 4 weeks and a follow-up phase of 4 weeks. Primary outcome was response rate. Secondary outcomes included Visual Analogue Scale (VAS), Western Ontario and McMaster Universities Osteoarthritis Index (WOMAC), and recurrence rate. Study was registered on Chinese Clinical Trial Registry (Registered number: ChiCTR2000034926).Results: The response rate in Treatment Group A, Treatment Group B, and Control Group was 71.43%, 85.29%, and 51.53%, respectively, at Week 4, and Treatment Group B was significantly higher than Control Group (difference[98.3% CI]: 33.86 [0.135,0.543], P = 0.003). Although no significant difference was found, Treatment Group A had a better response rate compared with Control Group (difference[98.3% CI]: 20.00 [-0.072, 0.472], P = 0.086). For VAS and WOMAC, there were significant differences within 3 groups at Week 4 compared with the baseline. There was a significant improvement in VAS scores and WOMAC function and pain subscales at Week 4 in Treatment Group B compared with Control Group and Treatment Group A.Conclusion: LI11 is an effective point for patients with KOA, and it could be a selection for young acupuncturists and acupuncturists who work in rural areas; however, large-sample studies are necessary to further verify results in the future.

C1 Peking Univ Third Hosp, Dept Acupuncture & Moxibust, Yanqing Hosp, Beijing 102100, Peoples R China; Nancaiyuan Community Hosp, Dept Tradit Chinese Med, Beijing 102100, Peoples R China; Peking Univ Third Hosp, Yanqing Hosp, Dept Pharm, Beijing 102100, Peoples R China

DI 10.2147/JPR.S445770

ID 55

ER

FN Clarivate Analytics Web of science

PT J

AU Wu, Q. L.

AU - Wu, Z. Q.

AU - Lu, Z. F.

PY 2023

TI Efficacy of acupotomy combined with sodium hyaluronate versus sodium hyaluronate alone in the treatment of knee osteoarthritis: A meta-analysis

DA 2023/1/1

SO MEDICINE

JO MEDICINE

VL 102

IS 37

SN 0025-7974

Z9 Times Cited in Web of Science Core Collection: 0 Total Times Cited: 0 Cited Reference Count: 32 ER -

M3 10.1097/MD.0000000000034930

N1 WuWu-63

N1 2025/2/13 1:16:00

DE acupotomy; knee osteoarthritis; meta-analysis; sodium hyaluronate; EPIDEMIOLOGY

AB Background: The efficacy of acupotomy combined with hyaluronic sodium acid in the treatment of knee osteoarthritis (KOA) is unclear. Therefore, this meta-analysis aims to evaluate the efficacy of acupotomy combined with hyaluronic sodium acid compared with hyaluronic sodium acid alone in the treatment of KOA.Methods: Studies from 8 Online databases were searched on KOA treatment using acupotomy combined with sodium hyaluronate until May 2022. The primary outcome indicator was clinical effectiveness, and the secondary outcome indicators included the visual analogue scale scores and Lysholm scores. We calculated the weighted mean difference (WMD) or relative risk for all relevant outcomes.Results: Nine studies were identified, involving 644 cases. The results showed that acupotomy combined with intra-articular sodium hyaluronate injection for KOA was superior to sodium hyaluronate injection alone in terms of clinical effectiveness (relative risk = 1.17, 95% confidence interval [CI]: 1.09-1.25, P < .001) and visual analogue scale (WMD = -2.1, 95% CI: -2.25 to 1.95, P < .001), Lysholm score (WMD = 13.83, 95% CI: 3.47-24.19, P = .009).Conclusion: Acupotomy combined with intra-articular sodium hyaluronate injection for KOA is superior to sodium hyaluronate injection alone. Limited by the number and quality of included studies, this conclusion still needs to be verified by more high-quality Research.INPLASY registration number: INPLASY202350029.

C1 Haikou Hosp Tradit Chinese Med, Dept Massage, Haikou, Peoples R China; Haikou Hosp Tradit Chinese Med, Dept Orthoped & Traumatol, 2 Poxiang Rd, Haikou 570216, Hainan, Peoples R China

DI 10.1097/MD.0000000000034930

ID 63

ER

FN Clarivate Analytics Web of science

PT J

AU Zhang, W.

AU - Zhang, L. L.

AU - Yang, S.

AU - Wen, B.

AU - Chen, J.

AU - Chang, J.

PY 2023

TI Electroacupuncture ameliorates knee osteoarthritis in rats via inhibiting NLRP3 inflammasome and reducing pyroptosis

DA 2023/1/1

SO MOLECULAR PAIN

JO MOLECULAR PAIN

VL 19

SN 1744-8069

Z9 Times Cited in Web of Science Core Collection: 5 Total Times Cited: 5 Cited Reference Count: 49 ER -

M3 10.1177/17448069221147792

N1 ZhangZhang-69

Y2 2025/1/19 22:10:00

N1 2025/2/13 1:16:00

DE Electroacupuncture; knee osteoarthritis; NLRP3 inflammasome; pyroptosis; ACUPUNCTURE

AB Objective: Knee Osteoarthritis (KOA), is the most common joint disease worldwide. The pathogenesis of KOA is complex and electroacupuncture (EA) is an effective therapy for KOA, but the mechanism remains unclear. In this study, we aim to investigate the potential therapeutic effect of EA on the rat model of KOA induced by monosodium iodoacetate (MIA) and its relationship with NLRP3 inflammasome by immunohistochemistry and western blot. Methods: KOA was induced by intra-articular injection of MIA (3 mg/50 mu L) into the right knee joint of rats. Forty-five male rats weighing 250-300 g were randomly divided into 3 groups: control group, KOA group, and KOA + electroacupuncture group (KOA+EA). EA treatment lasted for 2 weeks (6 times a week). Paw withdrawal threshold tests were used to assess mechanical allodynia once a week. Safranin O/Fast Green and hematoxylin and eosin (H&E) staining were used to assess the damage to cartilage, synovium, and subpatellar fat pad (IFP). Immunohistochemistry was used to observe NLRP3 inflammasome-associated protein-positive cells in the same field of view and western blot was used to detect the expression of the associated protein in cartilage tissue. Results: The KOA group showed mechanical hyperalgesia, joint inflammation, and significant cartilage tissue destruction. Safranin O/Fast Green and H&E staining revealed that EA alleviated the joint pathological changes caused by KOA and had a protective effect on cartilage, synovium, and IFP destruction. Mechanical allodynia pain and joint swelling were reduced in KOA rats after EA treatment. Immunohistochemistry and western blot showed significant inhibition of NLRP3 inflammasome-associated protein. Conclusion: The results indicate that EA can inhibit NLRP3 inflammasome and reduce pyroptosis, which results in the protection of cartilage tissue and the treatment of KOA. It provides reliable evidence for the development of EA in the treatment of KOA and the clinical application of acupuncture.

C1 Anhui Med Univ, Sch Basic Med Sci, Hefei, Peoples R China; Anhui Med Univ, Affiliated Hosp 1, Anhui Publ Hlth Clin Ctr, Hefei, Peoples R China; Huazhong Univ Sci & Technol, Tongji Med Coll, Sch Basic Med, Dept Biochem & Mol Biol, Wuhan, Hubei, Peoples R China; Huazhong Univ Sci & Technol, Tongji Med Coll, Collaborat Innovat Ctr Brain Sci, Wuhan, Hubei, Peoples R China; Anhui Med Univ, Sch Life Sci, Inflammat & Immune Mediated Dis Lab Anhui Prov, Hefei, Peoples R China; Huazhong Univ Sci & Technol, Tongji Med Coll, Sch Basic Med, Dept Biochem & Mol Biol, Wuhan 430030, Hubei, Peoples R China; Huazhong Univ Sci & Technol, Tongji Med Coll, Collaborat Innovat Ctr Brain Sci, Wuhan 430030, Hubei, Peoples R China; Anhui Med Univ, Affiliated Hosp 1, Anhui Publ Hlth Clin Ctr, Hefei 230000, Peoples R China

DI 10.1177/17448069221147792

ID 69

ER

FN Clarivate Analytics Web of science

PT J

AU Li, Y. Y.

AU - Hou, Y. M.

AU - Sun, J. W.

AU - Wei, J. B.

AU - Chai, Y. M.

AU - Guo, M. W.

AU - Wang, R. G.

PY 2023

TI Therapeutic Effect of Acupotomy at Sanheyang for Cartilage Collagen Damage in Moderate Knee Osteoarthritis: A Rabbit Model

PG 2241-2254

DA 2023/1/1

SO JOURNAL OF INFLAMMATION RESEARCH

JO JOURNAL OF INFLAMMATION RESEARCH

VL 16

SN 1178-7031

Z9 Times Cited in Web of Science Core Collection: 1 Total Times Cited: 1 Cited Reference Count: 40 ER -

M3 10.2147/JIR.S400956

N1 LiHou-70

N1 2025/2/13 1:16:00

DE acupotomy; cartilage; gastrocnemius; knee osteoarthritis; sinew; ARTICULAR CHONDROCYTES; EXPRESSION; BETA-1-INTEGRINS; BUPRENORPHINE; ACTIVATION; ANALGESIA; ADHESION; SAFETY

AB Objective: Acupotomy based on the meridian-sinew theory of traditional Chinese medicine has benefits in treating knee osteoarthritis (KOA). The current study aims to prove that acupotomy at the sinew points of Sanheyang protect the knee joint and alleviate the progression of moderate KOA by evaluating KOA symptoms, cartilage structure, and analyzing the changes of cytokines in rabbit cartilage.Methods: The model used was mono-iodoacetate-induced moderate KOA in the rabbit's right leg. Rabbits were divided into the model group, the acupotomy group, and the control group, with each group receiving two parts of treatment for 2 weeks and 4 weeks. We evaluated pain in the knee joint and range of motion. The articular cartilage sections were stained with Safranin O/Fast Green and Masson. We used immunohistochemistry and real-time PCR to detect the protein and mRNA expressions of collagen prototype II (COL-II), matrix metalloproteinase 13 (MMP13), and integrin-131 (ITG-131). Results: Compared with the model group, the acupotomy group had higher body weight, lower pain score, higher range of motion, lower Mankin score, and significantly lower protein and mRNA expression of MMP13. After 4 weeks of treatment, Col-II expression in the acupotomy group was significantly higher than that in the model group and the expression of ITG-131 in the model group was abnormally increased.Conclusion: Acupotomy at Sanheyang improved the pain symptoms and range of joint motion in rabbits with moderate KOA, and could protect Col-II by regulating MMP13, which may be related to ITG-131-mediated mechanical force transmission, thus reducing the damage to cartilage structure and delaying the progression of moderate KOA.

C1 Beijing Univ Chinese Med, Sch Acupuncture Moxibust & Tuina, Beijing 100029, Peoples R China; Beijing Univ Chinese Med, Sch Acupuncture Moxibust & Tuina, 11, Bei San Huan Dong Lu, Beijing 100029, Peoples R China

DI 10.2147/JIR.S400956

ID 70

ER

FN Clarivate Analytics Web of science

PT J

AU Mi, B. H.

AU - Wang, X. Z.

AU - Yang, J. W.

AU - Shi, G. X.

AU - Zhang, W. Z.

AU - Jin, L. N.

AU - Yang, L. S.

AU - Liu, D. H.

AU - Kang, S. B.

AU - Zhou, H.

AU - Wang, Y. R.

AU - Wang, L. Q.

AU - Tu, J. F.

PY 2023

TI Thermographic evaluation of acupoints in lower limb region of individuals with osteoarthritis: A cross-sectional case-control study protocol

DA 2023/1/1

SO PLOS ONE

JO PLOS ONE

VL 18

IS 4

SN 1932-6203

Z9 Times Cited in Web of Science Core Collection: 1 Total Times Cited: 1 Cited Reference Count: 38 ER -

M3 10.1371/journal.pone.0284381

N1 MiWang-77

Y2 2025/1/20 13:41:00

N1 2025/2/13 1:16:00

DE ACUPUNCTURE TREATMENT; SKIN TEMPERATURE; KNEE; GUIDELINES; MANAGEMENT; STATEMENT; CONSENSUS; DISEASE; HAND; PAIN

AB PurposeAcupuncture has been widely used in the treatment of knee osteoarthritis (KOA), but the selection of acupoints is indeterminate and lacks biological basis. The skin temperature of acupoints can reflect the state of local tissue and may be a potential factor for guiding acupoint selection. This study aims to compare the skin temperature of acupoints between KOA patients and the healthy population.Study design and methodsThis is a protocol for a cross-sectional case-control study with 170 KOA patients and 170 age- and gender-matched healthy individuals. Diagnosed patients aged 45 to 70 will be recruited in the KOA group. Participants in the healthy group will be matched with the KOA group based on mean age and gender distribution. Skin temperature of 11 acupoints (ST35, EX-LE5, GB33, GB34, EX-LE2, ST34, ST36, GB39, BL40, SP9, SP10) will be extracted from infrared thermography (IRT) images of the lower limbs. Other measurements will include demographic data (gender, age, ethnicity, education, height, weight, BMI) and disease-related data (numerical rating scale, pain sites, duration of pain, pain descriptors, pain activities).DiscussionThe results of this study will provide biological evidence for acupoint selection. This study is a precondition for follow-up studies, in which the value of optimized acupoint selection will be verified.

C1 Beijing Univ Chinese Med, Int Acupuncture & Moxibust Innovat Inst, Sch Acupuncture Moxibust & Tuina, Beijing, Peoples R China; Beijing Univ Chinese Med, Dongzhimen Hosp, Beijing, Peoples R China; Jiaodong Community Hlth Serv Stn, Beijing, Peoples R China; Xiaoguan East St Community Hlth Serv Stn, Beijing, Peoples R China; Deluyuan Community Hlth Serv Stn, Beijing, Peoples R China

DI 10.1371/journal.pone.0284381

ID 77

ER

FN Clarivate Analytics Web of science

PT J

AU Xing, L. F.

AU - Guo, Y.

AU - Chen, X. L.

AU - Hu, T. Y.

AU - Zhu, W. T.

AU - Ma, W. W.

AU - Du, M.

AU - Xu, Y.

AU - Guo, C. Q.

PY 2023

TI Application of Acupotomy in a Knee Osteoarthritis Model in Rabbit

DA 2023/1/1

SO JOVE-JOURNAL OF VISUALIZED EXPERIMENTS

JO JOVE-JOURNAL OF VISUALIZED EXPERIMENTS

IS 200

SN 1940-087X

Z9 Times Cited in Web of Science Core Collection: 1 Total Times Cited: 1 Cited Reference Count: 20 ER -

M3 10.3791/65584

N1 XingGuo-98

N1 2025/2/13 1:16:00

DE MUSCLE STRENGTH; WOMEN

AB Knee osteoarthritis (KOA) is one of the most frequently encountered diseases in the orthopedic department, which seriously reduces the quality of life of people with KOA. Among several pathogenic factors, the biomechanical imbalance of the knee joint is one of the main causes of KOA. Acupotomology believes that restoring the mechanical balance of the knee joint is the key to treating KOA. Clinical studies have shown that acupotomy can effectively reduce pain and improve knee mobility by reducing adhesion, contracture of soft tissues, and stress concentration points in muscles and tendons around the knee joint.In this protocol, we used the modified Videman method to establish a KOA model by immobilizing the left hindlimb in a straight position. We have outlined the method of operation and the precautions related to acupotomy in detail and evaluated the efficacy of acupotomy in conjunction with the theory of "Modulating Muscles and Tendons to Treat Bone Disorders" through the detection of the mechanical properties of quadriceps femoris and tendon, as well as cartilage mechanics and morphology. The results show that acupotomy has a protective effect on cartilage by adjusting the mechanical properties of the soft tissues around the knee joint, improving the cartilage stress environment, and delaying cartilage degeneration.

C1 Beijing Univ Chinese Med, Sch Acupuncture Moxibust & Tuina, Beijing, Peoples R China; Capital Med Univ, Beijing Hosp Tradit Chinese Med, Acupuncture & Moxibust Dept, Beijing, Peoples R China; Beijing Univ Chinese Med, Affiliated Hosp 3, Beijing, Peoples R China

DI 10.3791/65584

ID 98

ER

FN Clarivate Analytics Web of science

PT J

AU Lin, S. X.

AU - Lai, C. S.

AU - Wang, J. X.

AU - Lin, Y. T.

AU - Tu, Y. Y.

AU - Yang, Y. F.

AU - Zhang, R. P.

PY 2023

TI Efficacy of ultrasound-guided acupotomy for knee osteoarthritis: A systematic review and meta-analysis of randomized controlled trials

DA 2023/1/1

SO MEDICINE

JO MEDICINE

VL 102

IS 2

SN 0025-7974

Z9 Times Cited in Web of Science Core Collection: 4 Total Times Cited: 4 Cited Reference Count: 34 ER -

M3 10.1097/MD.0000000000032663

N1 LinLai-104

Y2 2025/1/17 14:44:00

N1 2025/2/13 1:16:00

DE knee osteoarthritis; meta-analysis; systematic review; ultrasound-guided acupotomy; SAFETY

AB Background:This systematic review aimed to evaluate the effectiveness and safety of ultrasound-guided acupotomy (UGAT) therapy in the treatment of patients with knee osteoarthritis (KOA). Methods:We conducted online researches in the databases including PubMed, the Cochrane Library, EMBASE, China national knowledge infrastructure, China biomedical literature database, and Wan Fang data. All data were collected until January 1, 2022. Relevant randomized controlled trials on the effectiveness of UGAT for the treatment of KOA were included. Meta-analyses were carried out by RevMan 5.3 software. Evidence quality was evaluated by the grading of recommendations, assessment development, and evaluation. Results:Eight studies including 543 participants were analyzed in this study. The pooled analysis indicated that UGAT was significantly more efficient than the control group in decreasing the visual analogue scale score (mean difference = -0.81, 95% confidence interval (CI) = [-1.15, -0.47], P < .00001, 8 studies), improving knee function on the Lysholm knee score (mean difference = 8.26, 95% CI = [1.56, 14.97], P = .02, 2 studies), and increasing clinical effective rate (relative risk = 1.14, 95% CI = [1.06, 1.23], P = .0005, 6 studies). For adverse events, UGAT was also associated with lower incidence of adverse event (odds ratio = 0.27, 95% CI = [0.12, 0.63], P = .002, 4 studies) compared to traditional acupotomy. Conclusion:Current evidence suggested that UGAT therapy was effective and safe in the clinical treatments of KOA, thus could be suggested in the clinical managements of KOA. However, considering the unsatisfactory quality of the available trials, more large-scale, and better quality randomized controlled trials were recommend in future.

C1 Fujian Univ Tradit Chinese Med, Quanzhou Osteopath Hosp, Quanzhou, Fujian, Peoples R China; Fujian Univ Tradit Chinese Med, Quanzhou Osteopath Hosp, 61 South Sect Citong West Rd, Quanzhou 362000, Fujian, Peoples R China

DI 10.1097/MD.0000000000032663

ID 104

ER

FN Clarivate Analytics Web of science

PT J

AU Yin, S.

AU - Chang, Y. N.

AU - Yan, X. L.

AU - Feng, X. D.

AU - Wu, N.

PY 2023

TI Effect of acupuncture for patients with knee osteoarthritis: study protocol for a double-dummy randomized controlled trial

DA 2023/1/1

SO JOURNAL OF ORTHOPAEDIC SURGERY AND RESEARCH

JO JOURNAL OF ORTHOPAEDIC SURGERY AND RESEARCH

VL 18

IS 1

SN 1749-799X

Z9 Times Cited in Web of Science Core Collection: 0 Total Times Cited: 0 Cited Reference Count: 47 ER -

M3 10.1186/s13018-023-04198-2

N1 YinChang-106

N1 2025/2/13 1:16:00

DE Knee osteoarthritis; Acupuncture; Pain; Randomized controlled trial; DOUBLE-BLIND; EFFICACY; PAIN; POPULATION; THERAPY; SAFETY; IMPACT

AB BackgroundAcupuncture has been used to relieve chronic pain in patients with knee osteoarthritis (KOA), but the evidence is contradictory. Therefore, we carefully designed a double-dummy randomized controlled trial (RCT) to explore the therapeutic effect of acupuncture for KOA.Methods/designA total of 138 eligible participants with KOA who consent to participate will be randomly divided into Groups A, B, and C in a ratio of 1:1:1. Participants in Group A will receive verum acupuncture and placebo gel, while those in Groups B and C will be treated with diclofenac diethylammon gel and sham acupuncture, sham acupuncture and placebo gel, respectively. The patients will receive 4 weeks of treatment, five times a week, including acupuncture treatment once a day for 30 min and gel treatment three times a day. The primary outcome will be the change of Western Ontario and McMaster Universities Osteoarthritis Index (WOMAC) at week 4. The secondary outcomes will include visual analog scale (VAS), Arthritis Quality of Life Measurement Scale Simplified Scale (AIMS2-SF), Beck Anxiety Inventory (BAI), Beck Depression Inventory (BDI) and Credibility/Expectancy Questionnaire. The evaluation will be performed at baseline, week 4, 8, and 12 after randomization.DiscussionThis double-dummy RCT used diclofenac diethylammon gel as a positive control, and the completion of this trial will provide detailed and accurate evidence of the efficacy and safety of acupuncture for KOA.Trial registration: China Clinical Trials Registry No.ChiCTR2100043947. Registered on September 24, 2020. https://www.chictr.org.cn/showproj.html?proj=122536.DiscussionThis double-dummy RCT used diclofenac diethylammon gel as a positive control, and the completion of this trial will provide detailed and accurate evidence of the efficacy and safety of acupuncture for KOA.Trial registration: China Clinical Trials Registry No.ChiCTR2100043947. Registered on September 24, 2020. https://www.chictr.org.cn/showproj.html?proj=122536.

C1 Henan Univ Chinese Med, Affiliated Hosp 1, Rehabil Ctr, Zhengzhou, Henan, Peoples R China; Zhengzhou Railway Vocat & Tech Coll, Sch Med Technol & Engn, Zhengzhou, Henan, Peoples R China; Henan Univ Chinese Med, Sch Rehabil Med, Zhengzhou, Henan, Peoples R China

DI 10.1186/s13018-023-04198-2

ID 106

ER

FN Clarivate Analytics Web of science

PT J

AU Guo, Y.

AU - Xu, Y.

AU - He, M.

AU - Chen, X. L.

AU - Xing, L. F.

AU - Hu, T. Y.

AU - Zhang, Y.

AU - Du, M.

AU - Zhang, D.

AU - Zhang, Q.

AU - Li, B.

PY 2023

TI Acupotomy Improves Synovial Hypoxia, Synovitis and Angiogenesis in KOA Rabbits

PG 749-760

DA 2023/1/1

SO JOURNAL OF PAIN RESEARCH

JO JOURNAL OF PAIN RESEARCH

VL 16

SN 1178-7090

Z9 Times Cited in Web of Science Core Collection: 1 Total Times Cited: 2 Cited Reference Count: 59 ER -

M3 10.2147/JPR.S396955

N1 GuoXu-108

N1 2025/2/13 1:16:00

DE acupotomy; KOA; synovium; intra-articular pressure; hypoxia; angiogenesis; COLLAGEN-INDUCED ARTHRITIS; BOLD-MRI; INDUCIBLE FACTOR-1-ALPHA; INFLAMMATORY ARTHRITIS; OSTEOARTHRITIS; OXYGEN; CARTILAGE; MODEL; EXPRESSION; VALIDITY

AB Purpose: Knee osteoarthritis (KOA) is a chronic inflammatory disease highly associated with intra-articular hypertension, hypoxia and angiogenesis of synovial tissue. Our previous studies showed that acupotomy could treat KOA in a variety of ways, including reducing cartilage deterioration and enhancing biomechanical qualities. However, the mechanism of hypoxia and angiogenesis induced by acupotomy in KOA synovium remains unclear. This study looked for the benign intervention of acupotomy in synovial pathology. Methods: The rabbits were divided into 3 groups, Normal group, KOA group, and KOA + Acupotomy (Apo) group, with 11 rabbits in each group. The KOA rabbit model was established by the modified Videman method with six weeks. The KOA + Apo group performed the intervention. The tendon insertion of vastus medialis, vastus lateralis, rectus femoris, biceps femoris, and anserine bursa were selected as treatment points in rabbits. Rabbits were treated once every 7 days for 3 weeks. We observed the intra-articular pressure and oxygen partial pressure (BOLD MRI). The synovial morphology was monitored by Hematoxylin-Eosin Staining (HE Staining). The expression of hypoxia-inducible transcription factor-1 alpha (HIF-1 alpha), vascular endothelial growth factor (VEGF), interleukin-1 beta (IL-1 beta) and tumour necrosis factor-alpha (TNF-alpha) was detected using Immunohistochemical (IHC), Western Blot and EnzymeResults: Acupotomy reduced intra-articular hypertension and improved the synovial oxygen situation, synovial inflammatory and angiogenesis. HIF-1 alpha, VEGF, IL-1 beta and TNF-alpha expression were downregulated by acupotomy. Conclusion: Acupotomy may reduce inflammation and angiogenesis in KOA rabbit by reducing abnormally elevated intra-articular pressure and improving synovial oxygen environment. The above may provide a new theoretical foundation for acupotomy treatment of KOA.

C1 Capital Med Univ, Beijing Hosp Tradit Chinese Med, Dept Acupuncture & Moxibust, Beijing Key Lab Acupuncture Neuromodulat, Beijing, Peoples R China; Beijing Univ Chinese Med, Sch Acupuncture Moxibust & Tuina, Beijing, Peoples R China; Capital Med Univ, Beijing Hosp Tradit Chinese Med, Dept Acupuncture & Moxibust, Beijing Key Lab Acupuncture Neuromodulat, 23 Meishuguan Back St, Beijing 100010, Peoples R China

DI 10.2147/JPR.S396955

ID 108

ER

FN Clarivate Analytics Web of science

PT J

AU Zhao, R. L.

AU - Ma, P. H.

AU - Liu, B. Y.

AU - Yu, C. H.

AU - Zhang, H. R.

AU - Lv, Q.

AU - Yang, D. W.

AU - Yang, Y. P.

AU - Liu, H. Y.

AU - Wang, F. Y.

AU - Yin, C. S.

AU - Su, S. G.

AU - Wang, H. C.

AU - Wang, X. Y.

AU - Yan, S. Y.

PY 2023

TI Short-term and long-term effectiveness of acupuncture and Tuina on knee osteoarthritis: study protocol for a randomized controlled trial

DA 2023/1/1

SO FRONTIERS IN NEUROLOGY

JO FRONTIERS IN NEUROLOGY

VL 14

SN 1664-2295

Z9 Times Cited in Web of Science Core Collection: 2 Total Times Cited: 2 Cited Reference Count: 54 ER -

M3 10.3389/fneur.2023.1301217

N1 ZhaoMa-115

N1 2025/2/13 1:16:00

DE knee osteoarthritis; acupuncture; tuina; exercise; randomized controlled trial; telehealth; QUALITY-OF-LIFE; ALTERNATIVE MEDICINE; CLINICAL-TRIALS; THERAPY; PAIN; EXERCISE; HEALTH; HIP; ELECTROACUPUNCTURE; COMPLEMENTARY

AB Background: The effectiveness of acupuncture and tuina in treating knee osteoarthritis (KOA) is still controversial, which limits their clinical application in practice. This study aims to evaluate the short-term and long-term effectiveness of acupuncture and tuina on KOA.Methods/design: This parallel-group, multicenter randomized clinical trial (RCT) will be conducted at the outpatient clinic of five traditional Chinese medicine hospitals in China. Three hundred and thirty participants with KOA will be randomly assigned to acupuncture, tuina, or home-based exercise group with a ratio of 1:1:1. The primary outcome is the proportion of participants achieving a minimal clinically important improvement defined as a >= 12% reduction on the Western Ontario and McMaster Universities Osteoarthritis Index (WOMAC) pain dimension on short term (week 8) and long term (week 26) compared with baseline. Secondary outcomes are knee joint conditions (pain, function, and stiffness), self-efficacy of arthritis, quality of life, and psychological conditions, which will be evaluated by the WOMAC score and the Patient Global Assessment (PGA), and in addition, the respondents index of OMERACT-OARSI, Short Form 12 Health Survey (SF-12), arthritis self-efficacy scale, and European five-dimensional health scale (EQ-5D). Adverse events will be collected by self-reported questionnaires predefined.

C1 Beijing Univ Chinese Med, Acupuncture & Moxibust Dept, Beijing, Peoples R China; China Acad Chinese Med Sci, Beijing, Peoples R China; Beijing Univ Chinese Med, Dongzhimen Hosp, Beijing, Peoples R China; Beijing Youth Polit Coll, Coll Presch Educ, Beijing, Peoples R China; China Acad Chinese Med Sci, Guanganmen Hosp Southern Dist, Beijing, Peoples R China; Weifang Hosp Tradit Chinese Med, Weifang, Peoples R China; Beijing Tradit Chinese Med Hosp, Shunyi Hosp, Beijing, Peoples R China

DI 10.3389/fneur.2023.1301217

ID 115

ER

FN Clarivate Analytics Web of science

PT J

AU Liu, S. F.

AU - Chen, Q. D.

AU - Zhang, Q. G.

AU - Tao, K.

AU - Li, C. H.

AU - Chang, B. L.

AU - Wang, W. F.

AU - Wu, Z.

PY 2023

TI Electroacupuncture combined with extracorporeal shock wave therapy improves pain symptoms and inflammatory factor levels in knee osteoarthritis patients

DA 2023/1/1

SO HELIYON

JO HELIYON

VL 9

IS 10

SN 2405-8440

Z9 Times Cited in Web of Science Core Collection: 0 Total Times Cited: 0 Cited Reference Count: 54 ER -

M3 10.1016/j.heliyon.2023.e20771

N1 LiuChen-141

N1 2025/2/13 1:16:00

DE Electroacupuncture; Extracorporeal shock wave therapy; Knee osteoarthritis; Synovial fluid; PLANTAR FASCIITIS; SUBCHONDRAL BONE; ACUPUNCTURE; CARTILAGE; MODEL

AB Objective: To compare the clinical efficacy and safety of electroacupuncture combined with extracorporeal shock wave therapy (EESWT) and extracorporeal shock wave therapy (ESWT) in the treatment of knee osteoarthritis (KOA).Methods: A total of 135 KOA patients who received EESWT treatment were selected as the EESWT group, and 135 KOA patients who received extracorporeal shock wave therapy (ESWT) were selected as the ESWT group. The clinical efficacy, inflammatory factors in joint synovial fluid and adverse events during treatment were compared before and after treatment.Results: The clinical effective rate of patients in the EESWT group (89.63 %) after treatment was significantly higher than that of the ESWT group (74.81 %) (p < 0.01). The lysholm kness (LKSS) score and range of motion (ROM) of the patients in the EESWT group after treatment were higher than those of the ESWT group, while Lequesne index score, visual analogue scale (VAS) score and Western Ontario and McMaster Universities Arthritis Index (WOMAC) were lower than those of the ESWT group (p < 0.01). Compared with ESWT group, the changes in the expression levels of nitric oxide (NO), superoxide dismutase (SOD), interleukin 1 beta (IL-1 beta), tumor necrosis factor-alpha (TNF-alpha), matrix metalloproteinase-3 (MMP-3), and transforming growth factor beta 1 (TGF-beta 1) in the synovial fluid of the EESWT group after treatment were significantly greater than those of the ESWT group (p < 0.01). No significant difference in the incidence of adverse events between the EESWT group and the ESWT group (p > 0.05).Conclusion: EESWT significantly improves pain symptoms and inflammatory factor levels in KOA patients and is an optional KOA treatment option worthy of clinical attention.

C1 Tongji Univ, Shanghai Peoples Hosp 10, Sch Med, Dept Orthoped, Shanghai 200072, Peoples R China; Fudan Univ, Jingan Dist Ctr Hosp Shanghai, Dept Cent Lab, Clin Lab, Shanghai 200040, Peoples R China; Peoples Hosp Gengma Dai & Va Autonomous Cty, Dept Surg 2, Lincang 677599, Yunnan, Peoples R China; Ma Anshan 17 Met Hosp, Maanshan 243000, Anhui, Peoples R China; Shanghai Univ Tradit Chinese Med, Longhua Hosp, Dept Lab Med, Shanghai 200032, Peoples R China; 301 Yanchang Zhong Rd, Shanghai 200072, Peoples R China

DI 10.1016/j.heliyon.2023.e20771

ID 141

ER

FN Clarivate Analytics Web of science

PT J

AU Kwak, S. G.

AU - Kwon, J. B.

AU - Seo, Y. W.

AU - Choi, W. K.

PY 2023

TI The effectiveness of acupuncture as an adjunctive therapy to oral pharmacological medication in patient with knee osteoarthritis: A systematic review and meta-analysis

DA 2023/1/1

SO MEDICINE

JO MEDICINE

VL 102

IS 11

SN 0025-7974

Z9 Times Cited in Web of Science Core Collection: 1 Total Times Cited: 1 Cited Reference Count: 32 ER -

M3 10.1097/MD.0000000000033262

N1 KwakKwon-143

N1 2025/2/13 1:16:00

DE acupuncture; knee; VAS; WOMAC; PAIN; MANAGEMENT; EFFICACY; GUIDELINE; PLACEBO; HIP

AB Background:We aimed to find out whether the combined treatment of acupuncture and oral medication is more effective than sole oral medication in reducing pain and improving knee function at the end of treatment and after short-term period (4-6 weeks after treatment). Second, if it is effective, we investigated whether the effect surpasses the minimal clinically important difference. Methods:Articles published between January 1, 1992, and August 31, 2022, were searched in PubMed, Cochrane, and Embase. The PICO (population, intervention, comparison, and outcome) of this study are as follows: Population: knee osteoarthritis patients; Intervention: acupuncture (non-sham acupuncture) + oral medication (analgesic or non-steroidal anti-inflammatory drugs); Comparison: oral medication (analgesic or non-steroidal anti-inflammatory drugs); Outcome: visual analog scale (VAS) or Western Ontario and McMaster University (WOMAC) osteoarthritis index Results:The combined treatment of oral medication and adjuvant acupuncture showed statistically significant improvement in VAS and WOMAC scores at the end of acupuncture treatment and short-term follow-up time (between 4 and 6 weeks after acupuncture). In addition, the degree of improvement of VAS and WOMAC index showed effects beyond minimal clinically important differences compared to pretreatment at both the end of acupuncture treatment and the short-term follow-up of acupuncture treatment. Conclusion:The existing evidence suggests that adjuvant acupuncture may play a role in the treatment of knee osteoarthritis. However, physicians should be aware of adverse effects such as hematoma in adjuvant acupuncture treatment.

C1 Daegu Catholic Univ, Coll Med, Dept Med Stat, Daegu, South Korea; Daegu Catholic Univ, Coll Med, Dept Orthopaed Surg, Daegu, South Korea; Daegu Catholic Univ, Coll Med, Dept Emergency Med, Daegu, South Korea; Daegu Catholic Univ Hosp, Dept Orthopaed Surg, 3056-6,Daemyung-4-dong, Daegu, South Korea

DI 10.1097/MD.0000000000033262

ID 143

ER

FN Clarivate Analytics Web of science

PT J

AU Xu, W. J.

AU - Xiao, Y.

AU - Zhao, M. Z.

AU - Zhu, J. H.

AU - Wang, Y.

AU - Wang, W. B.

AU - Wang, P.

AU - Meng, H.

PY 2023

TI Effective Treatment of Knee Osteoarthritis Using a Nano-Enabled Drug Acupuncture Technology in Mice

DA 2023/1/1

SO ADVANCED SCIENCE

JO ADVANCED SCIENCE

SN 2198-3844

Z9 Times Cited in Web of Science Core Collection: 8 Total Times Cited: 8 Cited Reference Count: 60 ER -

M3 10.1002/advs.202302586

N1 XuXiao-145

N1 2025/2/13 1:16:00

DE acupuncture; knee osteoarthritis; lidocaine; nano drug delivery; nd-Acu; BETA-CYCLODEXTRIN; INCLUSION COMPLEXES; MOUSE MODEL; KAPPA-B; ELECTROACUPUNCTURE; RECEPTOR; PAIN; EXPRESSION; RELEASE; RATS

AB A nano-enabled drug delivery acupuncture technology (nd-Acu) is developed that is based on traditional acupuncture needles where the stainless-steel surface is designed to deliver various payload molecules. To create the nd-Acu platform, an electrochemistry procedure is used to attach methyl salicylate-modified cyclodextrin in which the sugar rings allow the encapsulation of structurally defined single or multiple payload molecules via an inclusion complexation process. Drug loading and release profile are first studied using fluorescent dyes abiotically and at intact animal level. nd-Acu allows more efficient dye loading and time-dependent release compared to pristine needles without cyclodextrin modification. Subsequently, a proof-of-principle efficacy study is conducted using the platform to load a local anesthetic, lidocaine, for the treatment of knee osteoarthritis (KOA) in mice. It is demonstrated that lidocaine-laden nd-Acu can effectively alleviate pain, reduce inflammation, and slow down KOA development biochemically and histologically. Hypothesis-driven and proteomic approaches are utilized to investigate the working mechanisms of lidocaine nd-Acu, indicating that the therapeutic outcome is attributed to the in vivo modulation of the HMGB1/TLR4 signaling pathway. The study also obtained preliminary evidence suggesting the involvement of mitochondria as well as small GTPase such as cdc42 during the treatment by lidocaine nd-Acu.

C1 Natl Ctr Nanosci & Technol, CAS Ctr Excellence Nanosci, CAS Key Lab Biomed Effects Nanomat & Nanosafety, Beijing 100190, Peoples R China; Capital Med Univ, Beijing Hosp Tradit Chinese Med, Beijing 100010, Peoples R China; Univ Chinese Acad Sci, Beijing 100049, Peoples R China; Chongqing Univ Technol, Chongqing 400054, Peoples R China; Zhengzhou Univ, Affiliated Hosp 1, Zhengzhou 450052, Henan, Peoples R China

DI 10.1002/advs.202302586

ID 145

ER

FN Clarivate Analytics Web of science

PT J

AU Li, P. Q.

AU - Zhang, Y. C.

AU - Li, F. L.

AU - Cai, F. H.

AU - Xiao, B.

AU - Yang, H. Y.

PY 2023

TI The Efficacy of Electroacupuncture in the Treatment of Knee Osteoarthritis: A Systematic Review and Meta-Analysis

DA 2023/1/1

SO ADVANCED BIOLOGY

JO ADVANCED BIOLOGY

VL 7

IS 10

SN 2701-0198

Z9 Times Cited in Web of Science Core Collection: 3 Total Times Cited: 4 Cited Reference Count: 87 ER -

M3 10.1002/adbi.202200304

N1 LiZhang-147

N1 2025/2/13 1:16:00

DE electroacupuncture; knee osteoarthritis; meta-analysis; randomized controlled trials; PAIN; THERAPY; RECOMMENDATIONS; RHEUMATOLOGY; MANAGEMENT; SAFETY; INDEX; HIP

AB This study aims to evaluate the comparative efficacy of electroacupuncture (EA) and analgesics in treating knee osteoarthritis (KOA) and provide evidence-based medical support for EA for the treatment of KOA. Randomized controlled trials from January 2012 to December 2021 are included in electronic databases. The Cochrane risk of bias tool for randomized trials is used to assess the risk of bias in the included studies, while the Grading of Recommendations, Assessment, Development and Evaluation is used to assess the quality of evidence. Statistical analyses are performed using Review Manager V5.4. There are 1616 patients from 20 clinical studies, including 849 patients in the treatment group and 767 patients in the control group. The effective rate in the treatment group is significantly higher than in the control group (p < 0.00001). In the treatment group, Western Ontario and McMaster Universities Osteoarthritis Index (WOMAC) stiffness scores are significantly improved as compared to the control group (p < 0.0001). However, EA is similar to analgesics in improving visual analog scale scores and WOMAC subitems such as pain and joint function. EA is effective in treating KOA because it can significantly improve clinical symptoms and quality of life in KOA patients.

C1 Sch Acupuncture Moxibust, Shanghai, Peoples R China; Tuina Shanghai Univ, Tradit Chinese Med Shanghai, Shanghai 201203, Peoples R China

DI 10.1002/adbi.202200304

ID 147

ER

FN Clarivate Analytics Web of science

PT J

AU Ye, J. N.

AU - Su, C. G.

AU - Jiang, Y. Q.

AU - Zhou, Y.

AU - Sun, W. X.

AU - Zheng, X. X.

AU - Miao, J. T.

AU - Li, X. Y.

AU - Zhu, J.

PY 2023

TI Effects of acupuncture on cartilage p38MAPK and mitochondrial pathways in animal model of knee osteoarthritis: A systematic evaluation and meta-analysis

DA 2023/1/1

SO FRONTIERS IN NEUROSCIENCE

JO FRONTIERS IN NEUROSCIENCE

VL 16

SN 1662-453X

Z9 Times Cited in Web of Science Core Collection: 1 Total Times Cited: 2 Cited Reference Count: 69 ER -

M3 10.3389/fnins.2022.1098311

N1 YeSu-172

N1 2025/2/13 1:16:00

DE knee osteoarthritis; acupuncture; animal models; p38MAPK pathways; mitochondrial pathways; cytokines; meta-analysis; DEGENERATION; INFLAMMATION; APOPTOSIS; KINASES; PAIN

AB Background: Most previous studies on acupuncture in the treatment of knee osteoarthritis (KOA) have focused on improving functional efficacy and safety, while related mechanisms have not been systematically reviewed. Acupuncture modulates cytokines to attenuate cartilage extracellular matrix degradation and apoptosis, key to the pathogenesis of KOA, but the mechanisms are complex.Objectives: The purpose of this study is to assess the efficacy of acupuncture quantitatively and summarily in animal studies of KOA.Methods: Nine databases including PubMed, Embase, Web of Science (including Medline), Cochrane library, Scopus, CNKI, Wan Fang, and VIP were searched to retrieve animal studies on acupuncture interventions in KOA published since the inception of the journal. Relevant literature was screened, and information extracted. Meta-analysis was performed using Revman 5.4 and Stata 17.0 software.Results: The 35 included studies involved 247 animals, half of which were in acupuncture groups and half in model groups. The mean quality level was 6.7, indicating moderate quality. Meta-analysis showed that acupuncture had the following significant effects on cytokine levels in p38MAPK and mitochondrial pathways: (1) p38MAPK pathway: It significantly inhibits p38MAPK, interleukin-1beta (IL-1 beta), tumor necrosis factor alpha (TNF-alpha), phosphorylated (p)-p38MAPK, matrix metalloproteinase-13 (MMP-13), MMP-1, a disintegrin and metalloproteinase with thrombospondin motifs-5 (ADAMST-5) expression, and significantly increased the expression of collagen II and aggrecan. (2) mitochondrial pathway: It significantly inhibited the expression of Bcl-2-associated X protein (Bax), cysteine protease-3 (caspase-3), caspase-9, and Cytochrome-c (Cyt-c). And significantly increased the expression of B cell lymphocytoma-2 (Bcl-2). In addition, acupuncture significantly reduced chondrocyte apoptosis, Mankin's score (a measure of cartilage damage), and improved cartilage morphometric characteristics.Conclusion: Acupuncture may inhibit cytokine expression in the p38MAPK pathway to attenuate cartilage extracellular matrix degradation, regulate cytokines in the mitochondrial pathway to inhibit chondrocyte apoptosis, and improve cartilage tissue-related phenotypes to delay cartilage degeneration. These findings provide possible explanations for the therapeutic mechanisms and clinical benefits of acupuncture for KOA.

C1 Chengdu Univ Tradit Chinese Med, Sch Acupuncture Moxibust & Tuina, Chengdu, Peoples R China; China Acad Chinese Med Sci, Xiyuan Hosp, Beijing, Peoples R China; Guangzhou Univ Chinese Med, Grad Sch, Guangzhou, Peoples R China

DI 10.3389/fnins.2022.1098311

ID 172

ER

FN Clarivate Analytics Web of science

PT J

AU Venuti, A. J.

AU - Chiu, J. P.

AU - Yu, K. C.

AU - Chang, S. C.

AU - Lin, S. Y.

AU - Hsu, C. H.

PY 2023

TI Chinese Herbal Fumigation Steam Therapy and Acupuncture in the Treatment of Knee Osteoarthritis: A Three-armed, Randomized, Controlled Trial

PG 243-257

DA 2023/1/1

SO ALTERNATIVE THERAPIES IN HEALTH AND MEDICINE

JO ALTERNATIVE THERAPIES IN HEALTH AND MEDICINE

VL 29

IS 2

SN 1078-6791

Z9 Times Cited in Web of Science Core Collection: 0 Total Times Cited: 0 Cited Reference Count: 59 ER -

N1 VenutiChiu-180

Y2 2025/1/17 14:41:00

N1 2025/2/13 1:16:00

DE QUALITY-OF-LIFE; HEAT; PAIN; MEDICINE; VALIDITY; HIP; RELIABILITY; CARTILAGE; RESPONSES; HAND

AB Context . Knee osteoarthritis is a common form of joint disease found in humans and one of the leading causes of disability globally. Knee osteoarthritis (KOA) is responsible for a higher number of disabilities than any other medical condition affecting activities of daily living (ADL). To date no definitive, conventional medical protocol is available to deal with KOA. Objectives . The study intended to clinically investigate whether the benefits of acupuncture in the treatment of KOA) could be augmented by the addition of Chinese herbal fumigation steam therapy (CHFST) to the treatment protocol and to what degree it had benefits. Design . The research team designed a three-armed, randomized, controlled trial. The sample size was determined by analysis of power; for a sample size of 42, the power was 83.5%; the effect size was 0.5; and the alpha was 0.05. Setting . The setting was the traumatology department in the Chinese Medicine Branch of Taipei City Joint Hospital in Kun Ming, Taiwan. Participants . Participants were 45 males and females between the ages of 35 and 75, who were patients in the traumatology department and who were suffering from bilateral knee osteoarthritis. Three participants were lost to follow-up. Interventions . Participants were randomly assigned to one of three groups, each with 15 participants; (1) the control group, who received acupuncture only; (2) the normal CHFST group, an intervention group who received acupuncture plus a normal dose of CHFST; and (3) the one-sixteenth CHFST group, an intervention group who received acupuncture plus one-sixteenth of a normal dose of CHFST. Participants underwent biweekly treatments for four consecutive weeks. Outcomes Measures . The primary outcome measures included a visual analog scale (VAS) to assess variations in pain intensity and a goniometer measure for range of motion (ROM). Results . The addition of CHFST to acupuncture in the treatment of KOA significantly reduced pain (P =.0017) for the normal CHFST group compared to the control group and the one-sixteenth CHFST group. Chinese medical interventions overall for all groups showed a decrease in pain and increases in ROM, and health related quality of life (HrQoL). Conclusions . CHFST, in conjunction with acupuncture, showed promise in the treatment of KOA in reducing pain, increasing ROM, and improving quality of life (QoL).

C1 Natl Yang Ming Chiao Tung Univ, Inst Tradit Med, Taipei, Taiwan; Taipei City Hosp, Dept Educ & Res, Da An Dist, Taiwan; Taipei City Hosp, Div Chinese Traumatol, Dept Chinese Med, Linsen & Kun Ming Branch, Taipei, Taiwan; Taipei City Hosp, Dept Chinese Med, Linsen & Kun Ming Branch, Taipei, Taiwan; Taipei City Hosp, Dept Educ & Res, Taipei, Taiwan

ID 180

ER

FN Clarivate Analytics Web of science

PT J

AU Xing, L. F.

AU - Chen, X. L.

AU - Guo, C. Q.

AU - Zhu, W. T.

AU - Hu, T. Y.

AU - Ma, W. W.

AU - Du, M.

AU - Xu, Y.

AU - Guo, C. Q.

PY 2023

TI Electroacupuncture Exerts Chondroprotective Effect in Knee Osteoarthritis of Rabbits Through the Mitophagy Pathway

PG 2871-2882

DA 2023/1/1

SO JOURNAL OF PAIN RESEARCH

JO JOURNAL OF PAIN RESEARCH

VL 16

SN 1178-7090

Z9 Times Cited in Web of Science Core Collection: 2 Total Times Cited: 3 Cited Reference Count: 41 ER -

M3 10.2147/JPR.S416242

N1 XingChen-181

Y2 2025/1/18 22:10:00

N1 2025/2/13 1:16:00

DE osteoarthritis; electroacupuncture; mitophagy; mitochondria; Pink1-Parkin; ARTICULAR-CARTILAGE; MODEL

AB Purpose: Mitochondrial dysfunction of chondrocytes has become an area of focus in Knee Osteoarthritis (KOA) in recent years. Activation of mitophagy could promote the survival of chondrocytes and alleviate cartilage degeneration. The aim of this study was to explore whether mitophagy was involved in the cartilage protection of KOA rabbits after electroacupuncture (EA) intervention.Methods: The rabbits were divided into 3 groups, Control group, KOA group, EA group, with 6 rabbits in each group. KOA model rabbits were established by modified Videman's extended immobilization method for 6 weeks and randomly divided into KOA group and EA group. The rabbits in EA group were treated every other day for 3 weeks. The degree of cartilage degeneration was detected by Safranine O-Fast Green staining and immunofluorescence. The morphological changes of chondrocytes mitochondria were detected by transmission electron microscope. ATP concentration in cartilage was measured by ATP Assay Kit. The changes of Pink1-Parkin signal pathway were detected by immunofluorescence, Western blot, and Real-time PCR.Results: The morphology showed that EA could reduce the degeneration of KOA cartilage and increase the distribution of collagen II. We also found that EA could activate mitophagy in KOA rabbit chondrocytes to remove damaged mitochondria and restore mitochondrial homeostasis, which was manifested as increasing the expression of LC3 II/I, promoting the colocalization of TOM20 and LC3B, reducing the accumulation of mitochondrial markers outer mitochondrial membrane 20 (TOM20) and inner mitochondrial membrane 23 (TIM23), and increasing ATP production in chondrocytes. This regulation might be achieved by upregulating the Pink1-Parkin signal pathway.Conclusion: EA may play a role in protecting KOA cartilage by activating mitophagy mediated through Pink1-Parkin pathway.

C1 Beijing Univ Chinese Med, Sch Acupuncture Moxibust & Tuina, Beijing, Peoples R China; Guangzhou Univ Tradit Chinese Med, Coll Clin Med 5, Dept Acupuncture & Rehabil, Guangzhou, Peoples R China; Guangdong Second Hosp Tradit Chinese Med, Dept Acupuncture & Rehabil, Guangzhou, Peoples R China; Beijing Univ Chinese Med, Affiliated Hosp 3, Beijing 100029, Peoples R China; Shijiazhuang Med Coll, Dept Med Technol, Shijiazhuang, Hebei, Peoples R China; Shijiazhuang Med Coll, Shijiazhuang 050599, Hebei, Peoples R China

DI 10.2147/JPR.S416242

ID 181

ER

FN Clarivate Analytics Web of science

PT J

AU Chen, W.

AU - Zhang, X. N.

AU - Su, Y. S.

AU - Wang, X. Y.

AU - Li, H. C.

AU - Liu, Y. H.

AU - Wan, H. Y.

AU - Qu, Z. Y.

AU - Jing, X. H.

AU - He, W.

PY 2023

TI Electroacupuncture activated local sympathetic noradrenergic signaling to relieve synovitis and referred pain behaviors in knee osteoarthritis rats

DA 2023/1/1

SO FRONTIERS IN MOLECULAR NEUROSCIENCE

JO FRONTIERS IN MOLECULAR NEUROSCIENCE

VL 16

SN 1662-5099

Z9 Times Cited in Web of Science Core Collection: 9 Total Times Cited: 11 Cited Reference Count: 48 ER -

M3 10.3389/fnmol.2023.1069965

N1 ChenZhang-191

N1 2025/2/13 1:16:00

DE knee osteoarthritis; electroacupuncture; synovitis; macrophage; sympathetic noradrenergic signaling; CXCL1; IL-6; MOLECULAR-MECHANISMS; IN-VIVO; ARTHRITIS; NERVE; INFLAMMATION; RECRUITMENT; MACROPHAGES; ACUPUNCTURE; PROGRESSION; SUPPRESSOR

AB Introduction: Recent research has focused on the local control of articular inflammation through neuronal stimulation to avoid the systemic side effects of conventional pharmacological therapies. Electroacupuncture (EA) has been proven to be useful for inflammation suppressing and pain reduction in knee osteoarthritis (KOA) patients, yet its mechanism remains unclear. Methods: In the present study, the KOA model was established using the intra-articular injection of sodium monoiodoacetate (MIA) (1 mg/50 mu L) into the knee cavity. EA was delivered at the ipsilateral ST36-GB34 acupoints. Hind paw weight-bearing and withdrawl thresholds were measured. On day 9, the histology, dep enrichment proteins, cytokines contents, immune cell population of the synovial membrane of the affected limbs were measured using HE staining, Masson staining, DIA quantitative proteomic analysis, flow cytometry, immunofluorescence staining, ELISA, and Western Blot. The ultrastructure of the saphenous nerve of the affected limb was observed using transmission electron microscopy on the 14th day after modeling. Results: The result demonstrated that EA intervention during the midterm phase of the articular inflammation alleviated inflammatory pain behaviors and cartilage damage, but not during the early phase. Mid-term EA suppressed the levels of proinflammatory cytokines TNF-alpha, IL-1 beta, and IL-6 in the synovium on day 9 after MIA by elevating the level of sympathetic neurotransmitters Norepinephrine (NE) in the synovium but not systemic NE or systemic adrenaline. Selective blocking of the sympathetic function (6-OHDA) and beta 2-adrenergic receptor (ICI 118,551) prevented the anti-inflammatory effects of EA. EA-induced increment of the NE in the synovium inhibited the CXCL1-CXCR2 dependent overexpression of IL-6 in the synovial macrophages in a beta 2-adrenergic receptor (AR)-mediated manner. Discussion: These results revealed that EA activated sympathetic noradrenergic signaling to control local inflammation in KOA rats and contributed to the development of novel therapeutic neurostimulation strategies for inflammatory diseases.

C1 China Acad Chinese Med Sci, Inst Acupuncture & Moxibust, Beijing, Peoples R China

DI 10.3389/fnmol.2023.1069965

ID 191

ER

FN Clarivate Analytics Web of science

PT J

AU Chen, J.

AU - Guo, H.

AU - Pan, J. H.

AU - Li, H. P.

AU - Wang, Y. S.

AU - Liu, Z. X.

AU - Xie, Y. L.

AU - Jin, S.

PY 2023

TI Efficacy of acupuncture combined with active exercise training in improving pain and function of knee osteoarthritis individuals: a systematic review and meta-analysis

DA 2023/1/1

SO JOURNAL OF ORTHOPAEDIC SURGERY AND RESEARCH

JO JOURNAL OF ORTHOPAEDIC SURGERY AND RESEARCH

VL 18

IS 1

SN 1749-799X

Z9 Times Cited in Web of Science Core Collection: 1 Total Times Cited: 1 Cited Reference Count: 66 ER -

M3 10.1186/s13018-023-04403-2

N1 ChenGuo-228

Y2 2025/1/17 14:44:00

N1 2025/2/13 1:16:00

DE Acupuncture; Active exercise training; Knee osteoarthritis; Systematic review; Meta-analysis; CLINICAL-PRACTICE GUIDELINES; ELECTROACUPUNCTURE; MANAGEMENT

AB Objective To conduct a systematic review and meta-analysis to investigate the clinical efficacy of acupuncture combined with active exercise training in improving pain and function of knee osteoarthritis (KOA) individuals.Data sources PubMed, EMBASE, The Cochrane Library, Web of Science, China National Knowledge Infrastructure, Wan Fang Data, Technology Periodical Database and China Biology Medicine were searched from their inceptions to April 5, 2023.Review methods We analyzed trials of acupuncture combined with active exercise training for KOA. The included studies were of high quality (Jadad >= 4) and RCTs. Study selection, data extraction, risk of bias and quality assessment were independently performed by two reviewers. We performed systematic analyses based on different outcome measures, including total efficiency rate, visual analogue scale (VAS), the Western Ontario and Mcmaster Universities Osteoarthritis Index (WOMAC), the Lysholm Knee Scale (LKS) and range of motion (ROM). We used Review Manager 5.3 and Stata/MP 14.0 to analyze the data. And it was verified by trial sequence analysis (TSA). If I-2 > 50% and p < 0.05, we performed sensitivity analysis and subgroup analysis to find the source of heterogeneity. Publication bias was studied by funnel plot and Egger's test was used to verify it.Results Full 11 high-quality studies (Jadad >= 4) including 774 KOA individuals were included in this review for meta-analysis. The results showed that acupuncture combined with active exercise training (combined group) was superior to the acupuncture group in improving the total effective rate [RR = 1.13, 95%CI (1.05, 1.22), I-2 = 0%, P = 0.70], reducing the pain level (VAS) [MD = - 0.74, 95%CI (- 1.04, - 0.43), I-2 = 68%, P < 0.05], improving knee joint function (WOMAC) [MD = - 6.97, 95%CI (- 10.74, - 3.19), I-2 = 76%, P < 0.05] and improving joint range of motion (ROM) [MD = 6.25, 95%CI (2.37, 10.04), I-2 = 0%, P = 0.71]. Similarly, the combined group showed significant improvements in the total effective rate [RR = 1.31, 95% CI (1.18, 1.47), I-2 = 48%, P = 0.10], pain (VAS) [MD = 1.42, 95% CI (- 1.85, - 1.00), I-2 = 65%, P = 0.02] and knee function (WOMAC) [MD = 7.05, 95% CI (- 11.43, - 2.66), I-2 = 86%, P < 0.05] compared with the non-acupuncture group.Conclusion The combined effect of all studies showed significant benefits of acupuncture combined with active exercise training in improving the total effective rate, reducing pain, promoting recovery of knee function and expanding range of motion. However, some evaluation indicators are highly subjective and need to be further confirmed by more objective and evidence-based high-quality RCTs in future.

C1 Chengdu Univ Tradit Chinese Med, Sch Hlth Preservat & Rehabil, Chengdu, Sichuan, Peoples R China; Hosp Chengdu Univ Tradit Chinese Med, Rehabil Dept, 39,12 Bridge Rd, Chengdu 610000, Sichuan, Peoples R China; Chengdu Univ Tradit Chinese Med, Sch Med & Life Sci, Chengdu, Sichuan, Peoples R China

DI 10.1186/s13018-023-04403-2

ID 228

ER

FN Clarivate Analytics Web of science

PT J

AU Hyun, E.

AU - Shin, B. C.

AU - Kim, N.

AU - Lim, B.

PY 2023

TI Economic evaluation of acupuncture as an adjunctive treatment with usual care for mild-to-moderate knee osteoarthritis: A Markov model-based analysis

DA 2023/1/1

SO INTEGRATIVE MEDICINE RESEARCH

JO INTEGRATIVE MEDICINE RESEARCH

VL 12

IS 3

SN 2213-4220

Z9 Times Cited in Web of Science Core Collection: 0 Total Times Cited: 0 Cited Reference Count: 77 ER -

M3 10.1016/j.imr.2023.100982

N1 HyunShin-233

N1 2025/2/13 1:16:00

DE Acupuncture; Economic evaluation; Knee osteoarthritis; Korean medicine; Markov model; COST-EFFECTIVENESS; PHYSICAL-THERAPY; HIP; ARTHROPLASTY; PREVALENCE; MANAGEMENT; HAND

AB Background: Collaborative care (CC), in which acupuncture is combined with usual care (UC), improves clinical outcomes and increases costs in knee osteoarthritis (KOA). We evaluated the economic feasibility of CC for Korean female patients with mild-to-moderate KOA by using a cost-effectiveness approach.Methods: Two alternatives for KOA (1. UC as a comparator; and 2. CC as an intervention) were defined based on clinical guidelines, official Korean statistics, and expert validation. Each alternative was simulated in a Markov model every 6 months for 10 years. Estimates of costs, utilities, and transition probabilities were obtained from official statistics and previous studies. The effectiveness of CC was synthesized from randomized controlled trials. A base-case analysis of a limited societal perspective, univariate sensitivity analysis, and probability sensitivity analysis were performed. An annual discount rate of 4.5% and threshold of 20,000 United States dollar per Quality-adjusted life year (USD/QALY) were applied.Results: Every incremental cost-effectiveness ratio (ICER) of CC calculated from the analyses was sub-threshold. In the base-case analysis, with a limited societal perspective, the ICER was 11,085 USD/QALY. The ICERs from the univariate sensitivity analyses were -2,577-16,748 USD/QALY. The average ICER in the probability sensitivity analysis was 12,412 USD/QALY. When the threshold surpassed 8,000 USD/QALY, the cost-effectiveness of CC exceeded 50%. The probability was 70.27% when the threshold was 20,000 USD/QALY.Conclusions: CC for Korean female patients with mild-to-moderate KOA was generally cost-effective. Considering the limitations of the evidence, we propose a re-evaluation using further clinical studies in the future.

C1 Seoul Natl Univ, Inst Hlth Policy & Management, Med Res Ctr, Seoul, South Korea; Pusan Natl Univ, Korean Med Hosp, Dept Korean Med Rehabil, Yangsan, South Korea; Pusan Natl Univ, Ctr Big data & Comparat Effectiveness Res Econ Eva, Yangsan, South Korea; Pusan Natl Univ, Sch Korean Med, Div Humanities & Social Med, Yangsan, South Korea; Pusan Natl Univ, Sch Korean Med, Div Humanities & Social Med, Busandaehak ro 49, Yangsan 50612, South Korea

DI 10.1016/j.imr.2023.100982

ID 233

ER

FN Clarivate Analytics Web of science

PT J

AU Xin, S. Y.

AU - Liu, J. Y.

AU - Yang, Z. X.

AU - Li, C. H.

PY 2023

TI Comparative effectiveness of moxibustion and acupuncture for the management of osteoarthritis knee: A systematic review and meta-analysis

DA 2023/1/1

SO HELIYON

JO HELIYON

VL 9

IS 7

SN 2405-8440

Z9 Times Cited in Web of Science Core Collection: 1 Total Times Cited: 1 Cited Reference Count: 50 ER -

M3 10.1016/j.heliyon.2023.e17805

N1 XinLiu-259

Y2 2025/1/17 15:25:00

N1 2025/2/13 1:16:00

DE Acupuncture; Meta-analysis; Moxibustion; Osteoarthritis; RELEASE; HEALTH

AB Background: Moxibustion, a traditional Chinese medicine approach, stimulates blood circulation by burning wormwood at acupuncture points and is frequently used in conjunction with acupuncture for managing knee osteoarthritis. This review aims to compare the effectiveness of moxibustion and acupuncture in the management of knee osteoarthritis. Methods: Our team conducted a comprehensive search across several databases: PubMed Central, EMBASE, MEDLINE, the Chinese Biomedical Literature Database (CBM), China National Knowledge Infrastructure (CNKI), and the Cochrane Library, covering the timeframe from January 1964 up until April 2022. We implemented a meta-analysis, utilizing a random-effects model, and we've presented the pooled standardized mean difference (SMD) and risk ratio (RR) inclusive of the 95% confidence intervals (CIs), in accordance with the nature of the outcome. Results: 21 studies were included, of which, half were identified as having high risk of bias. The pooled SMD for the pain score was found to be 0.53 (95% CI: 0.91 to 0.15). In-depth analysis focusing on the kind of moxibustion indicated that fire needle moxibustion was more effective in pain reduction (SMD = -0.56; 95% CI: -1.10 to -0.01) compared to alternative moxibustion methods (SMD = -0.47; 95% CI: -0.80 to -0.13). The pooled RR for the success rate in treatment was 1.39 (95% CI: 1.19 to 1.62). Subgroup analysis demonstrated that fire needle moxibustion reported a superior success rate (RR = 1.43; 95% CI: 1.19 to 1.72) in comparison to other types of moxibustion (RR = 1.33; 95% CI: 1.02 to 1.74). Conclusion: Moxibustion, specifically fire needle moxibustion, demonstrated superior effectiveness in managing knee osteoarthritis compared to acupuncture.

C1 Chengde Med Univ, Chengde 067000, Hebei, Peoples R China; Hebei Key Lab Nerve Injury & Repair, Chengde 067000, Hebei, Peoples R China; Capital Med Univ, Beijing Elect Power Teaching Hosp, Dept Acupuncture & Physiotherapy, Beijing 100073, Peoples R China

DI 10.1016/j.heliyon.2023.e17805

ID 259

ER

FN Clarivate Analytics Web of science

PT J

AU Zhang, J. P.

AU - Shen, J.

AU - Xiang, Y. T.

AU - Xing, X. X.

AU - Kang, B. X.

AU - Zhao, C.

AU - Wu, J. J.

AU - Zheng, M. X.

AU - Hua, X. Y.

AU - Xiao, L. B.

AU - Xu, J. G.

PY 2023

TI Modulation of Brain Network Topological Properties in Knee Osteoarthritis by Electroacupuncture in Rats

PG 1595-1605

DA 2023/1/1

SO JOURNAL OF PAIN RESEARCH

JO JOURNAL OF PAIN RESEARCH

VL 16

SN 1178-7090

Z9 Times Cited in Web of Science Core Collection: 3 Total Times Cited: 3 Cited Reference Count: 40 ER -

M3 10.2147/JPR.S406374

N1 ZhangShen-261

N1 2025/2/13 1:16:00

DE osteoarthritis; electroacupuncture; pain; spatio-temporal analysis; neural plasticity; INTERSTITIAL NUCLEUS; ANTERIOR COMMISSURE; POSTERIOR LIMB; PAIN; CONNECTIVITY; ACUPUNCTURE; HIP

AB Introduction: Osteoarthritis is a chronic, ongoing disease that affects patients, and pain is considered a key factor affecting patients, but the brain changes during the development of osteoarthritis pain are currently unclear. In this study, we used electroacupuncture (EA) to intervene the rat model of knee osteoarthritis and analyzed the changes in topological properties of brain networks using graph theory.Methods: Sixteen SD rat models of right-knee osteoarthritis with anterior cruciate ligament transection (ACLT) were randomly divided into electroacupuncture intervention group and control group. The electroacupuncture group was intervened on Zusanli (ST36) and Futu (ST32) for 20 min each time, five times a week for 3 weeks, while the control group was applied sham stimulation. Both groups were measured for pain threshold. The small-world properties and node properties of the brain network between the two groups after the intervention were statistically analyzed by graph theory methods. Results: The differences are mainly in the changes in node attributes between the two groups, such as degree centrality, betweenness centrality, and so on in different brain regions (P<0.05). Both groups showed no small-world characteristics in the brain networks of the two groups. The mechanical thresholds and thermal pain thresholds were significantly higher in the EA group than in the control group (P<0.05).Conclusion: The study demonstrated that electroacupuncture intervention enhanced the activity of nodes related to pain circuit and relieved pain in osteoarthritis, which provides a complementary basis for explaining the effect of electroacupuncture intervention on pain through graphical analysis of changes in brain network topological properties and helps to develop an imaging model for pain affected

C1 Shanghai Univ Tradit Chinese Med, Sch Rehabil Sci, Shanghai, Peoples R China; Guanghua Hosp Integrat Chinese & Western Med, Dept Orthoped, Shanghai, Peoples R China; Shanghai Univ Tradit Chinese Med, Arthrit Inst Integrated Tradit Chinese & Western M, Shanghai Acad Tradit Chinese Med, Shanghai, Peoples R China; Shanghai Univ Tradit Chinese Med, Yueyang Hosp Integrated Tradit Chinese & Western M, Dept Rehabil Med, Shanghai, Peoples R China; Henan Univ Chinese Med, Affiliated Hosp 1, Zhengzhou, Peoples R China; Shanghai Univ Tradit Chinese Med, Yueyang Hosp Integrated Tradit Chinese & Western M, Dept Traumatol & Orthoped, Shanghai, Peoples R China; Minist Educ, Engn Res Ctr Tradit Chinese Med Intelligent Rehabi, Shanghai, Peoples R China; Shanghai Univ Tradit Chinese Med, Sch Rehabil Sci, 1200 Cailun Rd, Shanghai, Peoples R China; Guanghua Hosp Integrat Chinese & Western Med, Dept Orthoped, 540 Xinhua Rd, Shanghai, Peoples R China

DI 10.2147/JPR.S406374

ID 261

ER

FN Clarivate Analytics Web of science

PT J

AU Yang, J. X.

AU - Lan, X. D.

AU - Cai, Q. C.

AU - Lu, Z. H.

AU - Wang, Y. J.

PY 2023

TI Exercise Plus Acupuncture on Consensus Acupoints Versus Acupoints Selected by the Theory of Equal Impact on Tendons, Bones, and Muscles for Knee Osteoarthritis

PG 262-267

DA 2023/1/1

SO ALTERNATIVE THERAPIES IN HEALTH AND MEDICINE

JO ALTERNATIVE THERAPIES IN HEALTH AND MEDICINE

VL 29

IS 5

SN 1078-6791

Z9 Times Cited in Web of Science Core Collection: 0 Total Times Cited: 0 Cited Reference Count: 35 ER -

N1 YangLan-278

N1 2025/2/13 1:16:00

DE NF-KAPPA-B; MATRIX METALLOPROTEINASES; MEDICAL-MANAGEMENT; RECOMMENDATIONS; PREVALENCE; PAIN

AB Context center dot Knee osteoarthritis (KOA) is a degenerative disorder that significantly affects patients' quality of life. Acupuncture and exercise are the most popular treatments currently. The outcomes for acupuncture for KOA, however, are controversial, with some researchers finding that the addition of acupuncture to exercise therapy provided no additional improvement in pain scores. Objective center dot The study intended to evaluate the therapeutic effects for KOA of exercise in combination with acupuncture on acupoints selected using the Traditional Chinse Medicine (TCM) theory of Equal Impact on Tendons, Bones, and Muscles (EITBM) in comparison with that of acupoints selected using classical consensus for the treatment. Design center dot The research team performed a randomized controlled trial. Setting center dot The study took place in the Department of Acupuncture and Moxibustion at the First Affiliated Hospital of Hebei University of Chinese Medicine in Shijiazhuang, Hebei, China. Participants center dot Participants were 70 patients with KOA who visited the hospital between December 2020 and February 2021. Intervention center dot Participants in both group received acupuncture plus exercise therapy. The research team randomly assigned participants to one of two groups: (1) 35 to the intervention group, which received acupuncture using acupoints selected using EITBM, and (2) 35 to the control group, which received acupuncture using the classical consensus acupoints. Both groups performed a 25-min session of acupuncture three times weekly for 4 weeks, with the exercise therapy following the acupuncture each time. Outcome Measures center dot The research team assessed clinical efficacy at baseline and postintervention. The primary outcome measures included assessments: (1) of knee joint pain using a visual analog scale (VAS), (2) of knee joint pain, flexibility, and function using the Western Ontario and McMaster University Osteoarthritis Index (WOMAC) pain subscale; and (3) joint range of motion (ROM). The secondary outcome measures included measurement of serum levels of interleukin-1 beta (IL-1 beta), tumor necrosis factor alpha (TNF-alpha), and matrix metalloproteinase-13 (MMP-13) using enzyme-linked immunosorbent assays (ELISA). Results center dot The VAS and WOMAC scores significantly decreased for both groups between baseline and postintervention, and the intervention group's decrease was significantly greater than that of the control group. The ROM of knee flexion was significantly higher in both groups postintervention than at baseline, and the intervention group's increase was significantly higher than that of the control group. The serum IL-1 beta, TNF-alpha, and MMP-13 also significantly decreased postintervention in both groups, and the intervention group's levels were significantly lower than those of the control group. The total effective rate was 94.1% in the intervention group, 32 out of 34 participants, and 75.8% in the control group, 25 out of 33 participants, which was significantly different. Conclusions center dot Acupuncture, in combination with exercise, can relieve symptoms, improve joint function, and reduce pro-inflammatory cytokines (IL-1 beta and TNF-alpha) as well as MMP-13 for patients with KOA. The outcomes for acupuncture using EITBM acupoints were significantly better than those of the acupoints selected using classical consensus.

C1 Hebei Univ Chinese Med, Dept Acupuncture & Moxibust, Affiliated Hosp 1, Shijiazhuang, Hebei, Peoples R China

ID 278

ER

FN Clarivate Analytics Web of science

PT J

AU Li, J. J.

AU - Zhang, X. L.

AU - Xie, L. J.

AU - Lou, F.

AU - Shen, J. R.

PY 2023

TI Integration of Traditional Chinese Medicine and Western Medicine in the Treatment of Knee Osteoarthritis: Research Progress on the Combination of Acupuncture and Diclofenac Sodium

PG 199-203

DA 2023/1/1

SO INDIAN JOURNAL OF PHARMACEUTICAL SCIENCES

JO INDIAN JOURNAL OF PHARMACEUTICAL SCIENCES

VL 85

SN 0250-474X

Z9 Times Cited in Web of Science Core Collection: 0 Total Times Cited: 0 Cited Reference Count: 20 ER -

N1 LiZhang-319

N1 2025/2/13 1:16:00

DE Knee osteoarthritis; traditional Chinese medicine; western medicine; acupuncture; diclofenac; sodium; steroids; THERAPY

AB Knee osteoarthritis is a common chronic joint disease, and although conventional western medical treatments are effective, they also have certain limitations. Therefore, the integration of traditional Chinese medicine and western medicine has attracted attention as a potential treatment approach. Acupuncture, as a traditional Chinese medicine therapy with a long history, can alleviate pain and improve function by regulating the nervous and immune systems. Diclofenac sodium can reduce inflammation and relieve pain. The combined application of traditional Chinese medicine and western medicine can leverage the complementary advantages of both approaches, achieving better treatment outcomes. Although existing research supports the effectiveness of acupuncture combined with diclofenac sodium in the treatment of knee osteoarthritis, further large-scale and rigorously designed clinical studies are needed to validate its long-term efficacy and safety. The comprehensive evaluation of the combined therapy's effectiveness for knee osteoarthritis includes clinical efficacy, pain relief, functional improvement, enhancement of quality of life and safety. With the continuous progress of scientific research, this therapy is expected to become a more comprehensive and individualized treatment option, improving patients' quality of life and treatment outcomes.

C1 Fuyang Hosp Tradit Chinese Med, Dept Acupuncture, Hangzhou 311400, Zhejiang, Peoples R China; Ningbo Hosp Tradit Chinese Med, Dept Tradit Chinese Med, Ningbo 315000, Zhejiang, Peoples R China

ID 319

ER

FN Clarivate Analytics Web of science

PT J

AU Xu, W. J.

AU - Xiao, Y.

AU - Zhao, M. Z.

AU - Zhu, J. H.

AU - Wang, Y.

AU - Wang, W. B.

AU - Wang, P.

AU - Meng, H.

PY 2023

TI Effective Treatment of Knee Osteoarthritis Using a Nano-Enabled Drug Acupuncture Technology in Mice (Adv. Sci. 28/2023)

DA 2023/1/1

SO ADVANCED SCIENCE

JO ADVANCED SCIENCE

VL 10

IS 28

SN 2198-3844

Z9 Times Cited in Web of Science Core Collection: 2 Total Times Cited: 2 Cited Reference Count: 0 ER -

M3 10.1002/advs.202370188

N1 XuXiao-328

N1 2025/2/13 1:16:00

DI 10.1002/advs.202370188

ID 328

ER

FN Clarivate Analytics Web of science

PT J

AU Ding, L. B.

AU - Wang, H. J.

AU - Li, Y.

AU - Li, J.

AU - Li, L.

AU - Gao, Y. P.

AU - Guan, J.

AU - Geng, W. Q.

PY 2023

TI Electroacupuncture stimulating Neixiyan (EX-LE5) and Dubi (ST35) alleviates osteoarthritis in rats induced by anterior cruciate ligament transaction <i>via</i> affecting DNA methylation regulated transcription of miR-146a and miR-140-5p

PG 983-990

DA 2023/1/1

SO JOURNAL OF TRADITIONAL CHINESE MEDICINE

JO JOURNAL OF TRADITIONAL CHINESE MEDICINE

VL 43

IS 5

SN 0255-2922

Z9 Times Cited in Web of Science Core Collection: 0 Total Times Cited: 0 Cited Reference Count: 41 ER -

M3 10.19852/j.cnki.jtcm.2023.05.004

N1 DingWang-427

Y2 2025/1/17 15:24:00

N1 2025/2/13 1:16:00

DE osteoarthritis; knee; electroacupuncture; DNA methylation; MicroRNAs; KNEE OSTEOARTHRITIS; ACUPUNCTURE; MODEL; EXPRESSION; DAMAGE; PAIN; SHAM

AB OBJECTIVE: To explore whether electroacupuncture (EA) could alleviate osteoarthritis (OA) through affecting the DNA methylation regulated transcription of miR-146a and miR-140-5p. METHODS: Sixty male eight-week-old Sprague-Dawley rats were divided into three groups: normal group (normal healthy rats; no treatment), model group (OA rats; no treatment) and EA group (OA rats treated with EA). Safranin O staining and modified Mankin's score were performed to evaluate the histopathological alterations and degeneration of cartilage 8 weeks after 8 consecutive weeks of treatment. Quantitative real time polymerase chain reaction (qRT-PCR) assay was employed to evaluate the expression of miR-146a in the cartilage tissue and miR-140-5p in the synovium tissue, respectively. The bisulfite sequencing analysis and quantitative methylation specific PCR (qMSP) were used to analyze the status of methylation in the regulatory regions of miR-146a and miR-140-5p. Chromatin immunoprecipitation (ChIP) assay were performed to assess the binding of nuclear factor-kappa B (NF-kappa B) and signal transducer and activator of transcription 3 (SMAD-3) in the regulatory regions of miR-146a and miR-140-5p. Western blot analysis was performed to detect the expressions of DNA Methyltransferase 1 (DMNT1), DNA Methyltransferase 3A (DMNT3A), and DNA Methyltransferase 3A (DMNT3b), NF-kappa B, SMAD3 levels. RESULTS: Our results showed that EA treatment significantly upregulated miR-146a and miR-140-5p expressions. qMSP analysis showed that EA significantly decreased methylation levels of miR-140-5p regulated region and miR-146a promoter in OA cartilage and synovium. Bisulfite DNA sequencing (BDS) and ChIP analysis showed that EA significantly increased binding affinity of SMAD3 and NF-kB on the hypermethylated miR-140 regulatory region and miR-146a promoter, respectively. Western Blot analysis demonstrated that EA also significantly decreased expressions of methylation related proteins- DMNT1, DMNT3a, and DMNT3b as well as NF-kappa B and SMAD3. CONCLUSIONS: Electroacupuncture stimulating Neixiyan (EX-LE5) and Dubi (ST35) may alleviate OA via affecting the DNA methylation regulated transcription of miR-146a and miR-140-5p. (c) 2023 JTCM. All rights reserved.

C1 Third Hosp Shijiazhuang, Dept Orthoped, Shijiazhuang 050011, Peoples R China; Jinan Univ, Affiliated Hosp 1, Dept Orthoped Surg & Sports Med Ctr, Guangzhou 510630, Peoples R China; Guangdong Prov Peoples Hosp, Div Rheumatol, Guangzhou 510080, Peoples R China; Guangdong Acad Med Sci, Guangzhou 510080, Peoples R China; Southern Med Univ, Dept TCM Orthoped & Traumatol, Affiliated Hosp 3, Guangzhou 510630, Peoples R China; Bethune Int Hosp, Hosp 980, Dept Orthoped Surg, Joint Logist Support Force Peoples Liberat Army P, Handan Campus, Handan 050082, Peoples R China

DI 10.19852/j.cnki.jtcm.2023.05.004

ID 427

ER

FN Clarivate Analytics Web of science

PT J

AU Yu, C.

AU - Zhang, R.

AU - Shen, B.

AU - Li, X.

AU - Fang, Y. Y.

AU - Jiang, Y. Y.

AU - Jian, G. F.

PY 2023

TI Effects of sham acupuncture for chronic musculoskeletal pain syndrome: A systematic review and network meta-analysis of randomized controlled trials

DA 2023/1/1

SO MEDICINE

JO MEDICINE

VL 102

IS 46

SN 0025-7974

Z9 Times Cited in Web of Science Core Collection: 1 Total Times Cited: 1 Cited Reference Count: 83 ER -

M3 10.1097/MD.0000000000035275

N1 YuZhang-475

Y2 2025/1/17 14:37:00

N1 2025/2/13 1:16:00

DE chronic musculoskeletal pain; randomized controlled trials; sham acupuncture; systematic review; TENSION-TYPE HEADACHE; LOW-BACK-PAIN; KNEE OSTEOARTHRITIS; PHARMACOLOGICAL-TREATMENT; MYOFASCIAL PAIN; CHRONIC NECK; PLACEBO; THERAPY; FIBROMYALGIA; PREVALENCE

AB Background:Acupuncture has been widely used for chronic musculoskeletal pain syndrome (MPS). Due to the strong influence of sham acupuncture (SA) in clinical trials, the treatment of MPS by acupuncture remains controversial. Different types of SA procedures might produce different responses. The purpose of this systematic review was to assess the effect of SA on MPS.Methods:We searched 8 literature databases for randomized controlled trials (RCTs) on acupuncture for chronic MPS with SA as a control from database inception to November 29, 2022. SA included superficial acupuncture on non-acupoints (SANAs), non-penetration on acupoints (NPAs), and non-penetration on non-acupoints (NPNAs). Two independent reviewers assessed the risk of bias and conducted the research selection, data extraction, and quality assessment of the included RCTs. We conducted data analysis using the RevMan 5.3 and STATA 14 software packages, and traditional meta-analysis was adopted for direct comparison. A network meta-analysis (NMA) was executed using frequency models in which we combined all available direct and indirect evidence from RCTs. The pain-related indicators were set as primary outcomes, and GRADEpro online was implemented for the assessment of evidence quality.Results:Forty-two RCTs were included in this study, encompassing a total of 6876 patients and incorporating 3 types of SA procedures. In our traditional meta-analysis, true acupuncture (TA) was more effective than SANAs, NPAs, and NPANAs concerning MPS. In the NMA, TA was the most effective modality, followed by SANAs, NPAs and NPANAs, and then the blank control (BC). In this NMA and according to the therapeutic effects in the pain indicators, the rankings of SA were as follows: SANA (surface under the cumulative ranking curve [SUCRA], 65.3%), NPA (SUCRA, 46.2%), and NPANA (SUCRA, 34.2%). The quality of the evidence for outcomes ranged from "low" to "moderate."Conclusions:Compared with SA, TA was effective in treating MPS. The effects produced by different SA procedures were different, and the order of effects from greatest to least was as follows: SANA, NPA, and NPANA.

C1 Capital Med Univ, Beijing Hosp Tradit Chinese Med, Dept Acupuncture, 23 Meishuguanhou St, Beijing 100010, Peoples R China; Beijng Tradit Chinese Med Hosp, Pinggu Hosp, Dept Acupuncture, Beijing, Peoples R China

DI 10.1097/MD.0000000000035275

ID 475

ER

FN Clarivate Analytics Web of science

PT J

AU Jun, J. H.

AU - Choi, T. Y.

AU - Park, S.

AU - Lee, M. S.

PY 2023

TI Warm needle acupuncture for osteoarthritis: An overview of systematic reviews and meta-analysis

DA 2023/1/1

SO FRONTIERS IN MEDICINE

JO FRONTIERS IN MEDICINE

VL 10

SN 2296-858X

Z9 Times Cited in Web of Science Core Collection: 2 Total Times Cited: 2 Cited Reference Count: 40 ER -

M3 10.3389/fmed.2023.971147

N1 JunChoi-477

N1 2025/2/13 1:16:00

DE acupuncture; moxibustion; warm needle acupuncture; osteoarthritis; overview; systematic review; KNEE OSTEOARTHRITIS

AB Background: Osteoarthritis (OA) is a chronic disease that is a major cause of pain and functional disability. Warm needle acupuncture (WA) therapy has been widely used to treat OA. This overview summarizes the evidence from systematic reviews (SRs) and assesses the methodological quality of previous SRs that evaluated the use of WA therapy for OA.Methods: We searched electronic databases to identify SRs that evaluated the efficacy of WA therapy for OA. Two reviewers independently extracted data and assessed the methodological quality of the reviews according to the A Measurement Tool to Assess Systematic Reviews (AMSTAR 2) tool. The reporting quality was assessed using the Preferred Reporting Items for Systematic Reviews and Meta-Analysis 2020 (PRISMA 2020) guidelines. The quality of evidence was assessed according to the Grading of Recommendations Assessment, Development, and Evaluation (GRADE) approach.Results: Fifteen SRs were included in this study. WA therapy was more effective than control conditions for the treatment of OA. The results of the AMSTAR 2 tool showed that the methodological quality of all included studies was critically low. The items with the lowest scores were item 2 (reporting the protocol), item 7 (listing excluded studies and justifying the exclusions), and item 16 (including conflicts of interest). Regarding the PRISMA guidelines, 2 SRs exhibited greater than 85% compliance. The overall quality of evidence in the included SRs ranged from "very low" to "moderate."Conclusion: This overview shows that WA therapy was more effective than the control treatment for OA. However, the methodological quality of the reviews was low, indicating the need for improvements in the collection of evidence. Future studies are needed to collect high-quality evidence regarding the use of WA for OA.Systematic review registration, Research Registry (reviewregistry1317).

C1 Korea Inst Oriental Med, KM Sci Res Div, Daejeon, South Korea; Daejeon Univ, Coll Korean Med, Dept Prevent Med, Daejeon, South Korea

DI 10.3389/fmed.2023.971147

ID 477

ER

FN Clarivate Analytics Web of science

PT J

AU Chiu, P. E.

AU - Fu, Z. H.

AU - Sun, J.

AU - Jian, G. W.

AU - Li, T. M.

AU - Chou, L. W.

PY 2023

TI Fu's Subcutaneous Needling for Knee Osteoarthritis Pain

DA 2023/1/1

SO JOVE-JOURNAL OF VISUALIZED EXPERIMENTS

JO JOVE-JOURNAL OF VISUALIZED EXPERIMENTS

IS 193

SN 1940-087X

Z9 Times Cited in Web of Science Core Collection: 1 Total Times Cited: 1 Cited Reference Count: 33 ER -

M3 10.3791/65299

N1 ChiuFu-511

Y2 2025/1/18 22:05:00

N1 2025/2/13 1:16:00

DE EPIDEMIOLOGY; BURDEN; HIP

AB Fu's subcutaneous needling (FSN) is a new acupuncture and dry needling technique based on traditional Chinese medicine. It rapidly produces long-lasting effects in soft tissue injuries, particularly in painful musculoskeletal conditions, by providing stimulation primarily in the subcutaneous area. Osteoarthritis (OA) is the most common joint disease in adults worldwide and is often accompanied by a painful syndrome of structural changes in the peripheral joints of the knee. However, the etiology of OA pain is not fully understood, though myofascial trigger points (MTrPs) are commonly found in the lower limb muscles (so-called "tightened muscles") of patients with knee OA. FSN has been used in many fields for the treatment of acute pain problems and can relieve muscle contraction from MTrPs, thereby improving the local circulation. This study recruited patients with pain from knee OA into an FSN group or a transcutaneous electrical nerve stimulation (TENS) group with three treatment sessions and a followup over the course of 2 weeks. The results showed that FSN was effective in treating soft tissue pain around the knee with OA. This study aimed to establish and visualize three key technical indicators during FSN therapy, including the FSN needle insertion point and layer; the frequency and duration of the swaying movement; and the manipulation of the reperfusion approach. These findings have great potential for future applications in myofascial pain treatment, especially for pain management. Following this protocol could enhance FSN skills.

C1 Chang Bing Show Chwan Mem Hosp, Dept Chinese Med, Lukang, Taiwan; China Med Univ, Grad Inst Integrated Med, Coll Chinese Med, Shenyang, Peoples R China; Beijing Univ Chinese Med, Inst Fus Subcutaneous Needling, Beijing, Peoples R China; Guangzhou Univ Chinese Med, Clin Med Coll Acupuncture & Moxibust & Rehabil, Guangzhou, Peoples R China; Guangzhou Univ Chinese Med, Clin Med Coll 2, Guangzhou, Peoples R China; Guangzhou Univ Chinese Med, Affiliated Hosp 2, Guangdong Prov Hosp Chinese Med, Guangzhou, Peoples R China; China Med Univ, Grad Inst Acupuncture Sci, Shenyang, Peoples R China; Minist Hlth & Welf, Sinying Hosp, Dept Chinese Med, Taipei, Taiwan; China Med Univ, Coll Chinese Med, Sch Chinese Med, Shenyang, Peoples R China; China Med Univ Hosp, Dept Phys Med & Rehabil, Taichung, Taiwan; China Med Univ, Dept Phys Therapy, Shenyang, Peoples R China; China Med Univ, Grad Inst Rehabil Sci, Shenyang, Peoples R China; Asia Univ, Asia Univ Hosp, Dept Phys Med & Rehabil, Taichung, Taiwan

DI 10.3791/65299

ID 511

ER

FN Clarivate Analytics Web of science

PT J

AU Chang, Y. N.

AU - Wu, N.

AU - Zhang, Z. H.

AU - Zhang, Z. Y.

AU - Ren, B. B.

AU - Liu, F. L.

AU - Song, X. L.

AU - Wu, M. L.

AU - Feng, X. D.

AU - Yin, S.

PY 2022

TI Efficacy of manual acupuncture, electro-acupuncture, and warm acupuncture for knee osteoarthritis: study protocol for a randomized controlled trial

DA 2022/1/1

SO TRIALS

JO TRIALS

VL 23

IS 1

SN 1745-6215

Z9 Times Cited in Web of Science Core Collection: 2 Total Times Cited: 2 Cited Reference Count: 36 ER -

M3 10.1186/s13063-022-06653-7

N1 ChangWu-1

Y2 2025/1/20 13:38:00

N1 2025/2/13 1:16:00

DE Knee osteoarthritis; Manual acupuncture; Electro-acupuncture; Warm acupuncture; Randomized controlled trial; PREVALENCE; GUIDELINE; THERAPY; IMPACT; PAIN; HIP

AB Background Acupuncture is one of the most popular complementary and alternative treatments for knee osteoarthritis (KOA). There are many methods of acupuncture in the treatment of KOA, and the effects are different. According to our clinical observations and researches, it is found that manual acupuncture (MA), electro-acupuncture (EA), and warm acupuncture (WA) are used more frequently in the treatment of KOA, and the curative effects are satisfactory. However, there is currently a lack of efficacy comparison of efficacy between different acupuncture treatments, as well as a lack of standardized clinical research on the acupuncture treatment of KOA. Therefore, we will carry out a high-quality clinical randomized controlled trial to research the effect laws of MA, EA, and WA on KOA. Methods/design A total of 200 eligible participants with KOA will be randomly assigned to group A, B, C, or D in a ratio of 1:1:1:1. Patients in group A will receive MA, while those in group B, group C, and group D will be treated with EA, WA, and sham acupuncture (SA), respectively. Patients will be treated with acupuncture once a day, 30 min per session, 5 sessions per week for 4 weeks. The primary outcome is the change of Western Ontario and McMaster Universities Osteoarthritis Index (WOMAC) at week 4. The secondary outcomes include WOMAC, visual analog scale (VAS), Arthritis Quality of Life Measurement Scale Simplified Scale (AIMS2-SF), Beck Anxiety Inventory (BAI), Beck Depression Inventory (BDI), and Credibility/Expectancy Questionnaire. The evaluation will be performed at baseline and weeks 4, 8, and 12 respectively after randomization. Discussion This is a randomized controlled trial. We will observe the clinical effect of MA, EA, and WA on KOA to research the effect laws of these three acupuncture treatments on KOA and set up standardized treatment programs for acupuncture for KOA.

C1 Henan Univ Chinese Med, 156 East Jinshui Rd, Zhengzhou, Henan, Peoples R China; Henan Univ Chinese Med, Affiliated Hosp 1, Dept Rehabil, 19 Renmin Rd, Zhengzhou, Henan, Peoples R China

DI 10.1186/s13063-022-06653-7

ID 1

ER

FN Clarivate Analytics Web of science

PT J

AU Tan, Q.

AU - Cai, Z. K.

AU - Li, J.

AU - Li, J.

AU - Xiang, H. C.

AU - Li, B. C.

AU - Cai, G. W.

PY 2022

TI Imaging Study on Acupuncture Inhibiting Inflammation and Bone Destruction in Knee Osteoarthritis Induced by Monosodium Iodoacetate in Rat Model

PG 93-103

DA 2022/1/1

SO JOURNAL OF PAIN RESEARCH

JO JOURNAL OF PAIN RESEARCH

VL 15

SN 1178-7090

Z9 Times Cited in Web of Science Core Collection: 6 Total Times Cited: 7 Cited Reference Count: 49 ER -

M3 10.2147/JPR.S346242

N1 TanCai-8

N1 2025/2/13 1:16:00

DE osteoarthritis; acupuncture; PET; micro-CT; inflammation; bone destruction; FDG-PET/CT; HIP

AB Objective: We aim to explore whether acupuncture inhibits inflammation and bone destruction in rat model monosodium iodoacetate (MIA)-induced knee osteoarthritis (KOA) by 18Ffluorodeoxyglucose (18F-FDG) small-animal positron emission tomography (PET) and micro-computed tomography (CT) imaging. Methods: KOA was induced in rats by intra-articular injection MIA (2 mg/50 mu L) through the right knee of the rats. Forty male Sprague Dawley rats weighing 280 to 340 g (12 weeks old) were randomly divided into four groups including Control group, KOA group, KOA plus manual acupuncture group (KOA+MA), KOA plus sham acupuncture group (KOA +SA). The acupuncture treatment lasted for three weeks (one-day rest after six days of treatment). Paw withdrawal threshold test and open-field test were used to assess mechanical allodynia and locomotor activity respectively for once a week. Hematoxylin and eosin (H&E) staining was used to assess the damage of the cartilage, synovium and infrapatellar fat pad (IFP). 18F-FDG PET was performed to quantify joint inflammation. The influence on the subchondral bone in these rats was confirmed by micro-CT. Results: Mechanical hyperalgesia, joint inflammation, and obvious bone destruction were observed in the KOA group. H&E staining of the knee joint found that manual acupuncture played a protective effect in cartilage, synovium and IFP destruction. However, compared with KOA group, the results in sham acupuncture had no significant difference. After manual acupuncture treatment in KOA rats, inflammation was significantly suppressed shown by 18F-FDG PET imaging. Micro-CT analysis of the knee joint revealed that manual acupuncture protected bone by inhibiting osteophyte development and subchondral bone remodeling. Conclusion: The results of 18F-FDG PET and micro-CT showed that manual acupuncture inhibited inflammation and bone destruction, which provides reliable evidence for the effectiveness of acupuncture in hindering development of KOA, and provides reliable evidence for clinical application of acupuncture.

C1 Huazhong Univ Sci & Technol, Union Hosp, Tongji Med Coll, Dept Acupuncture, Wuhan, Peoples R China; Huazhong Univ Sci & Technol, Dept Publ Adm, Wuhan, Peoples R China; Hubei Univ Chinese Med, Coll Acupuncture & Orthoped, Wuhan, Peoples R China

DI 10.2147/JPR.S346242

ID 8

ER

FN Clarivate Analytics Web of science

PT J

AU Wei, J. Y.

AU - Liu, L.

AU - Li, Z. J.

AU - Lyu, T. L.

AU - Zhao, L. P.

AU - Xu, X. B.

AU - Song, Y. N.

AU - Dai, Y. D.

AU - Li, B.

PY 2022

TI Fire Needling Acupuncture Suppresses Cartilage Damage by Mediating Macrophage Polarization in Mice with Knee Osteoarthritis

PG 1071-1082

DA 2022/1/1

SO JOURNAL OF PAIN RESEARCH

JO JOURNAL OF PAIN RESEARCH

VL 15

SN 1178-7090

Z9 Times Cited in Web of Science Core Collection: 10 Total Times Cited: 11 Cited Reference Count: 68 ER -

M3 10.2147/JPR.S360555

N1 WeiLiu-9

N1 2025/2/13 1:16:00

DE fire needling acupuncture; knee osteoarthritis; synovial membrane; cartilage; macrophage polarization; LOW-GRADE; INFLAMMATION; PAIN; MOXIBUSTION; EXPRESSION; SYNOVITIS; CYTOKINES; THERAPY; CELLS

AB Purpose: Macrophage polarization contributes to the mechanisms of treating knee osteoarthritis (KOA). In previous studies, fire needling acupuncture has been shown to affect KOA favorably. However, the mechanism of fire needling acupuncture on macrophage polarization is not well-defined. Thus, this study was conducted to determine that fire needling acupuncture exerts a therapeutic role in KOA by modulating macrophage polarization. Methods: Thirty mice were allocated at random into three groups of ten. The groups were labeled as "control", "model", and "fire needling acupuncture". Each group consisted of ten mice. From the second day of intra-articular injection MIA, the right "xiyan" (EX-LE5), "dubi" (ST35), "liangqiu" (ST34), and "xuehai" (SP10) acupoints were manipulated once every other day for two weeks in the fire needling acupuncture group. Mechanical withdrawal threshold and weight distribution were evaluated for behavioral testing in each group. The synovial morphology was monitored by HE staining. Pathological morphology was observed by HE staining, Saf-O staining, and toluidine blue staining. The polarization of macrophages in synovial tissue was detected using immunofluorescence (F4/80, CD86, and CD206). Results: Fire needling acupuncture increased the percentage weight-bearing difference and the mechanical withdrawal threshold, and improved synovial inflammation and cartilage damage in MIA-induced KOA mice. F4/80 and CD86 expression were downregulated by fire needling acupuncture, but CD206 was increased. Conclusion: Fire needling acupuncture decreases pain behaviors in KOA mice and improves synovial membrane injury and pathological cartilage damage. The macrophage polarization is involved in the mechanism of fire needling acupuncture's amelioration of articular cartilage damage.

C1 Capital Med Univ, Beijing Hosp Tradit Chinese Med, Dept Acupuncture & Moxibust, Beijing Key Lab Acupuncture Neuromodulat, 23 Meishuguan Back St, Beijing 100010, Peoples R China; Capital Med Univ, Beijing Hosp Tradit Chinese Med, Beijing Inst Tradit Chinese Med, Beijing, Peoples R China

DI 10.2147/JPR.S360555

ID 9

ER

FN Clarivate Analytics Web of science

PT J

AU Wang, X. Y.

AU - Nie, Z. Y.

AU - Yu, Q. Q.

AU - Chen, W.

AU - Zhang, X. N.

AU - Hong, W. Y.

AU - Su, Y. S.

AU - He, W.

AU - Li, R. Y.

AU - Jing, X. H.

PY 2022

TI Acupuncture Enhances Signals at Sensitized Acupoints to Elevate Pressure Pain Threshold in Knee Osteoarthritis Patients

PG 1105-1110

DA 2022/1/1

SO CHINESE JOURNAL OF INTEGRATIVE MEDICINE

JO CHINESE JOURNAL OF INTEGRATIVE MEDICINE

VL 28

IS 12

SN 1672-0415

Z9 Times Cited in Web of Science Core Collection: 4 Total Times Cited: 4 Cited Reference Count: 31 ER -

M3 10.1007/s11655-022-3588-6

N1 WangNie-10

N1 2025/2/13 1:16:00

DE sensitized acupoint; pain; manual acupuncture; skin conductance; blood perfusion; SKIN BLOOD-FLOW; ELECTROACUPUNCTURE STIMULATION; INJECTIONS

AB Objective To observe the pressure pain threshold (PPT), skin conductance (SC) and blood perfusion (BP) of the sensitized acupoints in patients with knee osteoarthritis (KOA), and explore the mechanism of acupuncture at the sensitized acupoints for treating diseases. Methods Eleven healthy subjects and 11 unilateral KOA patients were recruited from July 2020 to March 2021 in this study. The PPT, SC and BP of control acupoints in healthy controls, and non-sensitized and sensitized acupoints in KOA patients were measured and compared between baseline and after manual acupuncture (MA) treatment. Results Before MA treatment, lower PPT was observed at the sensitized acupoints compared with non-sensitized and control acupoints (P<0.05). After MA treatment, PPT at the sensitized acupoints increased significantly in KOA patients (P<0.05). Before MA treatment, there was no statistical difference in SC and BP among control, non-sensitized and sensitized acupoints (P>0.05). Compared with the control and non-sensitized acupoints, there were significant increases of SC and BP in sensitized acupoints of KOA patients after MA treatment (P<0.05 or P<0.01). Conclusion MA at sensitized acupoints could elevate PPT of KOA patients, which may be associated with the increment of SC and BP.

C1 China Acad Chinese Med Sci, Inst Acupuncture & Moxibust, Beijing 100700, Peoples R China; Beihang Univ, Beijing Adv Innovat Ctr Biomed Engn, Sch Biol Sci & Med Engn, Key Lab Biomech & Mechanobiol,Minist Educ, Beijing 100083, Peoples R China

DI 10.1007/s11655-022-3588-6

ID 10

ER

FN Clarivate Analytics Web of science

PT J

AU Qin, L. X.

AU - Guo, C. Q.

AU - Zhao, R. L.

AU - Wang, T.

AU - Wang, J. M.

AU - Guo, Y.

AU - Zhang, W.

AU - Hu, T. Y.

AU - Chen, X. L.

AU - Zhang, Q.

AU - Zhang, D.

AU - Xu, Y.

PY 2022

TI Acupotomy inhibits aberrant formation of subchondral bone through regulating osteoprotegerin/receptor activator of nuclear factor-KB ligand pathway in rabbits with knee osteoarthritis induced by modified Videman method

PG 389-399

DA 2022/1/1

SO JOURNAL OF TRADITIONAL CHINESE MEDICINE

JO JOURNAL OF TRADITIONAL CHINESE MEDICINE

VL 42

IS 3

SN 0255-2922

Z9 Times Cited in Web of Science Core Collection: 6 Total Times Cited: 6 Cited Reference Count: 57 ER -

M3 10.19852/j.cnki.jtcm.2022.03.006

N1 QinGuo-27

Y2 2025/1/19 22:09:00

N1 2025/2/13 1:16:00

DE acupuncture therapy; osteogenesis; osteoarthritis; knee; subchondral bone; ATTENUATES OSTEOARTHRITIS; CARTILAGE DEGENERATION; COLLAGEN; METAANALYSIS; EXPRESSION

AB OBJECTIVE: To investigate the effects of acupotomy on inhibiting abnormal formation of subchondral bone in rabbits with knee osteoarthritis (KOA). METHODS: A total of 24 New Zealand rabbits were randomly divided into four groups of 6 rabbits each [control, model, electroacupuncture (EA) and acupotomy]. Eighteen KOA model rabbits were established using a modified Videman method. Rabbits in EA and acupotomy groups received the intervention for 3 weeks. Then, the cartilage and subchondral bone unit were obtained and the histomorphological changes were recorded. Osteoprotegerin (OPG) and receptor activator of nuclear factor KB ligand (RANKL) in subchondral bone were evaluated by Western blotting, real-time polymerase chain reaction and immunohistochemistry. RESULTS: Compared with the model group, both the acupotomy and EA groups showed a significant decrease in the Lequesne index (both P < 0.01) and Mankin score (P < 0.01, < 0.05). In addition, both EA and acupotomy groups had a higher expression of total articular cartilage (TAC) (P < 0.05, < 0.01) and lower expression of articular calcified cartilage (ACC)/TAC (P < 0.05, < 0.05) compared with the model group. The thickness of the subchondral bone plate in EA and acupotomy groups were decreased (both P < 0.01) compared to the model group. Moreover, trabecular bone volume (BV/TV), protein and relative expression of OPG and the ratio of OPG/RANKL in the subchondral bone of acupotomy group were decreased statistically significant, while these parameters were not significantly changed in the EA group compared with the model group. CONCLUSIONS: In the rabbit model of KOA, acupotomy inhibits aberrant formation of subchondral bone by suppressing OPG/RANKL ratio as a potential therapy for KOA. (c) 2022 JTCM. All reserved.

C1 Beijing Univ Chinese Med, Sch Acupuncture Moxibust & Tuina, Beijing 100029, Peoples R China; First Peoples Hosp Dongcheng Dist, Beijing 100050, Peoples R China; Capital Med Univ, Beijing Hosp Tradit Chinese Med, Beijing 100010, Peoples R China; Beijing Universal Chinese Med, Affiliated Hosp 3, Beijing 100029, Peoples R China

DI 10.19852/j.cnki.jtcm.2022.03.006

ID 27

ER

FN Clarivate Analytics Web of science

PT J

AU Chai, Y. P.

AU - Han, X. Q.

AU - Cui, J. Z.

PY 2022

TI Acupotomy combined with intra-articular injection of sodium hyaluronate in the treatment of knee osteoarthritis

DA 2022/1/1

SO MEDICINE

JO MEDICINE

VL 101

IS 35

SN 0025-7974

Z9 Times Cited in Web of Science Core Collection: 0 Total Times Cited: 0 Cited Reference Count: 29 ER -

M3 10.1097/MD.0000000000030225

N1 ChaiHan-37

Y2 2025/1/17 18:01:00

N1 2025/2/13 1:16:00

DE acupotomy; efficacy; intra-articular injection; knee osteoarthritis; sodium hyaluronate; METAANALYSIS; ACUPUNCTURE; THERAPY; PAIN; HIP

AB This retrospective study aimed to compare the effects of acupotomy combined with intra-articular injection of sodium hyaluronate (IA-SH) for the treatment of knee osteoarthritis (KOA). Eighty electronic medical records of patients with KOA were retrospectively analyzed. The patients were divided into an intervention group (n = 40, acupotomy plus IA-SH) and a control group (n = 40, IA-SH). Outcome measures included the visual analog scale, the Western Ontario and McMaster Universities Arthritis Index (WOMAC), and adverse events. Outcome data were collected and analyzed before and after treatment. The results of this study showed that there was a greater reduction in the visual analog scale (P < .01) and WOMAC scores (pain, P < .01; stiffness, P < .01; function, P < .01; total, P < .01) in the intervention group than in the control group. In addition, there were no significant differences in adverse events between the 2 groups. In this study, the effects of acupotomy plus IA-SH were superior to those of IA-SH alone for the treatment of patients with KOA. Further prospective studies are required to confirm these findings.

C1 Ankang Hosp Tradit Chinese Med, Orthoped Dept, Ward 2, Ankang, Shaanxi, Peoples R China; Ankang Hosp Tradit Chinese Med, High Tech Branch, Dept Orthoped, Cross Wenfing Rd East & Chuangxin Rd South, Ankang 725000, Shaanxi, Peoples R China

DI 10.1097/MD.0000000000030225

ID 37

ER

FN Clarivate Analytics Web of science

PT J

AU Sun, Z. W.

AU - Qu, X. L.

AU - Wang, T. M.

AU - Liu, F.

AU - Li, X.

PY 2022

TI Effects of Warm Acupuncture Combined with Meloxicam and Comprehensive Nursing on Pain Improvement and Joint Function in Patients with Knee Osteoarthritis

DA 2022/1/1

SO JOURNAL OF HEALTHCARE ENGINEERING

JO JOURNAL OF HEALTHCARE ENGINEERING

VL 2022

SN 2040-2295

Z9 Times Cited in Web of Science Core Collection: 7 Total Times Cited: 9 Cited Reference Count: 26 ER -

M3 10.1155/2022/9167956

N1 SunQu-62

N1 2025/2/13 1:16:00

DE RESVERATROL; THERAPY

AB Objective. To observe the effect of warm acupuncture combined with meloxicam and comprehensive nursing on pain improvement and joint function in patients with knee osteoarthritis. Method. Eighty-one patients with KOA were randomly divided into control group (CG), traditional Chinese medicine group (TCMG), and combined group (JG). The CG was treated with meloxicam. The TCMG received warm acupuncture treatment. The JG was treated with meloxicam combined with warm acupuncture. Three groups were given comprehensive nursing intervention, and the course of treatment was 4 weeks. Knee function was assessed by knee pain, activity, stability, walking ability, and ability to walk up and down stairs. Improvement time of clinical symptoms of patients was assessed from knee pain, swelling, and movement limitation. Pain mediators (prostaglandin E2 (PGE2), substance P (SP), dopamine (DA), 5-hydroxytryptamine (5-HT)) were detected by enzyme-linked immunosorbent assay (ELISA). Oxidative stress indicators (superoxide dismutase (SOD) and malondialdehyde (MDA)) of the enrolled patients were detected by water-soluble tetrazolium-1 (WST-1) and the thiobarbituric acid (TBA) method. The clinical efficacy was assessed by the visual analog scale (VAS) score. Results. After treatment, the pain scores of the three groups decreased, and the scores of mobility, stability, walking ability, and the ability to walk up and down stairs increased. Compared with the CG and the TCMG, the JG had a greater range of changes in pain, mobility, stability, walking ability, and ability to walk up and down stairs after treatment. After 7 d, 14 d, and 28 d treatment, PGE2, SP, DA, 5-HT, and MDA in the three groups were decreased compared with before treatment, and the decrease in the JG was more obvious than that in the CG and the TCMG. SOD levels in the three groups were increased, and the increase in the JG was more obvious than that in the CG and the TCMG. The total effective rate of the JG (96.30%) was significantly different from that of the CG (77.78%) and the TCMG (81.48%). The improvement time of knee pain, swelling, and movement limitation in the JG was shorter than that in the CG and the TCMG, and the difference in the improvement time of movement limitation in the TCMG was statistically significant. Conclusion. Warm acupuncture combined with meloxicam and comprehensive nursing can effectively improve knee swelling and pain in patients with KOA, and the mechanism may be related to reducing the content of inflammatory mediators.

C1 Yantatshan Hosp, Dept Traumatol 1, Yantai 264000, Peoples R China; Qingdao Eighth Peoples Hosp, Dept Emergency Med, Qingdao 266000, Peoples R China; Qingdao Univ, Med Coll, Affiliated Hosp 2, Dept Tradit Chinese Med,Affiliated Qingdao Cent H, Qingdao 266042, Peoples R China; Zhangqiu Dist Peoples Hosp, Dept Spine Surg, Jinan 250200, Peoples R China; Zhangqiu Dist Peoples Hosp, Dept Spine & Joint, Jinan 250200, Peoples R China

DI 10.1155/2022/9167956

ID 62

ER

FN Clarivate Analytics Web of science

PT J

AU Liu, J.

AU - Zeng, W. Q.

AU - Lin, Q. X.

AU - Dai, R. Q.

AU - Lu, L. M.

AU - Guo, Z. X.

AU - Lian, X. W.

AU - Pan, X. G.

AU - Liu, H.

AU - Xiu, Z. B.

PY 2022

TI Proteomic Analyses Reveals the Mechanism of Acupotomy Intervention on the Treatment of Knee Osteoarthritis in Rabbits

DA 2022/1/1

SO EVIDENCE-BASED COMPLEMENTARY AND ALTERNATIVE MEDICINE

JO EVIDENCE-BASED COMPLEMENTARY AND ALTERNATIVE MEDICINE

VL 2022

SN 1741-427X

Z9 Times Cited in Web of Science Core Collection: 2 Total Times Cited: 2 Cited Reference Count: 36 ER -

M3 10.1155/2022/5698387

N1 LiuZeng-68

N1 2025/2/13 1:16:00

DE CARTILAGE; METABOLISM; CELLS

AB Acupotomy intervention (AI) is an available treatment for knee osteoarthritis (KOA) in China, which is a common health problem over the world. However, the underlying mechanism of AI on the KOA treatment is still unknown. To further understand the mechanism of acupotomy in treating KOA, the morphological observation and TMT proteomic analyses were conducted in rabbits. By using X-ray and MRI, we found that the space of the knee joint was bigger in AI than in KOA. Moreover, the chondrocytes were neatly arranged in AI but disordered in KOA. With proteomic analyses in chondrocytes, 68 differently accumulated proteins (DAPs) were identified in AI vs. KOA and DAPs related to energy metabolism and the TCA cycle were suggested to play a central role in response to AI. Furthermore, AIFM1 was proposed to be an important regulator in controlling the energy production in mitochondrial. Besides, FN1, VIM, COL12A1, COL14A1, MYBPH, and DPYSL3 were suggested to play crucial roles in AI for the treatment of KOA. Our study was systematically elucidating the regulation mechanism of acupotomy intervention in the treatment of KOA.

C1 Fujian Univ Tradit Chinese Med, Affiliated Peoples Hosp, Fuzhou 350004, Peoples R China; Fujian Univ Tradit Chinese Med, Rehabil Hosp, Fuzhou 350003, Peoples R China; Third Peoples Hosp Fujian Prov, Fuzhou 350122, Peoples R China; Fujian Univ Tradit Chinese Med, Fuzhou 350122, Peoples R China; Fujian Univ TCM, Key Lab Orthoped & Traumatol Tradit Chinese Med &, Minist Educ, Fuzhou 350122, Peoples R China; Fujian Inst Orthopaed, Fuzhou, Fujian, Peoples R China

DI 10.1155/2022/5698387

ID 68

ER

FN Clarivate Analytics Web of science

PT J

AU Wang, Y.

AU - Lu, Q.

AU - Guo, H. Z.

AU - Sun, J. B.

AU - Li, X. M.

AU - Guan, H. Y.

AU - Gao, Y.

AU - Song, B. L.

PY 2022

TI Acupuncture combined with traditional Chinese medicine for knee osteoarthritis: A protocol for systematic review and meta-analysis

DA 2022/1/1

SO MEDICINE

JO MEDICINE

VL 101

IS 50

SN 0025-7974

Z9 Times Cited in Web of Science Core Collection: 0 Total Times Cited: 0 Cited Reference Count: 22 ER -

M3 10.1097/MD.0000000000031820

N1 WangLu-78

Y2 2025/1/17 16:47:00

N1 2025/2/13 1:16:00

DE acupuncture; knee osteoarthritis; meta-analysis; protocol; systematic review; traditional Chinese medicine; NONSTEROIDAL ANTIINFLAMMATORY DRUGS; HIP OSTEOARTHRITIS; AMERICAN-COLLEGE; MANAGEMENT; HAND; PAIN

AB Background: With the aging of society, the incidence of knee osteoarthritis (KOA) is increasing year by year, which seriously affects the quality of life. Acupuncture therapy has been widely used in the treatment of osteoarthritis, but there is no complete systematic review on acupuncture combined with traditional Chinese medicine. Therefore, this study aims to clarify the efficacy and safety of acupuncture combined with traditional Chinese medicine in the treatment of KOA through systematic review and meta-analysis. Methods: A structured and systematic literature search will be conducted in the following databases up to March, 20, 2022: PubMed, Embase, Cochrane Central Register of Controlled Trials, Web of Science, China National Knowledge Infrastructure, Chinese Biomedical Literature Database, Chinese Scientific and Journal Database, Wan Fang database and 2 clinical trials register platforms: Chinese Clinical Trial Registry, ClinicalTrials.gov (). We will use the Review Manager 5.4 software provided by the Cochrane Collaborative Network for statistical analysis. We then assessed the quality and risk of the included studies and observed the outcome measures. Results: This meta-analysis further established the efficacy of acupuncture combined with traditional Chinese medicine in the treatment of KOA. Conclusion: This meta-analysis aims to investigate the efficacy of acupuncture combined with traditional Chinese medicine on patients with KOA and provide reliable evidence. To provide more options for clinicians and patients in the treatment of KOA.

C1 Changchun Univ Chinese Med, Dept Acupuncture & Tuina, Changchun, Peoples R China; Sch Nursing Beihua Univ, Jilin, Peoples R China

DI 10.1097/MD.0000000000031820

ID 78

ER

FN Clarivate Analytics Web of science

PT J

AU Huang, H.

AU - Liang, Y. Y.

AU - Han, D. P.

AU - Chen, X. Y.

AU - Xiao, L. B.

AU - Wu, H. Y.

PY 2022

TI Case report: Electroacupuncture for acute pain flare-up of knee osteoarthritis

DA 2022/1/1

SO FRONTIERS IN NEUROLOGY

JO FRONTIERS IN NEUROLOGY

VL 13

SN 1664-2295

Z9 Times Cited in Web of Science Core Collection: 3 Total Times Cited: 3 Cited Reference Count: 30 ER -

M3 10.3389/fneur.2022.1026441

N1 HuangLiang-79

N1 2025/2/13 1:16:00

DE electroacupuncture; acute pain flare-up; knee; osteoarthritis; case report; RECOMMENDATIONS; ACUPUNCTURE; MANAGEMENT; HIP

AB Acute pain flare-up of knee osteoarthritis (KOA) is a common disease in orthopedics and is mainly treated with analgesic drugs. Patients usually refuse to take western medicines orally owing to gastrointestinal side effects or unsatisfactory treatment results. We report the case of a 69-year-old woman who had an acute pain flare-up of right KOA induced by long-distance walking. As the patient refused medication, we used electroacupuncture (EA) to relieve her symptoms. EA with a 2-Hz frequency and a 1-2-mA intensity had an analgesic effect on the acute pain flare-up of KOA. After 12 weeks of EA intervention, the bone marrow edema-like lesions (BMLs) improved significantly, as depicted on magnetic resonance imaging of the knee joint. However, more powerful evidence is needed to understand the mechanism of the EA technique that alleviates BMLs of KOA.

C1 Shanghai Univ Tradit Chinese Med, Shenzhen Hosp, Geriatr Dept, Shenzhen, Peoples R China; Shanghai Univ Tradit Chinese Med, Grad Sch, Shanghai, Peoples R China; Shanghai Univ Tradit Chinese Med, Guanghua Hosp, Joint Surg Dept, Shanghai, Peoples R China

DI 10.3389/fneur.2022.1026441

ID 79

ER

FN Clarivate Analytics Web of science

PT J

AU Yin, S.

AU - Zhang, Z. H.

AU - Chang, Y. N.

AU - Huang, J.

AU - Wu, M. L.

AU - Li, Q.

AU - Qiu, J. Q.

AU - Feng, X. D.

AU - Wu, N.

PY 2022

TI Effect of Acupuncture on the Cognitive Control Network of Patients with Knee Osteoarthritis : Study Protocol for a Randomized Controlled Trial

PG 1443-1455

DA 2022/1/1

SO JOURNAL OF PAIN RESEARCH

JO JOURNAL OF PAIN RESEARCH

VL 15

SN 1178-7090

Z9 Times Cited in Web of Science Core Collection: 1 Total Times Cited: 1 Cited Reference Count: 55 ER -

M3 10.2147/JPR.S356044

N1 YinZhang-88

N1 2025/2/13 1:16:00

DE knee osteoarthritis; acupuncture; cognitive control network; randomized controlled trial; CHRONIC PAIN; ARTHRITIS; THERAPY; CONNECTIVITY; METAANALYSIS; INVENTORY; ANXIETY; SCALES; IMPACT

AB Purpose: Abnormal central nervous system function is the key central pathological factor leading to chronic pain in patients with knee osteoarthritis (KOA). Acupuncture can effectively relieve the pain of KOA patients. However, the central nervous mechanism of acupuncture treating KOA is not fully understood. This trial will use functional magnetic resonance imaging (fMRI) analysis techniques to investigate the potential central nervous mechanism of acupuncture treatment of KOA. Materials and Methods: A total of 108 patients will be randomized (in a 1:1:1 ratio) into three groups, this trial will include 4-week treatment, patients in groups A and B will receive 20 acupuncture and sham acupuncture sessions, respectively, patients in group C will not receive any intervention, and all patients will receive fMRI scans before and after the intervention. The Western Ontario and McMaster Universities Osteoarthritis Index score (WOMAC) will be the primary clinical outcome. Then, we will explore the functional changes of the cognitive control network (CCN) in the brains of KOA patients through whole brain functional connectivity (FC) analysis and seed-based functional connectivity (sFC) analysis. Pearson correlation coefficient will be used to analyze the relationship between the improved value of the clinical correlation scale and the change of fMRI data. Discussion: This trial will analyze the efficacy of verum acupuncture, sham acupuncture and the waiting-list for KOA and explore the activity of the CCN in three groups of patients by fMRI, so as to reveal the central nervous mechanisms of acupuncture in the treatment of KOA. Study Registration: This study is approved by the Ethics Committee of the First Affiliated Hospital of Henan University of Traditional Chinese Medicine (No: 2019HL-133-01) and registered in the Chinese Clinical Trial Registry, ChiCTR2000038554.

C1 Henan Univ Chinese Med, Rehabil Ctr, Affiliated Hosp 1, 19 Renmin Rd, Zhengzhou 450000, Henan, Peoples R China; Henan Univ Chinese Med, Sch Rehabil Med, 156 Jinshui East Rd, Zhengzhou 450046, Henan, Peoples R China

DI 10.2147/JPR.S356044

ID 88

ER

FN Clarivate Analytics Web of science

PT J

AU Jiang, G. Y.

AU - Ding, J. G.

AU - Ge, C. L.

PY 2022

TI Deep Learning-Based CT Imaging to Evaluate the Therapeutic Effects of Acupuncture and Moxibustion Therapy on Knee Osteoarthritis

DA 2022/1/1

SO COMPUTATIONAL AND MATHEMATICAL METHODS IN MEDICINE

JO COMPUTATIONAL AND MATHEMATICAL METHODS IN MEDICINE

VL 2022

SN 1748-670X

Z9 Times Cited in Web of Science Core Collection: 4 Total Times Cited: 4 Cited Reference Count: 35 ER -

M3 10.1155/2022/1135196

N1 JiangDing-90

N1 2025/2/13 1:16:00

DE SAFETY

AB The study was aimed at analyzing the application value of deep learning-based computed tomography (CT) in evaluating the effect of acupuncture for knee osteoarthritis (KOA). Specifically, 124 patients with KOA were selected in the test group (warm acupuncture and moxibustion) and the control group (simple acupuncture), with 62 cases in each group. Deep learning-based CT scanning was performed before and after treatment to compare the Lequesne-Mery, Visual Analog Scale (VAS), Western Ontario and McMaster Universities (WOMAC), Hospital Special Surgery (HSS), and Knee Society Score (KSS) scores as well as the overall effective rate. The results showed that the trabecular thickness, quantity, bone mineral density (BMD), connection density, structural model index, and articular cartilage thickness were different significantly between the two groups (P < 0.05). After treatment, the Lequesne-Mery was 4.78, the VAS was 0.87, and the WOMAC score was 14.89 of the test group, which were reduced (P < 0.05). The KSS and HSS scores of the test group were improved significantly after treatment (P < 0.05). The total effective rate of the test group was 85.48%, and that of the control group was 51.61%; the former was significantly higher than the latter (P < 0.05). In conclusion, acupuncture could improve the clinical effect on KOA patients, and CT scanning under deep learning algorithm could evaluate the clinical effect of acupuncture for KOA.

C1 Changyi Peoples Hosp, Dept Chinese Med, Changyi 261300, Shandong, Peoples R China; Changyi Peoples Hosp, Dept Crit Med, Changyi 261300, Shandong, Peoples R China; Changyi Peoples Hosp, Dept CT, Changyi 261300, Shandong, Peoples R China

DI 10.1155/2022/1135196

ID 90

ER

FN Clarivate Analytics Web of science

PT J

AU Cai, F. H.

AU - Li, F. L.

AU - Zhang, Y. C.

AU - Li, P. Q.

AU - Xiao, B.

PY 2022

TI Research on electroacupuncture parameters for knee osteoarthritis based on data mining

DA 2022/1/1

SO EUROPEAN JOURNAL OF MEDICAL RESEARCH

JO EUROPEAN JOURNAL OF MEDICAL RESEARCH

VL 27

IS 1

SN 0949-2321

Z9 Times Cited in Web of Science Core Collection: 11 Total Times Cited: 12 Cited Reference Count: 68 ER -

M3 10.1186/s40001-022-00795-9

N1 CaiLi-92

N1 2025/2/13 1:16:00

DE Electroacupuncture; Knee osteoarthritis; Parameter; Data mining; ACUPUNCTURE; PAIN

AB Background Knee osteoarthritis, a common degenerative joint disease, has been widely treated by electroacupuncture in recent years. However, there are too many parameters of the treatment currently, resulting in various applications in clinical practice. This study aims to summarize the optimal stimulation parameters of electroacupuncture for knee osteoarthritis in clinical studies by applying data mining techniques. Methods Four databases including Pubmed, Cochrane Library, Embase, and Web of Science were searched for clinical studies on electroacupuncture treating knee osteoarthritis from 2012 to 2021. A database was established by Microsoft Excel 2020 and analyzed by R Version 4.1.1. Results Forty-six articles were included according to the established criteria. The most used electroacupuncture stimulation parameters were 0.30 mm x 40 mm needle, continuous wave, low frequency of current (mainly 2 Hz), stimulation duration for 30 min per treatment, and frequency of treatment for once a day. Eighteen acupoints were mentioned and the most used ones include Dubi (ST35), Liangqiu (ST34), Neixiyan (EX-LE4), Xuehai (SP10), Yanglingquan (GB34), and Yinlingquan (SP9), and those most generally used acupoints are closely arranged on the Stomach Channel of Foot Yangming. Cluster analysis showed two groups, one for obligatory acupoints and one for adjunctive ones. The association analysis showed the most supported acupoint pair was Liangqiu (ST34) and Xuehai (SP10). Conclusions Continuous wave, low frequency of current (2 Hz), 30-min stimulation, and local acupoint selection are frequently used for electroacupuncture treating knee osteoarthritis. Due to the limitations of this study, further research and more standardized, multi-centered, and large-sample clinical trials should be conducted to provide more convincing evidence.

C1 Shanghai Univ TCM, Coll Acupuncture & Tuina, Shanghai 201203, Peoples R China

DI 10.1186/s40001-022-00795-9

ID 92

ER

FN Clarivate Analytics Web of science

PT J

AU Jin, S. A.

AU - Guan, X. F.

PY 2022

TI A systematic review and meta-analysis of the comparative curative effects of warm acupuncture and other traditional Chinese medicines in the treatment of knee osteoarthritis

PG 708-716

DA 2022/1/1

SO ANNALS OF PALLIATIVE MEDICINE

JO ANNALS OF PALLIATIVE MEDICINE

VL 11

IS 2

SN 2224-5820

Z9 Times Cited in Web of Science Core Collection: 3 Total Times Cited: 3 Cited Reference Count: 32 ER -

M3 10.21037/apm-21-3972

N1 JinGuan-111

N1 2025/2/13 1:16:00

DE Warm needling acupuncture (WNA); traditional Chinese medicine (TCM); knee osteoarthritis (KOA); meta-analysis; PAIN; MANAGEMENT; EFFICACY; THERAPY

AB Backgrounds: Knee osteoarthritis (KOA) is more common in middle-aged and elderly people, and seriously affects the quality of life of those affected. Traditional Chinese medicine (TCM) treatment of KOA has been widely recognized. In recent years, warm needling acupuncture (WNA) has been used to treat KOA and has achieved good results. However, there is a lack of comparison of the efficacy of WNA and other TCM treatments for KOA. Methods: We conducted a search for reports of WNA and/or TCM treatment of KOA in English- and Chinese-language databases. The data was retrieved from inception of the database until October 2021. The Cochrane risk of bias tool was used to evaluate the quality of the included studies, and the network meta-analysis was performed using the software RevMan 5.20. Results: A total of 8 articles met the inclusion criteria, including 399 patients treated with WNA (WNA group), and 396 patients treated with other TCM (TC.114 group). The results of meta-analysis showed that compared with patients in the TCM group, the effective rate [relative risk (RR)] was 1.18, 95% confidence interval (CI): 1.06 to 1.33, the last follow-up osteoarthritis index [mean difference (MD)] was -6.93, 95% CI: -12.14 to -1.72, and the last follow-up knee pain visual analogue scale (VAS) MD was -1.06, 95% CI: -1.61 to -0.51, which were all statistically significant. However, the difference in daily activities (MD: -4.31, 95% CI: -10.90 to 2.28) was not statistically significant. Discussion Compared with other TCM treatments for KOA, WNA has better overall patient efficacy. However, further randomized controlled studies are needed to compare WNA and other TCM treatments individually to confirm the efficacy of WNA.

C1 Liaoning Univ Tradit Chinese Med, 79 Chongshan East Rd, Shenyang 110000, Peoples R China

DI 10.21037/apm-21-3972

ID 111

ER

FN Clarivate Analytics Web of science

PT J

AU Gao, N.

AU - Shi, H. P.

AU - Hu, S.

AU - Zha, B. X.

AU - Yuan, A. H.

AU - Shu, J. H.

AU - Fan, Y. Q.

AU - Bai, J.

AU - Xie, H. Y.

AU - Cui, J. C.

AU - Wang, X. X.

AU - Li, C. F.

AU - Qiu, B. S.

AU - Yang, J.

PY 2022

TI Acupuncture Enhances Dorsal Raphe Functional Connectivity in Knee Osteoarthritis With Chronic Pain

DA 2022/1/1

SO FRONTIERS IN NEUROLOGY

JO FRONTIERS IN NEUROLOGY

VL 12

SN 1664-2295

Z9 Times Cited in Web of Science Core Collection: 9 Total Times Cited: 10 Cited Reference Count: 32 ER -

M3 10.3389/fneur.2021.813723

N1 GaoShi-113

N1 2025/2/13 1:16:00

DE functional magnetic resonance imaging; chronic pain; acupuncture; functional connectivity; knee osteoarthritis; MODULATION; NUCLEUS

AB IntroductionKnee osteoarthritis is a common disease in the elderly. Patients suffer from long-term chronic pain and reduced life quality. Acupuncture has been proven to be an effective treatment for KOA. However, the neural mechanism of acupuncture is unclear, so far. Periaqueductal gray (PAG) and raphe nuclei (RPN) are essential structures associated with chronic pain in human brains. This study aims to investigate functional connectivity (FC) changes of PAG and RPN in KOA to interpret the neural mechanism of acupuncture. MethodsIn 15 patients with KOA and 15 healthy controls (HC), we acquired Visual Analog Scale (VAS) scores and resting-state fMRI images of each participant before and after acupuncture stimulation on EX-LE5 acupoint. Then, PAG and RPN were selected as seeds to perform FC analysis based on resting-state fMRI images. Finally, we compared FC patterns of PAG and RPN between patients with KOA and HC, then between pre-acupuncture and post-acupuncture. Correlations between FC values and VAS scores were calculated as well. ResultsFor PAG, FC of patients with KOA was lower in the right lingual gyrus at post-acupuncture compared with HC (p <0.001, uncorrected). For dorsal RPN, FC of patients with KOA was significantly higher in right putamen at post-acupuncture compared with HC (p <0.001, corrected with FDR), and FC changes were significant between pre-acupuncture and post-acupuncture in patients with KOA. Post-acupuncture FC values between dorsal RPN and right putamen were correlated with VAS scores. For medial RPN, FC of patients with KOA was lower in the right cerebellum at post-acupuncture compared with HC (p <0.001, uncorrected), but no significant FC changes were found between pre-acupuncture and post-acupuncture in patients with KOA. FC values between medial RPN and right cerebellum were not correlated with VAS scores at pre-acupuncture and post-acupuncture. DiscussionOur study demonstrated that acupuncture enhanced FC between dorsal RPN and the right putamen in patients with KOA, which was associated with chronic pain intensity. This result suggests that acupuncture stimulation can enhance FC between dorsal raphe and striatum, illustrating a neural mechanism that acupuncture can drive the patients' brain, with KOA, to perceive pain.

C1 Univ Sci & Technol China, Ctr Biomed Imaging, Hefei, Peoples R China; Anhui Univ Tradit Chinese Med, Affiliated Hosp 1, Hefei, Peoples R China; Anhui Univ Chinese Med, Sch Med Informat Engn, Hefei, Peoples R China; Third Peoples Hosp Hefei, Hefei, Peoples R China

DI 10.3389/fneur.2021.813723

ID 113

ER

FN Clarivate Analytics Web of science

PT J

AU Yin, X. L.

AU - Liu, Y.

AU - Liu, W.

AU - Liang, W.

AU - Liang, Q. Y.

PY 2022

TI Blade needle therapy versus conventional acupuncture for knee osteoarthritis: A meta-analysis

DA 2022/1/1

SO MEDICINE

JO MEDICINE

VL 101

IS 30

SN 0025-7974

Z9 Times Cited in Web of Science Core Collection: 0 Total Times Cited: 0 Cited Reference Count: 45 ER -

M3 10.1097/MD.0000000000029647

N1 YinLiu-132

Y2 2025/1/17 15:55:00

N1 2025/2/13 1:16:00

DE blade needle; conventional acupuncture; knee osteoarthritis; meta-analysis; HEALTH; PREVALENCE

AB Background: This study investigated the hypothesis that the efficacy of blade needle therapy for the treatment of knee osteoarthritis (KOA) is superior to that of conventional acupuncture. In addition, the efficacy of blade needle therapy versus conventional acupuncture for the treatment of KOA was analyzed in a meta-analysis. Methods: Randomized controlled trials (RCTs) of blade needle therapy and conventional acupuncture for treating KOA were retrieved from the electronic databases CNKL, Wanfang, VIP, PubMed, EMBASE and the Cochrane Library from the commencement of each database to July of 2021. Data were extracted and evaluated by 2 reviewers independently. RevMan 5.3 software was used to conduct the meta-analysis after the studies were evaluated. Results: A total of 11 RCTs were included, all from China, involving 1142 patients. The meta-analysis results showed that the effective rate of the blade needle group was better than that of the conventional acupuncture group (OR = 3.61, 95% CI [2.56-5.10], P < .00001). Conclusion: The efficacy of blade needle treatment for KOA is superior to that of conventional acupuncture, but more high-quality studies are needed for future validation due to the low proportion of high-quality studies included and the possible bias factor.

C1 Guangxi Univ Chinese Med, Nanning, Peoples R China

DI 10.1097/MD.0000000000029647

ID 132

ER

FN Clarivate Analytics Web of science

PT J

AU Wang, Z.

AU - Wang, Y. Q.

AU - Wang, C. A.

AU - Li, X. J.

AU - Zhou, Z. Y.

AU - Zhang, L. J.

AU - Li, M. X.

AU - Pan, Y. K.

AU - Jiao, T. Y.

AU - Shi, X. Y.

AU - Liu, Q.

PY 2022

TI Systematic Review and Network Meta-analysis of Acupuncture Combined with Massage in Treating Knee Osteoarthritis

DA 2022/1/1

SO BIOMED RESEARCH INTERNATIONAL

JO BIOMED RESEARCH INTERNATIONAL

VL 2022

SN 2314-6133

Z9 Times Cited in Web of Science Core Collection: 7 Total Times Cited: 7 Cited Reference Count: 90 ER -

M3 10.1155/2022/4048550

N1 WangWang-133

N1 2025/2/13 1:16:00

DE STRATEGIES

AB Background. Knee osteoarthritis is a common clinical disease with frequent occurrence. More and more studies have shown that external therapies such as acupuncture and massage are beneficial to the treatment of knee osteoarthritis. Objective. The purpose of this systematic review and meta-analysis of randomized controlled trials (RCTS) was to evaluate the efficacy and safety of acupuncture and massage combined with treatment of KOA and to provide some reference for clinical treatment of KOA. Methods. Network meta-analysis was used to evaluate the efficacy of acupuncture combined with massage in the treatment of knee osteoarthritis. PubMed, Cochrane Library, Web of Science, Embase, Chinese Knowledge Infrastructure (CNKI), Chinese Biomedical Literature Database (CBM), VIP, and Wanfang were searched by computer for randomized controlled trials on acupuncture combined with massage in the treatment of knee osteoarthritis. All researchers independently screened the literature, extracted data, and evaluated quality, and studies that met the quality criteria were analyzed using Stata16.0 software. Results. A total of 3076 articles were retrieved, and finally, 49 studies involving 10 acupuncture combined with massage methods were included. The total sample size was 4458, including 2182 in the experimental group and 2276 in the control group. The results of network meta-analysis showed the following: in terms of effective rate, the optimal first three interventions were floating needle+massage, needle knife+massage, and silver needle+massage; in terms of reducing VAS score, the optimal first three interventions were common acupuncture+massage, needle knife+massage, and warm needle+massage; in terms of improving total Lysholm index score, the optimal first three interventions were silver needle+massage, electroacupuncture+massage, and needle knife+massage; in terms of reducing total WOMAC score, the optimal first three interventions were silver needle+massage, electrothermal needle+massage, and common acupuncture+massage; in terms of reducing WOMAC stiffness score, the optimal first three interventions were warm needle+massage, silver needle+massage, and common acupuncture+massage; and in terms of reducing WOMAC joint function score, the optimal first three interventions were silver needle+massage, warm needle+massage, and common acupuncture+massage. Conclusion. The results showed that acupuncture combined with massage could improve the clinical therapeutic effect of patients with knee osteoarthritis. Limited by the quality of the included studies, the conclusions obtained still need to be further validated.

C1 Shandong Univ Tradit Chinese Med, Jinan, Peoples R China; Shandong Univ Tradit Chinese Med, Affiliated Hosp, Jinan, Peoples R China; Shandong First Med Univ, Neck Shoulder Waist & Leg Pain Hosp, Jinan, Peoples R China

DI 10.1155/2022/4048550

ID 133

ER

FN Clarivate Analytics Web of science

PT J

AU Liu, C. Y.

AU - Tu, J. F.

AU - Lee, M. S.

AU - Qi, L. Y.

AU - Yu, F. T.

AU - Yan, S. Y.

AU - Li, J. L.

AU - Lin, L. L.

AU - Hao, X. W.

AU - Su, X. T.

AU - Yang, J. W.

AU - Wang, L. Q.

PY 2022

TI Is acupuncture effective for knee osteoarthritis? A protocol for a systematic review and meta-analysis

DA 2022/1/1

SO BMJ OPEN

JO BMJ OPEN

VL 12

IS 1

SN 2044-6055

Z9 Times Cited in Web of Science Core Collection: 7 Total Times Cited: 7 Cited Reference Count: 53 ER -

M3 10.1136/bmjopen-2021-052270

N1 LiuTu-146

Y2 2025/1/17 16:05:00

N1 2025/2/13 1:16:00

DE knee; protocols & guidelines; complementary medicine; SHAM ACUPUNCTURE; ADVERSE EVENTS; RAT MODEL; PAIN; ELECTROACUPUNCTURE; MANAGEMENT; RECEPTOR; RISK; HAND; HIP

AB Introduction Knee osteoarthritis (KOA) is one of the leading causes of disability. The effectiveness of acupuncture for treating KOA remains controversial. This protocol describes the method of a systematic review and meta-analysis evaluating the effectiveness and safety of acupuncture for treating KOA. Methods and analysis Four English databases (PubMed, Embase, Cochrane Library databases and Web of Science) and four Chinese databases (China National Knowledge Infrastructure, Chinese Biomedical Literature Database, VIP Database for Chinese Technical Periodicals, and Wanfang) will be searched from the database inception to 1 September 2021. All randomised controlled trials related to acupuncture for KOA will be included. Extracted data will include publication details, basic information, demographic data, intervention details and patient outcomes. The primary outcome will be pain intensity. Risk of bias will be assessed using the Cochrane Collaboration's tool for assessing risk of bias. Article selection, data extraction and risk of bias assessment will be performed in duplicate by two independent reviewers. If the meta-analysis is precluded, we will conduct a descriptive synthesis using a best-evidence synthesis approach. The strength of recommendations and quality of evidence will be assessed using the Grading of Recommendations Assessment Development and Evaluation working group methodology. Ethics and dissemination Ethics approval is not required because individual patient data are not included. This protocol was registered in the international Prospective Register of Systematic Reviews on 25 February 2021. The systematic review and meta-analysis will be submitted for publication in a peer-reviewed journal. The findings will also be disseminated through conference presentations.

C1 Beijing Univ Chinese Med, Sch Tradit Chinese Med, Beijing, Peoples R China; Capital Med Univ, Sch Tradit Chinese Med, Beijing, Peoples R China; Beijing Univ Chinese Med, Sch Acupuncture Moxibust & Tuina, Beijing, Peoples R China; Korea Inst Oriental Med, KM Sci Res Div, Daejon, South Korea

DI 10.1136/bmjopen-2021-052270

ID 146

ER

FN Clarivate Analytics Web of science

PT J

AU Liu, J. L.

AU - Li, Y.

AU - Li, L.

AU - Luo, X. C.

AU - Li, N.

AU - Yang, X. G.

AU - Zhang, H. X.

AU - Liu, Z. B.

AU - Kang, D. Y.

AU - Luo, Y. A.

AU - Liu, Y. M.

AU - Jia, Y. L.

AU - Ren, Y.

AU - Yao, M. H.

AU - Wang, Y. N.

AU - Chen, J.

AU - Maiji, M.

AU - Zou, K.

AU - Zhao, L.

AU - Liang, F. R.

AU - Sun, X.

PY 2022

TI Effects of acupuncture at acupoints with lower versus higher pain threshold for knee osteoarthritis: a multicenter randomized controlled trial

DA 2022/1/1

SO CHINESE MEDICINE

JO CHINESE MEDICINE

VL 17

IS 1

SN 1749-8546

Z9 Times Cited in Web of Science Core Collection: 7 Total Times Cited: 7 Cited Reference Count: 48 ER -

M3 10.1186/s13020-022-00626-3

N1 LiuLi-161

Y2 2025/1/17 15:27:00

N1 2025/2/13 1:16:00

DE LOW-BACK-PAIN; EXPERT CONSENSUS; PRESSURE PAIN; DIAGNOSIS; MANAGEMENT; MEDICINE; SENSITIVITY; GUIDELINE; RELIEF; WOMAC

AB Background: The acupoint selections impact the effects of acupuncture, and preliminary evidence showed potential connection between pain threshold (PT) and acupuncture response. This study examined whether acupuncture at acupoints with lower PT versus higher PT would yield different effects in patients with knee osteoarthritis (KOA). Methods: In this multicenter randomized clinical trial, patients were randomly assigned (1:1:1) to receive acupuncture at acupoints with lower PT (LPT group), acupuncture at acupoints with higher PT (HPT group), and no acupuncture (waiting-list group). PT was measured with electronic von Frey detector. The primary outcome was the change in WOMAC total score from baseline to 16 weeks, and the secondary outcomes were SF-12 score, and active knee range of motion (ROM). Intention-to-treat analysis was conducted with linear mixed-effect model. Results: Among 666 randomized patients, 625 (93.84%) completed the study. From baseline to 16 weeks, patients in the LPT group versus HPT group had similar effects in reducing WOMAC total score (adjusted mean difference (MD) 2.21, 95% confidence interval (CI) -2.51 to 6.92, P =0.36), while a greater reduction in WOMAC total score was observed in LPT group (-9.77, 95% CI -14.47 to -5.07, P < 0.001) and HPT group (-11.97, 95% CI -16.71 to -7.24, P < 0.001) compared with waiting-list group. There were no differences in SF-12 score and knee ROM between LPT versus HPT groups. Conclusion: Our findings found that the effects of acupuncture at acupoints with lower versus higher PT were similar, both were effective for patients with KOA.

C1 Sichuan Univ, West China Hosp, Chinese Evidence Based Med Ctr, 37 Guo Xue Xiang, Chengdu 610041, Sichuan, Peoples R China; Sichuan Univ, West China Hosp, Cochrane China Ctr, 37 Guo Xue Xiang, Chengdu 610041, Sichuan, Peoples R China; NMPA Key Lab Real World Data Res & Evaluat Hainan, Chengdu 610041, Sichuan, Peoples R China; Sichuan Ctr Technol Innovat Real World Data, Chengdu 610041, Sichuan, Peoples R China; Chengdu Univ Tradit Chinese Med, Grad Sch, Chengdu 610075, Sichuan, Peoples R China; Sichuan Univ, West China Hosp, Dept Integrated Tradit Chinese & Western Med, Chengdu 610041, Peoples R China; Henan Univ Tradit Chinese Med, Coll Acupuncture & Massage, Zhengzhou 450008, Peoples R China; Wuhan Hosp Tradit Chinese & Western Med, Dept Acupuncture, Wuhan 430022, Peoples R China; Chengdu Univ Tradit Chinese Med, Acupuncture & Tuina Sch, Chengdu 610075, Sichuan, Peoples R China; Jiangxi Univ Tradit Chinese Med, Evidence Based Med Res Ctr, Sch Basic Sci, Nanchang 330004, Jiangxi, Peoples R China

DI 10.1186/s13020-022-00626-3

ID 161

ER

FN Clarivate Analytics Web of science

PT J

AU Wang, X. W.

AU - Sun, Q.

AU - Wang, M.

AU - Chen, Y. R.

AU - Wang, Q. G.

AU - Liu, L. X.

AU - Yuan, Y.

PY 2022

TI Electrical Dry Needling Plus Corticosteroid Injection for Osteoarthritis of the Knee: A Randomized Controlled Trial

PG 858-866

DA 2022/1/1

SO ARCHIVES OF PHYSICAL MEDICINE AND REHABILITATION

JO ARCHIVES OF PHYSICAL MEDICINE AND REHABILITATION

VL 103

IS 5

SN 0003-9993

Z9 Times Cited in Web of Science Core Collection: 1 Total Times Cited: 2 Cited Reference Count: 56 ER -

M3 10.1016/j.apmr.2021.12.026

N1 WangSun-171

Y2 2025/1/18 22:03:00

N1 2025/2/13 1:16:00

DE Corticosteroid injection; Dry needling; Osteoarthritis of the knee; Rehabilitation; Trigger point; MYOFASCIAL TRIGGER POINTS; MUSCLE STRENGTH; PHARMACOLOGICAL-TREATMENT; MANUAL ACUPUNCTURE; PHYSICAL FUNCTION; CHRONIC PAIN; HIP; EXERCISE; THERAPY; QUALITY

AB Objective: To investigate the effects of electrical dry needling (DN) plus corticosteroid injection (CSI) on pain, physical function, and global change in patients with osteoarthritis of the knee (KOA). Design: A prospective, single-blinded, randomized controlled trial. Setting: Pain treatment clinic. Participants: Sixty patients with KOA were randomly assigned to the electrical dry needling plus corticosteroid injection (electrical-DN+CSI) group or CSI group. Interventions: The CSI group received glucocorticoid injection only once during the trial, and the electrical-DN+CSI group received glucocorticoid injection combined with 4 sessions of electrical-DN. Main Outcomes Measures: The primary outcome was the numerical rating scale at 3 months. The secondary outcomes were the Western Ontario and McMaster Universities Osteoarthritis Index, the time to complete the Timed Up and Go test, and the score of the global rating of change scale at 3 months. A generalized linear mixed-effects model was used to analyze the repeated measurement data. Results: Baseline characteristics and measurements were similar in the 2 groups. The group by time interaction effect was significant for all variables (P<.05). The electrical-DN+CSI group obtained a more significant reduction in pain intensity and more significant improvement in dysfunction than the CSI group at 3 months (P<.05). The median global rating of change score for the CSI group was +3 (somewhat better), and that for the electrical-DN+CSI group was +4 (moderately better). Conclusion: Electrical-DN therapy at myofascial trigger points combined with CSI is more effective at alleviating pain, improving dysfunction, and creating global change than CSI alone for patients with KOA. Electrical-DN may be an essential part of treatment for KOA rehabilitation. (C) 2022 The American Congress of Rehabilitation Medicine. Published by Elsevier Inc. All rights reserved.

C1 Xuzhou Med Univ, Coll Anesthesiol, Xuzhou, Jiangsu, Peoples R China; Xuzhou Med Univ, Dept Pain Treatment, Affiliated Hosp, West Huaihai Rd, Xuzhou 221006, Jiangsu, Peoples R China

DI 10.1016/j.apmr.2021.12.026

ID 171

ER

FN Clarivate Analytics Web of science

PT J

AU Wang, L.

AU - Wei, J. T.

AU - Qian, Z.

AU - Qian, J.

PY 2022

TI Acupotomy by ultrasound-guided versus anatomical guidance in knee osteoarthritis: A protocol for systematic review and meta-analysis

DA 2022/1/1

SO MEDICINE

JO MEDICINE

VL 101

IS 47

SN 0025-7974

Z9 Times Cited in Web of Science Core Collection: 0 Total Times Cited: 0 Cited Reference Count: 20 ER -

M3 10.1097/MD.0000000000031693

N1 WangWei-179

Y2 2025/1/17 15:28:00

N1 2025/2/13 1:16:00

DE knee osteoarthritis; pain; range of motion; ultrasonic-guided acupotomy; SAFETY

AB Background:At present, there is no systematic evaluation on whether ultrasonic-guided acupotomy is more effective compared with anatomical guidance in knee osteoarthritis. We conducted a protocol for systematic review and meta-analysis to provide a method for evaluating the effectiveness and safety of acupotomy by ultrasound-guided technique. Methods:An all-round retrieval will be performed in the following electronic journal databases from their inception to October 2022, which comprise PubMed, MEDLINE, EMBASE, Cochrane Library, China National Knowledge Infrastructure, Wanfang data, Chinese Scientific Journals Database, and China biomedical literature database. The following key words were used on combination with Boolean operators AND or OR: "acupotomy," "ultrasound," "knee osteoarthritis." Two authors completed the quality assessment using the Cochrane Collaborations risk of bias tool. The meta-analysis was conducted using Review Manager 5.3 software from the Cochrane Collaboration (London, UK). Results:The findings of this study will be submitted to peer-reviewed journals for publication. Conclusion:This systematic review will provide evidence to judge whether acupotomy by ultrasound-guided technique is effective and safe for knee osteoarthritis.

C1 Hexi Univ, Dept Ultrasound, Zhangye Peoples Hosp, Zhangye, Gansu, Peoples R China; Hexi Univ, Dept Orthoped, Zhangye Peoples Hosp, Zhangye, Gansu, Peoples R China; Hexi Univ, Dept Orthoped, Zhangye Peoples Hosp, Zhangye 734000, Gansu, Peoples R China

DI 10.1097/MD.0000000000031693

ID 179

ER

FN Clarivate Analytics Web of science

PT J

AU Lin, X. Z.

AU - Li, F.

AU - Lu, H. L.

AU - Zhu, M. H.

AU - Peng, T. Z.

PY 2022

TI Acupuncturing of myofascial pain trigger points for the treatment of knee osteoarthritis A systematic review and meta-analysis

DA 2022/1/1

SO MEDICINE

JO MEDICINE

VL 101

IS 8

SN 0025-7974

Z9 Times Cited in Web of Science Core Collection: 2 Total Times Cited: 2 Cited Reference Count: 21 ER -

M3 10.1097/MD.0000000000028838

N1 LinLi-182

N1 2025/2/13 1:16:00

DE acupuncture; knee osteoarthritis; meta-analysis; myofascial pain trigger points; systematic evaluation

AB Background: Osteoarthritis of the knee is one of the major disorders leading to social dysfunction, economic loss and social development. This study was conducted to systematically evaluate the efficacy and safety effectiveness of acupuncture inactivation of myofascial pain trigger points in the treatment of osteoarthritis of the knee. Methods: Randomized Controlled Trials (RCTs) on the treatment of knee pain were searched by computer from PubMed, The Cochrane Library, China journal full-text database (CNKI), Chinese biomedical literature database (CBM), China academic journal database (Wanfang Data) databases from the date of creation to December 2021, and the data were analyzed by Reman 5.3 software for data analysis. Results: A total of 724 patients from 9 RCTs were finally included, and the results of meta-analysis showed that the acupuncture myofascial pain trigger point group was better than the control group in terms of total effective rate, cure rate, VAS score, Lysholm score, and WOMAC score. Conclusion: The efficacy and safety of acupuncturing myofascial pain trigger points in the treatment of knee osteoarthritis is positive, but due to the limited number of literature included in this study and the low quality of the included literature, there is still a need for high-quality and large sample size RCTs for the analysis of this treatment option.

C1 Nanchang Hongdu Hosp Tradit Chinese Med, Nanchang 330000, Jiangxi, Peoples R China

DI 10.1097/MD.0000000000028838

ID 182

ER

FN Clarivate Analytics Web of science

PT J

AU Woo, S. H.

AU - Lee, H. J.

AU - Park, Y. K.

AU - Han, J.

AU - Kim, J. S.

AU - Lee, J. H.

AU - Park, C. A.

AU - Choi, S. H.

AU - Lee, W. D.

AU - Yang, C. S.

AU - Kim, M. J.

AU - Han, C. H.

PY 2022

TI Efficacy and safety of thread embedding acupuncture for knee osteoarthritis: A randomized controlled pilot trial

DA 2022/1/1

SO MEDICINE

JO MEDICINE

VL 101

IS 31

SN 0025-7974

Z9 Times Cited in Web of Science Core Collection: 4 Total Times Cited: 4 Cited Reference Count: 19 ER -

M3 10.1097/MD.0000000000029306

N1 WooLee-195

Y2 2025/1/17 15:59:00

N1 2025/2/13 1:16:00

DE acupuncture; knee osteoarthritis; thread-embedding acupuncture; PAIN; INTERVENTIONS; RELIABILITY; THERAPY

AB Background: Thread embedding acupuncture (TEA) is a widely used clinical procedure for the treatment of musculoskeletal pain. However, few clinical studies have been conducted on the efficacy and safety of TEA for knee osteoarthritis (KOA), and data from randomized controlled trials are lacking. This randomized controlled pilot study aimed to assess the feasibility of conducting large-scale studies on the efficacy and safety of TEA for KOA. Methods: Forty participants were included in the study and randomly divided into 2 groups (TEA and acupuncture) of 20 each. The intervention period was 6 weeks. The experimental group received TEA once a week (total of 6 sessions) on 14 defined knee areas, and the control group received acupuncture twice a week (total of 12 sessions) on 9 defined acupuncture points. The primary outcome measure was the visual analogue scale score, and the secondary outcome measures were the short-form McGill pain questionnaire, and Western Ontario and McMaster Universities Osteoarthritis Index scores. Participants were assessed prior to the intervention (baseline) and at 3, 6, and 10 weeks (4 weeks after the end of intervention). The adverse effects of TEA and acupuncture were documented. Hematological examination and biochemical tests were performed at the screening and at 6 weeks. Results: Of the 40 participants, 37 completed the study and 3 participants dropped out. Both the TEA and acupuncture groups showed a significant improvement in the visual analogue scale, short-form McGill Pain Questionnaire, and Western Ontario and McMaster Universities Osteoarthritis Index scores in a time-dependent manner. However, there was no significant interaction between group and time. No serious adverse events were reported in the groups, and no clinically significant changes were observed in the hematological and biochemical parameters. Conclusion: This pilot study suggests that TEA is a safe and effective procedure for relieving pain in patients with KOA. The results of this study provide basic data and indicate the feasibility of large-scale clinical studies to evaluate the efficacy and safety of TEA for KOA.

C1 Daegu Haany Univ, Coll Korean Med, Dept Acupuncture & Moxibust, Daegu, South Korea; Daegu Haany Univ, Coll Korean Med, Dept Diagnost, Daegu, South Korea; Daegu Haany Univ, Coll Korean Med, Dept Anat & Histol, Daegu, South Korea; Daegu Haany Univ, Coll Herbal Bioind, Premajor Cosmet & Pharmaceut, Daegu, South Korea; Korea Inst Oriental Med, KM Sci Res Div, Daejeon 34054, South Korea; Korea Inst Oriental Med, R&D Strategy Div, Daejeon, South Korea; Univ Sci & Technol UST, Campus Korea Inst Oriental Med, Korean Convergence Med, Daejeon, South Korea

DI 10.1097/MD.0000000000029306

ID 195

ER

FN Clarivate Analytics Web of science

PT J

AU Yuan, X. C.

AU - Wang, Y. Y.

AU - Tian, L. X.

AU - Yan, X. J.

AU - Guo, Y. X.

AU - Zhao, Y. L.

AU - Baba, S. S.

AU - Jia, H.

AU - Wang, H. S.

AU - Li, M.

AU - Huo, F. Q.

PY 2022

TI Spinal 5-HT<sub>2A</sub> receptor is involved in electroacupuncture inhibition of chronic pain

DA 2022/1/1

SO MOLECULAR PAIN

JO MOLECULAR PAIN

VL 18

SN 1744-8069

Z9 Times Cited in Web of Science Core Collection: 8 Total Times Cited: 10 Cited Reference Count: 31 ER -

M3 10.1177/17448069221087583

N1 YuanWang-224

Y2 2025/1/17 14:32:00

N1 2025/2/13 1:16:00

DE knee osteoarthritis (KOA); electroacupuncture analgesia; 5-HT2A receptor; GABA(A) receptor; KCC2; chronic pain; GABAERGIC INHIBITION; DOWN-REGULATION; DORSAL-HORN; MOUSE MODEL; OSTEOARTHRITIS; ACTIVATION; ALLODYNIA; AGONISTS; KNEE; RATS

AB Knee osteoarthritis (KOA) is a highly prevalent, chronic joint disorder, and it is a typical disease which can develop chronic pain. Our previous study has proved that endocannabinoid (2-AG)-CB1R-GABA-5-HT pathway is involved in electroacupuncture (EA) mediated inhibition of chronic pain. However, it is still unclear which among the 5-HT receptor subtype is involved in EA evoked 5-HT mediated inhibition of chronic pain in the dorsal spinal cord. 5-HT2A is a G protein-coupled receptor and it is involved in 5-HT descending pain modulation system. We found that EA treatment at frequency of 2 Hz +1 mA significantly increased the expression of 5-HT2A receptor in the dorsal spinal cord and intrathecal injection of 5-HT2A receptor antagonist or agonist reversed or mimicked the analgesic effect of EA in each case respectively. Intrathecal injection of a selective GABA(A) receptor antagonist Bicuculline also reversed the EA effect on pain hypersensitivity. Additionally, EA treatment reversed the reduced expression of GABA(A) receptor and KCC2 in the dorsal spinal cord of KOA mice. Furthermore, we demonstrated that intrathecal 5-HT2A receptor antagonist/agonist reversed or mimicked the effect of EA up-regulate of KCC2 expression, respectively. Similarly, intrathecal injection of PLC and PKC inhibitors prevented both anti-allodynic effect and up-regulation of KCC2 expression by EA treatment. Our data suggest that EA treatment up-regulated KCC2 expression through activating 5-HT2A-Gq-PLC-PKC pathway and enhanced the inhibitory function of GABA(A) receptor, thereby inhibiting chronic pain in a mouse model of KOA.

C1 Xi An Jiao Tong Univ, Hlth Sci Ctr, Dept Physiol & Pathophysiol, Sch Basic Med Sci,Inst Neurosci,Translat Med, Xian, Peoples R China; Xi An Jiao Tong Univ, Key Lab Environm & Genes Related Dis, Minist Educ, Xian, Peoples R China; Huazhong Univ Sci & Technol, Dept Neurobiol, Wuhan, Peoples R China; Huazhong Univ Sci & Technol, Key Lab Neurol Dis, Minist Educ, Inst Brain Res,Sch Basic Med,Tongji Med Coll, Wuhan, Peoples R China

DI 10.1177/17448069221087583

ID 224

ER

FN Clarivate Analytics Web of science

PT J

AU Choi, S.

AU - Park, S.

AU - Lim, Y. S.

AU - Park, T. Y.

AU - Do, K. S.

AU - Byun, S. H.

AU - Yoon, S. H.

AU - Lee, J. H.

PY 2022

TI A comparative study of a nerve block therapy with and without a deeply inserted acupotomy applied to hyeopcheok points for lumbosacral radiculopathy Safety, effectiveness, cost-effectiveness (a randomized controlled, two-arm, parallel study, pilot study, assessor-blind)

DA 2022/1/1

SO MEDICINE

JO MEDICINE

VL 101

IS 9

SN 0025-7974

Z9 Times Cited in Web of Science Core Collection: 2 Total Times Cited: 2 Cited Reference Count: 22 ER -

M3 10.1097/MD.0000000000028983

N1 ChoiPark-365

Y2 2025/1/18 16:03:00

N1 2025/2/13 1:16:00

DE acupotomy; lumbosacral radiculopathy; nerve block therapy; pilot study; randomized; KNEE OSTEOARTHRITIS; OUTCOMES; VERSION

AB Introduction: The prevalence of lumbosacral radiculopathy is estimated to be approximately 3% to 5% in patient populations. Lumbosacral radiculopathy is largely caused by a complex interaction between biomechanical and biochemical factors. Nerve block therapy (NBT) mainly treats lumbosacral radiculopathy by improving the biochemical factors, whereas acupotomy mainly focuses on improving the biomechanical factors. Therefore, it is thought that synergistic effects may be obtained for the treatment of lumbosacral radiculopathy when both NBT and acupotomy are combined. However, no study in China and Korea, where acupotomy is majorly provided, has reported the effects of such a combination treatment. Therefore, this study aimed to evaluate the safety, effectiveness, and cost-effectiveness of the concurrent use of a deeply inserted acupotomy and NBT for the treatment of lumbosacral radiculopathy. Methods/design: This is an open-label, parallel, assessor-blinded, randomized controlled trial, which will include 50 patients with lumbosacral radiculopathy. After patients voluntarily agree to participate in the study, they will be screened, and will undergo necessary examinations and tests according to the protocol. Those who satisfy the selection criteria will be randomly assigned to either the NBT + acupotomy or NBT groups in a 1:1 ratio. Both groups will undergo 2 NBTs once every 2 weeks from 1 week after the screening test. The treatment group will receive additional acupotomy twice a week for 4 weeks. The primary endpoint is the Oswestry Disability Index, whereas the secondary endpoints are the Numeral Rating Scale, European Quality of Life 5-dimension, McGill pain Questionnaire, Roland-Morris Disability Questionnaire, safety assessment, and economic feasibility evaluation. The measurements will be made at 0, 2, 4, and 8 weeks. Ethics and dissemination: This trial has received complete ethical approval from the Ethics Committee of Catholic Kwandong University International St. Mary's Hospital (IS20OISE0085). We intend to submit the results of the trial to a peer-reviewed journal and/or conferences.

C1 Catholic Kwandong Univ, Int St Marys Hosp, Dept Anesthesiol & Pain Med, Incheon, South Korea; Catholic Kwandong Univ, Int St Marys Hosp, Inst Integrat Med, Simgok Ro 100Gil 25, Incheon 22711, South Korea; S HEAL Pain & Korean Med Clin, Seoul, South Korea; Kyung Hee Univ, Grad Sch, Dept Appl Korean Med, Seoul, South Korea

DI 10.1097/MD.0000000000028983

ID 365

ER

FN Clarivate Analytics Web of science

PT J

AU Pang, JCY

AU - Fu, ASN

AU - Lam, SKH

AU - Peng, B.

AU - Fu, ACL

PY 2022

TI Ultrasound-guided dry needling versus traditional dry needling for patients with knee osteoarthritis: A double-blind randomized controlled trial

DA 2022/1/1

SO PLOS ONE

JO PLOS ONE

VL 17

IS 9

SN 1932-6203

Z9 Times Cited in Web of Science Core Collection: 5 Total Times Cited: 5 Cited Reference Count: 69 ER -

M3 10.1371/journal.pone.0274990

N1 PangFu-378

N1 2025/2/13 1:16:00

DE AUTOLOGOUS BLOOD INJECTION; HIP OSTEOARTHRITIS; VISUAL ANALOG; HEALTH-CARE; ACUPUNCTURE; PAIN; RECOMMENDATIONS; STIMULATION; RELIABILITY; DISABILITY

AB Objective To compare the effect of ultrasound (US)-guided dry needling (DN) with traditional DN in the treatment of pain and dysfunction for patients with knee osteoarthritis (KOA). Design A double-blind, randomized controlled trial. Methods Patients (25 male and 65 female), age 50-80 years diagnosed with KOA were recruited and randomly assigned to one of three groups in a 1:1:1 ratio for intervention: real US-guided DN with exercise therapy (G1), placebo US-guided DN with exercise therapy (G2), and exercise therapy solely (G3). G1 and G2 were blinded to the application of real or placebo US guidance by turning the monitor of US imaging out-of-view from participants' vantage points. The effectiveness of blinding was evaluated by asking the participants whether they had received real-US guided DN. The responses were assessed by Chi-square test. Visual Analogue Scale (VAS), Knee injury, and Osteoarthritis Outcome Score (KOOS) subscales (KOOS-pain, KOOS-symptoms, KOOS-quality-of-life (QoL)) were collected at baseline, 4 weeks, and 8 weeks by a blinded assessor. Data were analyzed by mixed model analysis of variance (ANOVA) with Bonferroni correction. Results Eighty-four participants (61.26 +/- 5.57 years) completed the study. G1 achieved significant improvement in VAS at 8 weeks compared to G2 and G3 (G1 vs. G2: MD = -15.61, 95% CI [-25.49, -5.51], p = 0.001; G1 vs. G3: MD = -19.90, 95% CI [-29.71, -10.08], p<0.001). G1 achieved significant improvement in KOOS-pain at 8 weeks compared to G2 and G3 (G1 vs. G2: MD = 9.76, 95% CI [2.38, 17.14], p = 0.006; G1 vs. G3: MD = 9.48, 95% CI [2.31, 16.66], p = 0.010). KOOS-symptoms and KOOS-QoL were not statistically significant between groups. G2 had no significant difference of the perceptions as G1 with p = 0.128. G2 were successfully blinded to placebo US-guided DN. Conclusion US-guided DN with exercise therapy may be more effective than traditional DN with exercise therapy or exercise therapy alone in reduce pain of KOA.

C1 Caritas Inst Higher Educ, Sch Hlth Sci, Hong Kong, Peoples R China; Hong Kong Polytech Univ, Dept Rehabil Sci, Hong Kong, Peoples R China; Hong Kong Inst Musculoskeletal Med, Hong Kong, Peoples R China; Chinese Univ Hong Kong, Dept Family Med, Hong Kong, Peoples R China; Univ Hong Kong, Dept Family Med, Hong Kong, Peoples R China; Univ Elect Sci & Technol China, Sichuan Translat Med Res Hosp, Chinese Acad, Dept Rehabil Med,Sichuan Prov Peoples Hosp, Chengdu, Sichuan, Peoples R China; Univ Sydney, Sydney Sch Hlth Sci, Discipline Physiotherapy, Sydney, NSW, Australia; Univ Sydney, Charles Perkins Ctr, Musculoskeletal Res Hub, Sydney, NSW, Australia; 2 Chui Ling Lane, Hong Kong, Peoples R China; Hong Kong Polytech Univ, ST 535, Hong Kong, Peoples R China; KOLOUR Tsuen Wan, Room 1201,12-F, Hong Kong, Peoples R China; Chinese Univ Hong Kong, Hong Kong, Peoples R China; Univ Hong Kong, Hong Kong, Peoples R China; Sichuan Acad Med Sci, Sichuan Prov Peoples Hosp, Chengdu, Sichuan, Peoples R China; Univ Sydney, Level 7,Susan Wakil Hlth Bldg D18, Sydney, NSW, Australia

DI 10.1371/journal.pone.0274990

ID 378

ER

FN Clarivate Analytics Web of science

PT J

AU Martins, G. A.

AU - Degen, A. N.

AU - Antunes, FTT

AU - Rosa, LGD

AU - Ferraz, A. G.

AU - Wiilland, E.

AU - Vieira, L. B.

AU - Souza, AHD

PY 2022

TI Benefits of electroacupuncture and a swimming association when compared with isolated protocols in an osteoarthritis model

PG 375-383

DA 2022/1/1

SO JOURNAL OF TRADITIONAL AND COMPLEMENTARY MEDICINE

JO JOURNAL OF TRADITIONAL AND COMPLEMENTARY MEDICINE

VL 12

IS 4

SN 2225-4110

Z9 Times Cited in Web of Science Core Collection: 0 Total Times Cited: 0 Cited Reference Count: 54 ER -

M3 10.1016/j.jtcme.2021.11.002

N1 MartinsDegen-394

Y2 2025/1/17 15:55:00

N1 2025/2/13 1:16:00

DE osteoarthritis; Pain behavior; Electroacupuncture; Swimming; Non-pharmacological approaches; RAT MODEL; KNEE OSTEOARTHRITIS; CARTILAGE DEGENERATION; PAIN; ARTHRITIS; EXERCISE; INFLAMMATION; MONOARTHRITIS; STIMULATION; INTENSITIES

AB Background and aim: Osteoarthritis (OA) is characterized by pain and inflammation. Electroacupuncture (EA) and swimming (SW) are non-pharmacological interventions recommended for treating OA. The study evaluated the benefits of electroacupuncture (EA) and swimming (SW) association when compared with isolated protocols in an OA rodent model. Experimental. Procedures: An ankle monoarthritis model was induced in rats by applying Complete Freund's Adjuvant (CFA). After seven days of induced OA, the groups were submitted to EA (ST36 and the GB 30 Acupoint), SW, or the EA + SW protocol. The nociceptive behavior was measured by the Von Frey test, the Cold Stimulation test, and the Paw Flick Immersion test. Inflammatory activity was evaluated by measuring TNF levels, myeloperoxidase, NAGase, immunological parameters and the histology from the subcutaneous tissue. Results: Compared to CFA group, EA decreased the nociceptive scores in the cold stimulation test (p < 0.05), and it also increased the latency time in thermal cold (p < 0.01) and heat hyperalgesia (p < 0.001). Also, EA reduced NAGase (p < 0.01). SW reduced the edema (p < 0.05) and did not increase the inflammatory infiltrates or congestion, neither in the histological measurements nor by analyzing the levels of TNF. The association of EA + SW decreased the neutrophils and the monocytes, MPO (p < 0.05), and the glutamate levels in the cerebrospinal fluid (CSF, p < 0.001). Conclusion: There were statistical differences between combination therapy and monotherapy as seen by the inflammatory parameters, which could be associate to the delay of the chronification osteoarthritis retardation. However, EA + SW did not show benefits when compared to isolated protocols in nociceptive behavior. (c) 2021 Center for Food and Biomolecules, National Taiwan University. Production and hosting by Elsevier Taiwan LLC. This is an open access article under the CC BY-NC-ND license (http://creativecommons.org/ licenses/by-nc-nd/4.0/).

C1 Univ Luterana Brasil, Postgrad Program Cellular & Mol Biol Hlth Sci, Canoas, Rio Grande do S, Brazil; Univ Luterana Brasil, Dept Pharmacol, Canoas, Rio Grande do S, Brazil; Univ Fed Minas Gerais, Inst Biol Sci, Dept Pharmacol, Belo Horizonte, Brazil; Univ Luterana Brasil, Ave Farroupilha,8001, BR-92425900 Canoas, Rio Grande do S, Brazil

DI 10.1016/j.jtcme.2021.11.002

ID 394

ER

FN Clarivate Analytics Web of science

PT J

AU Huang, C. H.

AU - Yeh, M. L.

AU - Chen, F. P.

AU - Wu, D. P.

PY 2022

TI Low-level laser acupuncture reduces postoperative pain and morphine consumption in older patients with total knee arthroplasty: A randomized placebo-controlled trial

PG 321-328

DA 2022/1/1

SO JOURNAL OF INTEGRATIVE MEDICINE-JIM

JO JOURNAL OF INTEGRATIVE MEDICINE-JIM

VL 20

IS 4

SN 2095-4964

Z9 Times Cited in Web of Science Core Collection: 9 Total Times Cited: 9 Cited Reference Count: 49 ER -

M3 10.1016/j.joim.2022.04.002

N1 HuangYeh-443

Y2 2025/1/17 15:46:00

N1 2025/2/13 1:16:00

DE Acupuncture; Morphine; Low-level laser; Older patients; Osteoarthritis; Pain; Total knee arthroplasty; Randomized controlled trial; ACUPOINT STIMULATION; HIP-ARTHROPLASTY; PREVALENCE; THERAPY; OSTEOARTHRITIS; ANALGESIA; EFFICACY

AB Background: Patients commonly develop postoperative pain after total knee arthroplasty (TKA). Acupuncture-related techniques and low-level laser therapy could be beneficial for pain management for older individuals.Objective: To examine the effect of low-level laser acupuncture (LA) in reducing postoperative pain, pain -related interference in daily life, morphine consumption, and morphine-related side effects in older patients with knee osteoarthritis who underwent TKA. Design, setting, participants and intervention: A single-blind randomized placebo-controlled trial was con-ducted. Patients (N = 82) were recruited and randomly assigned via a computer-generated list to the LA group or a placebo group. The LA group received low-level laser therapy at Sanyinjiao (SP6), Taixi (KI3), Kunlun (BL60), Fengshi (GB31), Futu (ST32) and Neiguan (PC6) after TKA, while the placebo acupuncture group received the same treatment procedure without laser energy output.Main outcome measures: The primary outcome was postoperative pain intensity, and it was measured at baseline and hours 2, 6, 10, 24, 48 and 72 after TKA. The secondary outcomes, including relative pain, postoperative pain-related interference in daily life and morphine consumption, were measured at hours 24, 48 and 72 after TKA.Results: Generalized estimating equations revealed significant between-group differences in pain inten-sity (P = 0.01), and trend differences in pain intensity for the LA group starting at hours 10 to 72 (P < 0.05) and morphine consumption at hours 48 and 72 (P < 0.05). The changes in pain-related interference in daily life were significant (P < 0.05) at 72 h, with the exception of the parameters for worst pain, mood, and sleep. Nausea and vomiting side effects from morphine had significant between-group differences at hours 10 and 24 (P < 0.05).Conclusion: Low-level LA gradually reduced older patients' postoperative pain intensity and morphine consumption within the first 72 h after their TKA for osteoarthritis. Low-level LA may have benefits as an adjuvant pain management technique for clinical care.Trial registration: ClinicalTrials.gov registration number NCT03995446.Please cite this article as: Huang CH, Yeh ML, Chen FP, Wu D. Low-level laser acupuncture reduces post-operative pain and morphine consumption in older patients with total knee arthroplasty: A randomized placebo-controlled trial. J Integr Med. 2022; 20(4): 321-328.(c) 2022 Shanghai Yueyang Hospital Affiliated to Shanghai University of Traditional Chinese Medicine. Published by Elsevier B.V. All rights reserved.

C1 Taipei Vet Gen Hosp, Dept Nursing, Taipei City 11217, Taiwan; Natl Taipei Univ Nursing & Hlth Sci, Sch Nursing, Taipei City 11219, Taiwan; Taipei Vet Gen Hosp, Ctr Tradit Med, Taipei City 11217, Taiwan; Natl Taipei Univ Nursing & Hlth Sci, Sch Nursing, Taipei City 11217, Taiwan; Sch Med, Taipei City 11217, Taiwan

DI 10.1016/j.joim.2022.04.002

ID 443

ER

FN Clarivate Analytics Web of science

PT J

AU Chiu, P. E.

AU - Fu, Z. H.

AU - Sun, J.

AU - Jian, G. W.

AU - Li, T. M.

AU - Chou, L. W.

PY 2022

TI Efficacy of Fu's Subcutaneous Needling in Treating Soft Tissue Pain of Knee Osteoarthritis: A Randomized Clinical Trial

DA 2022/1/1

SO JOURNAL OF CLINICAL MEDICINE

JO JOURNAL OF CLINICAL MEDICINE

VL 11

IS 23

SN 2077-0383

Z9 Times Cited in Web of Science Core Collection: 17 Total Times Cited: 17 Cited Reference Count: 57 ER -

M3 10.3390/jcm11237184

N1 ChiuFu-447

N1 2025/2/13 1:16:00

DE physiotherapy; muscle pain; dry needling; Fu's subcutaneous needling; acupuncture; knee osteoarthritis; myofascial pain; trigger point; tightened muscle; MCMASTER UNIVERSITIES OSTEOARTHRITIS; QUALITY-OF-LIFE; SYMPTOMATIC KNEE; WESTERN ONTARIO; MUSCLE TONE; ASSOCIATION; DISORDERS; STIFFNESS; QUANTIFICATION; RELIABILITY

AB Purpose: Fu's subcutaneous needling (FSN) is a new acupuncture technique that produces a long-lasting effect in soft-tissue injuries. In patients with degenerative knee osteoarthritis (OA), myofascial trigger points (MTrPs) are common in the lower-limb muscles. In this randomized clinical trial, we evaluated the immediate, 1-week and 2-week effectiveness of FSN therapy in the treatment of degenerative knee OA. Patients and methods: We randomly divided 32 patients with knee OA into the FSN group (mean age: 65.73 +/- 6.79 years) or the transcutaneous electrical nerve stimulation (TENS) group (mean age: 62.81 +/- 5.72 years). The pressure pain threshold (PPT) and tissue hardness (TH) of the muscle and tendon attachment sites, knee range of motion, and physical ability (average walking speed) were measured. The subjective pain intensity index, Western Ontario and McMaster Universities OA Index (WOMAC), and Lequesne index were used to determine the efficacy of FSN on MTrP-induced soft-tissue pain compared with that of TENS. Results: A significantly greater improvement in pain qualities in the VAS (p < 0.05) was found in the FSN group. Moreover, in muscle and tendon qualities (including PPT and TH), a significant difference in the PPT of the quadriceps muscle (p < 0.05) was also observed among the immediate treatments in the FSN group. As for the functional index questionnaire assessment, the FSN group exhibited significant improvements among the immediate, 1-week and 2-week efficacies in terms of WOMAC (p < 0.05) and Lequesne index scores (p < 0.05). Conclusion: FSN was effective in treating soft-tissue pain in degenerative knee OA in terms of alleviating pain, strengthening walking ability, and improving overall functional performance. Pain relief was the primary benefit of FSN and a significant correlation between pain relief and knee joint mobility improvement was found. Trial registration: ClinicalTrials.gov Protocol Registration and Results System (registration number: NCT04356651).

C1 Chang Bing Show Chwan Mem Hosp, Dept Chinese Med, Changhua 505029, Taiwan; China Med Univ, Grad Inst Integrated Med, Coll Chinese Med, Taichung 404333, Taiwan; Beijing Univ Chinese Med, Inst Fus Subcutaneous Needling, Beijing 100029, Peoples R China; Guangzhou Univ Chinese Med, Clin Med Coll Acupuncture & Moxibust & Rehabil, Guangzhou 510405, Peoples R China; Guangzhou Univ Chinese Med, Clin Med Coll 2, Guangzhou 510006, Peoples R China; Guangzhou Univ Chinese Med, Affiliated Hosp 2, Guangdong Prov Hosp Chinese Med, Guangzhou 510260, Peoples R China; China Med Univ, Grad Inst Acupuncture Sci, Taichung 404333, Taiwan; Minist Hlth & Welf, Sinying Hosp, Dept Chinese Med, Tainan, Taiwan; China Med Univ, Coll Chinese Med, Sch Chinese Med, Taichung 404333, Taiwan; China Med Univ Hosp, Dept Phys Med & Rehabil, Taichung 404332, Taiwan; China Med Univ, Grad Inst Rehabil Sci, Dept Phys Therapy, Taichung 406040, Taiwan; Asia Univ, Asia Univ Hosp, Dept Phys Med & Rehabil, Taichung 413505, Taiwan

DI 10.3390/jcm11237184

ID 447

ER

FN Clarivate Analytics Web of science

PT J

AU Tian, X. D.

AU - Li, X.

AU - Zhou, L. Q.

AU - Zhao, J. P.

AU - Li, X. M.

AU - Huang, Y.

AU - Ding, T. S.

PY 2022

TI On the Effect of Electroacupuncture in Promoting Healing after High Tibial Osteotomy

DA 2022/1/1

SO COMPUTATIONAL AND MATHEMATICAL METHODS IN MEDICINE

JO COMPUTATIONAL AND MATHEMATICAL METHODS IN MEDICINE

VL 2022

SN 1748-670X

Z9 Times Cited in Web of Science Core Collection: 4 Total Times Cited: 4 Cited Reference Count: 38 ER -

M3 10.1155/2022/6428759

N1 TianLi-463

Y2 2025/1/18 22:03:00

N1 2025/2/13 1:16:00

DE ELECTRICAL-STIMULATION; DOUBLE-BLIND; OSTEOARTHRITIS; NONUNION; CURRENTS; FIELDS

AB Purpose. To explore the clinical effect of electroacupuncture in promoting the healing of the osteotomy area after high tibial osteotomy. Methods. 50 patients with knee osteoarthritis who underwent open wedge high tibial osteotomy (OWHTO) were selected and randomly divided into the observation group and control group. The control group got the common postoperative treatment, and the observation group was added electroacupuncture from the 3rd day after the operation on the basis of the control group. The electroacupuncture acupoints were selected SP10, ST34, ST32, EX-LE2, ST40,KI6, KI3, SP6, and ST41, once a day, and 14 days were a course of treatment. And then we contrasted the index of the Lane-Sandhu X-ray score, the skin incision healing time, the swelling subsided time, Visual Analogue Scale (VAS), Western Ontario and McMaster Universities Osteoarthritis Index Score (WOMAC), and Lysholm in different time. Results. The Lane-Sandhu X-ray score of the observation group was better than that of the control group at all time points (P < 0.05), and the time to achieve bone healing was about 2 weeks earlier than that of the control group. The skin healing and swelling were the subsided time in the osteotomy area. Both were better than the control group, and the difference was statistically significant (P < 0.05). The VAS score, WOMAC score, and Lysholm score of the two groups were significantly improved compared with preoperatively, and the difference was statistically significant (P < 0.05). The improvement of the observation group's VAS score, WOMAC score, and Lysholm score at 1 week, 4 weeks, and 8 weeks after the end of the treatment course was better than that of the control group, and the difference was statistically significant (P < 0.05). Conclusion. Electroacupuncture can quicken the healing of bone tissue and surrounding soft tissues in the osteotomy area after high tibial osteotomy, and at the same time, it can help the relief of knee joint pain and improve knee joint function.

C1 Beijing Univ Chinese Med, Dongzhimen Hosp, Beijing 100700, Peoples R China; Beijing Univ Chinese Med, Wudang Med Inst, Beijing 100029, Peoples R China; Beijing Univ Chinese Med, Affiliated Hosp 3, Beijing 100029, Peoples R China

DI 10.1155/2022/6428759

ID 463

ER

FN Clarivate Analytics Web of science

PT J

AU Liu, W.

AU - Fan, Y. H.

AU - Wu, Y. H.

AU - Hou, X.

AU - Xue, B.

AU - Li, P. H.

AU - Zhang, S. M.

AU - Yue, Q. Y.

PY 2021

TI Efficacy of Acupuncture-Related Therapy in the Treatment of Knee Osteoarthritis: A Network Meta-Analysis of Randomized Controlled Trials

PG 2209-2228

DA 2021/1/1

SO JOURNAL OF PAIN RESEARCH

JO JOURNAL OF PAIN RESEARCH

VL 14

SN 1178-7090

Z9 Times Cited in Web of Science Core Collection: 24 Total Times Cited: 28 Cited Reference Count: 63 ER -

M3 10.2147/JPR.S315956

N1 LiuFan-7

Y2 2025/1/18 21:53:00

N1 2025/2/13 1:16:00

DE knee osteoarthritis; acupuncture; moxibustion; randomized controlled trials; network meta-analysis; PAIN

AB Objective: Knee osteoarthritis (KOA) is prevalent in middle-aged and elderly people. This condition negatively affects the quality of life of patients. Although non-steroidal antiinflammatory drugs (NSAIDs) are often used to relieve symptoms associated with KOA, it is associated with many side effects. Acupuncture and moxibustion therapies have been applied in the treatment of KOA. However, the efficacy of various acupuncture and moxibustion treatments has not been compared. Methods: Randomized controlled trials (RCTs) on the application of acupuncture and moxibustion in the treatment of KOA were searched in English databases and Chinese databases. Data were retrieved from establishment of the database to September 2020. Data analysis was performed using Stata14.0 and GeMTC 0.14.3 softwares. Results: A total of 40 RCTs involving 3215 patients with KOA were retrieved. Network meta-analysis revealed that the fire needle was superior to western medicine, electro-acupuncture, conventional acupuncture, warm needle and sham acupuncture; warm needle was better than conventional acupuncture and western medicine whereas electro-acupuncture was better than conventional acupuncture in improving pain scores in the Western Ontario and McMaster Universities Osteoarthritis Index (WOMAC). Moreover, we found that fire needle and warm needle more effectively improved WOMAC stiffness scores than western medicine and sham moxibustion, whereas electro-acupuncture was superior to western medicine and sham moxibustion in improving WOMAC stiffness scores. Further analysis revealed that fire needle, warm needle and electro-acupuncture were more effective in improving WOMAC joint function scores than conventional acupuncture and western medicine. The fire needle was superior to conventional acupuncture and sham acupuncture, whereas electroacupuncture was better than western medicine, conventional acupuncture and sham acupuncture in improving visual analogue scale scores. Conclusion: This study shows that fire needle is superior to warm needle and electroacupuncture, whereas warm needle and electro-acupuncture are better than conventional acupuncture, western medicine, sham moxibustion and sham acupuncture.

C1 Univ Tradit Chinese Med, Teaching Hosp Tianjin 1, Tianjin 300193, Peoples R China; Natl Clin Res Ctr Chinese Med Acupuncture & Moxib, Tianjin 300381, Peoples R China; Shandong First Med Univ, Shandong Prov Hosp, Dept Endocrinol & Metab Dis, Jinan 250021, Shandong, Peoples R China

DI 10.2147/JPR.S315956

ID 7

ER

FN Clarivate Analytics Web of science

PT J

AU Liu, J. W.

AU - Wu, Y. L.

AU - Wei, W.

AU - Zhang, Y. L.

AU - Liu, D.

AU - Ma, X. X.

AU - Li, C.

AU - Ma, Y. Y.

PY 2021

TI Effect of Warm Acupuncture Combined with Bone Marrow Mesenchymal Stem Cells Transplantation on Cartilage Tissue in Rabbit Knee Osteoarthritis

DA 2021/1/1

SO EVIDENCE-BASED COMPLEMENTARY AND ALTERNATIVE MEDICINE

JO EVIDENCE-BASED COMPLEMENTARY AND ALTERNATIVE MEDICINE

VL 2021

SN 1741-427X

Z9 Times Cited in Web of Science Core Collection: 10 Total Times Cited: 15 Cited Reference Count: 45 ER -

M3 10.1155/2021/5523726

N1 LiuWu-19

N1 2025/2/13 1:16:00

DE CHONDROCYTE APOPTOSIS; GROWTH-FACTOR; INFLAMMATION; INJECTIONS; RATS

AB The current study was designed to investigate the effect and underlying mechanism of warm acupuncture combined with bone marrow mesenchymal stem cells (BMSC) transplantation on cartilage tissue injury in rabbit knee osteoarthritis (KOA). In the study, 50 rabbits were randomly divided into 5 groups: blank group, KOA group, warm acupuncture group, BMSCs group, and warm acupuncture combined with BMSCs group. After warm acupuncture combined with BMSCs, the Modified Lequesne MG knee joint assessment scale was used to evaluate the degree of knee joint behavior, the Taiping Peng method generally observed the histomorphology changes of KOA rabbit cartilage, and hematoxylin-eosin staining, safranin O green staining, and toluidine blue staining were conducted to evaluate the extent of cartilage tissue pathology. Furthermore, transmission electron microscopy and TUNEL staining were used to observe cell apoptosis, and immunohistochemistry and qPCR analysis were used to detect the expression of apoptosis-related proteins and mRNA. Results showed that administration of warm acupuncture combined with BMSCs recovered the joint function and significantly decreased Lequesne MG score. The degree of cartilage tissue pathological damage has been improved, cartilage ultrastructure degeneration has recovered, peripheral blood vessels have mild edema, blood supply has gradually recovered, and even small amounts of red blood cells have appeared. In addition, warm acupuncture combined with BMSCs treatment suppressed chondrocyte apoptosis in rabbits with knee osteoarthritis by reduced TUNEL-positive chondrocytes and simultaneously reversed the mRNA expression of Bax, Bcl-2, and Caspase-3. These results indicate that warm acupuncture combined with BMSCs transplantation has a potential protective effect on rabbit KOA, which may be mediated by inhibiting chondrocyte apoptosis.

C1 Ningxia Med Univ, Dept Tradit Chinese Med Orthoped & Traumatol, Gen Hosp, Yinchuan 750004, Ningxia Hui Aut, Peoples R China; Ningxia Med Univ, Key Lab Hui Ethn Med Modernizat, Minist Educ, Yinchuan 750004, Ningxia Hui Aut, Peoples R China; Ningxia Med Univ, Yinchuan 750004, Ningxia Hui Aut, Peoples R China

DI 10.1155/2021/5523726

ID 19

ER

FN Clarivate Analytics Web of science

PT J

AU Qu, B.

AU - Wu, X. Y.

AU - Liu, H. P.

AU - Cai, W. X.

AU - Wang, G. Q.

AU - Song, H. B.

AU - Wang, F.

PY 2021

TI Meta-analysis and systematic review of acupotomy combined with puncture and moxibustion in the treatment of knee osteoarthritis

PG 6637-6649

DA 2021/1/1

SO ANNALS OF PALLIATIVE MEDICINE

JO ANNALS OF PALLIATIVE MEDICINE

VL 10

IS 6

SN 2224-5820

Z9 Times Cited in Web of Science Core Collection: 4 Total Times Cited: 4 Cited Reference Count: 29 ER -

M3 10.21037/apm-21-1083

N1 QuWu-21

N1 2025/2/13 1:16:00

DE Acupotomy; acupuncture and moxibustion; knee osteoarthritis (KOA); meta-analysis

AB Background: This study aimed to systematically evaluate the therapeutic effects of acupotomy combined with acupuncture and moxibustion on knee osteoarthritis (KOA), which was expected to provide a reference for clinical treatment of KOA using traditional Chinese medicine (TCM). Methods: The databases PubMed, Embase, Medline, Ovid, and Springer were searched to retrieve randomized controlled trials ( RCTs) on KOA treatment by acupotomy combined with acupuncture and moxibustion. The search time was set as from the date the database was established to 31 December 2020. The Cochrane Handbook for Systematic Reviews of Intervention 5.0.2 was used to conduct bias risk assessment on the included literature, and Review Manager 5.3 software was used for meta-analysis. Results: A total of 10 RCTs were included in this study, including 1,073 participants. Meta-analysis results showed that compared with the control group, the clinical treatment efficiency of the experimental group was higher [mean difference (MD) =5.72; 95% confidence interval (CI): 3.39 to 9.64; Z=6.54; P<0.00001], and the postoperative visual analogue scale (VAS) scores were reduced (MD =-1.72; 95% CI: -2.41 to -1.03; Z=4.86; P<0.00001). Discussion: Acupotomy combined with acupuncture and moxibustion treatment for KOA can increase clinical treatment efficiency, and relieve postoperative pain, suggesting that the combination of acupotomy, acupuncture, and moxibustion has better therapeutic effects on KOA and can be promoted clinically.

C1 Heilongjiang Univ Chinese Med, Affiliated Hosp 1, Div CT & MRI, Harbin, Peoples R China; Heilongjiang Univ Tradit Chinese Med, Basic Med Sch, Harbin, Peoples R China; Heilongjiang Univ Chinese Med, Affiliated Hosp 2, Orthoped 3, Harbin, Peoples R China; Heilongjiang Univ Chinese Med, Affiliated Hosp 1, Orthoped 3, Harbin, Peoples R China; Heilongjiang Univ Chinese Med, Affiliated Hosp 1, Orthoped 4, Harbin, Peoples R China

DI 10.21037/apm-21-1083

ID 21

ER

FN Clarivate Analytics Web of science

PT J

AU Wang, T.

AU - Guo, Y.

AU - Shi, X. W.

AU - Gao, Y.

AU - Zhang, J. Y.

AU - Wang, C. J.

AU - Yang, X.

AU - Shu, Q.

AU - Chen, X. L.

AU - Fu, X. Y.

AU - Xie, W. S.

AU - Zhang, Y.

AU - Li, B.

AU - Guo, C. Q.

PY 2021

TI Acupotomy Contributes to Suppressing Subchondral Bone Resorption in KOA Rabbits by Regulating the OPG/RANKL Signaling Pathway

DA 2021/1/1

SO EVIDENCE-BASED COMPLEMENTARY AND ALTERNATIVE MEDICINE

JO EVIDENCE-BASED COMPLEMENTARY AND ALTERNATIVE MEDICINE

VL 2021

SN 1741-427X

Z9 Times Cited in Web of Science Core Collection: 10 Total Times Cited: 10 Cited Reference Count: 59 ER -

M3 10.1155/2021/8168657

N1 WangGuo-41

N1 2025/2/13 1:16:00

DE KNEE OSTEOARTHRITIS; REDUCES CARTILAGE; MODEL; BISPHOSPHONATES; EXPRESSION; LESIONS; DAMAGE

AB Subchondral bone lesions, as the crucial inducement for accelerating cartilage degeneration, have been considered as the initiating factor and the potential therapeutic target of knee osteoarthritis (KOA). Acupotomy, the biomechanical therapy guided by traditional Chinese meridians theory, alleviates cartilage deterioration by correcting abnormal mechanics. Whether this mechanical effect of acupotomy inhibits KOA subchondral bone lesions is indistinct. This study aimed to investigate the effects of acupotomy on inhibiting subchondral bone resorption and to define the possible mechanism in immobilization-induced KOA rabbits. After KOA modeling, 8 groups of rabbits (4w/6w acupotomy, 4w/6w electroacupuncture, 4w/6w model, and 4w/6w control groups) received the indicated intervention for 3 weeks. Histological and bone histomorphometry analyses revealed that acupotomy prevented both cartilage surface erosion and subchondral bone loss. Further, acupotomy suppressed osteoclast activity and enhanced osteoblast activity in KOA subchondral bone, showing a significantly decreased expression of tartrate-resistant acid phosphatase (TRAP), matrix metalloproteinases-9 (MMP-9), and cathepsin K (Ctsk) and a significantly increased expression of osteocalcin (OCN); this regulation may be mediated by blocking the decrease in osteoprotegerin (OPG) and the increase in NF-kappa B receptor activated protein ligand (RANKL). These findings indicated that acupotomy inhibited osteoclast activity and promoted osteoblast activity to ameliorate hyperactive subchondral bone resorption and cartilage degeneration in immobilization-induced KOA rabbits, which may be mediated by the OPG/RANKL signaling pathway. Taken together, our results indicate that acupotomy may have therapeutic potential in KOA by restoring the balance between bone formation and bone resorption to attenuate subchondral bone lesions.

C1 Beijing Univ Chinese Med, Sch Acupuncture Moxibust & Tuina, Beijing 100029, Peoples R China; Capital Med Univ, Beijing Hosp Tradit Chinese Med, Acupuncture & Moxibust Dept, Beijing 100010, Peoples R China; Beijing Univ Chinese Med, Affiliated Hosp 3, Massage Dept, Beijing 100029, Peoples R China; Beijing Nankou Hosp, Tradit Chinese Med Dept, Beijing 102200, Peoples R China

DI 10.1155/2021/8168657

ID 41

ER

FN Clarivate Analytics Web of science

PT J

AU Deng, X. L.

AU - Li, B. R.

AU - Hou, X. J.

AU - Xu, X.

AU - Xiong, W.

PY 2021

TI Evaluation of efficiency and safety of muscular region's acupuncture treatments for knee osteoarthritis A protocol for systematic review and network meta-analysis

DA 2021/1/1

SO MEDICINE

JO MEDICINE

VL 100

IS 31

SN 0025-7974

Z9 Times Cited in Web of Science Core Collection: 1 Total Times Cited: 1 Cited Reference Count: 13 ER -

M3 10.1097/MD.0000000000026810

N1 DengLi-57

N1 2025/2/13 1:16:00

DE acupuncture; knee osteoarthritis; network meta-analysis; protocol

AB Background: As a chronic degenerative disease, knee osteoarthritis (KOA) is mainly characterized by loose ligaments around the knee joint, degeneration of cartilage in the knee joint, and atrophy of surrounding muscles. According to related investigations, the incidence of knee osteoarthritis in China is 8.1%, of which 10.3% are women and 5.7% are men. Therefore, in order to improve the therapeutic effect of KOA, we must constantly explore new ways to treat the disease. The purpose of this study is to evaluate the effectiveness and safety of acupuncture with needle knife, blade needle, long-round needle, fire needle, micro-needle knife with conventional acupuncture intervention in KOA. Methods: Computer search of PubMed, Cochrane Library, Web of Science, CNKI, Wangfang, and VIP database, search for randomized controlled trials of muscular region's acupuncture therapy on KOA, the search time limit is to build the database until July 9, 2021. To ensure the comprehensiveness of the search, relevant references and conference literature are also included. After 2 researchers independently screened the literature, extracted data, and evaluated the risk of bias in the included studies, the Stata 14.0 software was used for data analysis. Results: The effectiveness and safety of muscular region's acupuncture in the treatment of patients with KOA will be systematically evaluated. Conclusion: The results of this study will provide strong evidence to determine whether muscular region's acupuncture is effective in the treatment of KOA. Registration number: INPLASY202170031 ()

C1 Nanchang Hongdu Hosp Tradit Chinese Med, 1399 Diezihu Rd, Nanchang 330008, Jiangxi, Peoples R China

DI 10.1097/MD.0000000000026810

ID 57

ER

FN Clarivate Analytics Web of science

PT J

AU Xu, D. H.

AU - Lee, M. H.

AU - Huang, C. H.

AU - Wei, J.

AU - Zhou, M. X.

AU - Yao, T. T.

AU - Lu, J. J.

AU - Zhao, W. J.

AU - Xu, N.

AU - Huang, R. N.

AU - He, J.

AU - Zheng, L.

PY 2021

TI Effect of acupotomy in knee osteoarthritis patients: study protocol for a randomized controlled trial

DA 2021/1/1

SO TRIALS

JO TRIALS

VL 22

IS 1

SN 1745-6215

Z9 Times Cited in Web of Science Core Collection: 5 Total Times Cited: 6 Cited Reference Count: 48 ER -

M3 10.1186/s13063-021-05247-z

N1 XuLee-64

N1 2025/2/13 1:16:00

DE Acupotomy; Needle-knife; Knee osteoarthritis; Study protocol; Randomized controlled trial; THERAPY; PAIN; HIP; MANAGEMENT; RECOMMENDATIONS; METAANALYSIS; HEALTH

AB BackgroundSymptomatic knee osteoarthritis (KOA) is common in China. Pharmacological therapy is not the first recommendation because of its safety issues. Nonpharmacological therapy, such as lifestyle adjustments, weight loss, muscle strengthening, and aerobic exercise programs, is strongly recommended for KOA. However, these approaches may fail due to poor patient compliance. There is a lack of high-quality randomized controlled trials of acupotomy, an effective treatment for KOA. This study was designed to investigate the efficacy of acupotomy in patients with KOA.MethodsA total of 136 patients will be enrolled at the First Affiliated Hospital of Guangzhou University of Chinese Medicine and assigned to the acupotomy group or sham acupotomy group according to the block randomization scheme. Patients in the acupotomy group will receive 2 sessions of acupotomy for 2weeks (once a week). Patients in the sham group will receive 2 sessions of sham stimulation for 2weeks (once a week). All patients will use indomethacin cream externally. The primary outcome will be the Western Ontario and McMaster Universities Osteoarthritis Index (WOMAC), and the secondary outcomes will be the visual analog scale (VAS) score, plantar pressure distribution test result, X-ray examination findings, musculoskeletal ultrasound findings, maximum knee circumference, joint mobility, and quality of life. Measurements will be taken at baseline, 1 week after the end of treatment, and at the 3- and 6-month follow-ups.DiscussionTo the best of our knowledge, this will be the first single-blind, sham-controlled study of acupotomy. The outcome assessors will also be blinded. The aim of this work is to demonstrate the efficacy of acupotomy in treating KOA.Trial registrationChinese Clinical Trial Registry ChiCTR2000033047. Registered on 18 May 2020.

C1 Guangzhou Univ Chinese Med, Affiliated Hosp 1, Guangzhou, Peoples R China; Guangzhou Univ Chinese Med, Clin Med Coll 1, Guangzhou, Peoples R China; Kyoto Prefectural Univ Med, Kyoto, Japan; Shenzhen Pingle Orthopaed Hosp, Shenzhen, Peoples R China; Univ Alabama Birmingham, Birmingham, AL USA; Sun Yat Sen Univ, Affiliated Hosp 8, Shenzhen, Peoples R China

DI 10.1186/s13063-021-05247-z

ID 64

ER

FN Clarivate Analytics Web of science

PT J

AU Li, S. M.

AU - Li, T. L.

AU - Guo, R.

AU - Chen, P.

AU - Du, W. S.

AU - Kang, S. B.

AU - Yan, M. Z.

AU - Cheng, W. Z.

PY 2021

TI Effectiveness and safety of acupotomy for knee osteoarthritis: study protocol for a randomized controlled trial

DA 2021/1/1

SO TRIALS

JO TRIALS

VL 22

IS 1

SN 1745-6215

Z9 Times Cited in Web of Science Core Collection: 4 Total Times Cited: 4 Cited Reference Count: 32 ER -

M3 10.1186/s13063-021-05786-5

N1 LiLi-74

N1 2025/2/13 1:16:00

DE Knee osteoarthritis; Acupotomy; NSAIDs; Randomized controlled trial; RISK-FACTORS; THERAPY; PAIN; HIP; HEALTH; PREVALENCE; OUTCOMES; HAND

AB Background: Knee osteoarthritis (KOA) is one of the most common musculoskeletal disorders. Acupotomy may be effective for KOA, but the evidence is limited. This trial aims to determine the effectiveness and safety of acupotomy for KOA. Methods/design: This is a parallel-group, assessor-blinded randomized controlled trial. Two hundred patients with KOA will be recruited and randomly assigned to two groups (group A or group D) in a 1:1 ratio. Patients in group A will receive acupotomy and topical diclofenac diethylamine for 4 weeks, while patients in group D will receive topical diclofenac diethylamine alone for 4 weeks. The primary outcome will be the response rate-the proportion of patients who achieve the minimal clinically important improvement in pain and function at week 4 compared with baseline. Secondary outcomes will include pain, function, quality of life, the use of rescue medicine (loxoprofen sodium), and adverse events at weeks 4, 8, and 24 after randomization. Besides, joint fluid and serum will be collected to assess the level of inflammatory cytokines, like TNF-alpha, IL-1 beta, and MMP-3. Discussion: This study will contribute to a better understanding of the effectiveness and safety of acupotomy in combination with topical nonsteroidal anti-inflammatory drugs. If the hypothesis is confirmed, acupotomy may be recommended as adjunctive therapy for patients with KOA. Results of the study will be of great importance for the guidelines of clinical therapy.

C1 Capital Med Univ, Beijing Hosp Tradit Chinese Med, Dept Pain, Beijing, Peoples R China; Beijing Univ Chinese Med, Dongzhimen Hosp, Beijing, Peoples R China; Beijing Univ Chinese Med, Sch Acupuncture Moxibust & Tuina, Beijing, Peoples R China; Capital Med Univ, Beijing Hosp Tradit Chinese Med, Dept Tuina, Beijing, Peoples R China

DI 10.1186/s13063-021-05786-5

ID 74

ER

FN Clarivate Analytics Web of science

PT J

AU Tong, J.

AU - Deng, C. Y.

AU - Sun, G. H.

AU - Zhou, J.

AU - Zhong, P. R.

AU - Wang, T. T.

AU - Zeng, Y.

AU - Wu, Q.

AU - Liao, Y.

AU - Luo, F.

AU - Peng, T.

AU - Liao, Y.

PY 2021

TI Electroacupuncture Upregulates HIF-1α and SOX9 Expression in Knee Osteoarthritis

DA 2021/1/1

SO EVIDENCE-BASED COMPLEMENTARY AND ALTERNATIVE MEDICINE

JO EVIDENCE-BASED COMPLEMENTARY AND ALTERNATIVE MEDICINE

VL 2021

SN 1741-427X

Z9 Times Cited in Web of Science Core Collection: 4 Total Times Cited: 4 Cited Reference Count: 36 ER -

M3 10.1155/2021/2047097

N1 TongDeng-80

N1 2025/2/13 1:16:00

DE MANUAL ACUPUNCTURE; CELLS; DELIVERY

AB Electroacupuncture (EA) has been clinically used in knee osteoarthritis broadly and proved to be effective than other therapies with fewer side effects; however, the mechanism of electroacupuncture to work on cartilage remains unclear. In this study, we aimed to evaluate the effect of EA treatment on cartilage and the relationship between EA and proteins such as HIF-a and SOX9. EA (dilatational wave, 3-15 HZ, 1 mA) has been applied to bilateral Zusanli (ST36), Xuehai (SP10), Taixi (KI3), and Yanglingquan (GB34) of rats. Results showed that the cartilage of the knee osteoarthritis group had obvious damage and fissure formation while the EA group showed that the cartilage destruction was generally milder. In addition, the protein expression levels of HIF-1 alpha, and chondrogenic markers such as Sox9, and ACAN in the electroacupuncture group were higher than those in the ACLT group. Also, the extracellular matrix protein expression levels of MMP13 and ADAMTS5 were decreased in the EA group. These findings indicate that EA could alleviate the severity of knee osteoarthritis, and HIF-a and SOX9 may closely attribute to the treatment.

C1 Univ South China, Dept Rehabil, Affiliated Hosp 1, Hengyang 421001, Hunan, Peoples R China; Univ South China, Rehabil Lab, Affiliated Hosp 1, Hengyang 421001, Hunan, Peoples R China; Hunan Tradit Chinese Med Coll, Dept Anat, Zhuzhou 412000, Hunan, Peoples R China

DI 10.1155/2021/2047097

ID 80

ER

FN Clarivate Analytics Web of science

PT J

AU Hua, Z. J.

AU - Deng, H.

AU - Tang, H.

AU - Ruan, Z. Z.

AU - Wang, P.

AU - Zhang, M.

AU - Ma, H.

AU - Wang, P.

AU - Dong, C.

AU - Huang, Z. L.

AU - Hong, H. Q.

AU - Zhou, Q.

AU - Zhou, H.

AU - Cheng, C. C.

AU - Lin, W. Q.

AU - Zhang, C. R.

AU - Chen, D. C.

PY 2021

TI Clinical Study of Acupotomy for Knee Osteoarthritis Based on the Meridian-Sinew Theory: A Randomized Controlled Clinical Trial

DA 2021/1/1

SO EVIDENCE-BASED COMPLEMENTARY AND ALTERNATIVE MEDICINE

JO EVIDENCE-BASED COMPLEMENTARY AND ALTERNATIVE MEDICINE

VL 2021

SN 1741-427X

Z9 Times Cited in Web of Science Core Collection: 6 Total Times Cited: 6 Cited Reference Count: 24 ER -

M3 10.1155/2021/3987002

N1 HuaDeng-85

N1 2025/2/13 1:16:00

DE NONSTEROIDAL ANTIINFLAMMATORY DRUGS; METAANALYSIS

AB This study was performed to compare the effectiveness of acupotomy based on the meridian-sinew theory with acupotomy based on the anatomical theory in the treatment of knee osteoarthritis (KOA). A total of 124 patients with knee osteoarthritis were randomized into the meridian-sinew (MS) group (63 patients) and anatomy group (61 patients). In the MS group, acupotomy based on the meridian-sinew theory was performed. In the anatomy group, acupotomy based on anatomy was applied. Patients were subgrouped by TCM Constitutions. The Western Ontario and McMaster Universities (WOMAC) Osteoarthritis Index and visual analog scale (VAS) were used to evaluate treatment effectiveness. The results showed that VAS (F = 22.61, p<0.01) and WOMAC (F = 24.84, p<0.01) scores declined with time, and there was no significant difference between the two groups nor subgroups (Yang deficiency subgroup, Yin-Yang harmony subgroup, and the subgroup of the others). A total of 5 patients reported 6 cases of the minor adverse effect, and all patients achieved complete recovery without medical intervention. This study indicates that the effectiveness and safety of acupotomy based on the meridian-sinew theory are equivalent to that of acupotomy based on anatomy in KOA treatment.

C1 Daishan Community Hlth Serv Ctr, Nanjing 210042, Jiangsu, Peoples R China; Nanjing Univ Tradit Chinese Med, Nanjing 210029, Jiangsu, Peoples R China; Nanjing Univ Chinese Med, Nanjing Hosp Chinese Med, Nanjing 210029, Jiangsu, Peoples R China; Nanjing Univ Chinese Med, Nanjing Hosp Tradit Chinese Med, Dept Acupuncture & Moxibust, Nanjing 210001, Jiangsu, Peoples R China; Taihu Sanat Jiangsu Prov, Wuxi 214100, Jiangsu, Peoples R China; Guangdong Pharmaceut Univ, Sch Publ Hlth, Guangzhou, Guangdong, Peoples R China; Nanjing Univ Chinese Med, Affiliated Hosp 2, Dept Acupuncture & Moxibust, Nanjing 210017, Jiangsu, Peoples R China; Nanjing Med Univ, Nanjing Hosp 1, Nanjing 210006, Jiangsu, Peoples R China; Nanjing Integrated Tradit Chinese & Western Med H, Nanjing 210014, Jiangsu, Peoples R China

DI 10.1155/2021/3987002

ID 85

ER

FN Clarivate Analytics Web of science

PT J

AU Guo, J. M.

AU - Xiao, Y.

AU - Cai, T. Y.

AU - Wang, J. H.

AU - Li, B. L.

AU - Huang, L. L.

AU - Mao, X.

AU - Lai, X. Q.

AU - Zhu, Y. J.

AU - Zhang, Y. Q.

AU - Chen, S. Q.

AU - Su, Y. X.

PY 2021

TI Chinese Medicine Involving Triple Rehabilitation Therapy for Knee Osteoarthritis in 696 Outpatients: A Multi-Center, Randomized Controlled Trial

PG 729-736

DA 2021/1/1

SO CHINESE JOURNAL OF INTEGRATIVE MEDICINE

JO CHINESE JOURNAL OF INTEGRATIVE MEDICINE

VL 27

IS 10

SN 1672-0415

Z9 Times Cited in Web of Science Core Collection: 7 Total Times Cited: 8 Cited Reference Count: 22 ER -

M3 10.1007/s11655-021-3488-6

N1 GuoXiao-93

Y2 2025/1/18 23:04:00

N1 2025/2/13 1:16:00

DE knee osteoarthritis; Chinese medicine involving triple rehabilitation therapy; electro-acupuncture; Chinese medicinal herb fumigating-washing; traditional exercises; community rehabilitation; EFFICACY

AB Objective: To determine the effects of Chinese medicine (CM) involving triple rehabilitation therapy on the progression of knee osteoarthritis (KOA). Methods: A total of 722 patients recruited from 38 community health service centers located in China from March 2013 to March 2017 were randomly divided into treatment and control groups equally, using a cluster randomization design. Health education combined with CM involving triple rehabilitation therapy for KOA (electro-acupuncture, Chinese medicinal herb fumigating-washing, and traditional exercises) was administered in the treatment group while conventional rehabilitation therapy (physical factor therapy, joint movement training, and muscle strength training) was administered in the control group. Patients with a visual analog scale (VAS) scores >= 4 were treated with dispersible meloxicam tablets (7.5 mg, once daily). The Lequesne index scores, VAS scores, range of motion (ROM), lower limb muscle strength, knee joint circumference, quantitative scores of KOA symptoms, and the short-form 36 item health survey questionnaire (SF-36) scores were measured for each patient at 5 checkpoints (before treatment, at the 2nd week and the 4th week during the 4-week treatment period, at 1 month and 3 months after end of treatment), and adverse reactions were observed also. Results: A total of 696 patients completed the entire process, with 351 in the treatment group and 345 in the control group. At all treatment checkpoints, the treatment group demonstrated better outcomes than the control group with regard to the total Lequesne index scores, effective rate and improvement rate of the total Lequesne index scores, VAS scores, lower limb muscle strength, knee circumference, quantitative scores of KOA symptoms, and SF-36 scores as well (P < 0.05 or P < 0.01). No adverse reactions were encountered in this study. Conclusions: CM involving triple rehabilitation therapy can alleviate KOA-related pain and swelling, improve lower limb muscle strength, promote flexion and activity of the knee and improve the quality of life in patients undergoing KOA. It is suitable for patients with early or mid-stage KOA.

C1 Fujian Univ Tradit Chinese Med, Dept Rehabil Med, Fuzhou 350122, Peoples R China; Fujian Hlth Coll, Dept Clin Med, Fuzhou 350101, Peoples R China; Fujian Univ Tradit Chinese Med, Dept Tradit Chinese Med, Fuzhou 350122, Peoples R China; Fujian Tingzhou Hosp, Dept Rehabil, Longyan 366300, Fujian, Peoples R China; Xiaogan Chinese Med Hosp, Dept Emergency, Xiaogan 432100, Hubei, Peoples R China; Fuzhou First Hosp, Dept Rehabil, Fuzhou 350009, Peoples R China; Fujian Univ Tradit Chinese Med, Fuzhou 350122, Peoples R China

DI 10.1007/s11655-021-3488-6

ID 93

ER

FN Clarivate Analytics Web of science

PT J

AU Liu, M.

AU - Liu, MNA

AU - Zhang, H. T.

AU - Peng, G. R.

AU - Sun, X. B.

AU - Zhu, X. Y.

AU - Zeng, Y. R.

PY 2021

TI Efficacy and safety of abdominal acupuncture for knee osteoarthritis A protocol for systematic review and meta-analysis

DA 2021/1/1

SO MEDICINE

JO MEDICINE

VL 100

IS 15

SN 0025-7974

Z9 Times Cited in Web of Science Core Collection: 0 Total Times Cited: 0 Cited Reference Count: 23 ER -

M3 10.1097/MD.0000000000023628

N1 LiuLiu-95

N1 2025/2/13 1:16:00

DE abdominal acupuncture; knee osteoarthritis; protocol; randomized controlled trial; RISK-FACTORS

AB Background: Knee osteoarthritis (KOA) is a disease based on degenerative pathological changes. Most commonly seen in the elderly and is one of Kenn's leading causes, its symptoms include swollen knees, pain in walking up and downstairs. If left untreated, it can lead to joint deformity and disability. Many clinical studies have reported that abdominal acupuncture has a good effect on KOA treatment, but there is no relevant systematic review. So the purpose of this study is to evaluate the effectiveness and safety of abdominal acupuncture in treating KOA. Methods: The following 8 electronic databases will be searched, including PubMed, Embase, the Cochrane Library, China National Knowledge Infrastructure (CNKI), Web of Science, Chinese Scientific Journal Database (VIP), Wanfang Database, and Chinese Biomedical Literatures Database (CBM) from their inception to November 1, 2020 without any restrictions. Researchers retrieve the literature and extracted the data, evaluation of research methods, quality of literature. The outcomes will include a Visual Analogue Scale. The Western Ontario and McMaster Universities Osteoarthritis Index, total effective rate, incidence of any adverse events. We use the Cochrane Risk of a bias assessment tool to evaluate methodological qualities. Data synthesis will be completed by RevMan 5.3.0. Results: We will show the results of this study in a peer-reviewed journal. Conclusions: This meta-analysis will provide reliable evidence for abdominal acupuncture treatment of KOA. INPLASY registration number: INPLASY2020110020.

C1 Guangzhou Univ Chinese Med, Clin Med Sch 1, Guangzhou, Guangdong, Peoples R China; Jiangxi Univ Tradit Chinese Med, Affiliated Hosp, Nanchang, Jiangxi, Peoples R China; Univ Chinese Med, Dept Orthopaed, Affiliated Hosp Guangzhou 1, Jichang Rd 16, Guangzhou 510405, Guangdong, Peoples R China

DI 10.1097/MD.0000000000023628

ID 95

ER

FN Clarivate Analytics Web of science

PT J

AU Ruan, A. M.

AU - Wang, Q. F.

AU - Ma, Y. F.

AU - Zhang, D.

AU - Yang, L. L.

AU - Wang, Z. P.

AU - Xie, Q.

AU - Yin, Y. S.

PY 2021

TI Efficacy and Mechanism of Electroacupuncture Treatment of Rabbits With Different Degrees of Knee Osteoarthritis: A Study Based on Synovial Innate Immune Response

DA 2021/1/1

SO FRONTIERS IN PHYSIOLOGY

JO FRONTIERS IN PHYSIOLOGY

VL 12

SN 1664-042X

Z9 Times Cited in Web of Science Core Collection: 15 Total Times Cited: 17 Cited Reference Count: 34 ER -

M3 10.3389/fphys.2021.642178

N1 RuanWang-100

N1 2025/2/13 1:16:00

DE electro-acupuncture; knee osteoarthritis; innate immune response; signaling pathway; mechanism research; ARTICULAR-CARTILAGE; INFLAMMATION; ACUPUNCTURE; ARTHRITIS; WEIGHT; SYSTEM; MODEL; RISK; PAIN

AB Knee osteoarthritis (KOA) is a chronic degenerative bone and joint disease, which is often clinically manifested as pain, joint swelling, and deformity. Its pathological manifestations are mainly synovial inflammation and cartilage degeneration. This study aims to investigate the efficacy of electro-acupuncture (EA) on model rabbits with varying degrees of KOA and to study the mechanism of EA on KOA based on the innate immune response. Mild and moderate rabbit KOA models were established using a modified Hluth method, and EA was given to both the mild and moderate model groups. The Lequesne-MG index was used to evaluate the behavioral changes in the rabbits before and after EA treatment. Morphological changes in the synovial membrane and cartilage of each group were observed by H&E staining. The Mankin scoring standard and the Krenn scoring standard were used to score the pathology of the cartilage tissue and synovial tissue, respectively. The inflammatory factors and metalloproteinases were detected in the serum of each group by ELISA. The protein and messenger RNA (mRNA) expressions of important elements related to Toll-like receptors (TLRs)-mediated innate immune response in the synovial tissue were detected by Western blot and quantitative PCR (qPCR). The Lequesne-MG index score of the rabbits gradually increased with the modeling prolonged but decreased significantly after EA treatment, indicating that EA has a better effect on alleviating the pain and improving the dysfunction. The morphological analysis showed that the inflammation of and the damage to the synovial membrane and the cartilage tissue gradually deteriorated with the modeling prolonged. However, the synovial membrane inflammation was significantly relieved after EA treatment, and the cartilage injury showed signs of repair. The ELISA analysis showed that, with the modeling prolonged, the serum-related inflammatory factors and mechanism of metalloproteinases gradually increased but decreased after EA treatment. The tumor necrosis factor alpha (TNF-alpha), interleukin 6 (IL-6), and matrix metalloproteinase3 (MMP3) of EA1 group were significantly lower than those of EA2 group. Both Western blot and qPCR results showed that the protein and mRNA expressions of the elements related to the innate immune response in the synovial membrane increased gradually with the modeling prolonged, but decreased significantly after EA treatment. Additionally, the expression of some components in EA1 group was significantly lower than that in EA2 group. These results confirm that synovial inflammation gradually aggravated with time from the early to mid-stage of KOA. EA alleviated the inflammation and histological changes in KOA rabbits by inhibiting the TLRs-mediated innate synovial immune response. This suggests that using EA in the early stage of KOA may achieve a desirable efficacy.

C1 Beijing Longfu Hosp, Dept Orthoped, Beijing, Peoples R China; Beijing Univ Chinese Med, Dept Tendon & Injury, Hosp 3, Beijing, Peoples R China; Beijing Univ Chinese Med, Dept Gen Surg, Hosp 2, Beijing, Peoples R China; Beijing Univ Chinese Med, Acupuncture & Moxibust Dept, Hosp 3, Beijing, Peoples R China; Beijing Univ Chinese Med, Grad Sch, Beijing, Peoples R China

DI 10.3389/fphys.2021.642178

ID 100

ER

FN Clarivate Analytics Web of science

PT J

AU Chen, J. X.

AU - Liu, A. F.

AU - Zhou, Q. X.

AU - Yu, W. J.

AU - Guo, T. C.

AU - Jia, Y. Z.

AU - Yang, K.

AU - Niu, P. Y.

AU - Feng, H. C.

PY 2021

TI Acupuncture for the Treatment of Knee Osteoarthritis: An Overview of Systematic Reviews

PG 8481-8494

DA 2021/1/1

SO INTERNATIONAL JOURNAL OF GENERAL MEDICINE

JO INTERNATIONAL JOURNAL OF GENERAL MEDICINE

VL 14

SN 1178-7074

Z9 Times Cited in Web of Science Core Collection: 16 Total Times Cited: 17 Cited Reference Count: 57 ER -

M3 10.2147/IJGM.S342435

N1 ChenLiu-105

N1 2025/2/13 1:16:00

DE acupuncture; knee osteoarthritis; overview; AMSTAR 2; GRADE; systematic review; HIP OSTEOARTHRITIS; SAFETY; CELECOXIB; EFFICACY; NAPROXEN; PAIN; ELECTROACUPUNCTURE; METAANALYSIS; IBUPROFEN; THERAPY

AB Background: Acupuncture has been widely used in the clinical management of osteoarthritis of the knee (KOA). Many systematic reviews (SRs) and meta-analyses (MAs) have reported its effectiveness in relieving pain. This overview aimed to summarize SRs and MAs on the effectiveness and safety of acupuncture for KOA and evaluate their methodological and evidence quality of the included SRs and MAs. Methods: We conducted a comprehensive literature search for SRs and MAs in four Chinese and four international databases from their inception until August 2021. Two researchers independently searched the reviews, extracted the data, and cross-checked the data. The Assessing the Methodological Quality of Systematic Reviews 2 (AMSTAR 2) tool was used to evaluate the methodological quality of the included SRs and MAs. The Grades of Recommendations, Assessment, Development, and Evaluation (GRADE) system was used to assess the quality of evidence for the outcomes of the included SRs and MAs. Results: A total of 14 SRs and MAs were included. The evaluation results of the AMSTAR 2 tool showed that the methodological quality of all the 14 SRs and MAs was critically low. The principal causes are the lack of a pre-registration proposal and a list of excluded studies and justify the exclusions, the report on the sources of funding, and the reasons for the study designs for inclusion. The results of the GRADE evaluation showed 25 of 46 outcomes were very low-level evidence. Seventeen were of low level, four were of moderate level and none were of high level. Most outcomes were downgraded in quality of evidence mainly because of publication bias and imprecision. Conclusion: The existing evidence suggests that acupuncture seems to be an effective and safe therapy for KOA. However, the deficiencies in the methodological quality and quality of evidence of the included SRs/MAs have limited the reliability of the conclusions. Therefore, further rigorous and comprehensive studies are warranted to verify the effectiveness and safety of acupuncture in KOA.

C1 Tianjin Univ Tradit Chinese Med, Dept Orthopaed Surg, First Teaching Hosp, 88 Changling Rd, Tianjin 300381, Peoples R China; Natl Clin Res Ctr Chinese Med Acupuncture & Moxib, Tianjin 300381, Peoples R China

DI 10.2147/IJGM.S342435

ID 105

ER

FN Clarivate Analytics Web of science

PT J

AU Tu, J. F.

AU - Yang, J. W.

AU - Shi, G. X.

AU - Yu, Z. S.

AU - Li, J. L.

AU - Lin, L. L.

AU - Du, Y. Z.

AU - Yu, X. G.

AU - Hu, H.

AU - Liu, Z. S.

AU - Jia, C. S.

AU - Wang, L. Q.

AU - Zhao, J. J.

AU - Wang, J.

AU - Wang, T.

AU - Wang, Y.

AU - Wang, T. Q.

AU - Zhang, N.

AU - Zou, X.

AU - Wang, Y.

AU - Shao, J. K.

AU - Liu, C. Z.

PY 2021

TI Efficacy of Intensive Acupuncture Versus Sham Acupuncture in Knee Osteoarthritis: A Randomized Controlled Trial

PG 448-458

DA 2021/1/1

SO ARTHRITIS & RHEUMATOLOGY

JO ARTHRITIS & RHEUMATOLOGY

VL 73

IS 3

SN 2326-5191

Z9 Times Cited in Web of Science Core Collection: 105 Total Times Cited: 128 Cited Reference Count: 47 ER -

M3 10.1002/art.41584

N1 TuYang-110

Y2 2025/1/20 13:41:00

N1 2025/2/13 1:16:00

DE MANUAL ACUPUNCTURE; HIP OSTEOARTHRITIS; COST-EFFECTIVENESS; CHRONIC PAIN; ELECTROACUPUNCTURE; MANAGEMENT; ANALGESIA; CONSENSUS

AB Objective. To assess the efficacy of intensive acupuncture (3 times weekly for 8 weeks) versus sham acupuncture for knee osteoarthritis (OA). Methods. In this multicenter, randomized, sham-controlled trial, patients with knee OA were randomly assigned to receive electroacupuncture (EA), manual acupuncture (MA), or sham acupuncture (SA) 3 times weekly for 8 weeks. Participants, outcome assessors, and statisticians were blinded with regard to treatment group assignment. The primary outcome measure was response rate, which is the proportion of participants who simultaneously achieved minimal clinically important improvement in pain and function by week 8. The primary analysis was conducted using a Z test for proportions in the modified intent-to-treat population, which included all randomized participants who had >= 1 post-baseline measurement. Results. Of the 480 participants recruited in the trial, 442 were evaluated for efficacy. The response rates at week 8 were 60.3% (91 of 151), 58.6% (85 of 145), and 47.3% (69 of 146) in the EA, MA, and SA groups, respectively. The between-group differences were 13.0% (97.5% confidence interval [97.5% CI] 0.2%, 25.9%; P = 0.0234) for EA versus SA and 11.3% (97.5% CI -1.6%, 24.4%; P = 0.0507) for MA versus SA. The response rates in the EA and MA groups were both significantly higher than those in the SA group at weeks 16 and 26. Conclusion. Among patients with knee OA, intensive EA resulted in less pain and better function at week 8, compared with SA, and these effects persisted though week 26. Intensive MA had no benefit for knee OA at week 8, although it showed benefits during follow-up.

C1 Beijing Univ Chinese Med, Beijing, Peoples R China; Shanghai Jiao Tong Univ, Shanghai, Peoples R China; Tianjin Univ Tradit Chinese Med, Teaching Hosp 1, Tianjin, Peoples R China; Beijing Hosp Integrated Tradit Chinese & Western, Beijing, Peoples R China; Beijing Univ Chinese Med, Dongfang Hosp, Beijing, Peoples R China; China Acad Chinese Med Sci, Guangan Men Hosp, Beijing, Peoples R China; Hebei Univ Chinese Med, Hebei, Peoples R China; Capital Med Univ, Beijing Friendship Hosp, Beijing, Peoples R China; Beijing Univ Chinese Med, Dongzhimen Hosp, Beijing, Peoples R China; China Acad Chinese Med Sci, Beijing, Peoples R China; Beijing Hosp Tradit Chinese Med, Beijing, Peoples R China

DI 10.1002/art.41584

ID 110

ER

FN Clarivate Analytics Web of science

PT J

AU Wang, T. Q.

AU - Li, L. R.

AU - Tan, C. X.

AU - Yang, J. W.

AU - Shi, G. X.

AU - Wang, L. Q.

AU - Hu, H.

AU - Liu, Z. S.

AU - Wang, J.

AU - Wang, T.

AU - Yuan, Y.

AU - Jia, W. R.

AU - Li, H.

AU - Wang, X. W.

AU - Wu, B.

AU - Tu, J. F.

AU - Liu, C. Z.

PY 2021

TI Effect of Electroacupuncture on Gut Microbiota in Participants With Knee Osteoarthritis

DA 2021/1/1

SO FRONTIERS IN CELLULAR AND INFECTION MICROBIOLOGY

JO FRONTIERS IN CELLULAR AND INFECTION MICROBIOLOGY

VL 11

SN 2235-2988

Z9 Times Cited in Web of Science Core Collection: 22 Total Times Cited: 28 Cited Reference Count: 27 ER -

M3 10.3389/fcimb.2021.597431

N1 WangLi-117

Y2 2025/1/17 16:07:00

N1 2025/2/13 1:16:00

DE electroacupuncture; gut microbiota; knee osteoarthritis; sham acupuncture; effect; CHAIN FATTY-ACIDS; ACUPUNCTURE; PREVALENCE; HEALTH

AB A close relationship between knee osteoarthritis (KOA) and gut microbiota has recently been described. Herein, we aim to investigate the effect of electroacupuncture (EA) on gut microbiota in participants with KOA. We conducted a study of 60 participants with KOA and 30 matched healthy controls (HCs). Sixty participants were allocated to either EA group (n=30) or sham acupuncture (SA) group (n=30). Five obligatory acupoints and three adjunct acupoints were punctured in the EA group. Eight non-acupoints that were separated from conventional acupoints or meridians were used for the SA group. Participants in both groups received 24 sessions within eight weeks. Fecal microbial analyses by 16S ribosomal RNA gene sequencing were carried out after collecting stools at T-0 and T-8 weeks (Four samples with changed defecation habits were excluded). The results showed that both Western Ontario and McMaster Universities Osteoarthritis Index (WOMAC) total score (P=0.043) and NRS score (P=0.002) decreased more in EA group than those in SA group. Moreover, EA could reverse more KOA-related bacteria including Bacteroides, [Eubacterium]_hallii_group, Agathobacter and Streptococcus. The number of significantly different genera between KOA patients and HCs were less after EA treatment than that after SA treatment. This meant that EA modified the composition of the gut microbiome, making it closer to healthy people, while not significantly affecting the microbial diversity. Two genera including Agathobacter (P=0.0163), Lachnoclostridium (P=0.0144) were statistically increased than baseline in EA group (paired Wilcoxon rank sum test). After EA treatment, Bacteroides (P=0.0394) was more abundant and Streptococcus (P=0.0306) was significantly reduced in patients who demonstrated adequate response than in those with inadequate response (Wilcoxon rank-sum test). Spearman correlation test between gut microbe and KOA clinical outcomes indicated that Bacteroides and Agathobacter was negatively correlated with NRS score, WOMAC total score, and WOMAC pain, stiffness and pain scores (P<0.001 or 0.05 or 0.01), while Streptococcus was positively correlated with them (P<0.05 or 0.01). Our study suggests that EA contributes to the improvement of KOA and gut microbiota could be a potential therapeutic target.

C1 Beijing Univ Chinese Med, Sch Acupuncture Moxibust & Tuina, Int Acupuncture & Moxibust Innovat Inst, Beijing, Peoples R China; Beijing Univ Chinese Med, Natl Inst Tradit Chinese Med Constitut & Prevent, Beijing, Peoples R China; Beijing Univ Chinese Med, Dongfang Hosp, Dept Acupuncture & Moxibust, Beijing, Peoples R China; China Acad Chinese Med Sci, Guangan Men Hosp, Dept Acupuncture & Moxibust, Beijing, Peoples R China; Beijing Univ Chinese Med, Dongzhimen Hosp, Dept Acupuncture & Moxibust, Beijing, Peoples R China; China Acad Chinese Med Sci, Inst Acupuncture & Moxibust, Dept Orthoped, Beijing, Peoples R China

DI 10.3389/fcimb.2021.597431

ID 117

ER

FN Clarivate Analytics Web of science

PT J

AU Yu, W. Z.

AU - Huang, C. M.

AU - Ng, H. P.

AU - Lee, Y. C.

PY 2021

TI Distal Acupoints Outperform Proximal Acupoints in Treating Knee Osteoarthritis: A Randomized Controlled Trial

DA 2021/1/1

SO EVIDENCE-BASED COMPLEMENTARY AND ALTERNATIVE MEDICINE

JO EVIDENCE-BASED COMPLEMENTARY AND ALTERNATIVE MEDICINE

VL 2021

SN 1741-427X

Z9 Times Cited in Web of Science Core Collection: 7 Total Times Cited: 7 Cited Reference Count: 35 ER -

M3 10.1155/2021/4827123

N1 YuHuang-142

Y2 2025/1/17 18:01:00

N1 2025/2/13 1:16:00

DE RADIAL PRESSURE PULSE; ACUPUNCTURE; PAIN

AB Objectives. To determine the difference in efficacy between distal and proximal acupoints in treating knee osteoarthritis. Design. Ninety-two eligible participants were randomly assigned into three groups: distal acupoint treatment group (DG), proximal acupoint treatment group (PG), and sham acupuncture control group (SG). Primary and secondary outcomes were compared before and after the intervention. Interventions. A single acupuncture treatment was applied at Quchi (LI11), Shaohai (HT3), and Tianjing (TE10) in DG participants; Yanglingquan (GB34), Yinlingquan (SP9), and Heding (EX-LE2) in PG participants; and Zhongwan (CV12) and Liangmen (ST21) in SG participants. Main outcome measures. The visual analog scale (VAS) and active and passive knee range of motion (ROM) were used primarily to evaluate the treatment efficacy. The radial pulse diagnosis was used as a secondary outcome measure to determine the changes in the spectral energy of the radial pulses. Results. The three groups had significant pain reduction after acupuncture (p<0.05). DG had the greatest difference in pre- and post-VAS scores. Compared with the control group, significant improvement was observed in DG active and passive ROM and in PG passive ROM (p<0.05). The high-frequency spectral energy of the left chi pulse in PG was significantly decreased, while the low-frequency spectral energy of the left cun pulse in PG and the left guan pulse in DG were significantly increased after acupuncture. Conclusions. Distal acupoints provide better pain relief and improve ROM than proximal acupoints in treating knee osteoarthritis. Significant changes in spectral energy were observed in the left cun, guan, and chi pulses, indicating pain relief and blood flow improvement after acupuncture.

C1 China Med Univ, Grad Inst Acupuncture Sci, Taichung 40402, Taiwan; China Med Univ, Sch Postbaccalaureate Chinese Med, Taichung 40402, Taiwan; Singapore Chung Hwa Med Inst, Singapore, Singapore; Singapore Coll Tradit Chinese Med, Singapore, Singapore; China Med Univ Hosp, Dept Acupuncture, Taichung 40402, Taiwan; China Med Univ, Chinese Med Res Ctr, Taichung 40402, Taiwan

DI 10.1155/2021/4827123

ID 142

ER

FN Clarivate Analytics Web of science

PT J

AU Lam, W. C.

AU - Au, K. Y.

AU - Qin, Z. S.

AU - Wu, F. M.

AU - Chong, C. O.

AU - Jiang, F.

AU - He, Y.

AU - Ng, BFL

AU - Yeung, W. F.

AU - Lao, L. X.

AU - Chen, H. Y.

PY 2021

TI Superficial Needling Acupuncture vs Sham Acupuncture for Knee Osteoarthritis: A Randomized Controlled Trial

PG 1286-+

DA 2021/1/1

SO AMERICAN JOURNAL OF MEDICINE

JO AMERICAN JOURNAL OF MEDICINE

VL 134

IS 10

SN 0002-9343

Z9 Times Cited in Web of Science Core Collection: 15 Total Times Cited: 17 Cited Reference Count: 38 ER -

M3 10.1016/j.amjmed.2021.05.002

N1 LamAu-153

Y2 2025/1/18 16:05:00

N1 2025/2/13 1:16:00

DE Acupuncture; knee osteoarthritis; randomized controlled trial; sham control; PHYSICAL FUNCTION; PAIN; HIP; ACUPRESSURE; PREVALENCE; GUIDELINE; EXERCISE; THERAPY; ADULTS

AB BACKGROUND: Acupuncture has been an alternative approach for pain management, but trial evidence is conflicting. METHODS: Eighty-six patients with knee osteoarthritis were randomly assigned in a 1:1 ratio from June 14, 2017, to January 20, 2019, to receive either superficial needling acupuncture treatment or sham acupuncture for 10 sessions over a 4-week treatment period, followed by a 6-week follow-up period. The primary outcome was the change of pain intensity at week 4 measured using a 100-mm visual analogue scale. Secondary outcomes included the Western Ontario and McMaster Universities Osteoarthritis Index and 36Item Short Form Health Survey. RESULTS: At the end of the 4-week treatment period, mean changes in the visual analogue scale were - 30.8 (95% confidence interval [CI], - 38.2 to - 23.0; p < .001) in the acupuncture group and - 26.7 (95% CI, -34.4 to -18.8; P < .001) in the sham group. The difference between the acupuncture group and the sham group was -4.1 (95% CI, -14.4 to 6.2; P = 0.431). At week 10, the difference between the groups was -2.2 (95% CI, -13.1 to 8.8; P = 0.699). There was no statistically significant difference in Western Ontario and McMaster Universities Osteoarthritis Index subscores (pain, stiffness, and physical function) and 36-Item Short Form Health Survey-related outcomes across groups from weeks 2 to 10. The incidence of treatment-related adverse events was 4.4% in the acupuncture group and 0.8% in the sham acupuncture group. All adverse events were classified as mild. CONCLUSION: Acupuncture for 4 weeks is not superior to non-penetrating sham acupuncture. The current study cannot confirm that superficial acupuncture has efficacy for the treatment of knee osteoarthritis. (C) 2021 Elsevier Inc. All rights reserved.

C1 Univ Hong Kong, Hong Kong TB Assoc Chinese Med Clin Cum Training, Hong Kong, Peoples R China; Chinese Univ Hong Kong, Fac Med, Hong Kong Inst Integrat Med, Hong Kong, Peoples R China; Univ Hong Kong, Sch Chinese Med, 10 Sassoon Rd, Hong Kong, Peoples R China; Univ Hong Kong, Dept Stat & Actuarial Sci, Hong Kong, Peoples R China; Hosp Author, Chinese Med Dept, Hong Kong, Peoples R China; Hong Kong Poly Tech Univ, Sch Nursing, Hong Kong, Peoples R China; Virginia Univ Integrat Med, Fairfax, VA USA

DI 10.1016/j.amjmed.2021.05.002

ID 153

ER

FN Clarivate Analytics Web of science

PT J

AU Wang, T. Q.

AU - Wang, Y. J.

AU - Xu, S. T.

PY 2021

TI Discussion on the Theoretical Basis for Acupuncture to Improve Knee Osteoarthritis from "Intestinal Microecology"

PG 295-305

DA 2021/1/1

SO ACUPUNCTURE & ELECTRO-THERAPEUTICS RESEARCH

JO ACUPUNCTURE & ELECTRO-THERAPEUTICS RESEARCH

VL 46

IS 4

SN 0360-1293

Z9 Times Cited in Web of Science Core Collection: 0 Total Times Cited: 0 Cited Reference Count: 34 ER -

M3 10.3727/036012921X16281724938195

N1 WangWang-157

Y2 2025/1/17 18:02:00

N1 2025/2/13 1:16:00

DE theoretical basis; knee osteoarthritis; intestinal microecology; MICROBIOTA

AB The balance of the intestinal microecosystem is essentially consistent with the theory of "Yin Ping Yang Mi" in Chinese medicine. The imbalance of the intestinal microenvironment's homeostasis is closely related to the occurrence of diseases. Acupuncture and moxibustion can improve the disease state by regulating the stability of the intestinal microecology, which has a certain theoretical basis. By exploring the law of the body's meridian circulation, it provides a theoretical basis for studying the regulation of acupuncture on the intestinal microecology of patients with knee osteoarthritis (KOA).

C1 Capital Med Univ, Xuanwu Hosp, Dept Neurol, Beijing 100053, Peoples R China; Hebei Med Univ, Hosp 1, Shijianzhuang 050051, Peoples R China; Natl Univ Singapore, Fac Engn, Singapore 117575, Singapore

DI 10.3727/036012921X16281724938195

ID 157

ER

FN Clarivate Analytics Web of science

PT J

AU Atalay, S. G.

AU - Durmus, A.

AU - Gezginaslan, Ö.

PY 2021

TI SF0 The Effect of Acupuncture and Physiotherapy on Patients with Knee Osteoarthritis: A Randomized Controlled Study

PG E269-E278

DA 2021/1/1

SO PAIN PHYSICIAN

JO PAIN PHYSICIAN

VL 24

IS 3

SN 1533-3159

Z9 Times Cited in Web of Science Core Collection: 13 Total Times Cited: 13 Cited Reference Count: 34 ER -

N1 AtalayDurmus-169

N1 2025/2/13 1:16:00

DE Knee osteoarthritis; acupuncture; physiotherapy; randomized clinical trial; ELECTRICAL NERVE-STIMULATION; QUALITY-OF-LIFE; PAIN; EPIDEMIOLOGY; METAANALYSIS; THERAPY; BLIND; WOMAC; TENS; HIP

AB Background: Osteoarthritis is the most prevalent form of joint disease, and the most common location is the knee. Objectives: The aim of this study was to determine the effect of acupuncture treatment and physiotherapy on pain, physical function, and quality of life (QOL) in patients with knee osteoarthritis (KOA). Study Design: This study was a prospective, randomized, controlled clinical trial. Settings: The research took place in the interventional pain unit of a tertiary center in a university hospital. Methods: One hundred patients with KOA were randomly divided into the acupuncture group and the physiotherapy group. Both treatments were given in 12 sessions over 6 weeks. Thirteen acupuncture points were selected for the knee. Local points were GB34, SP10, SP9, ST36, ST35, ST34, EX-LE2, EXLE5, EXLE4, and distal (distant) points were defined as KI3, SP6, LI4, and ST41. The Visual Analog Scale (VAS) was used to measure pain intensity. The Western Ontario and McMaster Universities Osteoarthritis Index (WOMAC) and the 36-Item Short Form Health Survey (SF-36) were used to determine functional status and health-related QOL, respectively. All patients were evaluated at baseline, after the last treatment, and at the 12-week follow-up period. Results: There was no statistically significant difference between the acupuncture group and physiotherapy group in terms of pain, total WOMAC, and SF-36 levels at baseline, after treatment, and at the 12th week after treatment (P > 0.05). Both treatments significantly improved functional status (acupuncture, from 63.8 +/- 20.81 to 53.72 +/- 19.43; and physiotherapy, from 59.04 +/- 21.49 to 52.28 +/- 19.54; P < 0.05) and decreased the level of pain assessed by VAS (acupuncture, from 8.32 +/- 1.61 to 5.54 +/- 2.34; and physiotherapy, from 7.86 +/- 1.9 to 5.68 +/- 2.42; P < 0.05) at the 12-week follow-up of the study. There was no adverse advent related to therapeutic methods. Limitations: Sham or placebo control groups are lacking in this study. Conclusions: The acupuncture and physiotherapy performed twice weekly for 6 weeks have similar effects with regard to pain, functional status, and QOL. There were no significant differences between the acupuncture and physiotherapy groups in relief of pain, improved functional status, and QOL in the treatment of KOA. Both acupuncture and physiotherapy treatments were found to yield significantly superior results when compared with baseline values.

C1 Ankara City Hosp, Phys Therapy & Rehabil Clin, TR-06800 Ankara, Turkey

ID 169

ER

FN Clarivate Analytics Web of science

PT J

AU Lu, L. B.

AU - Ye, J.

AU - Xiong, J.

AU - Chen, J.

AU - Zhu, S. Y.

AU - Zhong, Z. Y.

AU - Tang, G. H.

AU - Zhou, X. C.

AU - Guo, H.

PY 2021

TI Effectiveness and safety of fire needle for knee osteoarthritis A protocol of systematic review and meta-analysis

DA 2021/1/1

SO MEDICINE

JO MEDICINE

VL 100

IS 3

SN 0025-7974

Z9 Times Cited in Web of Science Core Collection: 1 Total Times Cited: 2 Cited Reference Count: 27 ER -

M3 10.1097/MD.0000000000023962

N1 LuYe-175

N1 2025/2/13 1:16:00

DE efficacy evaluation; fire needle; knee osteoarthritis; meta-analysis; protocols; randomized controlled trials; ACUPUNCTURE; HIP; VALIDATION; EXERCISE; WOMAC

AB Background: There is a lack of curative medical treatment for patients with knee osteoarthritis (KOA). Acupuncture represents an important alternative therapy. In various forms of acupuncture and moxibustion, the fire needle is an indispensable part. Knee osteoarthritis (KOA) is a series of symptoms and signs of knee joint caused by local injury and inflammation and chronic strain of the knee joint resulting in cartilage degeneration of the articular surface and reactive bone loss of the subchondral bone plate. The results of clinical trial indicated that the fire needle therapy has obvious curative effect in treating KOA. This protocol is intended to describe how to collate and accumulate evidence for the current efficient and safe treatment of KOA with fire needle. Methods: Seven electronic databases were used to retrieve the literature for the KOA randomized controlled trials, including 3 English databases (PubMed, EMBASE, the Cochrane Central Register of Controlled Trials [Cochrane Library]), and 4 Chinese databases (Chinese National Knowledge Infrastructure, Chinese VIP Information, Wanfang Database, and Chinese Biomedical Literature Database). This systematic review will include all randomized controlled clinical trials using fire needle therapy for KOA. The observation Index is the Change of Western Ontario and McMaster Universities Osteoarthritis Index Total, first proposed by Bellamy in 1988. The selection of the study will be completed independently by 2 reviewers, extract the data, and evaluate the quality of the study before selecting the title, abstract, and full text. Revman 5.4 software will be used to perform meta-analyses of randomized controlled trials, where risk ratios for dichotomous data and standardized or weighted mean differences for continuous data are the results. Result: The results will be published in a peer-reviewed journal. Conclusion: This systematic review will provide the latest evidence to evaluate the safety and efficacy of fire needle therapy in patients with KOA.

C1 Jiangxi Univ Tradit Chinese Med, 818 Meiling Ave, Nanchang, Jiangxi, Peoples R China; Jiangxi Univ Tradit Chinese Med, Affiliated Hosp, Nanchang, Jiangxi, Peoples R China

DI 10.1097/MD.0000000000023962

ID 175

ER

FN Clarivate Analytics Web of science

PT J

AU Yang, F.

AU - Chen, Y.

AU - Lu, Z. H.

AU - Xie, W. Y.

AU - Yan, S.

AU - Yang, J.

AU - Li, Y. H.

PY 2021

TI Treatment of knee osteoarthritis with acupuncture combined with Chinese herbal medicine: a systematic review and meta-analysis

PG 11430-11444

DA 2021/1/1

SO ANNALS OF PALLIATIVE MEDICINE

JO ANNALS OF PALLIATIVE MEDICINE

VL 10

IS 11

SN 2224-5820

Z9 Times Cited in Web of Science Core Collection: 6 Total Times Cited: 6 Cited Reference Count: 43 ER -

M3 10.21037/apm-21-2565

N1 YangChen-194

N1 2025/2/13 1:16:00

DE Knee osteoarthritis (KOA); acupuncture; traditional Chinese medicine; randomized controlled trial; meta-analysis

AB Background: Many studies have demonstrated that acupuncture combined with Chinese herbal medicine (CHM) effectively treats knee osteoarthritis (KOA), with few side effects. However, few systematic reviews have offered evidence-based support. Here we conducted a meta-analysis on the combination of acupuncture with CHM in treating KOA. Methods: Databases including CNKI, Wanfang, VIP, PubMed, EMBASE, and Cochrane library were systematically searched for articles on the treatment of KOA by acupuncture combined with CHM from the establishment of the database to May 2021. Three researchers independently searched, screened, extracted, and included articles that met the inclusion standards. The primary outcome measure was overall response rate (ORR), and the secondary outcome measures included Visual Analogue Scale ( VAS) score, Western Ontario and McMaster Universities Osteoarthritis Index (WOMAC) score, and Lysholm score. ORR was a binary variable, while other indicators were continuous variables. The quality of literature was assessed with a modified Jadad scale. The RevMan 5.3 software provided by the Cochrane Collaboration was used for statistical analysis. Results: Thirty-three randomized controlled trials involving 3,954 patients were included. Meta-analysis showed that ORR [odds ratio (OR)=5.41; 95% confidence interval (CI): (4.38, 6.68); P<0.00001], VAS score [mean difference (MD)=-1.86; 95% CI: (-2.44, -1.29); P<0.00001], WOMAC score [MD=-13.05; 95% CI: (-21.70, -4.41); P=0.003], and Lysholm score [MD=10.47; 95% CI: (5.21, 15.72); P<0.0001] in the combination group were significantly superior to those in the control group. Discussion: Compared with acupuncture alone or CHM/Western drug alone, acupuncture combined with CHM can effectively alleviate knee pain, improve knee function, and increase the quality of life. Thus, this combination can be used as a conservative treatment for KOA. However, due to the small number of high-quality articles and possible biases in our analysis, our conclusions need to be further verified in more and higher-quality studies.

C1 Hubei Univ Chinese Med, Clin Coll Tradit Chinese Med, Wuhan 430061, Peoples R China

DI 10.21037/apm-21-2565

ID 194

ER

FN Clarivate Analytics Web of science

PT J

AU Li, H. M.

AU - Zheng, Y.

AU - Wang, Y.

AU - Meng, L. C.

AU - Guo, Y. X.

AU - Bian, C.

AU - Liu, P. J.

PY 2021

TI Therapeutic effect of Qinglong tail-wagging acupuncture method in knee osteoarthritis and its influence on inflammatory factors

PG 3206-3213

DA 2021/1/1

SO AMERICAN JOURNAL OF TRANSLATIONAL RESEARCH

JO AMERICAN JOURNAL OF TRANSLATIONAL RESEARCH

VL 13

IS 4

SN 1943-8141

Z9 Times Cited in Web of Science Core Collection: 3 Total Times Cited: 4 Cited Reference Count: 26 ER -

N1 LiZheng-217

N1 2025/2/13 1:16:00

DE Qinglong tail-wagging acupuncture method; the elderly; knee osteoarthritis; clinical efficacy; inflammatory factor; PLATELET-RICH PLASMA; RISK

AB Objectives: This study explored and analyzed the healing effect of Qinglong tail-wagging acupuncture method in therapy of knee osteoarthritis and its influence on inflammatory factors. Methods: 93 elderly patients with acute onset of knee osteoarthritis that hospitalized from March 2017 to June 2019 were admitted and randomly separated into observation group (n=48) and control group (n=45) in line with the method of random data form. The control group was given conventional electro-acupuncture, and the observation group was treated with the qinglong tail-waving acupuncture method. The clinical efficacy, pre- and post-VAS score, Lequesne and WOMAC indexes, and changes in serum inflammatory factor levels pre-and-post treatment were compared between the two sets of subjects. Results: The overall response rate of clinical treatment in observation group was remarkably higher than that of the control group (P < 0.05). Besides, the VAS, Lequesne as well as WOMAC scores of two groups after treatment were significantly reduced than those in pre-treatment (P < 0.05), and the indexes in observation group were remarkably lower than those in control group (P < 0.05). In addition, the serum IL-6, NO and TNF-alpha concentration of the two sets of subjects in post-treatment reduced significantly than that in pre-treatment (P < 0.05), and the serum IL-6, NO and TNF-alpha concentration in observation group were remarkably decreased than those in control group (P < 0.05). Conclusion: The acupuncture method of Qinglong tail-wagging has good healing effect on remedy of elderly knee osteoarthritis. The curative method is able to improve the patients' function of knee joint and reduce the concentration of serum inflammatory factor in patients with knew osteoarthritis.

C1 Peoples Hosp Sishui Cty, Dept Rehabil, Sishui 272200, Peoples R China; Jining Fourth Peoples Hosp, Dept Med Secur, Jining 272137, Peoples R China; Jining First Peoples Hosp, Dept Acupuncture & Physiotherapy, Jining 272011, Peoples R China; Jining Hosp Tradit Chinese Med, Dept Acupuncture & Massage, 3 Huancheng North Rd, Jining 272000, Shandong, Peoples R China

ID 217

ER

FN Clarivate Analytics Web of science

PT J

AU Yuan, X. C.

AU - Yan, X. J.

AU - Tian, L. X.

AU - Guo, Y. X.

AU - Zhao, Y. L.

AU - Baba, S. S.

AU - Wang, Y. Y.

AU - Liang, L. L.

AU - Jia, H.

AU - Xu, L. P.

AU - Li, L.

AU - Lin, H.

AU - Huo, F. Q.

PY 2021

TI 5-HT<sub>7</sub> Receptor Is Involved in Electroacupuncture Inhibition of Chronic Pain in the Spinal Cord

DA 2021/1/1

SO FRONTIERS IN NEUROSCIENCE

JO FRONTIERS IN NEUROSCIENCE

VL 15

SN 1662-453X

Z9 Times Cited in Web of Science Core Collection: 6 Total Times Cited: 6 Cited Reference Count: 26 ER -

M3 10.3389/fnins.2021.733779

N1 YuanYan-221

N1 2025/2/13 1:16:00

DE knee osteoarthritis (KOA); 5-HT7 receptor; GABA(A) receptor; chronic pain; electroacupuncture analgesia (EAA); OSTEOARTHRITIS; MODULATION; ACTIVATION; KNEE

AB Knee osteoarthritis (KOA) is a common and disabling condition characterized by attacks of pain around the joints, and it is a typical disease that develops chronic pain. Previous studies have proved that 5-HT1, 5-HT2, and 5-HT3 receptors in the spinal cord are involved in electroacupuncture (EA) analgesia. The 5-HT7 receptor plays antinociceptive role in the spinal cord. However, it is unclear whether the 5-HT7 receptor is involved in EA analgesia. The 5-HT7 receptor is a stimulatory G-protein (Gs)-coupled receptor that activates adenylyl cyclase (AC) to stimulate cyclic adenosine monophosphate (cAMP) formation, which in turn activates protein kinase A (PKA). In the present study, we found that EA significantly increased the tactile threshold and the expression of the 5-HT7 receptor in the dorsal spinal cord. Intrathecal injection of 5-HT7 receptor agonist AS-19 mimicked the analgesic effect of EA, while a selective 5-HT7 receptor antagonist reversed this effect. Moreover, intrathecal injection of AC and PKA antagonists prior to EA intervention prevented its anti-allodynic effect. In addition, GABA(A) receptor antagonist bicuculline administered (intrathecal, i.t.) prior to EA intervention blocked the EA effect on pain hypersensitivity. Our data suggest that the spinal 5-HT7 receptor activates GABAergic neurons through the Gs-cAMP-PKA pathway and participates in EA-mediated inhibition of chronic pain in a mouse model of KOA.

C1 Xi An Jiao Tong Univ, Sch Basic Med Sci, Inst Neurosci, Translat Med Inst,Hlth Sci Ctr, Xian, Peoples R China; Xi An Jiao Tong Univ, Key Lab Environm & Genes Related Dis, Minist Educ, Xian, Peoples R China; Wenzhou Med Univ, Affiliated Hosp 2, Dept Anesthesiol, Key Lab Anesthesiol Zhejiang Prov, Wenzhou, Peoples R China

DI 10.3389/fnins.2021.733779

ID 221

ER

FN Clarivate Analytics Web of science

PT J

AU Shi, X. W.

AU - Yu, W. J.

AU - Zhang, W.

AU - Wang, T.

AU - Battulga, O.

AU - Wang, L. J.

AU - Guo, C. Q.

PY 2021

TI A comparison of the effects of electroacupuncture versus transcutaneous electrical nerve stimulation for pain control in knee osteoarthritis: a Bayesian network meta-analysis of randomized controlled trials

PG 163-174

DA 2021/1/1

SO ACUPUNCTURE IN MEDICINE

JO ACUPUNCTURE IN MEDICINE

VL 39

IS 3

SN 0964-5284

Z9 Times Cited in Web of Science Core Collection: 6 Total Times Cited: 7 Cited Reference Count: 39 ER -

M3 10.1177/0964528420921193

N1 ShiYu-230

Y2 2025/1/17 16:05:00

N1 2025/2/13 1:16:00

DE electroacupuncture; knee osteoarthritis; meta-analysis; pain; transcutaneous electrical nerve stimulation; NONSTEROIDAL ANTIINFLAMMATORY DRUGS; DOUBLE-BLIND; DICLOFENAC; MANAGEMENT; HIP; PREVALENCE; CELECOXIB; PLACEBO; RELIEF; RISK

AB Background To compare the effectiveness of electroacupuncture (EA) and transcutaneous electrical nerve stimulation (TENS) for pain control in knee osteoarthritis (KOA). Methods Four English (MEDLINE, EMBASE, Cochrane Library and Web of Science) and three Chinese (China Science Journal Citation Report (VIP), Wanfang and China National Knowledge Infrastructure (CNKI)) language databases were searched for eligible randomized controlled trials (RCTs), comparing four approaches: EA, TENS, medication and sham/placebo controls. The primary outcome was pain intensity, measured by visual analogue scale (VAS), numeric-rating scale (NRS) or Western Ontario and McMaster Universities Osteoarthritis Index (WOMAC) scale. Classic pairwise and Bayesian network meta-analyses were conducted to integrate the treatment efficacy/effectiveness through direct and indirect evidence. Results Thirteen studies were included. In the direct meta-analyses, there was no statistically significant overall effect of EA (mean difference (MD) -4.77, 95% confidence interval (CI) -12.51 to 2.96), while the overall effects of high-frequency transcutaneous electrical nerve stimulation (H-TENS) (MD -16.63, 95% CI -24.57 to -8.69) and medication (MD -7.12, 95% CI -12.07 to -2.17) were statistically significant. In the network meta-analyses, the relative effect of the EA and H-TENS groups (MD 5.07, 95% CI -11.33 to 21.93) on pain control did not differ. Meanwhile, H-TENS demonstrated the highest probability of being the first best treatment, and EA had the second highest probability. Conclusion The present analysis indicated that both EA and TENS exert significant pain relieving effects in KOA. Among the four treatments, H-TENS was found to be the optimal treatment choice for the management of KOA pain in the short-term, and EA the second best treatment option. Given that the application of TENS is recommended by various international guidelines for the treatment of KOA, EA may also represent a potentially effective non-pharmacologic therapy.

C1 Beijing Univ Chinese Med, Affiliated Hosp 3, Dept Massage, Beijing, Peoples R China; Beijing Univ Chinese Med, Affiliated Hosp 3, Dept Pediat, Beijing, Peoples R China; Beijing Univ Chinese Med, Sch Acupuncture Moxibust & Tuina, 11 East North Third Ring Rd, Beijing 100029, Peoples R China

DI 10.1177/0964528420921193

ID 230

ER

FN Clarivate Analytics Web of science

PT J

AU Wang, H. Y.

AU - Sun, J. X.

AU - Yu, X. Y.

AU - He, Y.

PY 2021

TI Acupoint injection in improving pain and joint function of knee osteoarthritis patients A protocol for systematic review and meta-analysis

DA 2021/1/1

SO MEDICINE

JO MEDICINE

VL 100

IS 12

SN 0025-7974

Z9 Times Cited in Web of Science Core Collection: 0 Total Times Cited: 0 Cited Reference Count: 27 ER -

M3 10.1097/MD.0000000000024997

N1 WangSun-265

Y2 2025/1/17 16:02:00

N1 2025/2/13 1:16:00

DE acupoint injection; knee osteoarthritis; meta-analysis; protocol; systematic review

AB Background: Knee osteoarthritis is a common chronic progressive disease, which seriously affects the quality of life of the middle-aged and elderly, and even leads to disability. More and more evidence shows that acupoint injection is beneficial to the clinical treatment of knee osteoarthritis, but there are differences in the efficacy of different acupoints and injection drugs, and there is no systematic review to assess this therapy at present. The purpose of this study is to systematically evaluate the efficacy and safety of acupoint injection in improving pain and joint function in patients with knee osteoathrosis. Methods: According to the retrieval strategy, we will search from CNKI, Wanfang, VIP, Chinese Biomedical Science, PubMed, Embase, Web of Science and the Cochrane Library for randomized controlled trials of acupoint injection in the treatment of knee osteoarthritis from the establishment of the database to February 2021. The study will be screened according to the inclusion and exclusion criteria, and the Cochrane risk bias assessment tool will be used to evaluate the quality of the study. Revman 5.4 software is used for meta-analysis. Results: This study will evaluate the efficacy of acupoint injection in the treatment of knee osteoarthritis by evaluating the total effective rate, the degree of pain relief, joint function score, adverse reactions, and so on. Conclusion: This study will provide reliable evidence-based basis for the clinical application of acupoint injection in the treatment of knee osteoarthritis. Ethics and dissemination Private information from individuals will not be published. This systematic review also does not involve endangering participant rights. Ethical approval will not be required. The results may be published in a peer-reviewed journal or disseminated at relevant conferences. OSF Registration number-doi: 10.17605/OSF.IO/M5FTK.

C1 Chinese Med Hosp Linyi City, 211 Jiefang Rd, Linyi 276000, Shandong, Peoples R China

DI 10.1097/MD.0000000000024997

ID 265

ER

FN Clarivate Analytics Web of science

PT J

AU Li, X. J.

AU - Yu, W.

AU - Li, H. B.

AU - Wang, B. Y.

AU - Xu, J.

PY 2021

TI Prospective, Single-Center Comparison of Transcranial Direct Current Stimulation Plus Electroacupuncture and Standard Analgesia in Patients After Total Knee Arthroplasty: Effect on Rehabilitation and Functional Recovery

DA 2021/1/1

SO MEDICAL SCIENCE MONITOR

JO MEDICAL SCIENCE MONITOR

VL 27

SN 1643-3750

Z9 Times Cited in Web of Science Core Collection: 3 Total Times Cited: 3 Cited Reference Count: 31 ER -

M3 10.12659/MSM.930363

N1 LiYu-413

Y2 2025/1/18 21:56:00

N1 2025/2/13 1:16:00

DE Arthroplasty, Replacement, Knee; Chronic Pain; Electroacupuncture; Rehabilitation; Transcranial Magnetic Stimulation; POSTOPERATIVE PAIN

AB Background: The aim of this prospective study was to compare transcranial direct current stimulation (tDCS) plus electroacupuncture with standard analgesia in patients after total knee arthroplasty (TKA) to determine the effects on rehabilitation and functional recovery. Material/Methods: Eighty patients with osteoarthritis of the knee who underwent TKA were included in the study. They were divided into experimental (n=40) and control groups (n=40) according to postoperative analgesia method. The control group received multimodal analgesia after TKA and the experimental group received additional tDCS plus electroacupuncture. Postoperative pain, knee function, and quality of life were compared between the 2 groups. Results: Compared with the control group, the experimental group had significantly lower visual analog scale scores at 3 and 7 days and 3 and 6 weeks after TKA (P<0.05). At 6 weeks after TKA, knee injury and osteoarthritis outcome and Hospital for Special Surgery scores and maximum knee flexion in the experimental group were significantly better than those in the control group (P<0.05). In the experimental group compared with the control group, the Short Form-36 Health Survey score also was significantly increased (P<0.05). Conclusions: The findings from this study showed that tDCS plus electroacupuncture effectively reduced pain after TKA and improved rehabilitation and functional recovery.

C1 Xuzhou Med Univ, Dept Rehabil Med, Affiliated Huaian Hosp, Peoples Hosp Huaian 2, Huaian, Jiangsu, Peoples R China; Huaian Rehabil Hosp, Dept Rehabil Med, Huaian, Jiangsu, Peoples R China; Nanjing Med Univ, Dept Rehabil Med, Lianshui Cty Peoples Hosp, Kangda Coll, Huaian, Jiangsu, Peoples R China

DI 10.12659/MSM.930363

ID 413

ER

FN Clarivate Analytics Web of science

PT J

AU Shi, G. X.

AU - Tu, J. F.

AU - Wang, T. Q.

AU - Yang, J. W.

AU - Wang, L. Q.

AU - Lin, L. L.

AU - Wang, Y.

AU - Li, Y. T.

AU - Liu, C. Z.

PY 2020

TI Effect of Electro-Acupuncture (EA) and Manual Acupuncture (MA) on Markers of Inflammation in Knee Osteoarthritis

PG 2171-2179

DA 2020/1/1

SO JOURNAL OF PAIN RESEARCH

JO JOURNAL OF PAIN RESEARCH

VL 13

SN 1178-7090

Z9 Times Cited in Web of Science Core Collection: 41 Total Times Cited: 50 Cited Reference Count: 30 ER -

M3 10.2147/JPR.S256950

N1 ShiTu-13

Y2 2025/1/20 13:41:00

N1 2025/2/13 1:16:00

DE knee osteoarthritis; electro-acupuncture; manual acupuncture; inflammation; PAIN; EXPRESSION; CARTILAGE; CHONDROCYTES; MODULATION; OUTCOMES

AB Background: Inflammation plays a significant role in the pathogenesis of knee osteoarthritis (KOA). Although both electro-acupuncture (EA) and manual acupuncture (MA) are known to influence systemic inflammation, little is known about the potential changes in inflammation as a working mechanism of EA and MA in KOA. Methods: Data from the Acupuncture for Knee Osteoarthritis Trial (ATKOA) were used. Serum concentrations of inflammatory factors (tumor necrosis factor-alpha (TNF-alpha), interleukin-1 beta (IL-1 beta), IL-6, IL-8, IL-18, IL-4, IL-10, IL-13, IL-15, IL-17, monocyte chemotactic protein-1 (MCP-1), CC-chemokine ligand 5 (CCL5), and cartilage degradation biomarkers (matrix metalloproteinase-1 MMP-1, MMP-3, MMP-13 and cartilage oligomeric matrix protein COMP)) were measured at baseline and after 8 weeks of treatment. Clinical outcomes were valid and reliable self-reported pain and function measures for osteoarthritis using the Western Ontario and McMaster Universities Osteoarthritis Index (WOMAC) and visual analogue scale (VAS) at baseline and post-treatment. Results: Both 8-weeks EA and MA significantly reduced pro-inflammatory cytokines (TNF alpha, IL-1 beta), and cartilage degradation biomarkers (MMP-3, MMP-13) significantly increased the anti-inflammatory cytokine IL-13 compared with pre-treatment (p<0.05). Further, the reduction of TNF-alpha was more significant in EA when compared to MA (p=0.046). While there was no significant difference between groups in cytokines IL-1 beta (p=0.102), MMP-3 (p=0.113), MMP-13 (p=0.623) or IL-13 (p=0.935). Moreover, in both EA and MA, the effect of acupuncture on the VAS and WOMAC function scale after 8 weeks is clinically important, although no significant differences were found between groups. Conclusion: Eight weeks of both EA and MA seem to provide improvement in pain relief and function among individuals with mild to moderate knee OA. This benefit is partly mediated by changes of major inflammatory factors TNF-alpha, IL-1 beta and IL-13.

C1 Capital Med Univ, Dept Acupuncture & Moxibust, Beijing Hosp Tradit Chinese Med, Beijing, Peoples R China; Beijing Univ Chinese Med, Sch Acupuncture Moxibust & Tuina, Beijing, Peoples R China

DI 10.2147/JPR.S256950

ID 13

ER

FN Clarivate Analytics Web of science

PT J

AU Kim, D.

AU - Jang, S.

AU - Park, J.

PY 2020

TI Electroacupuncture and Manual Acupuncture Increase Joint Flexibility but Reduce Muscle Strength

DA 2020/1/1

SO HEALTHCARE

JO HEALTHCARE

VL 8

IS 4

SN 2227-9032

Z9 Times Cited in Web of Science Core Collection: 6 Total Times Cited: 6 Cited Reference Count: 45 ER -

M3 10.3390/healthcare8040414

N1 KimJang-16

N1 2025/2/13 1:16:00

DE range of motion; joint position sense; quadriceps; central activation ratio; ELECTRICAL NERVE-STIMULATION; KNEE OSTEOARTHRITIS; SHAM ACUPUNCTURE; PAIN; CRYOTHERAPY; ACTIVATION; POSITION; STRETCH; TRIAL; TENS

AB The objective of this study was to investigate the immediate effects of electroacupuncture and manual acupuncture on hip flexion range of motion (ROM), knee joint (flexion replication at 15 degrees and 45 degrees) and quadriceps (strength and activation) function. Forty-five neurologically healthy adults participated in this randomized controlled laboratory study. Straight leg raise test, modified Thomas test, and hip abductors strength test were performed to determine acupoints. Afterwards, one of three 15-min treatments (control-no treatment, electroacupuncture, or manual acupuncture) was randomly applied using determined acupoints. Measurements (hip flexion ROM, and knee joint and quadriceps function) were recorded at baseline, and at 0, 20, and 40 min post treatment. Both electroacupuncture (4.0 degrees, ES = 0.41) and manual acupuncture (5.4 degrees, ES = 0.95) treatment immediately increased hip flexion ROM, and the increased values persisted for 40-min (p = 0.01). Knee flexion replication (at 15 degrees: p = 0.17; 45 degrees: p = 0.19) and quadriceps activation (p = 0.71) did not change at any of the time points. Post-treatment, both electroacupuncture and manual acupuncture decreased quadriceps strength at 0-min (electroacupuncture: 9.2%, p < 0.0001, ES = 0.60) and 40-min (electroacupuncture: 7.3%, p = 0.005, ES = 0.55; manual acupuncture: 8.7%, p = 0.01, ES = 0.54). A single session of either electroacupuncture or manual acupuncture treatment (selected acupoints based on physical examination) may immediately improve joint flexibility but reduce muscle strength.

C1 Woosong Univ, Dept Sports Sci & Rehabil, Daejeon 34606, South Korea; Bareun Korean Med Clin, Seoul 05616, South Korea; Kyung Hee Univ, Athlet Training Lab, Dept Sports Med, Yongin 17104, South Korea

DI 10.3390/healthcare8040414

ID 16

ER

FN Clarivate Analytics Web of science

PT J

AU Wang, T. Q.

AU - Li, Y. T.

AU - Wang, L. Q.

AU - Shi, G. X.

AU - Tu, J. F.

AU - Yang, J. W.

AU - Hou, Y. Q.

AU - Lin, L. L.

AU - Sun, N.

AU - Zhao, J. J.

AU - Hou, H. K.

AU - Liu, C. Z.

PY 2020

TI Electroacupuncture versus manual acupuncture for knee osteoarthritis: a randomized controlled pilot trial

PG 291-300

DA 2020/1/1

SO ACUPUNCTURE IN MEDICINE

JO ACUPUNCTURE IN MEDICINE

VL 38

IS 5

SN 0964-5284

Z9 Times Cited in Web of Science Core Collection: 18 Total Times Cited: 18 Cited Reference Count: 37 ER -

M3 10.1177/0964528419900781

N1 WangLi-22

Y2 2025/1/20 13:41:00

N1 2025/2/13 1:16:00

DE electroacupuncture; knee osteoarthritis; manual acupuncture; randomized controlled trial; ALTERNATIVE MEDICINE; HEALTH SURVEY; ARTHRITIS; INTERVENTION; PREVALENCE; OUTCOMES; THERAPY; PATIENT; WOMAC; HIP

AB Objective: We aimed to explore the feasibility of evaluating the comparative effectiveness and safety of electroacupuncture (EA) relative to manual acupuncture (MA) for the treatment of knee osteoarthritis (KOA). Methods: A multicenter randomized controlled clinical trial was conducted in Beijing from September 2017 to January 2018. A total of 60 participants with KOA were randomly allocated to either EA (n = 30) or MA (n = 30) groups. Participants in the EA group were treated with EA at six to seven local traditional acupuncture points orah shipoints, and two to three distal points. Participants in the MA group had the same schedule as the EA group except that the electrical apparatus featured a working power indicator without actual current output, constituting a sham EA procedure, in order to blind participants. Both groups received 24 sessions over 8 weeks. The primary outcome was response rate, defined as a change of > 50% from baseline in the total scores of the Western Ontario and McMaster Universities Osteoarthritis Index (WOMAC) after 8 weeks. Secondary outcomes included pain, stiffness, function, quality of life, and acupuncture-related adverse events (AEs) at 4 and 8 weeks. Results: Of 60 participants randomized, 53 (88%) completed the study. Response rates were 43% for the EA group and 30% for the MA group by the intention-to-treat analysis. Although significant differences were observed in WOMAC pain, stiffness, and function scores within both groups, between-group differences at 8 weeks did not reach statistical significance (odds ratio = 1.75 (95% confidence interval = 0.593-5.162)). Rates of AEs were low and similarly distributed between groups. Conclusion: Both EA and MA interventions in KOA were feasible and appeared safe. Whether or not EA may have a stronger impact on pain and function requires further evaluation through larger, adequately powered, randomized controlled trials.

C1 Beijing Univ Chinese Med, Acupuncture Res Ctr, Sch Acupuncture Moxibust & Tuina, 11 Bei San Huan Dong Lu, Beijing 100029, Peoples R China; Capital Med Univ, Dept Acupuncture & Moxibust, Beijing Hosp Tradit Chinese Med, Beijing, Peoples R China; Chengdu Univ Tradit Chinese Med, Acupuncture & Tuina Sch, Chengdu, Peoples R China; Capital Med Univ, Dept Acupuncture & Moxibust, Beijing Friendship Hosp, Beijing, Peoples R China; Peking Univ, Dept Acupuncture & Moxibust, Beijing Jishuitan Hosp, Med Coll 4, Beijing, Peoples R China

DI 10.1177/0964528419900781

ID 22

ER

FN Clarivate Analytics Web of science

PT J

AU Zhang, Q. F.

AU - Fang, J. Q.

AU - Chen, L. F.

AU - Wu, J. Y.

AU - Ni, J.

AU - Liu, F.

AU - Sun, J.

PY 2020

TI Different kinds of acupuncture treatments for knee osteoarthritis: a multicentre, randomized controlled trial

DA 2020/1/1

SO TRIALS

JO TRIALS

VL 21

IS 1

SN 1745-6215

Z9 Times Cited in Web of Science Core Collection: 4 Total Times Cited: 4 Cited Reference Count: 37 ER -

M3 10.1186/s13063-019-4034-8

N1 ZhangFang-26

N1 2025/2/13 1:16:00

DE Acupuncture; Knee osteoarthritis; Electro-acupuncture; Warm-needling; Sham acupuncture; Mild moxibustion; Multicentre randomized controlled trial; HIP; RECOMMENDATIONS; ELECTROACUPUNCTURE; METAANALYSIS; THERAPY; PAIN

AB Introduction Knee osteoarthritis (KOA) is a chronic disease with symptoms of persistent pain or resting pain, joint stiffness, numbness, limitation of activity and even disability, with significant associated costs and effects on individuals' life quality. The use of acupuncture for the management of chronic pain is receiving increasing recognition from both the public and professionals. The aim of this study is to identify the effects of three commonly used acupuncture treatments for KOA. Methods/analysis In a prospective trial involving six hospitals in Zhejiang Province (China), 360 patients with KOA will be included. Eligible patients will be randomized into six groups: Acupuncture, Electro-acupuncture, Mild moxibustion, Warm-needling, Sham acupuncture and Celebrex treatment. Twelve treatment sessions will be performed over a 4-week period. The primary outcome will be the visual analogue scale and Western Ontario and McMaster Universities Osteoarthritis Index (WOMAC) function scores (the average of the past 3 days) at weeks 2 and 4 and at 3-month and 6-month follow-up. Secondary outcome measures will be as follows: the WOMAC pain score and WOMAC stiffness score (the average of the past 3 days); the Physical Activity Scale of the Elderly (PASE); knee joint swelling measurement; the WHO Quality Of Life-BREF (WHOQOL-BREF) life quality scale; and the incidence of adverse events.

C1 Zhejiang Chinese Med Univ, Clin Med Coll 3, 548 Binwen Rd, Hangzhou, Zhejiang, Peoples R China; Zhejiang Chinese Med Univ, Dept Acupuncture, Affiliated Hosp 3, 548 Binwen Rd, Hangzhou, Zhejiang, Peoples R China; Zhejiang Tradit Chinese Med Univ, Dept Acupuncture, Affiliated Hosp 3, 219 Moganshan Rd, Hangzhou, Zhejiang, Peoples R China; Hangzhou Red Cross Hosp, Dept Acupuncture, 208 Huanchengdong Rd, Hangzhou, Zhejiang, Peoples R China

DI 10.1186/s13063-019-4034-8

ID 26

ER

FN Clarivate Analytics Web of science

PT J

AU Zhou, X.

AU - Xiang, K. M.

AU - Yuan, X. Y.

AU - Wang, Z. P.

AU - Li, K. L.

PY 2020

TI A comparison of the effects of acupoint injection combined with hyaluronic acid versus isolated hyaluronic acid for knee osteoarthritis A protocol for systematic review and meta-analysis of randomized controlled trials

DA 2020/1/1

SO MEDICINE

JO MEDICINE

VL 99

IS 47

SN 0025-7974

Z9 Times Cited in Web of Science Core Collection: 1 Total Times Cited: 2 Cited Reference Count: 27 ER -

M3 10.1097/MD.0000000000023262

N1 ZhouXiang-35

N1 2025/2/13 1:16:00

DE acupoint injection; hyaluronic acid; knee osteoarthritis; protocol; randomized controlled trial; systematic review; ACUPUNCTURE; PATHOPHYSIOLOGY; RISK; PAIN; HIP

AB Background: Knee osteoarthritis (KOA) is a kind of degenerative osteoarthropathy, which causes joint pain and limited mobility, seriously affects the quality of life of the patient. Traditional Chinese Medicine acupuncture and moxibustion has been widely used to treat KOA, and acupoint injection is 1 of the acupuncture treatment methods. The purpose of this work is to evaluate the effectiveness of Acupoint injection combined with Hyaluronic Acid injection compared with isolated Hyaluronic Acid injection for KOA. Methods: We will search articles in 7 electronic databases including Chinese National Knowledge Infrastructure, Wanfang Data, Chinese Scientific Journals Database, Chinese databases SinoMed, PubMed, Embase, and Cochrane Library databases. All the publications, with no time restrictions, will be searched without any restriction of language and status, the time from the establishment of the database to October 2020. Two reviewers will independently assess the quality of the selected studies, NoteExpress and Excel software will be used to extract data, and the content will be stored in an electronic chart. Different researchers will separately screen the titles and abstracts of records acquired potential eligibility which comes from the electronic databases. Full-text screening and data extraction will be conducted afterward independently. Statistical analysis will be conducted using RevMan 5.4 software. Results: This study will evaluate the efficacy and safety of Acupoint injection combined with Hyaluronic Acid injection compared with isolated Hyaluronic Acid injection in the treatment of KOA, to provide high-quality, evidence-based clinical recommendations. Conclusion: This study will provide reliable evidence on whether Acupoint injection combined with Hyaluronic Acid injection compared with isolated Hyaluronic Acid injection is more effective in treating KOA.

C1 Taizhou Tradit Chinese Med Hosp, 278 Zhongshan West Rd, Tatehou City, Zhejiang, Peoples R China

DI 10.1097/MD.0000000000023262

ID 35

ER

FN Clarivate Analytics Web of science

PT J

AU Li, R. R.

AU - Sun, J.

AU - Hu, H. T.

AU - Zhang, Q. F.

AU - Sun, R. H.

AU - Zhou, S. N.

AU - Zhang, H. J.

AU - Fang, J. Q.

PY 2020

TI Research Trends of Acupuncture Therapy on Knee Osteoarthritis from 2010 to 2019: A Bibliometric Analysis

PG 1901-1913

DA 2020/1/1

SO JOURNAL OF PAIN RESEARCH

JO JOURNAL OF PAIN RESEARCH

VL 13

SN 1178-7090

Z9 Times Cited in Web of Science Core Collection: 55 Total Times Cited: 58 Cited Reference Count: 50 ER -

M3 10.2147/JPR.S258739

N1 LiSun-40

N1 2025/2/13 1:16:00

DE acupuncture; knee osteoarthritis; bibliometric analysis; CiteSpace; CLINICAL-PRACTICE GUIDELINES; RISK-FACTORS; PAIN; MANAGEMENT; EXERCISE; METAANALYSIS

AB Background: Acupuncture has been widely applied to relieve knee osteoarthritis (KOA) in many countries. However, the bibliometric analysis of the global use of acupuncture on KOA is largely unknown. Therefore, this study aimed to explore the current status and trends of the global use of acupuncture on KOA in recent 10 years by using CiteSpace (5.6.R3). Methods: Publications regarding acupuncture therapy for treating KOA between 2010 and 2019 were extracted from the Web of Science database. CiteSpace was used to analyze the number of publications, countries, institutions, journals, authors, cited references and keywords by using standard bibliometric indicators. Results: A total of 343 publications were retrieved from 2010 to 2019. The total number of publications continually increased over the past four years, and the most active journals, countries, institutions and authors in the field of acupuncture therapy on patients with KOA were identified. The Evid Based Complement Alternat Med (28) was the most prolific journal, and the Ann Intern Med (202) was the most cited journal. The most productive country and institution in this field was China (115) and University of York (18), respectively. Hugh Macpherson (18) was the most prolific author and Witt C ranked the first in the cited authors. In the ranking of frequency in cited reference, the first article was published by Scharf HP (54). The keyword of 'randomized controlled trial' ranked first for research developments with the highest citation burst (3.9486). Besides, there were three main frontiers in keywords for KOA research, including 'research method, 'age' and 'measure of intervention'. Conclusion: The findings from this bibliometric study provide current status and trends in clinical research of acupuncture therapy on patients with KOA over the past ten years, which may help researchers identify hot topics and explore new directions for future research in this field.
[truncated: 456,391 more chars]
